# A versatile strategy for oligonucleotide functionalization via on-support phosphitylation

**DOI:** 10.1038/s41557-026-02214-6

**Published:** 2026-07-22

**Authors:** Ayan Dasgupta, Sebastian Golojuch, Li Xiao, Nour Eddine Fahmi, Edward A. Anderson, Tom Brown

**Affiliations:** 1 Chemistry Research Laboratory, Department of Chemistry, University of Oxford, Oxford, UK; 2 Eli Lilly and Company, Lilly Corporate Center, Indianapolis, IN USA

**Keywords:** Nucleic acids, Solid-phase synthesis

## Abstract

Chemically modified nucleic acids such as antisense oligonucleotides, small interfering RNA and CRISPR guide RNA have emerged as transformative modalities in modern medicine, providing new strategies for the treatment of genetic and metabolic disorders, viral infections and cancer. However, their application is limited by challenges related to intracellular delivery and targeting, advances in which would benefit from a broader range of methods for chemical functionalization. Here we describe a streamlined approach using inexpensive, bench-stable alcohols or nucleosides that enables site-selective modification at the 5′ and 3′ termini and at internal sites of synthetic oligonucleotides. Our strategy offers a conceptually straightforward solution to this problem through the in situ generation of phosphoramidites on the solid-supported oligonucleotide. The methodology displays high coupling efficiency, high functional-group compatibility with azides, amines and thiols, and efficient recovery of excess alcohols/monomers, thus offering a robust platform for advancing and diversifying therapeutic oligonucleotide modification.

**Figure:**
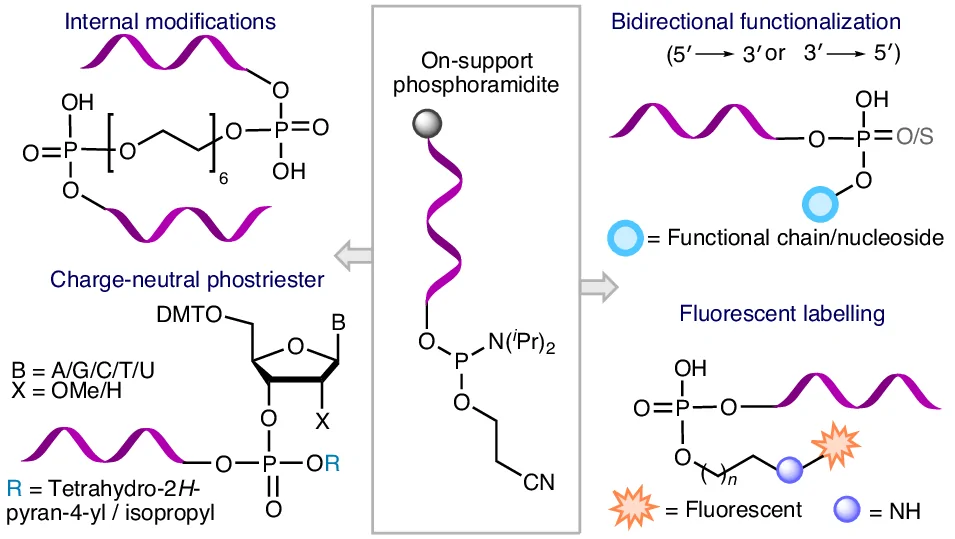

## Abstract (web summary)

Oligonucleotide modification largely relies on unstable, expensive phosphoramidite reagents, limiting the diversity and efficiency of chemical functionalization. Now, a site-selective modification of synthetic oligonucleotides is reported via the in situ generation of phosphoramidites on the solid-supported oligonucleotide. This approach enables cost-effective and versatile modification of oligonucleotides, including terminal and internal modifications.

## Main

**Fig. 1:**
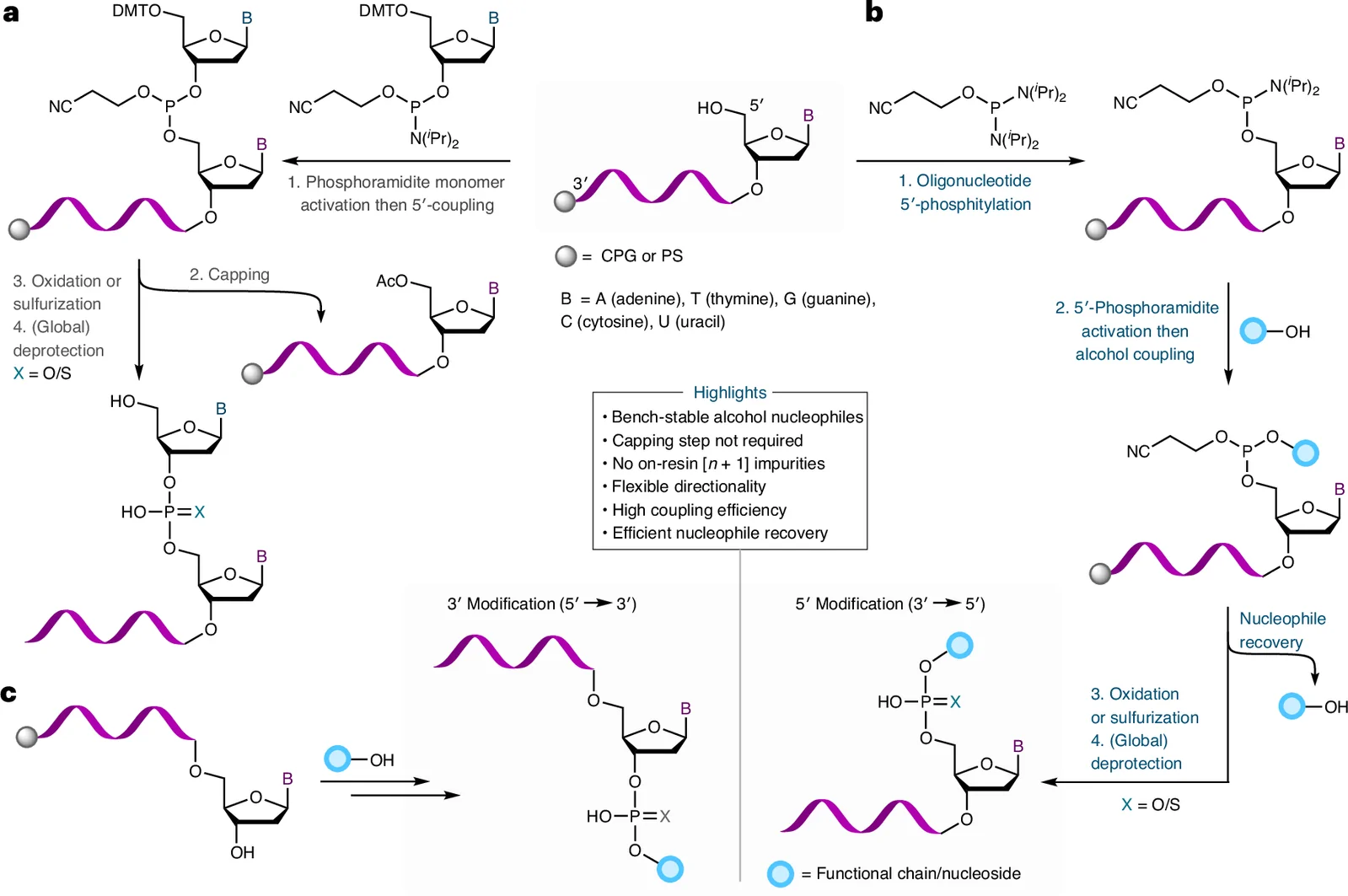
Limitations of existing methods and advantages of the current strategy. **a**, The standard synthetic cycle for oligonucleotide synthesis requires the use of unstable nucleoside phosphoramidite reagents and additional capping steps. **b**, The strategy described in this work: phosphitylation of the solid-supported oligonucleotide enables the introduction of a range of modifications using stable alcohol nucleophiles, including nucleosides, and also offers the practical advantage that any unreacted alcohol/nucleosides can be readily recovered. **c**, The strategy also enables functionalization of the 3′ terminus of an oligonucleotide in the ‘reverse’ synthesis direction. ^*i*^Pr, isopropyl.

Oligonucleotides are essential tools in molecular biology, chemical biology, sequencing, diagnostics and therapeutics^1–6^, enabling precise control over gene expression and molecular recognition^7–9^. Strategic chemical modifications, both internally and at oligonucleotide termini, are essential to enhance stability, fine-tune hybridization and introduce functional handles for conjugation of various labels, haptens, and targeting and delivery agents^6,10–13^. Such modifications are crucial for molecular biology applications, and for next-generation therapeutics using antisense oligonucleotides and small interfering RNA (siRNA)^1,14,15^. For more than four decades, phosphoramidite chemistry has represented the gold standard in oligonucleotide synthesis, inspiring a plethora of critical applications^16,17^. The standard synthesis comprises four steps (Fig. 1a): coupling of the 5′-hydroxyl of the growing oligo chain with a 3′-phosphoramidite nucleoside monomer; capping of unreacted 5′-hydroxyl groups to prevent truncated/mixed-sequence synthesis; oxidation of the resulting phosphite to a phosphotriester; and cleavage of the 5′-trityl protecting group in readiness for chain extension (or global deprotection and cleavage from the solid support)^18,19^. This synthesis relies on phosphoramidite building blocks, which are intrinsically sensitive and require storage at low temperatures under an inert atmosphere to avoid degradation via oxidation and/or hydrolysis^20,21^. Even when handled under these conditions, their routine use in solution form at ambient temperature inevitably leads to degradation over time via both autocatalytic and hydrolytic pathways. In addition, phosphoramidite monomers that are used to assist cell uptake and targeting^22^ are expensive and limited in range and availability, and the excess reagent needed to drive the coupling reaction to completion is not easily recovered^23–25^. Postsynthetic modifications such as activated ester-based amide bond formation, CuAAC or other click conjugations offer a potential solution to these challenges but can require additional steps such as protecting-group manipulation or functional-group activation (and associated purifications)^26^.

We reasoned that these limitations could be addressed by the development of a strategy that uses readily available, inexpensive and air/moisture-stable alcohols (including nucleosides) to directly functionalize the growing oligonucleotide chain. Such a strategy would enable site-specific terminal and internal oligonucleotide modification, and would facilitate recovery and recycling of unreacted alcohol/nucleoside monomers. Key to this approach is the direct phosphitylation of the 5′-hydoxyl group of the growing immobilized oligonucleotide chain, followed by in situ coupling of the activated 5′-P(III) terminus with the hydroxyl group of a nucleophilic modifier or, for oligonucleotide chain extension, the 3′-hydroxyl group of a solution-phase 5′-DMT-protected (DMT, dimethoxytrityl) nucleoside (Fig. 1b). This strategy offers several advantages over the state of the art: first, 5′-DMT-protected nucleosides and other alcohol nucleophiles are stable, readily available, cost-effective alternatives to conventional sensitive 3′-phosphoramidites; second, unwanted on-resin activation of the newly introduced DMT-protected nucleoside, which can otherwise lead to the formation of (*n* + 1) impurities^7^, is intrinsically avoided; and third, any unreacted 5′-phosphoramidite is converted to the corresponding (unreactive) 5′-phosphate in the ensuing oxidation step, which eliminates the need for a separate ‘capping’ step of any truncated oligonucleotides. This strategy is in principle agnostic of directionality, so would be compatible with the synthesis and modification of an oligonucleotide chain in either the standard (3′ → 5′, Fig. 1b) or reverse (5′ → 3′, Fig. 1c) directions, thus offering opportunities to access oligonucleotides modified with a wide range of functionalities at the 5′ or 3′ termini. This is important because the current ‘phosphoramidite monomer’ approach is poorly tolerant of certain functional groups, which restricts the diversity and tunability of oligonucleotide conjugates and, consequently, the physicochemical properties and biological activity of modified oligonucleotides. The introduction of an azide functionality is highly desirable for alkyne-based bioconjugation; however, it remains challenging to achieve via traditional solid-phase oligonucleotide synthesis due to the incompatibility of azides with the P(III) phosphoramidite reagents typically used during coupling. Likewise, the installation of free amine groups generally requires amine protection during synthesis to prevent side reactions during the coupling step. This reduces synthetic efficiency and results in burdensome purification requirements. Our strategy circumvents these limitations because nucleophilic attack on the phosphoramidite by the primary alcohol outcompetes potential side reactions involving azide or free amine functionalities. This chemoselectivity eliminates the need for protecting groups and enables improved functional-group tolerance.

## Results

**Fig. 2:**
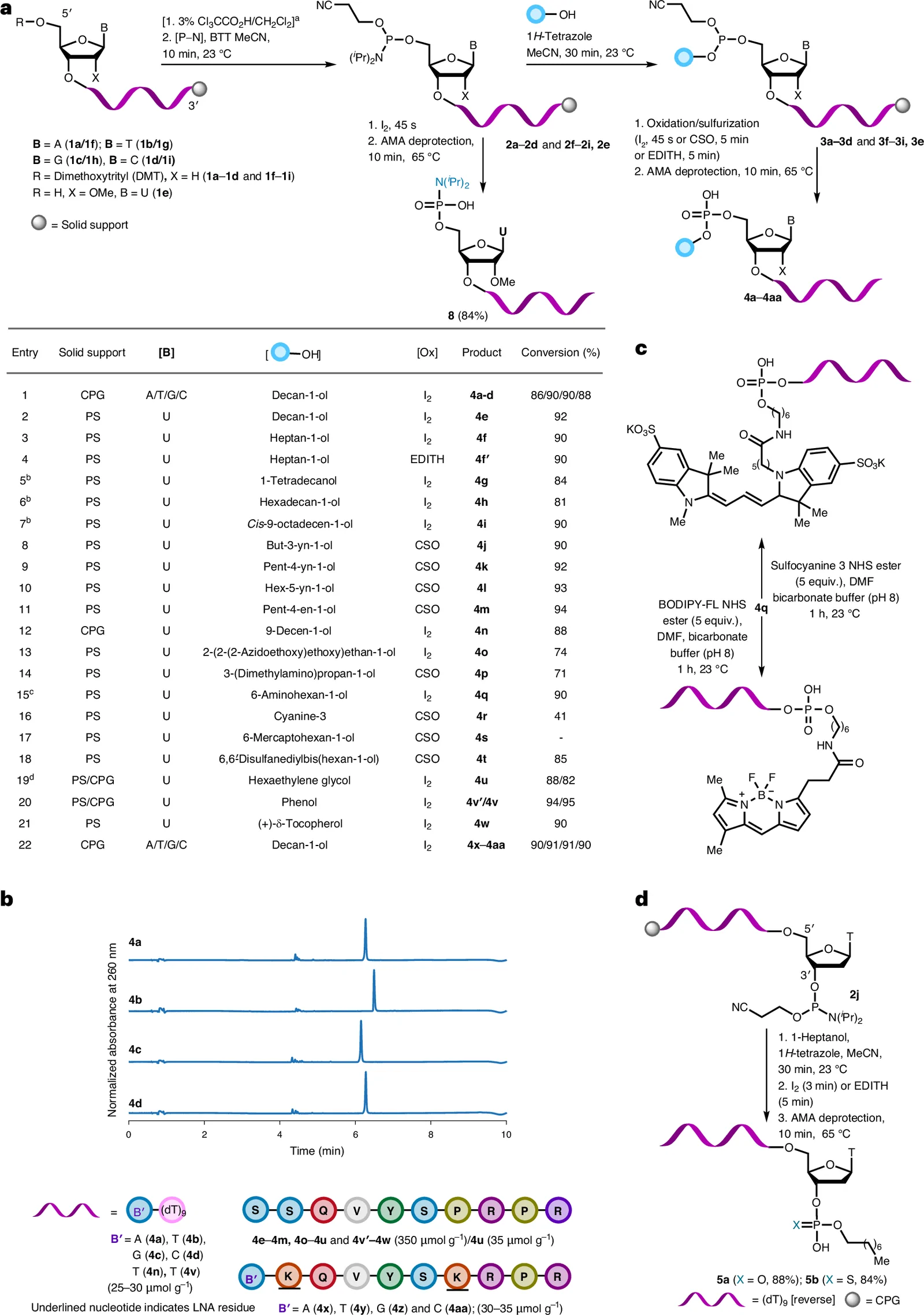
Versatile on-support functionalization of oligonucleotides at the 5′ and 3′ positions. **a**, Functionalization at the 5′ position of CPG/mixed-sequence PS-supported oligonucleotides. Both I_2_ and CSO oxidation are compatible with our strategy. ^a^Detritylation performed for oligonucleotides **1a**–**1d** and **1f**–**1i**. ^b^Alcohols were dissolved in MeCN/toluene (1:1). ^c^6-Aminohexan-1-ol was dissolved in MeCN/DMF (8:2). ^d^Conversion to a mixture of monomeric and dimeric oligonucleotides. **b**, UHPLC chromatograms of the reaction mixtures for formation of **4a**–**4d**. **c**, Fluorescent labelling of **4q**. **d**, Functionalization at the 3′ position of an CPG-supported reverse amidite (dT)_10_-mer. B, nucleobase (protected during solid-phase synthesis; deprotected after cleavage from resin). For the coupling reactions, the alcohol (0.25 M) and 1*H*-tetrazole (0.45 M) solutions were premixed in a 1:1 (v/v) ratio prior to delivery to the synthesis columns. BODIPY-FL NHS ester, 4,4-difluoro-5,7-dimethyl-4-bora-3a,4a-diaza-s-indacene-3-propionic acid, succinimidyl ester. Ammonia–methylamine (AMA) deprotection was performed with 35% NH_3_ (aq.) and 40% CH_3_NH_2_ (aq.), 65 °C, 10 min. S, uridine-2′-ome; Q, cytidine-2′-ome; V, adenosine-2′-F; Y, uridine-2′-F; P, adenosine-2′-ome; R, guanosine-2′-ome; K, thymidine-LNA. Source data

Our realization of these synthetic strategies began with a series of model 3′-CPG-supported (CPG, controlled pore glass) **B**(dT)_9_ oligonucleotides (Fig. 2a, B = A (**1a**), T (**1b**), G (**1c**) and C (**1d**), 25–30 µmol g^−1^ loading; see also Supplementary Information, section 2.1–2.7 and 3.1–3.2). Deprotection of the 5′-terminal DMT ethers of oligonucleotides **1a**–**1d** was followed by phosphitylation of the resulting free 5′-hydroxyl using 3-((bis(diisopropylamino)phosphino)oxy)propanenitrile ([P–N]) with 5-benzylthio-1*H*-tetrazole (BTT) as an activator, which gave the corresponding phosphoramidites **2a**–**2d**. In situ coupling of these intermediates with decan-1-ol afforded intermediate phosphite-triesters **3a**–**3d**, which were then oxidized with iodine and subjected to global deprotection to yield the free oligonucleotides **4a**–**4d** in 86–90% conversion (Fig. 2a, table entry 1; see Supplementary Information, section 3.3 for detailed reaction protocols, section 4.1 for spectral analyses, and sections 13 and 14 for the supplementary cycle method). We next applied the optimized reaction conditions to a high-loaded, mixed-sequence oligonucleotide **1e** bearing 2′-F and 2′-*O*-Me substituted nucleotides (which are key motifs in pharmaceutically relevant oligonucleotides^1^) immobilized on polystyrene (PS) resin (350 µmol g^−1^; Supplementary Information, section 3.2). Subjection of **1e** to phosphitylation was followed by coupling with a range of alcohols to study the scope of this step; oxidation and sulfurization was achieved with iodine, (1*S*)-( + )-(10-camphorsulfonyl)-oxaziridine (CSO) or 3-ethoxy-1,2,4-dithiazolidin-5-one (EDITH). Under our optimized reaction conditions, coupling of phosphoramidite **2e** with decan-1-ol or heptan-1-ol followed by oxidation/sulfurization and global deprotection afforded the free modified oligonucleotides **4e**, **4f** and **4f′** in 90–92% conversion (Fig. 2a, entries 2–4; Supplementary Information, section 4.2), highlighting the compatibility of the method with more complex oligonucleotide sequences. Incorporation of lipidic alcohols, including 1-tetradecanol, hexadecan-1-ol and *cis*-9-octadecen-1-ol was successfully achieved, with conversions of 81–90% (**4g**, **4h** and **4i**, entries 5–7), demonstrating that the methodology is compatible with hydrophobic motifs. The facilitated introduction of a lipophilic ‘tail’ holds substantial value for the development oligonucleotide therapeutics because it has been shown to enhance membrane affinity, facilitate cellular uptake and improve the biological stability of oligonucleotides^27,28^.

We next addressed the incorporation of nucleophiles featuring additional functional groups, which would greatly enhance the utility of the protocol; for example, the introduction of alkyne, alkene and azide sidechains would facilitate highly selective click ligations^26,29–32^, while amines and thiols provide orthogonal routes for conjugation to fluorophores, peptides and other molecules^33,34^. Coupling of alkynyl and alkenyl alcohols proceeded in near-quantitative conversions in all cases (**4j**–**4n**, 88–94%, entries 8–12), while the reaction with 2-(2-(2-azidoethoxy)ethoxy)ethan-1-ol afforded the modified azido oligonucleotide **4o** with 74% conversion; in the latter case, no Staudinger-like reduction of the azide by the P(III) functionality was observed (entry 13). This considerably expands opportunities for downstream click-functionalization of oligonucleotides by thiol–ene or azide–alkyne cycloaddition reactions. Due to the high oxophilicity of the phosphoramidite intermediate, we were also able to achieve a number of chemoselective oligonucleotide functionalizations using bifunctional nucleophiles. For example, 3-(dimethylamino)propan-1-ol and 6-aminohexan-1-ol underwent selective reaction at the hydroxyl group with 71–90% conversion (**4p** and **4q**, entries 14 and 15), facilitating access to 5′-amino tagged oligonucleotides that offer a variety of synthetic opportunities and biological applications^33^. For example, the deprotected oligonucleotide was successfully labelled with BODIPY-FL and sulfo-cyanine 3 *N*-hydroxysuccinimide esters in near-quantitative conversion, providing a facile means for fluorescent labelling of the oligonucleotide (Fig. 2c and Supplementary Information, section 4.3). We also found that a fluorescent oligonucleotide could be directly prepared through coupling of the activated oligonucleotide with a cyanine dye (Cy 3) featuring a primary-alcohol-containing sidechain (**4r**, entry 16; Supplementary Information, section 4.4). 6-Mercaptohexan-1-ol similarly underwent chemoselective reaction at its hydroxyl group with the P(III) centre, although competing formation of the disulfide oligonucleotide dimer, and the thia-Michael adduct of acrylonitrile (generated during phosphodiester deprotection), was also observed (**4s**, entry 17; Supplementary Information, section 4.2). To suppress the latter pathways, we used 6,6′-disulfanediylbis(hexan-1-ol), which afforded only two products: the desired monosubstituted oligonucleotide, and a disulfide-linked bis-oligonucleotide (entry 18). Treatment of this mixture with tris(2-carboxyethyl)phosphine hydrochloride (TCEP) afforded the free thiol **4t** with an overall conversion of 85%, thus offering a useful entry to thiol-substituted oligonucleotides. Indeed, it is common practice to store 5′-thiol-modified oligonucleotides as disulfides, and to liberate the free thiol just prior to use. Furthermore, using hexaethylene glycol afforded mono- and difunctionalized products in a 3:2 ratio (**4u**, entry 19), with the diol acting as an oligonucleotide ‘staple’ in the latter case. Oligonucleotide dimers including higher-order siRNA constructs are of considerable interest for applications in oligonucleotide therapeutics^35^. Comparative studies using the same sequence on a high-loading PS support (350 µmol g⁻^1^) and a lower-loading CPG support (35 µmol g⁻^1^) under identical conditions revealed that the overall distribution of monomeric and dimeric products was largely maintained, with the lower-loading CPG showing a slight improvement in the ratio of desired monomer to dimer (entry 19; Supplementary Information, section 4.2). Further experiments using the lower-loading CPG sequences (35 µmol g⁻^1^) with varying concentrations of hexaethylene glycol (0.25–3.0 M) demonstrated that increasing the alcohol concentration progressively suppresses dimer formation, but slightly reduces overall coupling efficiency. Based on these data, an alcohol concentration of 0.25 M was identified as providing the optimal balance between efficient coupling and minimization of intermolecular difunctionalization.

We next sought to evaluate the reactivity of aromatic alcohols. Pleasingly, phenols also coupled efficiently with the in situ-formed phosphoramidite **2b**/**2e**, delivering phenol-functionalized oligonucleotides **4v**/**4v′** in near-quantitative conversion (95% and 94% for CPG- and PS- supported mixed sequences, respectively; entry 20). Furthermore, the lipophilic δ-tocopherol moiety, an important motif used to enhance membrane association and delivery of therapeutic oligonucleotides^36^, was successfully incorporated, affording the δ-tocopherol-modified oligonucleotide **4w** in 90% conversion (entry 21) and further demonstrating the robustness of the methodology. To further evaluate the generality of the method, we investigated terminal nucleobase variation using other structurally diverse sequences. A CPG-supported sequence (30–35 µmol g^−^^1^), containing a locked nucleic acid (LNA) 2′-*O*-Me, and 2′-F modifications, was prepared with different terminal bases (A (**1f**), T (**1g**), G (**1h**) and C (**1i**)) and subjected to the optimized reaction conditions using decan-1-ol as nucleophile. Once again, the desired oligonucleotides (**4x**–**4aa**, entry 22) were obtained in near-quantitative conversion (90–91%), demonstrating the broad applicability of this strategy across diverse nucleobase contexts and complex sequences.

We then turned our focus to application of the functionalization strategy to the ‘reverse’ direction (5′ → 3′) oligonucleotide synthesis. A CPG-supported 5′ → 3′ (dT)_10_ oligonucleotide bearing a 3′-hydroxyl group was thus subjected to phosphitylation under the optimized reaction conditions (**2j**), followed by coupling with the 1-heptanol. Following oxidation or sulfurization and global deprotection, the desired 3′-functionalized oligonucleotides **5a** and **5a′** were obtained in 88% and 84% conversion respectively (Fig. 2d and Supplementary Information, section 4.5). We were further able to apply the oligonucleotide phosphitylation strategy to the synthesis of a 5′-phosphoramidate—an analogue of 5′-phosphate that can confer favourable physicochemical and biological properties on oligonucleotides for therapeutic applications by enhancing metabolic stability, which improves tissue accumulation and extends the duration of gene silencing activity in vivo^37^. This was achieved by direct oxidation and global deprotection of the phosphitylated intermediate **2e**, which afforded phosphoramidate ester **8** (Fig. 2a and Supplementary Information, section 4.6) with 84% conversion. This approach would in principal offer flexibility in the choice of amine group by tuning of the phosphitylating agent.

**Fig. 3:**
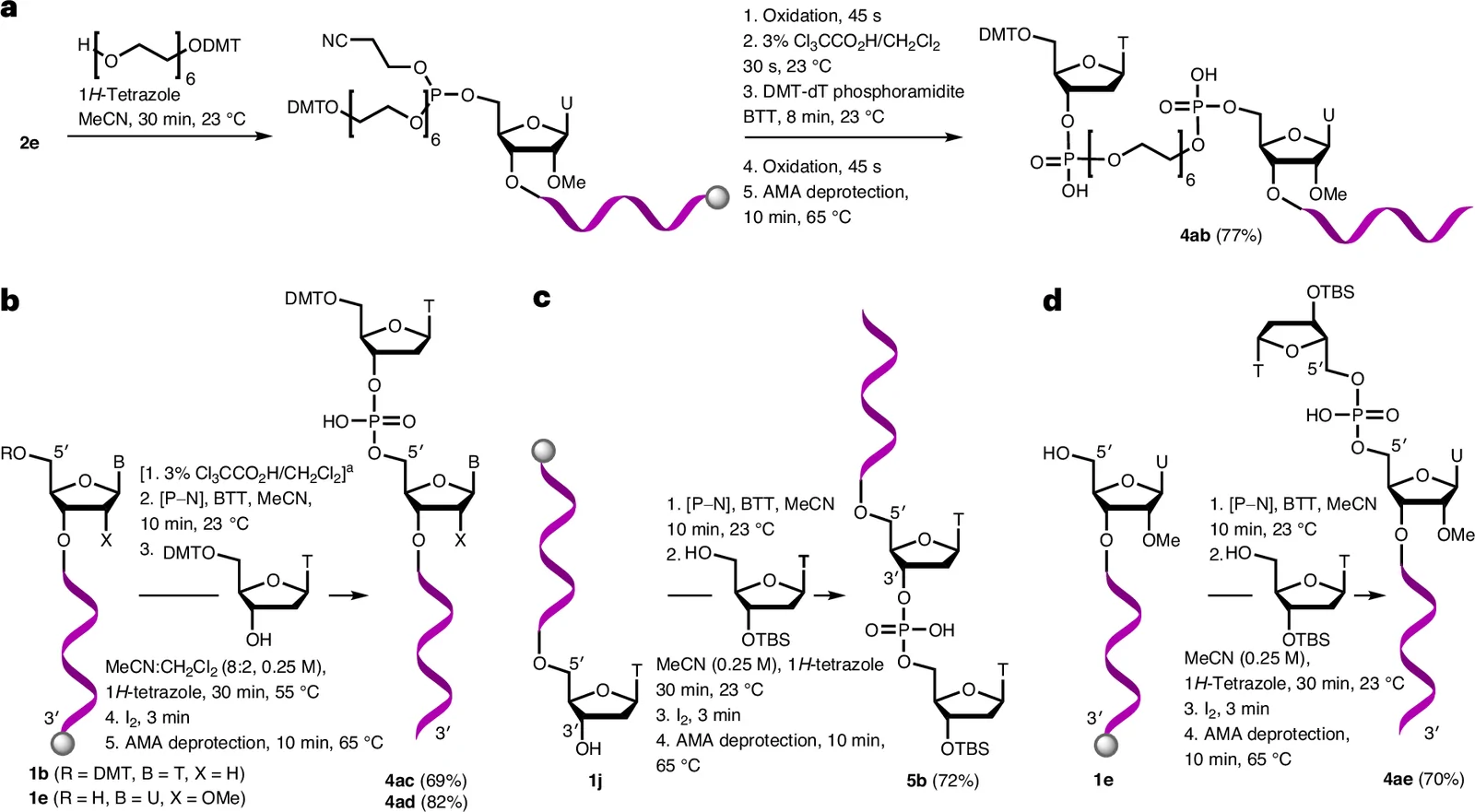
Internal and terminal bidirectional chain extension strategies. **a**, Internal modification of PS-supported oligonucleotides using DMTO-PEG_6_-OH. **b**, 3′ → 5′ chain extension using oligonucleotides **1b** and **1e**. ^a^Detritylation performed for oligonucleotide **1b**. **c**, 5′ → 3′ chain extension using **1j**. **d**, 5′ → 5′ chain extension using oligonucleotide **1e**. TBS, *tert*-butyldimethylsilyl.

After establishing the protocol for terminal modifications, we sought to evaluate whether the methodology could be extended to site-selective internal modifications. We therefore investigated the phosphitylation of the 5′-hydroxyl group of **1e**, followed by coupling with DMTO-PEG_6_-OH (DMT, dimethoxytrityl; PEG, polyethylene glycol) (Fig. 3a and Supplementary Information, section 5). Subsequent detritylation using 3% trichloroacetic acid was followed by DMT-dT phosphoramidite coupling, affording **4ab** in 77% conversion (over two steps). This result demonstrates that the strategy is effective for controlled internal functionalization.

With a range of oligonucleotide functionalizations established using primary alcohol nucleophiles, we next explored the use of nucleosides as coupling partners, which would effect oligonucleotide chain extension without the need for sensitive phosphoramidite monomers. To test this, we coupled oligonucleotides **1b** and **1e** with a 5′-DMT-dT nucleoside (Fig. 3b). Following oxidation and global deprotection, the free [*n* + 1] oligonucleotides **4ac** and **4ad** were isolated with 69% and 82% conversion, respectively (Supplementary Information, section 6). Next, 5′ → 3′ (dT)_10_ oligonucleotide **1j** featuring a 3′-hydroxyl group was subjected to phosphitylation under the optimized reaction conditions, followed by coupling with the 5′-hydroxyl of 3′-OTBS-dT (Fig. 3c); oxidation and global deprotection afforded the [*n* + 1] oligonucleotide **5b** with 72% conversion. Finally, we constructed a 5′ → 5′ phosphodiester linkage through reaction of **1e** with 3′-OTBS-dT, which afforded **4ae** in 70% conversion after oxidation and global deprotection (Fig. 3d). The latter is a useful finding because inverted nucleotides are commonly used at the termini of therapeutic siRNA constructs to improve resistance to nuclease degradation^38^.

Throughout these studies, we noted that a key advantage of this strategy is the efficient recovery of unreacted alcohol nucleophiles or 5′-DMT-protected nucleoside (dT) by diluting the reaction mixture (containing the coupling base and activator, 1*H*-tetrazole) with an organic solvent such as dichloromethane, followed by washing with aqueous Na_2_CO_3_. This straightforward cost-effective procedure afforded DMT-dT with 92% recovery (Supplementary Information, section 7); in contrast, the recycling of nucleoside phosphoramidites in traditional oligonucleotide synthesis requires additional steps to convert the phosphotetrazolide intermediate back to a phosphoramidite via reaction with diisopropylamine^25^. In this approach, a single inexpensive phosphitylating agent efficiently converts either the 5′- or 3′-hydroxyl groups of solid-supported oligonucleotides into the corresponding phosphitylated intermediates, which subsequently undergo coupling with diverse alcohols. Under the optimized conditions, a broad range of oligonucleotide functionalizations was achieved using primary aliphatic alcohol and phenol nucleophiles, with high functional-group tolerance and chemoselectivity over other nucleophiles, and with coupling efficiencies of up to 95%. In contrast, secondary alcohols and nucleophiles such as alkyl amines and thiols showed poor conversion, with predominantly unreacted phosphoramidite intermediates observed.

**Fig. 4:**
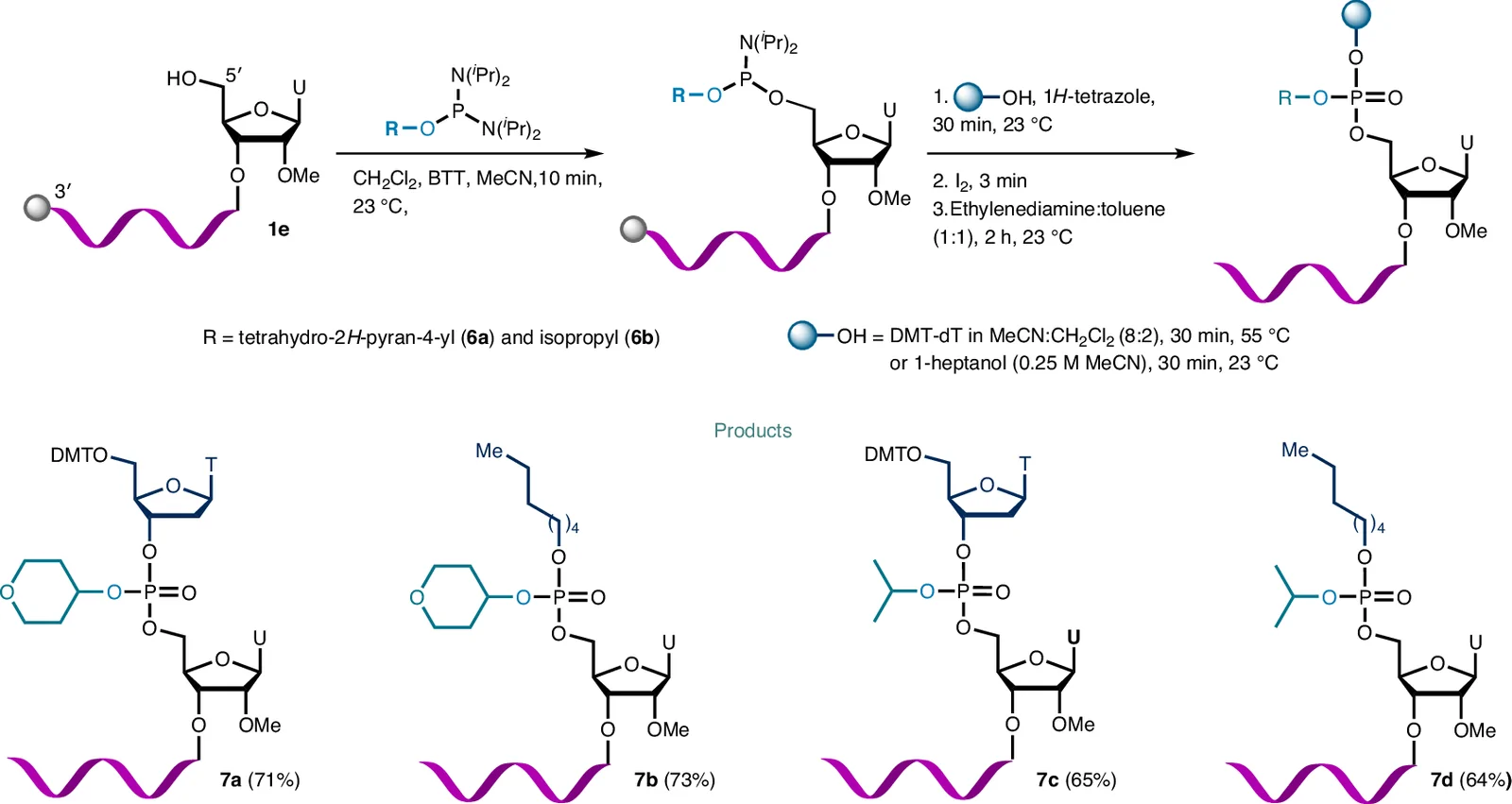
Synthesis of oligonucleotides containing a phosphotriester backbone. OMe, methoxy.

We next questioned whether this approach could be applied not only to the synthesis of phosphodiester linkages, but also to phosphotriester backbones, which are desirable as nuclease-stable motifs that have been used as biomarkers^39^ and in biodegradable prodrugs^40,41^. To this end, we prepared alkylphosphordiamidites **6a** and **6b**^42^ (Fig. 4 and Supplementary Information, section 8) and applied them to phosphitylation of the 5′-hydroxyl of **1e**, followed by coupling with DMT-dT or heptan-1-ol as exemplar nucleophiles. Oxidation and global deprotection afforded the corresponding phosphotriester backbone-containing oligonucleotides **7a**–**7d** in 64–71% conversion, demonstrating the successful synthesis of uncharged phosphotriester backbones by activation of the oligonucleotide chain on the solid phase, and with much potential to modify the triester component.

**Fig. 5:**
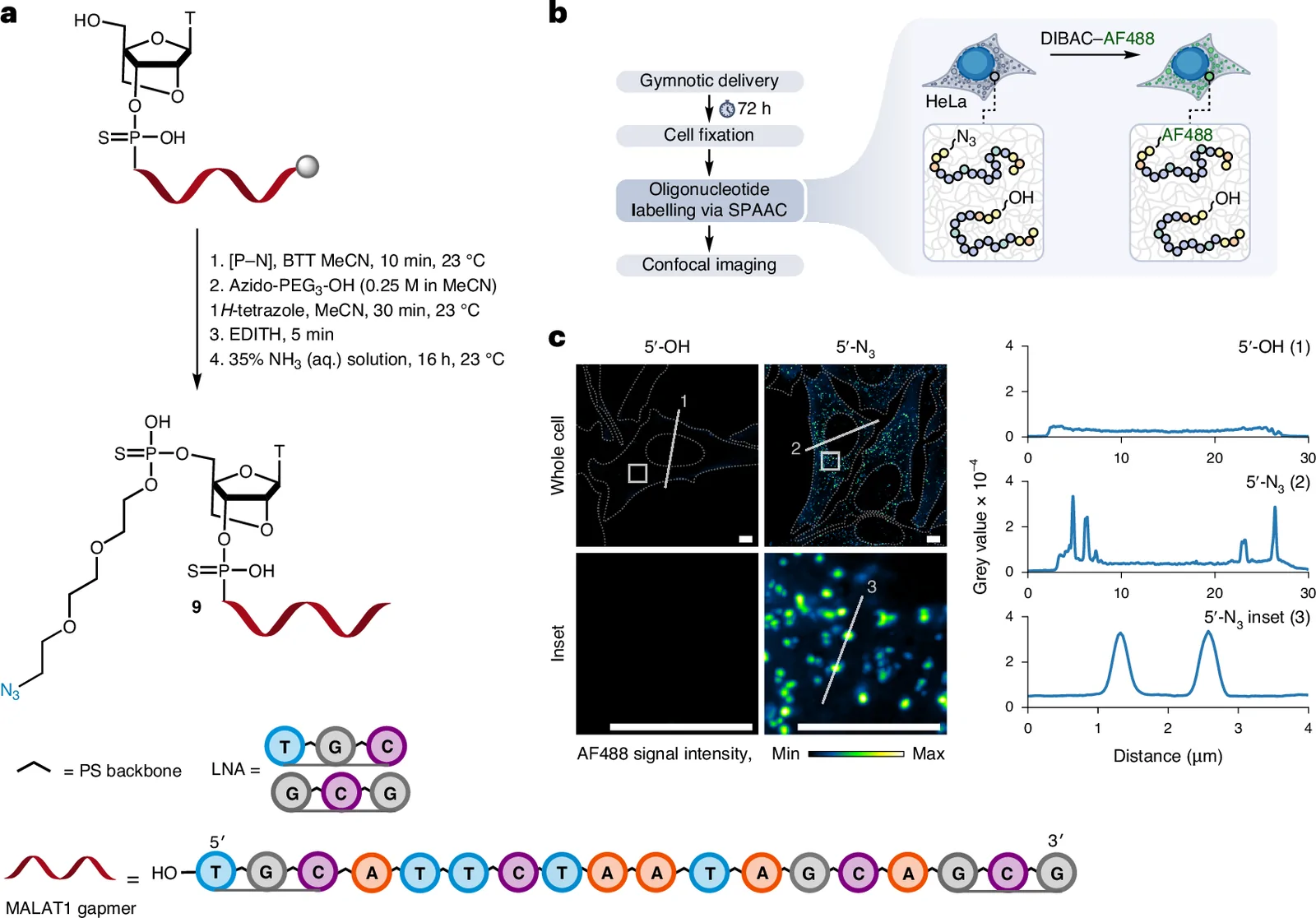
Application of the 5′-functionalization methodology for oligonucleotide detection in cultured HeLa cells. **a**, Sequence and the synthesis of the MALAT1-targeting LNA/PS gapmer (antisense oligonucleotide) used in the study. **b**, Design of the click-chemistry-based postfixation labelling with AF488 fluorophore. **c**, Mapping subcellular localization of the postfixation-labelled oligonucleotide in HeLa cells via confocal imaging. Whole cell and nucleus segmentation based on CellTracker Deep Red and DAPI staining. Maximum intensity projection. Scale bars: 5 μm. Plots in the right part of the panel show intensity profiles of image fragments 1–3. DAPI, 4′,6-diamidino-2-phenylindole; DIBAC, dibenzoazacyclooctyne; Max, maximum; Min, minimum. Source data

The ability to selectively introduce a range of functionalities at the 5′ terminus opens up many opportunities for the modification of therapeutically relevant oligonucleotides. For example, the terminal 5′-azide modification is of high utility for oligonucleotide conjugation but is challenging to prepare when combined with an LNA phosphorothioate backbone. This is due to the competing Staudinger reaction of the azide with phosphoramidite reagents, and of intramolecular cyclization of the phosphorothioate sulfur atom with azide precursors such as 5′-halides^43,44^ (Supplementary Information, section 9.1). Using our optimized reaction conditions, we were able to efficiently synthesize **9** (Fig. 5a), an azide-containing MALAT1-targeting antisense oligonucleotide with an LNA phosphorothioate backbone. To confirm the successful installation of the azide and its reactivity, we carried out strain-promoted azide–alkyne cycloaddition (SPAAC) between the azido-tagged oligonucleotide and DIBAC-AF488 fluorophore in fixed HeLa cells and performed confocal imaging (Fig. 5b and Supplementary Information, sections 10–12). Cells treated with the 5′-azido-modified oligonucleotide showed a strong punctate fluorescence signal, whereas an unmodified 5′-hydroxyl control produced a uniform, low-intensity background (Fig. 5c). Moreover, the AF488 fluorescence signal of the 5′-azido-modified version was well separated from the background. These results show that our terminal modification method can be successfully utilized to install 5′-azide tags in oligonucleotides and that the installed tags remain reactive in the modified oligonucleotides, offering a robust and specific postfixation fluorescent labelling strategy.

While high reagent equivalents were used to ensure complete coupling on small-scale CPG- and PS-supported sequences (for automated synthesis: CPG (1 µmol), 120 equiv. of phosphitylating agent, 150 equiv. of alcohol; PS (5.25 µmol), 22.9 equiv. of phosphitylating agent, 28.6 equiv. of alcohol), lower equivalents (11.4 equiv. of phosphitylating agent, 21.4 equiv. of alcohol for 5.25-µmol synthesis on PS support) were also found to lead to efficient reaction without notable reduction in conversion (Supplementary Information, section 3.3). Importantly, efficient recovery strategies allow recycling of more valuable alcohols and nucleosides. It is well established that, for larger-scale syntheses on optimized automated platforms at pilot scale, high conversion can be achieved using reduced equivalents of coupling reagent.

## Discussion

Many existing oligonucleotide modification strategies provide ≥98% stepwise coupling efficiency with growing oligonucleotide chains but are critically dependent on the previous preparation of chemically modified phosphoramidites. Due to their short shelf-life arising from poor stability towards hydrolysis and oxidation, only a limited number of expensive and unstable phosphoramidites are available and necessitate protecting-group chemistry when non-tolerated functional groups are present. This limits both the scope and flexibility of oligonucleotide functionalization. In this study, we developed a straightforward, cost-effective method for oligonucleotide modification via on-support oligonucleotide phosphitylation and in situ nucleophilic functionalization using ubiquitous P(III) phosphitylating reagents, and an array of readily available alcohol nucleophiles. The advantages of this strategy are emphasized by our ability to directly use bifunctional nucleophiles, such as 6-aminohexyl and 6-thiohexyl, and azido-alcohols, which would not be tolerated under the conventional phosphoramidite approach. As a result, our methodology simplifies the workflow of oligonucleotide functionalization and purification, while maintaining coupling efficiencies of up to 95%. Furthermore, the unreacted nucleosides and alcohols used in the coupling step can be recovered in over 90% yield with high purity through a simple aqueous work-up and extraction protocol, markedly improving recyclability. This methodology therefore provides convenient access to a wide range of terminal and internal oligonucleotide modifications, offering flexibility that cannot be matched by the current provision of modified phosphoramidites.

## Methods

Oligonucleotide synthesis via on-support phosphitylation was performed using automated and manual solid-phase synthesis workflows on CPG and PS supports. Automated syntheses were carried out on an Applied Biosystems 394 DNA/RNA synthesizer on 1-μmol (CPG) and 5.25-μmol (PS) scales. On-column phosphitylation reactions used 2-cyanoethyl *N,N,N*′*,N*′-tetraisopropylphosphorodiamidite with BTT activation under anhydrous conditions. Coupling of nucleophiles to on-support phosphitylated intermediates was carried out using 1*H*-tetrazole as activator, followed by oxidation or sulfurization as required. The resulting functionalized oligonucleotides were subjected to global deprotection using AMA. Crude oligonucleotides were purified by high-performance liquid chromatography where indicated and characterized by liquid chromatography–mass spectrometry. Detailed experimental procedures, synthesis protocols, reagent information, purification conditions, analytical methods, supplementary cycle methods and scripts are provided in the Supplementary Information.

### Reporting summary

Further information on research design is available in the Nature Portfolio Reporting Summary linked to this article.

## Online content

Any methods, additional references, Nature Portfolio reporting summaries, source data, extended data, supplementary information, acknowledgements, peer review information; details of author contributions and competing interests; and statements of data and code availability are available at https://doi.org/10.1038/s41557-026-02214-6.

## Supplementary information

Supplementary Information Supplementary Methods (sections 1–15), spectral data and figures, scripts and references (section 16).

Reporting Summary

Peer Review File

## Source data

Source Data Fig. 2 Statistical source data for Fig. 2b.

Source Data Fig. 5 Statistical source data for Fig. 5c.

## Data Availability

The data generated in this study are available in the Supplementary Information. Correspondence and requests for materials should be addressed to T.B. Source data are provided with this paper.

## Code availability

Custom script for encoding/decoding ABI 394 oligonucleotide synthesis cycle files is available via GitHub at https://github.com/SGolojuch/OligoNET-cycle-encoder-decoder.
