# Supplementary material for: A versatile strategy for oligonucleotide functionalization via on-support phosphitylation
Source: Nat Chem. 2026 Jul 22;18(10):1747–54. doi: 10.1038/s41557-026-02214-6 (PMC13623645; doi:10.1038/s41557-026-02214-6)
Supplement: Supplementary file 1 [file 41557_2026_2214_MOESM1_ESM.pdf]

nature chemistry

Supplementary information https://doi.org/10.1038/s41557-026-02214-6

A versatile strategy for oligonucleotide functionalization via on-support phosphitylation

In the format provided by the authors and unedited

Table of Contents

1. General statements 2
2. Experimental procedures 3
2.1 General reaction protocol-1 3
2.2 Automated solid-phase synthesis 3
2.3 Syringe-based manual synthesis 4
2.4 Purification of antisense oligonucleotides 5
2.5 Nucleic acids desalting: 5
2.6 Determination of concentrations of nucleic acids 5
2.7 Mass- spectrometric analysis 5
3.1 Oligonucleotide sequence composition 6
3.2 Spectral analysis of the $(dT)_{10}$ sequence (**1b**) and mixed sequence (**1e**) 7
3.3 Optimizations 9
4. 5'-Functionalization of solid-supported oligonucleotides 13
4.1 Functionalization at the 5'-position of a CPG supported $B(dT)_9$ sequence using alcohols15
4.2 Functionalization at the 5'-position of a polystyrene supported mixed sequence 19
4.3 Labelling of the NH_2 group 49
4.4 Characterization of cyanine 3 dye 52
4.5 'Reverse' (5'→3') modified oligonucleotide synthesis of the $(dT)_{10}$ sequence 54
4.6 Oligonucleotide phosphoramidate (**8**) synthesis 56
5. Internal modifications 58
6. Nucleoside Coupling 59
6.1 Nucleoside (3'-OH) coupling with 5'-OH of $(dT)_{10}$ sequence on CPG solid support........ 59
6.2 Nucleoside (3'-OH) coupling with 5'-OH of SSQVYSPRPR sequence on PS solid support60
6.3 Nucleoside (5'-OH) coupling with 3'-OH of reverse $(dT)_{10}$ sequence on CPG solid support 61
6.4 Nucleoside (5'-OH) coupling with 5'-OH of mixed SSQVYSPRPR sequence on PS solid support 62
7. Recovery of the DMT-dT nucleoside 63
8. Phosphotriester backbone synthesis 65
8.1 Spectral characterization of the phosphonamidites 6a and 6b 65
8.2 Reaction conditions for charge neutral phosphotriester backbone synthesis 68
8.3 UHPLC Analysis of crude phosphotriester-modified oligonucleotides (**7a-d**) 69
9. 5' Modification of the MALAT1 LNA/PS gapmer: installation of the azide group 73
9.1 Attempted installation of the 5'-azide functionalities of a LNA sequences 74
9.2 Synthesis and chromatogram of the azido-modified MALAT1 gapmer (**9**) 76
10. Statistics & reproducibility 77
11. Cell culture 77
12. Microscopy 77
13. Supplementary scripts 79
14. Supplementary cycle method 86
15. Molar extinction calculation for the 4,4'-dimethoxytrityl (DMT) group 92
16. References 94

1. General statements

NMR Spectroscopy: Proton (^1H), and carbon (^{13}C) NMR spectra were recorded on Bruker AVIII HD 400, NEO 600, AVIII HD 500, or AVII 500 spectrometers. ^1H, ^{31}P and ^{13}C, chemical shifts (δ) are quoted in parts per million (ppm). ^1H NMR spectra were recorded using an internal deuterium lock for the residual protons in DMSO-d_6 (δ = 2.50) and acetonitrile-d_3 (δ = 1.94). ^{13}C NMR spectra were recorded using an internal deuterium lock in DMSO-d_6 (δ = 39.5) or acetonitrile-d_3 (δ = 118.2). Assignments were determined either on the basis of unambiguous chemical shift or coupling patterns. Peak multiplicities are defined as s (singlet), d (doublet), t (triplet), q (quartet), p (pentet), sext (sextet), sept (septet), and m (multiplet). Coupling constants (J) are reported to the nearest 0.1 Hz.

Chromatography: Column chromatography refers to normal phase column chromatography and was performed on silica gel obtained from Merck (Silica gel Si 60, 0.040–0.063 mm) under a positive pressure of nitrogen, using the stated solvent system. Analytical thin-layer chromatography was performed on pre-coated aluminium-backed plates (Merck Kieselgel 60 F_{254} plates) with visualization by ultraviolet light (254 nm) and/or by staining with potassium permanganate. Retention factors (R_f) are reported with the solvent system in parentheses.

Materials/procedures: All reactions were carried out in anhydrous solvents using Empty Synthesis Columns-TWIST 40nm, 0.2um or 1um and oven-dried glass vials (used for reagent preparation) under an inert atmosphere of argon. Dry tetrahydrofuran, CH_2Cl_2, toluene, pyridine, triethylamine, MeCN, DMF and diethyl ether were purchased from Merck and also collected from an mBraun SPS-800 solvent purification system, having been passed through anhydrous alumina columns. All other commercially available reagents and solvents, where appropriate, were dried and purified before use, using standard procedures.

2. Experimental procedures

2.1 General reaction protocol-1: For all sequences, 15 mg of the respective resin was loaded into an empty synthesis column (synthesis columns-TWIST 40nm, 0.2um or 1um - 20-0030-00), flushed with argon, and washed with 1 mL of dry MeCN prior to initiating synthesis. A 0.3 M solution of the 2-cyanoethyl *N*,*N*,*N*',*N*'-tetraisopropylphosphorodiamidite in MeCN and **6a** and **6b** in CH_2Cl_2 were prepared prior to the reaction. Commercially available 5-benzylthio-1*H*-tetrazole (BTT) solution (0.3 M in MeCN) was used as the activator for the phosphitylation step.

2.2 Automated solid-phase synthesis: Automated solid-phase synthesis of oligonucleotides was performed on an Applied Biosystems 394 automated DNA/RNA synthesizer on a 1 µmol scale (CPG) 5.25 µmol scale (PS) under the control of OligoNet 1.0.1 (Applied Biosystems) software. For these syntheses, LNA-G (dmf) CNA 1000 Å CPG 42 µmol g^{-1} solid support (#BG7-0813-B, LGC Biosearch Technologies), 2'-OMe-G (dmf) CPG Column, 1000 Å, 1 µmol (LK2311-P008, LGC Biosearch Technologies), dT CPG Column, 1000 Å, 1 µmol (#LK2271-P011, LGC Biosearch Technologies) and DMT-dG(ib) (#G111030-12X2G, Sigma-Aldrich), DMT-dA(bz) (#A111030-12X2G, Sigma-Aldrich), DMT-dT (#T111030-12X2G, Sigma-Aldrich), 5-Me-dC-CE (#10-1060-02, Glen Research), DMT-locT (#TL11430-1G, Sigma-Aldrich), DMT-locMeC(bz) (#CL11430-1G, Sigma-Aldrich), DMT-locG(ib) (#GL11430-1G, Sigma-Aldrich), TheraPure™ 2'-OMe-C (#27-2043-04, Thermo Fisher Scientific), TheraPure™ 2'-OMe-U (#27-2044-04, Thermo Fisher Scientific), TheraPure™ 2'-OMe-G (#27-2046-04, Thermo Fisher Scientific), TheraPure™ 2'-OMe-Bz-A (#27-2042-04, Thermo Fisher Scientific), 2'-Fluoro-Bz-A (#27-1601-02, Thermo Fisher Scientific), TheraPure™ 2'-fluoro-U (#27-1602-02, Thermo Fisher Scientific) phosphoramidites were used. All phosphoramidites were dissolved in anhydrous MeCN (#L010000-6X100ML, Sigma-Aldrich) to a concentration of 0.1 M immediately prior to use. For detritylation, coupling, capping, sulfurization, and washes during the synthesis cycle, the following reagents were used (respectively): TCA Deblock (#L020000-4X4L, Sigma-Aldrich), BTT Activator (#L033000-1L, Sigma-Aldrich), Cap A (#L040030-6X450ML, Sigma-Aldrich)/Cap B (#L050030-6X450ML, Sigma-Aldrich), EDITH (#LK2171-G023, LGC Biosearch Technologies), and MeCN (#L010000-4X4L,

Sigma-Aldrich). The coupling time was 60 s for standard DNA phosphoramidites and 10 min for LNA, 2'-fluoro and 5-Me-dC phosphoramidites. The EDITH sulfurization time was 5 min. After the synthesis, the solid support-bound oligonucleotides were optionally subject to 5' modification. Cleavage from the solid support and deprotection were achieved by exposure to 35% aqueous ammonia (#10305220, Fisher Scientific) overnight at 55 °C or AMA (35% aqueous ammonia (#10305220, Fisher Scientific) / 40% aqueous methylamine (#426466-4X100ML, Sigma-Aldrich) 1:1 v/v) for 10 min at 65 °C. Obtained oligonucleotides were purified using HPLC (details in the HPLC section) and desalted (details in the nucleic acids desalting section).

For the automation of on-column phosphitylation and coupling, a custom cycle method was written (see Supplementary Cycle Methods) and converted into a machine-readable format using a Python script (see Supplementary Scripts, Section 14).

2.3 **Syringe-based manual synthesis:** For the CPG supported **B**$(dT)_9$ and $(dT)_{10}$ sequence, 0.5 mL of the phosphitylating solution [P–N] and 0.5 mL of the BTT solution were loaded into separate 1 mL syringes and alternately pushed back and forth through the synthesis column. Phosphitylation was carried out for 5 minutes at ambient temperature. For the polystyrene-supported SSQVYSPRPR sequence, 0.9 mL of the phosphitylating solution and 0.9 mL of the BTT solution were loaded into separate 2 mL syringes and pumped back and forth through the synthesis column for 10 minutes. To ensure complete reaction, efficient syringe push–pull mixing was performed every 1–2 minutes during the reaction time. After phosphitylation, the column was washed with 1 mL of MeCN, flushed with argon, and subjected to the coupling step. For the coupling step, alcohol (0.25 M in MeCN) or nucleoside (0.25 M in MeCN/CH_2Cl_2, 8:2 v/v) was premixed 1:1 with 1*H*-tetrazole (0.45 M in MeCN). Only the DMT-dT nucleoside coupling was carried out at 55 °C; all other reactions were conducted at ambient temperature (23 °C) for 30 minutes. For the, CPG supported **B**$(dT)_9$ and $(dT)_{10}$ sequence, 0.5 mL of each reagent was used, while for the polystyrene supported SSQVYSPRPR sequence, 0.9 mL was applied, with syringe mixing every 5 minutes to ensure efficient mixing. Following each coupling step, the resin was washed with 1 mL of MeCN, flushed with argon, and treated with 0.02 M iodine solution (Sigma-Aldrich, product number L060020) for 2–3 minutes at ambient temperature to oxidize P(III) to P(V). Alternatively, CSO (0.5 M in MeCN) was used for 5 min. For sulfurization, 3-ethoxy-1,2,4-dithiazoline-5-one (EDITH, 0.05 M in MeCN) was applied for 5 minutes at ambient temperature (23 ^{o}C). Afterward, the resin was thoroughly

washed with MeCN and flushed with argon. Deprotection was carried out using an AMA solution [1 mL each of 35% NH_3 (aq) and 40% methylamine (aq), mixed in equal volumes] at 65 °C for 10 minutes, unless otherwise specified.

2.4 **Purification of antisense oligonucleotides:** Type I water used in all experiments was produced using Synergy® coupled to RiOs™ 3 Water Purification System (Merck).
Automated high-performance liquid chromatography separations of antisense oligonucleotides and 5'-amino modified oligonucleotides for subsequent NHS ester labelling were carried out on an Agilent 1260 Infinity instrument equipped with an autosampler, a column oven, a diode array detector and a fraction collector. For instrument control, data acquisition and data processing, OpenLab CDS 2.7 (Agilent Technologies) software was used. Ion-pairing reversed-phase separations of oligonucleotides were carried out on Phenomenex Kinetex® EVO C18, 5 µm, 100 Å, 250 x 10.0 mm column (mobile phase A: 100 mM triethylammonium acetate, pH 7.0 in water, mobile phase B: 100 mM triethylammonium acetate, pH 7.0 in water/MeCN 1:1 (v/v), linear gradient: 0-20% B over 1 min, 20-50% B over 16 min, 50-100% B over 0.1 min, then 100% B for 0.9 min, temperature 55 °C, flow rate 5 mL/min, detection at 260 nm).

2.5 **Nucleic acids desalting:** Collected fractions of nucleic acids after HPLC purification were desalted on Vivaspin® Turbo 15 3,000 MWCO (#VS15T92, Sartorius) centrifugal filters following the manufacturer's recommendations. After the first round of centrifugation (concentration of samples), samples were repeatedly diluted with water and concentrated (three times in total).

2.6 **Determination of concentrations of nucleic acids:** Nucleic acids concentrations were calculated using Oligonucleotide Properties Calculator (Kibbe, W. A. OligoCalc: an online oligonucleotide properties calculator. Nucleic Acids Res. 35, W43–W46 (2007).) based on absorbance measured using NanoDrop 2000 spectrophotometer (Thermo Scientific) under the control of NanoDrop 2000/2000c 1.6 software (Thermo Scientific)**.**

2.7 **Mass- spectrometric analysis:** LC-MS analyses of nucleic acids were carried out on a Waters Acquity UHPLC instrument coupled with Waters Xevo G2 QTof mass spectrometer. For instrument control, data acquisition and data processing (including deconvolution of the raw spectra), MassLynx 4.1 software (Waters) was used. Ion-pair

reversed-phase separations of oligonucleotides were carried out on Waters ACQUITY Premier Oligonucleotide C18, 1.7 µm, 130Å, 2.1 x 50 mm column or Waters ACQUITY Premier Protein BEH C4 300 Å 1.7 µm, 2.1 x 50 mm column. Mobile phase A: 200 mM 1,1,1,3,3,3-hexafluoro-2-propanol, 8.15 mM triethylamine, in water/methanol 95:5 (v/v), mobile phase B: mobile phase A/methanol 2:8 (v/v), linear gradient: 100% A for 0.5 min, 0-70% B in A over 7.5 min, 70-0% B in A over 0.1 min, then 100% A for 3.9 min, temperature 55 °C, flow rate 0.2 mL/min, PDA detection at 260 nm (0-10 min). The following tuning parameters were used for the MS data acquisition: negative polarity, capillary voltage 1.8 kV, sampling cone voltage 30 V, extraction cone voltage 2.1 V, source temperature 80 °C, cone gas flow 30 L/h, desolvation gas flow 600 L/h. The MS data were acquired in resolution mode and normal dynamic range, from m/z 340 to 3,000, with 1.0 s scan time. Perfluorotetradecanoic acid was used as a LockSpray reference. The raw mass spectra were deconvolved using the maximum entropy method to produce the true molecular mass spectra.

3.1 Oligonucleotide sequence composition

The following sequences were used for all of our experiments.

1. 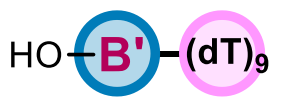

B' = A (**1a**), T (**1b**), G (**1c**), and C (**1d**) (on CPG resin, 25–30 µmol g^{-1} loading)

2. 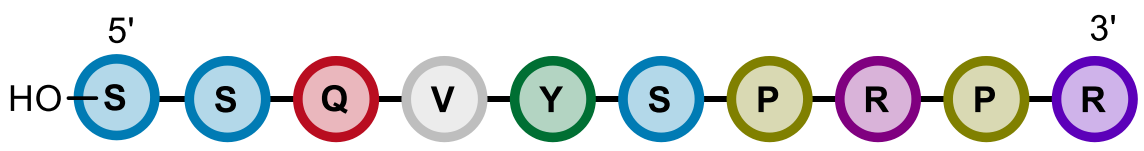

1e; S = [2'OMe_U]; Q = [2'OMe_C]; V = [2'F_A]; Y = [2'F_U]; P = [2'OMe_A]; R = [2'OMe_G]; polystyrene supported, loading: 350 µmol g^{-1} and 35 µmol g^{-1}

3. 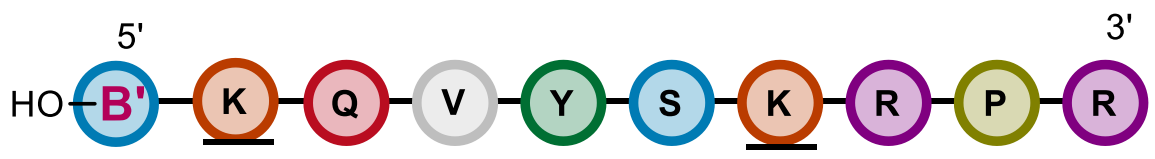

K = [LNA_T], Q = [2'OMe_C], V = [2'F_A], Y = [2'F_U], S = [2'OMe_U], K = [LNA_T], R = [2'OMe_G], P = [2'OMe_A], R = [2'OMe_G]

B' = A (**1f**), T (**1g**), G (**1h**), and C (**1i**); CPG supported, loading: 30–35 µmol g^{-1}

4. 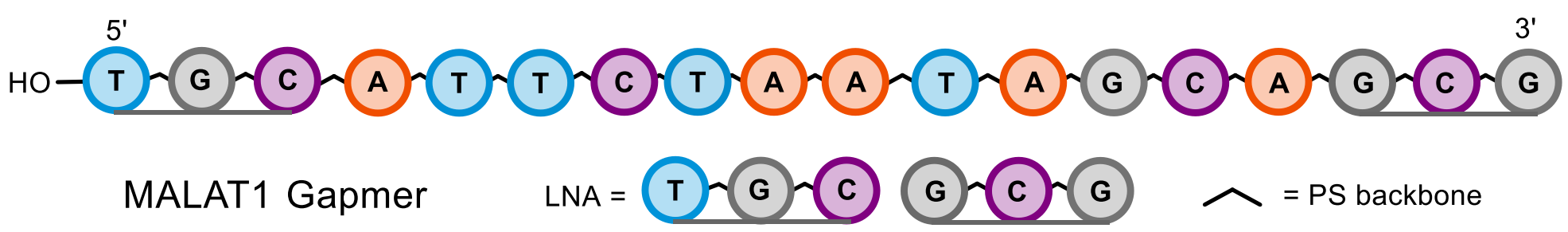

3.2 Spectral analysis of the $(dT)_{10}$ sequence (1b) and mixed sequence (1e)

Ion-pair reversed-phase UHPLC chromatogram of the $(dT)_{10}$ sequence (**1b**) synthesized on CPG support (UV absorbance at 260 nm vs. time in minutes).

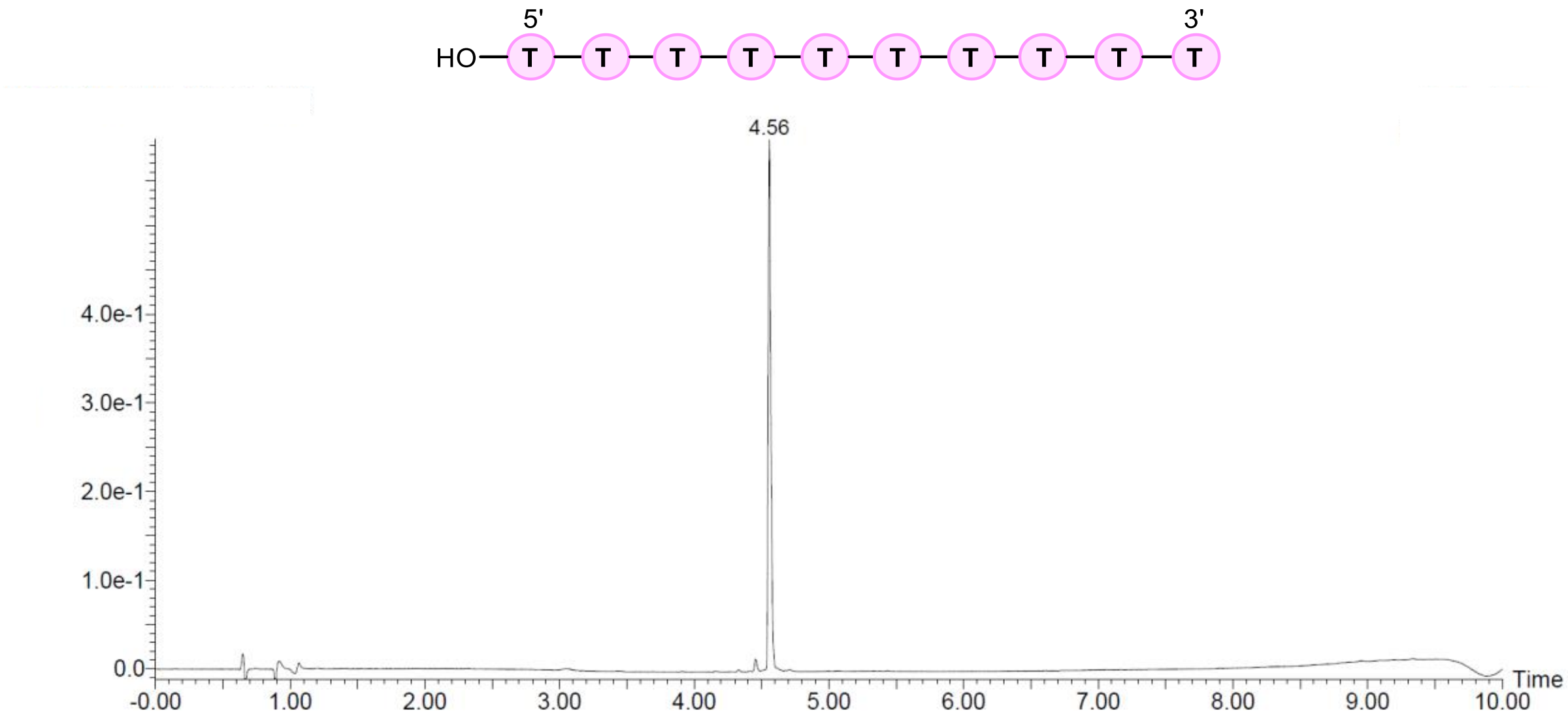

Mass spectrum (ES$^-$) of the $(dT)_{10}$ sequence **(1b)** synthesized on CPG support (y-axis = relative intensity [%], x-axis = deconvolved mass [Da]); 2980.0, found: 2980.5.

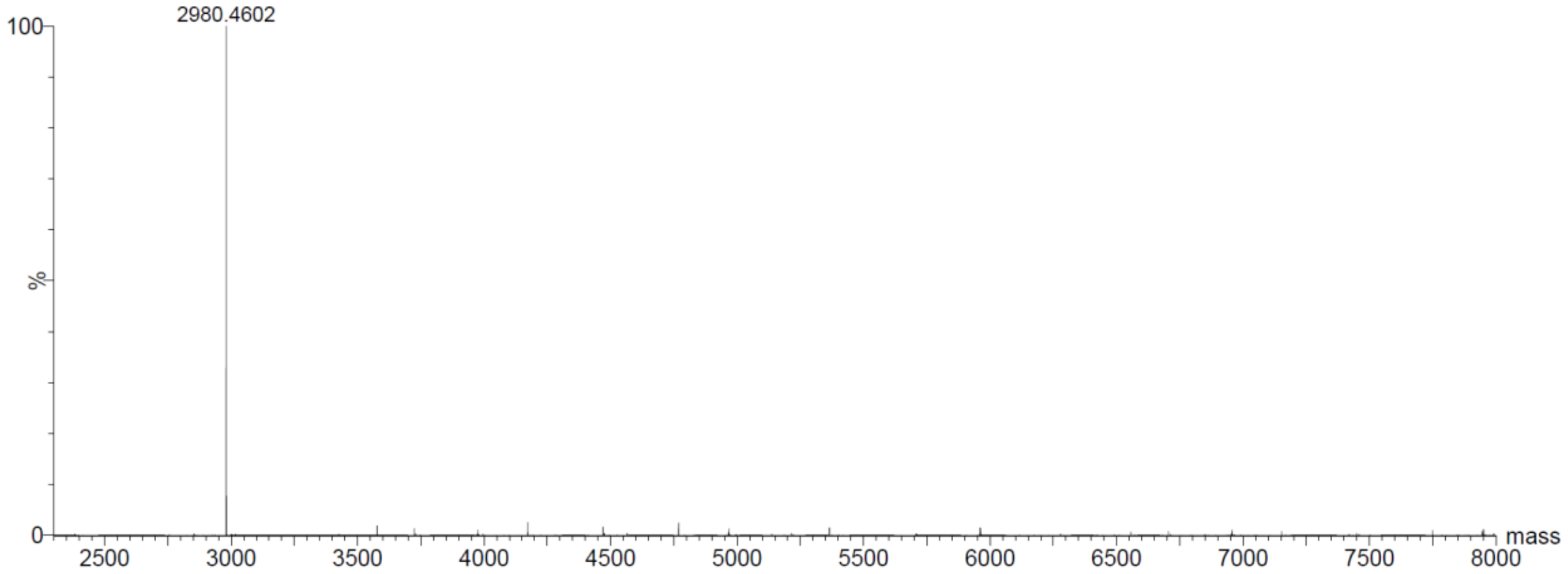

Ion-pair reversed-phase UHPLC chromatogram of the mixed oligonucleotide sequence (**1e**) synthesized on polystyrene support (UV absorbance at 260 nm vs. time in minutes).

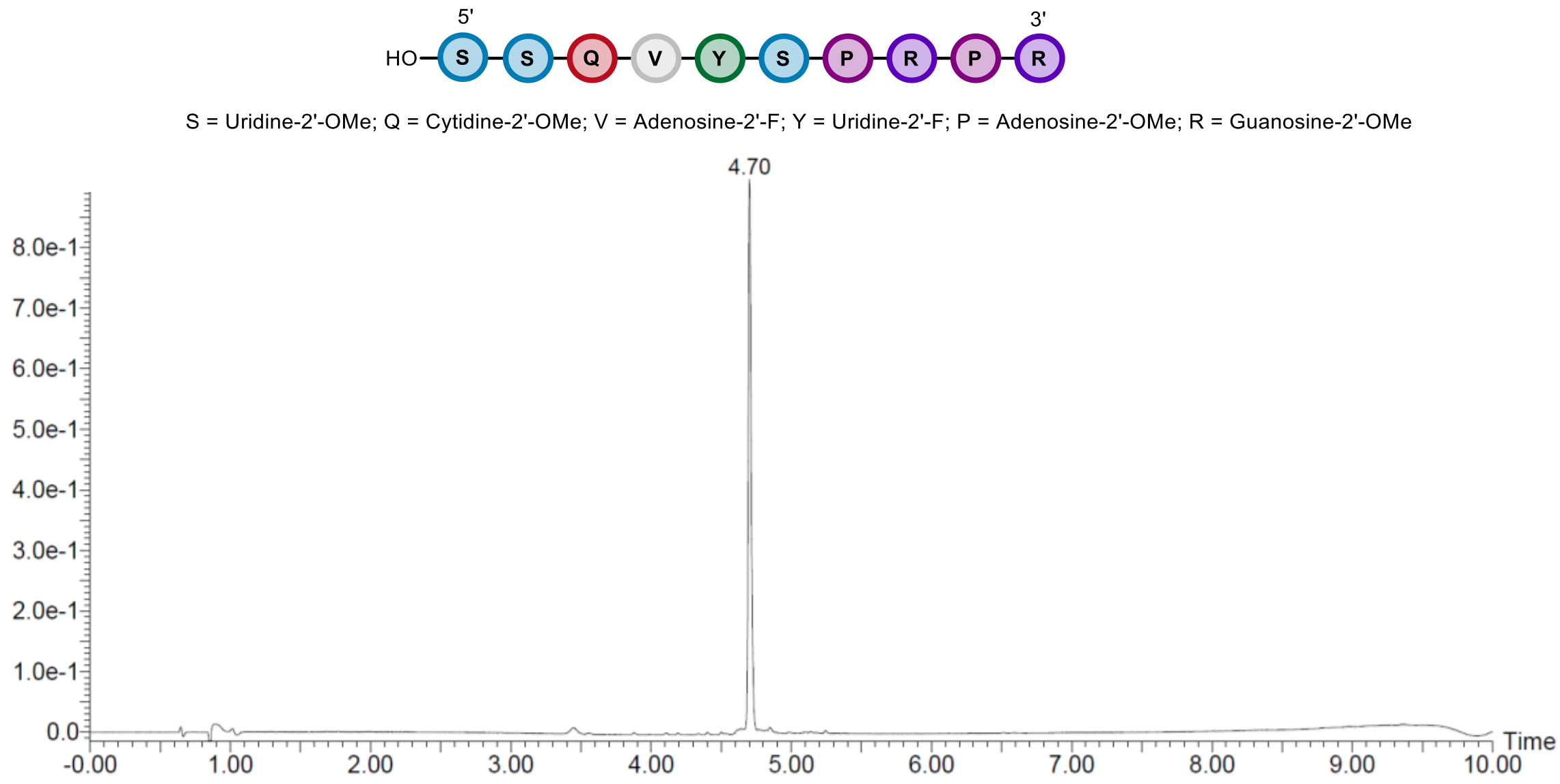

Mass spectrum (ES-) of the mixed oligonucleotide sequence (**1e**) (y-axis = relative intensity [%], x-axis = deconvolved mass [Da]); calculated mass: 3262.1, found: 3262.7.

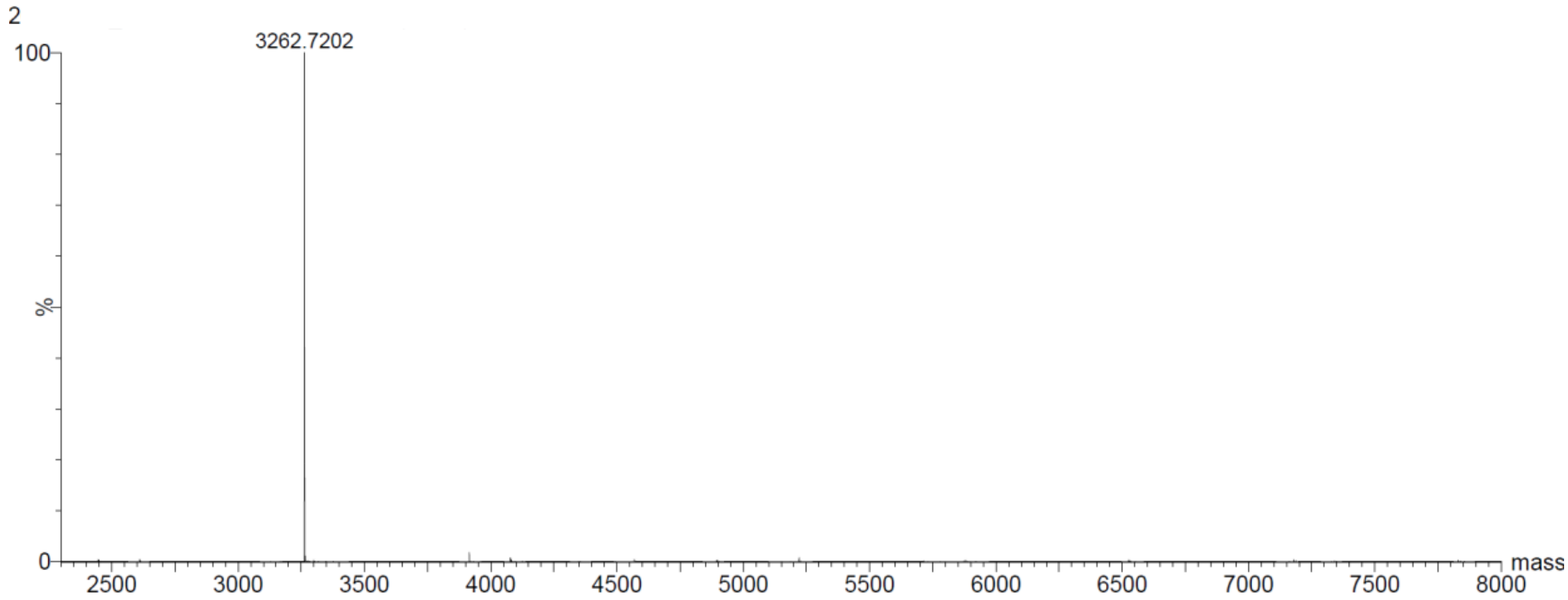

3.3 **Optimizations**

All reactions were performed using either the syringe-based method or the ABI 394 synthesizer, and both approaches yielded equivalent results. The compatibility of the phosphitylation reagent or the reaction with other synthesizers was not investigated.

1-Tetradecanol, hexadecan-1-ol, *cis*-9-octadecen-1-ol and δ-tocopherol were dissolved in MeCN: toluene (1:1, v/v), aminoalcohol was dissolved in MeCN/DMF (8:2 v/v) and DMT-dT was dissolved in MeCN/CH_2Cl_2 (8:2, v/v).

After completing the reaction, whether using the automated synthesizer (ABI 394) or the manual syringe-based method, the ammonia solution was removed by rotary evaporation (55 °C), and the resulting crude product was dissolved in 1 mL of deionized water to prepare the stock solution.

We evaluated the stoichiometry of the phosphitylating reagent [P–N] and alcohol (decan-1-ol) to achieve optimal conversion on an ABI 394 synthesizer. To this end, a PS-based mixed sequence (5.25 µmol) was used for all experiments. The concentrations of the phosphitylating agent (0.3 M in acetonitrile) and 1-decanol (0.25 M) in acetonitrile premixed 1:1 (v/v) with tetrazole (0.45 M) in acetonitrile were kept constant, while the delivery times of these solutions on an ABI 394 synthesizer were varied to test different reagent excesses (see the table and chromatograms below).

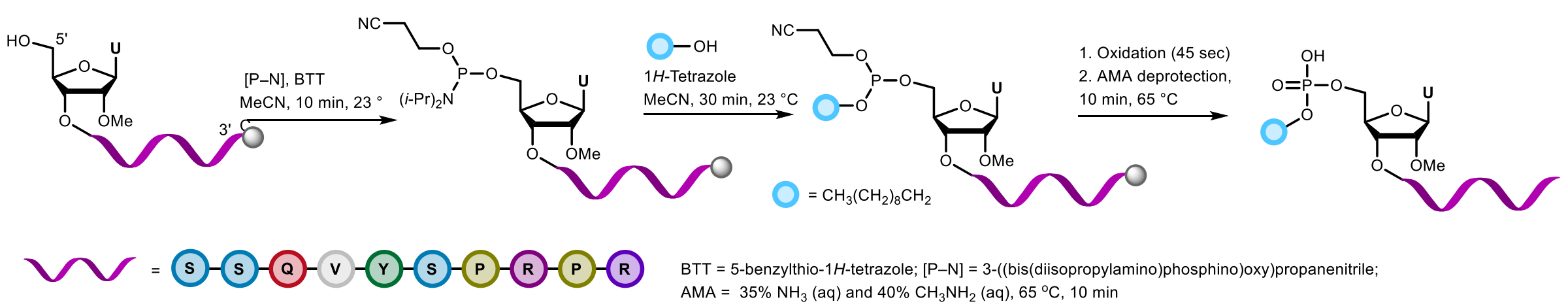

S = Uridine-2'-OMe; Q = Cytidine-2'-OMe; V = Adenosine-2'-F; Y = Uridine-2'-F; P = Adenosine-2'-OMe; R = Guanosine-2'-OMe) sequence (on polystyrene resin, 350 µmol/g and 35 µmol g^{-1} loading)

Entry	[P–N] (equiv.)	decan-1-ol (equiv.)	Conversion (%)
1	22.9	28.6	92
2	17.1	28.6	91
3	11.4	28.6	88
4	22.9	21.4	86
5	22.9	14.3	76

Entry 1

Sequence	rt	Area (A)	Extinction coefficient (ε)	A/ε	Conversion
SSQVYSPRPR	4.734	3519.71	110270	0.031919	
R-SSQVYSPRPR	6.713	42804.832	110270	0.388182	92%

R = decan-1-ol

Entry 2

Sequence	rt	Area (A)	Extinction coefficient (ε)	A/ε	Conversion
SSQVYSPRPR	4.544	1388.031	110270	0.01258757	
SSQVYSPRPR	4.597	807.959	110270	0.0073271	
SSQVYSPRPR	4.637	994.718	110270	0.00902075	
R-SSQVYSPRPR	6.885	32543.594	110270	0.29512645	91%

Entry 3

Sequence	rt	Area (A)	Extinction coefficient (ε)	A/ε	Conversion
SSQVYSPRPR	4.552	1993.424	110270	0.01807766	
SSQVYSPRPR	4.604	910.176	110270	0.00825407	
SSQVYSPRPR	4.644	998.017	110270	0.00905067	
SSQVYSPRPR	5.022	1199.046	110270	0.01087373	
R-SSQVYSPRPR	6.893	36397.594	110270	0.33007703	88%

Entry 4

Sequence	rt	Area (A)	Extinction coefficient (ε)	A/ε	Conversion
SSQVYSPRPR	4.555	1967.074	110270	0.0178387	
SSQVYSPRPR	4.608	2975.431	110270	0.02698314	
SSQVYSPRPR	5.024	576.802	110270	0.00523082	
R-SSQVYSPRPR	6.89	34220.715	110270	0.31033568	86%

Entry 5

Sequence	rt	Area (A)	Extinction coefficient (ε)	A/ε	Conversion
SSQVYSPRPR	4.564	2127.864	110270	0.01929685	
SSQVYSPRPR	4.657	3876.015	110270	0.03515022	
SSQVYSPRPR	5.38	1477.22	110270	0.01339639	
R-SSQVYSPRPR	6.906	23797.609	110270	0.21581218	76%

Stacked ion-pair reversed-phase UHPLC chromatogram of the crude reaction mixture (UV absorbance at 260 nm vs. time in minutes).

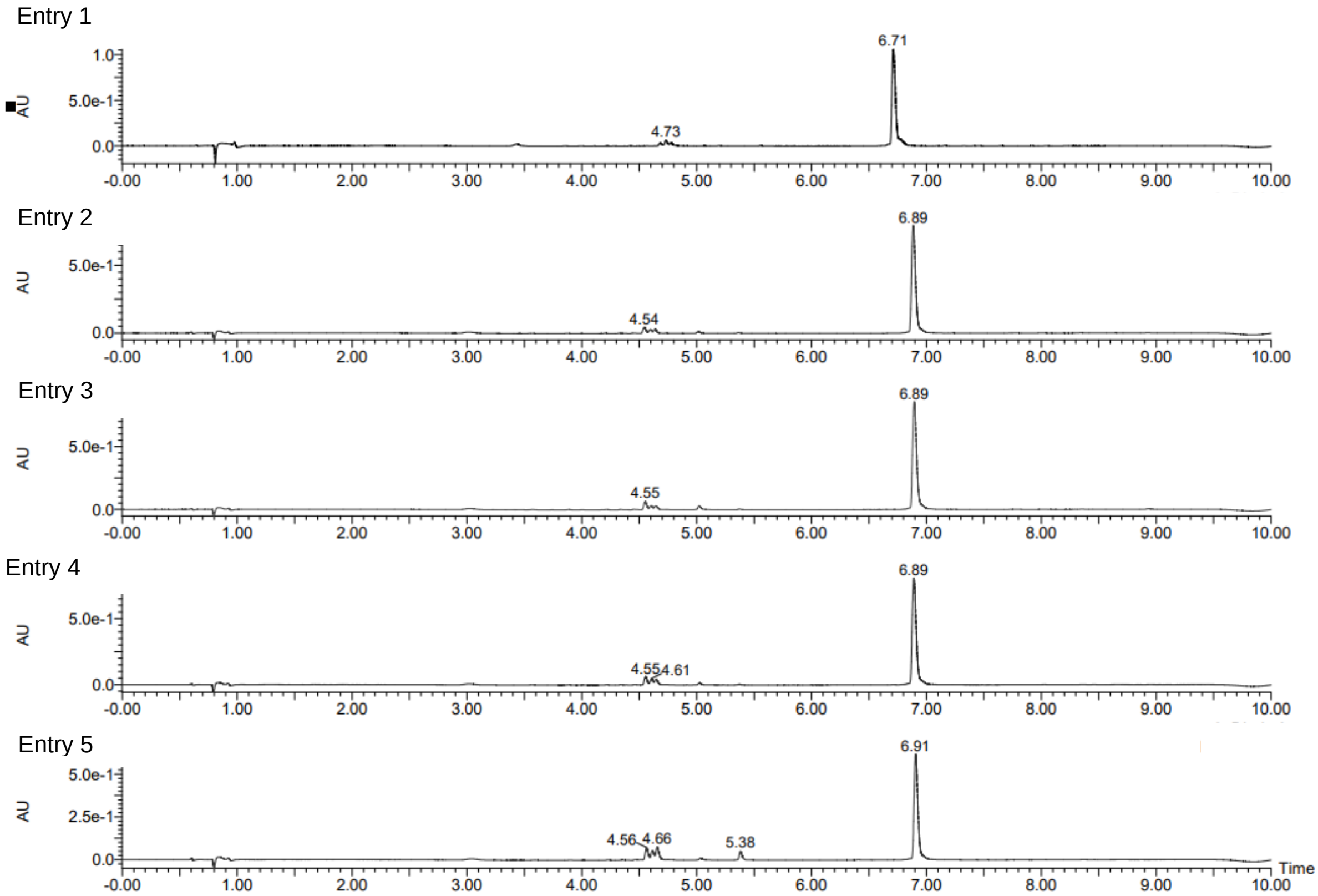

Reduction of the excess of the phisphitylating agent to half (11.4 equiv.) and alcohol to ¾ (21.4 equiv.) was possible without significant reduction in the conversion. These results demonstrate that efficient coupling can indeed be maintained at lower equivalents under optimized delivery conditions.

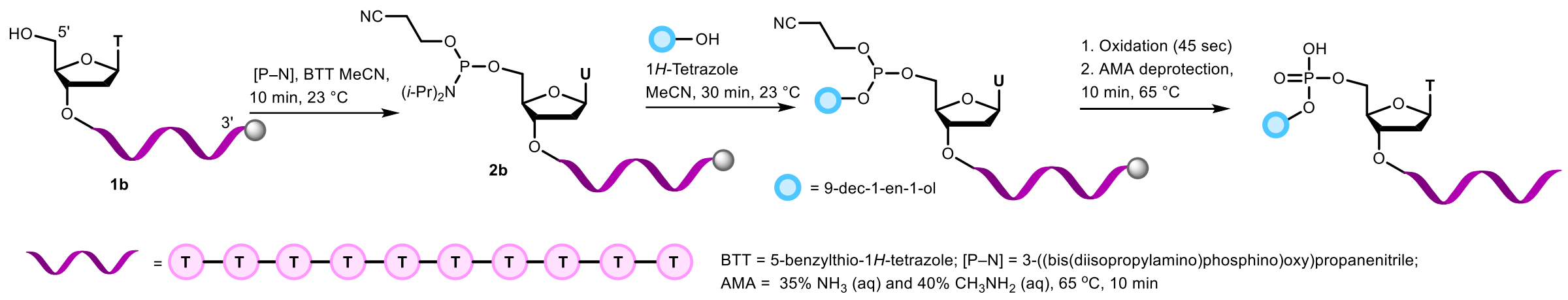

A parallel reaction on an ABI394 synthesiser using a $(dT)_{10}$ sequence confirmed that phosphitylation of **1b** followed by coupling (of the resultant phosphoramidite **2b**) with 9-dec-1-en-1-ol gave 88% conversion with 1*H*-tetrazole (0.45 M), compared to 68% with BTT (0.3 M). BTT was highly effective as an activator for the phosphitylation of the 5'-OH of the oligonucleotide chain, producing near-quantitative conversion to the phosphoramidite intermediates (e.g., **2b**), whereas 1*H*-tetrazole proved superior for subsequent alcohol coupling. This is likely due to the optimal acidity and controlled reactivity of the tetrazolide intermediate compared to that formed from BTT, which facilitates efficient nucleophilic substitution and higher conversion.

Using 1*H*-tetrazole as an activator for the coupling step

**Sequence**	**rt**	**Area (A)**	**Extinction coefficient (ε)**	**A/ε**	**Conversion**
TTTTTTTTTT	4.291	795.418	81600	0.00974777	
TTTTTTTTTT	4.337	1513.403	81600	0.01854661	
TTTTTTTTTT	4.374	703.766	81600	0.00862458	
TTTTTTTTTT	4.469	771.724	81600	0.0094574	
**R**-TTTTTTTTTT	6.456	27182.768	81600	0.33312216	88%

R = 9-dec-1-en-1-ol

Using BTT as an activator for the coupling step

**Sequence**	**rt**	**Area (A)**	**Extinction coefficient (ε)**	**A/ε**	**Conversion**
TTTTTTTTTT	4.296	4295.951	81600	0.05264646	
TTTTTTTTTT	4.337	5422.711	81600	0.06645479	
TTTTTTTTTT	4.373	2377.373	81600	0.02913447	
TTTTTTTTTT	4.469	2091.229	81600	0.02562781	
**R**-TTTTTTTTTT	6.45	29875.25	81600	0.36611826	68%

Stacked ion-pair reversed-phase UHPLC chromatograms of the crude reaction mixtures from the coupling of 9-decen-1-ol with P(III) phosphoramidite **2b** using 1*H*-tetrazole (bottom) and BTT (top) as activators (UV absorbance at 260 nm vs. time in minutes).

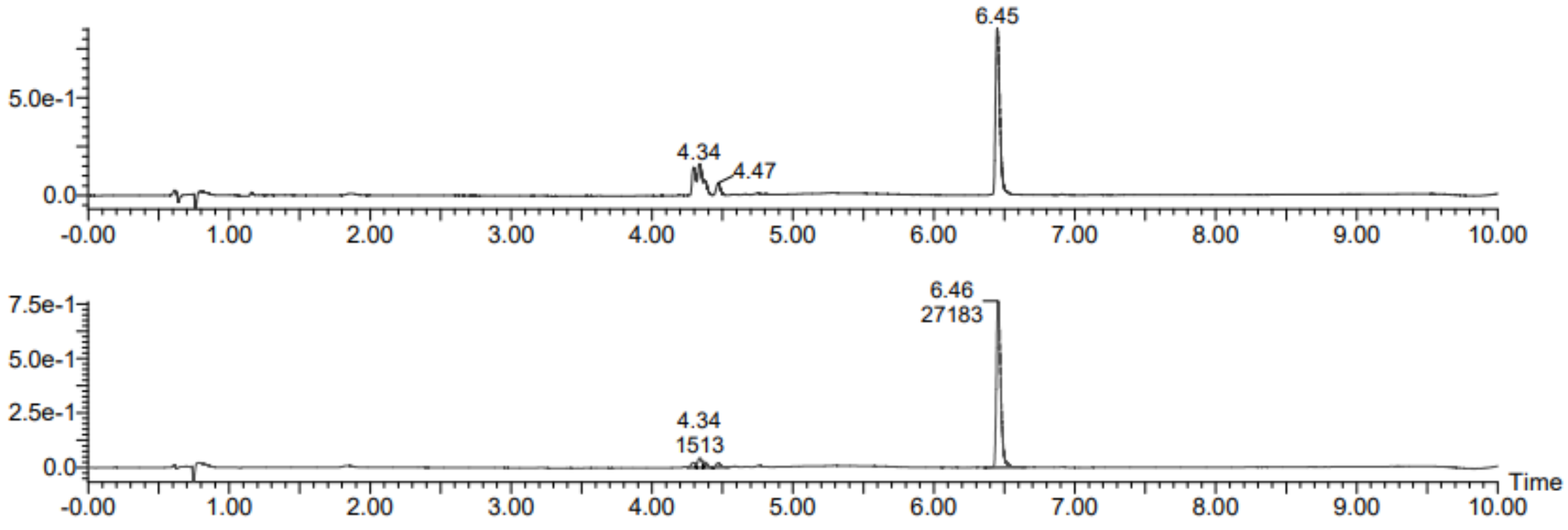

4. 5'-Functionalization of solid-supported oligonucleotides

a

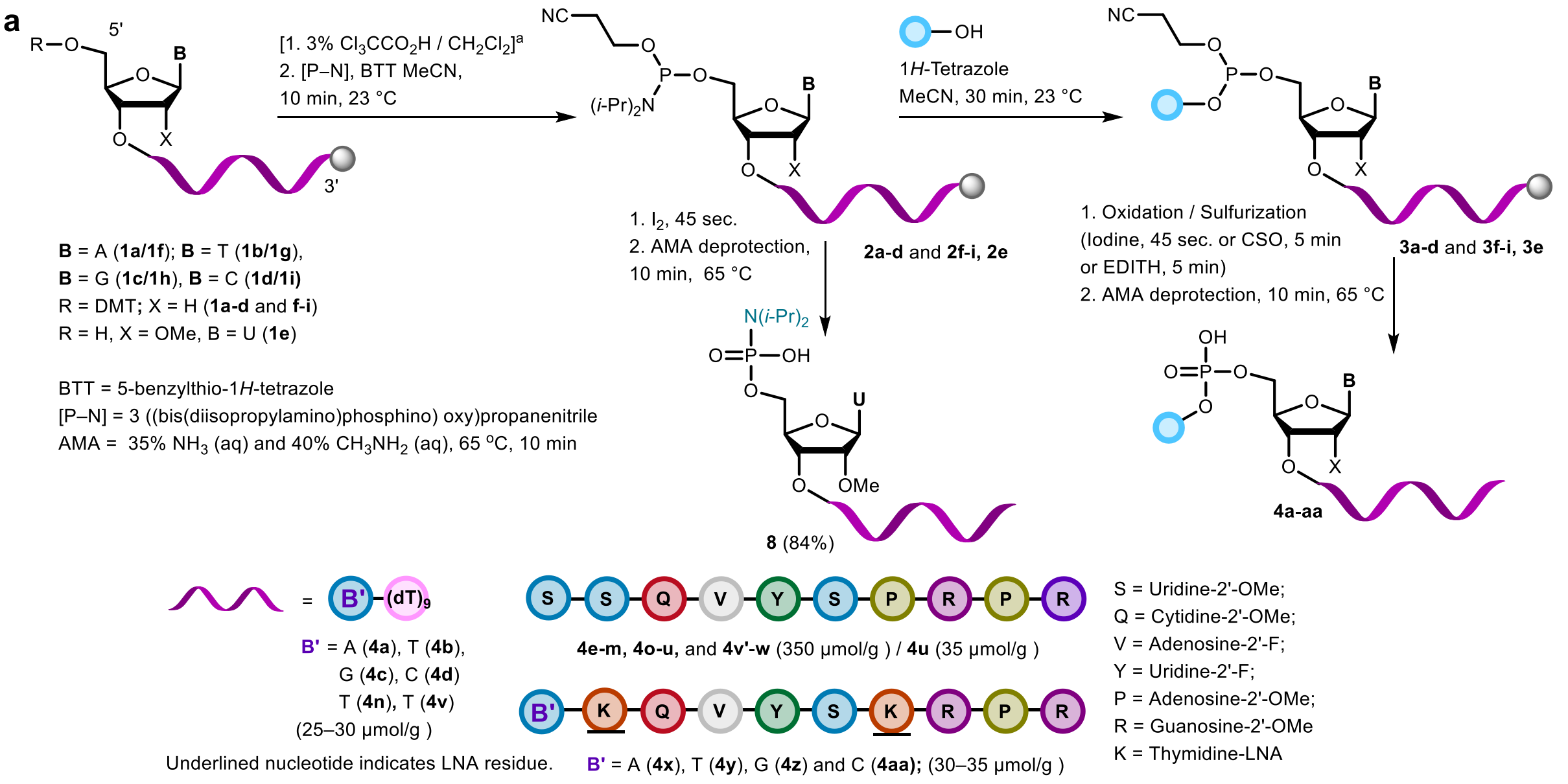

[a]Detritylation performed for oligonucleotides **1a-d** and **1f-i**.

B = nucleobase (protected during solid-phase synthesis; unprotected after cleavage from resin). Underlined nucleobase indicates LNA residue.

Entry	Solid Support	[B]	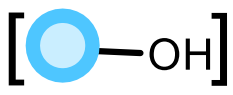	[Ox]	Product	Conversion (%)
1[a]	CPG	A/T/G/C	decan-1-ol	I_2	**4a-d**	86/90/90/88
2	PS	U	decan-1-ol	I_2	**4e**	92
3	PS	U	heptan-1-ol	I_2	**4f**	90
4	PS	U	heptan-1-ol	EDITH	**4f'**	90
5[b]	PS	U	1-tetradecanol	I_2	**4g**	84
6[b]	PS	U	hexadecan-1-ol	I_2	**4h**	81
7[b]	PS	U	*cis*-9-octadecen-1-ol	I_2	**4i**	90
8	PS	U	but-3-yn-1-ol	CSO	**4j**	90
9	PS	U	pent-4-yn-1-ol	CSO	**4k**	92
10	PS	U	hex-5-yn-1-ol	CSO	**4l**	93
11	PS	U	pent-4-en-1-ol	CSO	**4m**	94
12	CPG	U	9-decen-1-ol	I_2	**4n**	88
13	PS	U	2-(2-(2-azidoethoxy)ethoxy)ethan-1-ol	I_2	**4o**	74
14	PS	U	3-(dimethylamino)propan-1-ol	CSO	**4p**	71
15[c]	PS	U	6-aminohexan-1-ol	I_2	**4q**	90
16	PS	U	cyanine-3	CSO	**4r**	41
17	PS	U	6-mercaptohexan-1-ol	CSO	**4s**	-
18	PS	U	6, 6′-disulfanediylbis(hexan-1-ol)	CSO	**4t**	85
19[d]	PS/CPG	U	hexaethylene glycol	I_2	**4u**	88/84
20	PS/CPG	U	phenol	I_2	**4v'/4v**	94/95
21	PS	U	(+)-δ-tocopherol	I_2	**4w**	90
22	CPG	A/T/G/C	decan-1-ol	I_2	**4x-aa**	90/91/91/90

Entries 1–7 and 19–22 were carried out on an ABI 394 synthesizer using the cycle method described in Section 14 (Supplementary Cycle Method), whereas entries 8–18 were conducted manually *via* a syringe technique in accordance with General reaction protocol 1. Both I_2 and CSO oxidation are compatible with our strategy.

For automated synthesis (ABI 394):

i. CPG-supported sequences (1 µmol scale): 120 equiv. of the phosphitylating agent and 150 equiv. of alcohol were employed.

ii. PS-supported sequences (5.25 µmol scale): 22.9 equiv. of the phosphitylating agent and 28.6 equiv. of alcohol were used.

When acetonitrile alone was used to dissolve the alcohol, lower equivalents (e.g., 11.4 equiv. of phosphitylating agent and 21.4 equiv. of alcohol for a 5.25 µmol synthesis on PS-supported sequences were sufficient.

4.1 Functionalization at the 5'-position of a CPG supported **B**(dT)9 sequence using alcohols

Entry 1 (4a)

Sequence	rt	Area (A)	Extinction coefficient (ε)	A/ε	Conversion
TTTTTTTTTT	4.411	2521.313	88200	0.028586315	
TTTTTTTTTT	4.458	1255.676	88200	0.014236689	
TTTTTTTTTT	4.507	660.459	88200	0.007488197	
**R-A**TTTTTTTTT	6.271	27065.88	88200	0.306869354	86%

[R = $CH_3(CH_2)_9O$]

Ion-pair reversed-phase UHPLC chromatogram of the crude reaction (UV absorbance at 260 nm vs. time in minutes).

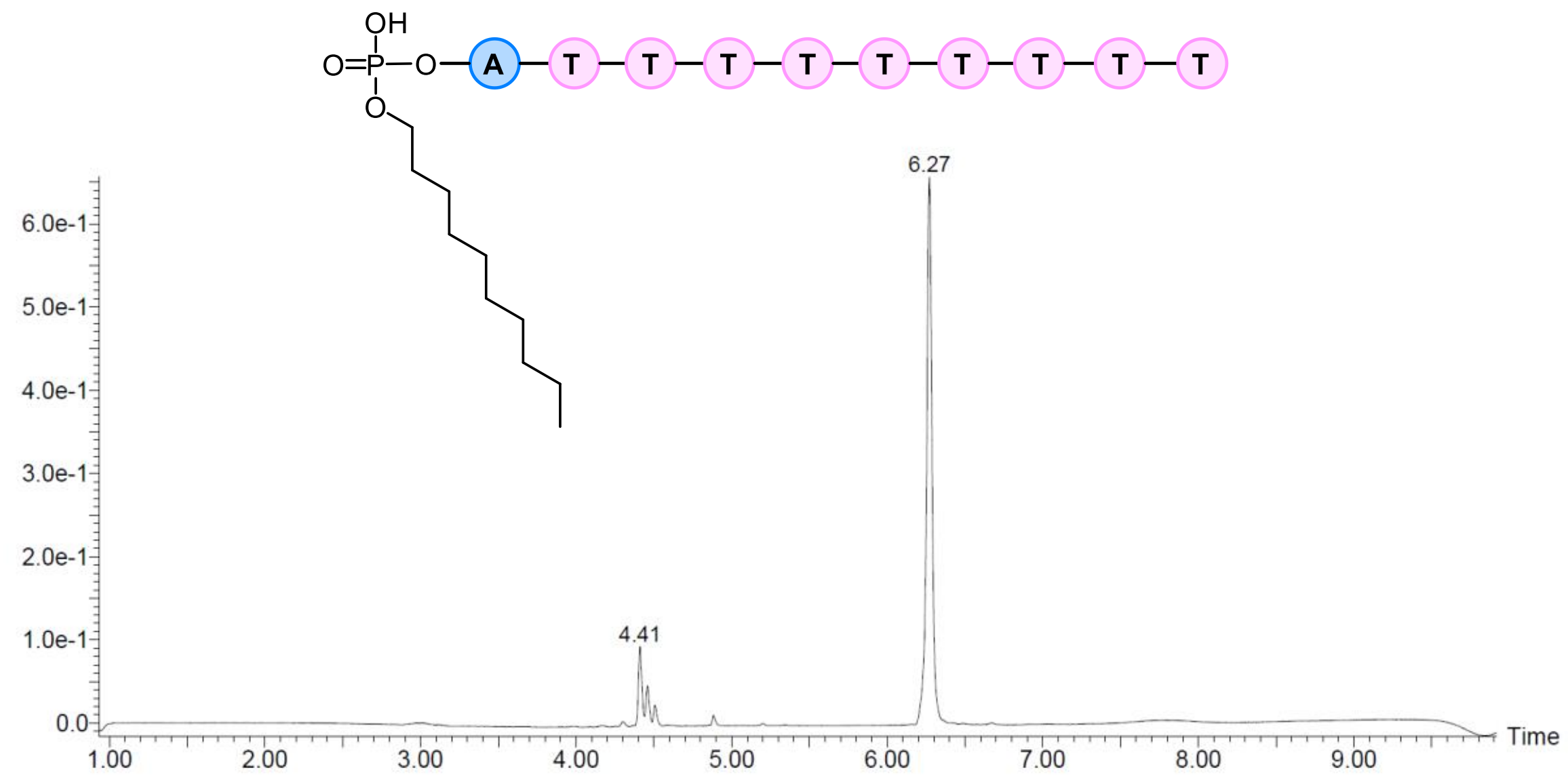

Mass spectrum (ES-) of **4a** (y-axis = relative intensity [%], x-axis = deconvolved mass [Da]); calculated mass: 3209.1, found: 3207.3.

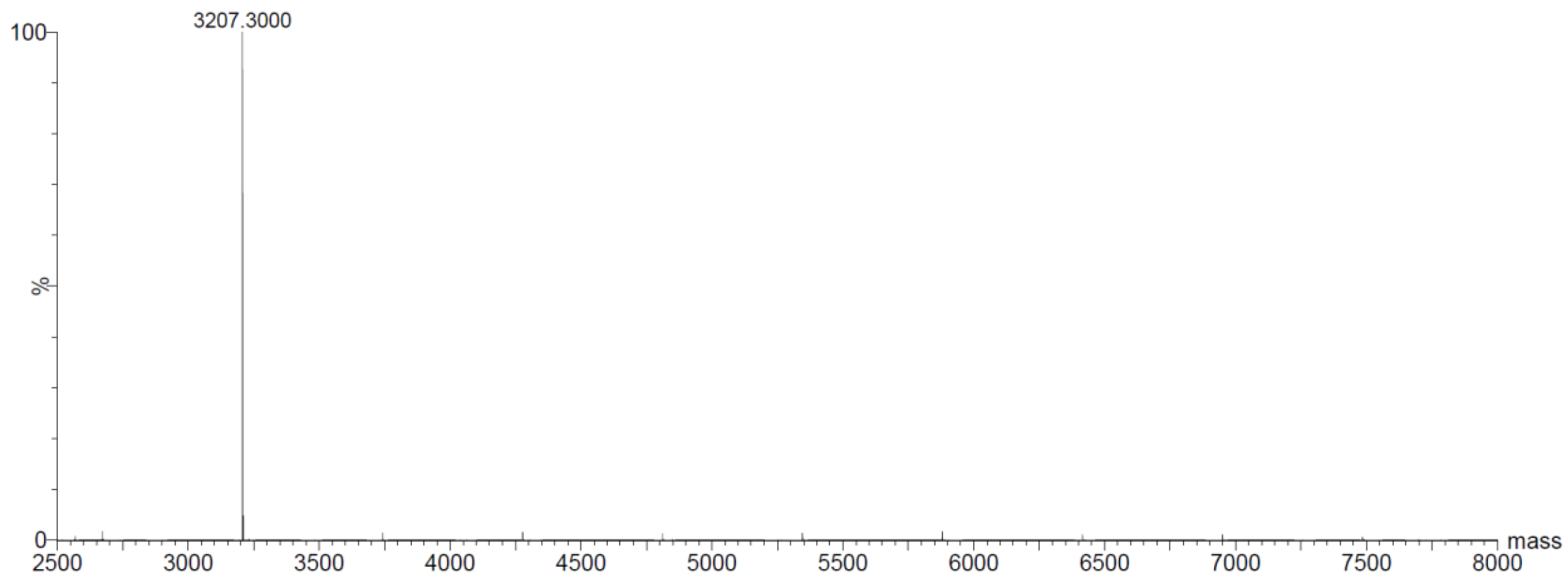

Entry 1 (4b)

Sequence	rt	Area (A)	Extinction coefficient (ε)	A/ε	Conversion
TTTTTTTTTT	4.434	477.774	81600	0.005855074	
TTTTTTTTTT	4.492	288.53	81600	0.003535907	
TTTTTTTTTT	4.531	176.007	81600	0.002156949	
R-TTTTTTTTTT	6.495	8732.724	81600	0.107018676	90%

[R = $CH_3(CH_2)_9O$]

Ion-pair reversed-phase UHPLC chromatogram of the crude reaction (UV absorbance at 260 nm vs. time in minutes)

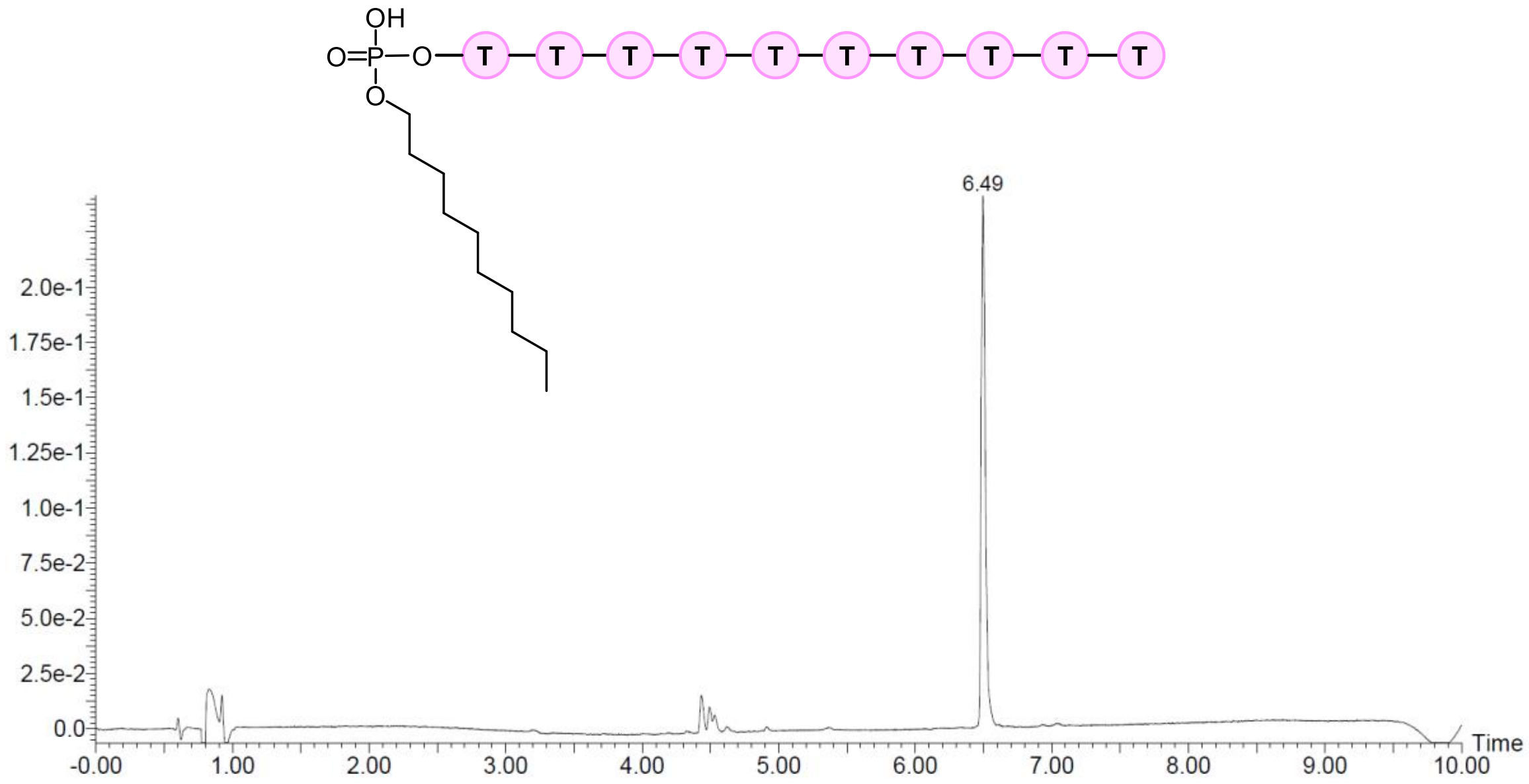

Mass spectrum (ES^-) of **4b** (y-axis = relative intensity [%], x-axis = deconvolved mass [Da]); calculated mass: 3198.2, found: 3198.9.

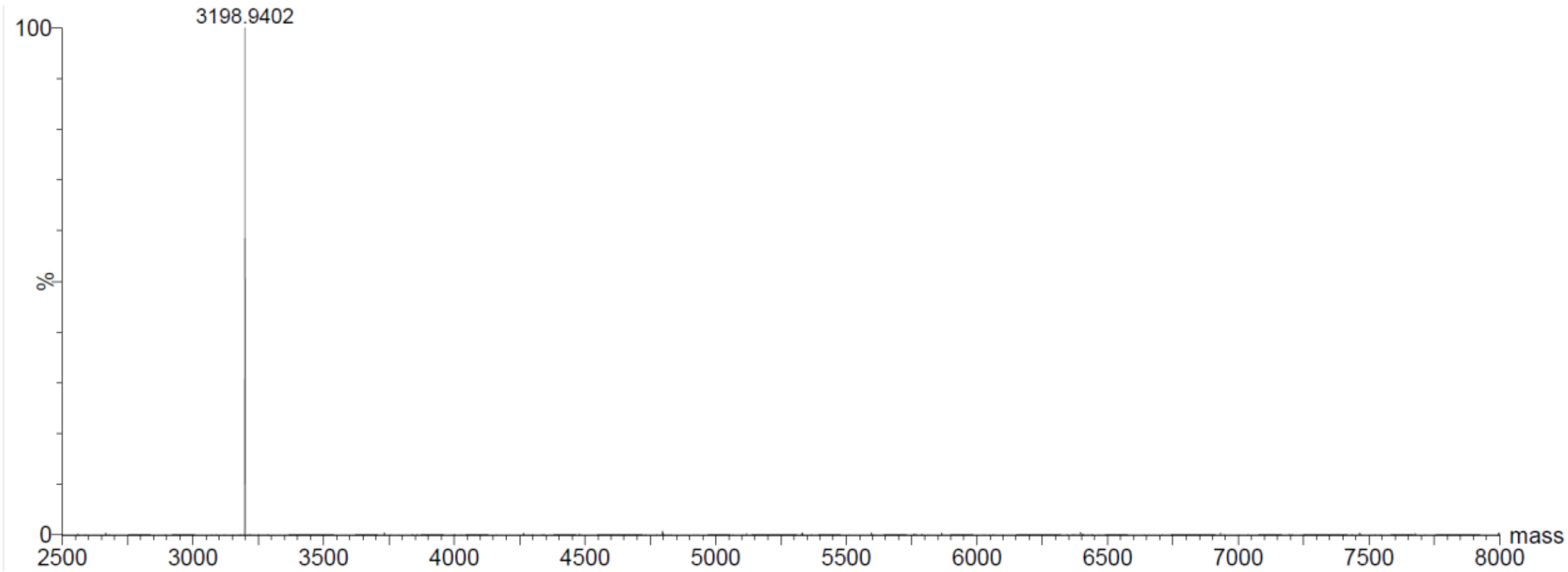

Entry 1 (4c)

Sequence	rt	Area (A)	Extinction coefficient (ε)	A/ε	Conversion
TTTTTTTTTT	4.358	763.461	83800	0.009110513	
TTTTTTTTTT	4.438	424.007	83800	0.005059749	
**R-G**TTTTTTTTT	6.209	11252.309	83800	0.134275764	90%

[R = $CH_3(CH_2)_9O$]

Ion-pair reversed-phase UHPLC chromatogram of the crude reaction mixture (UV absorbance at 260 nm vs. time in minutes).

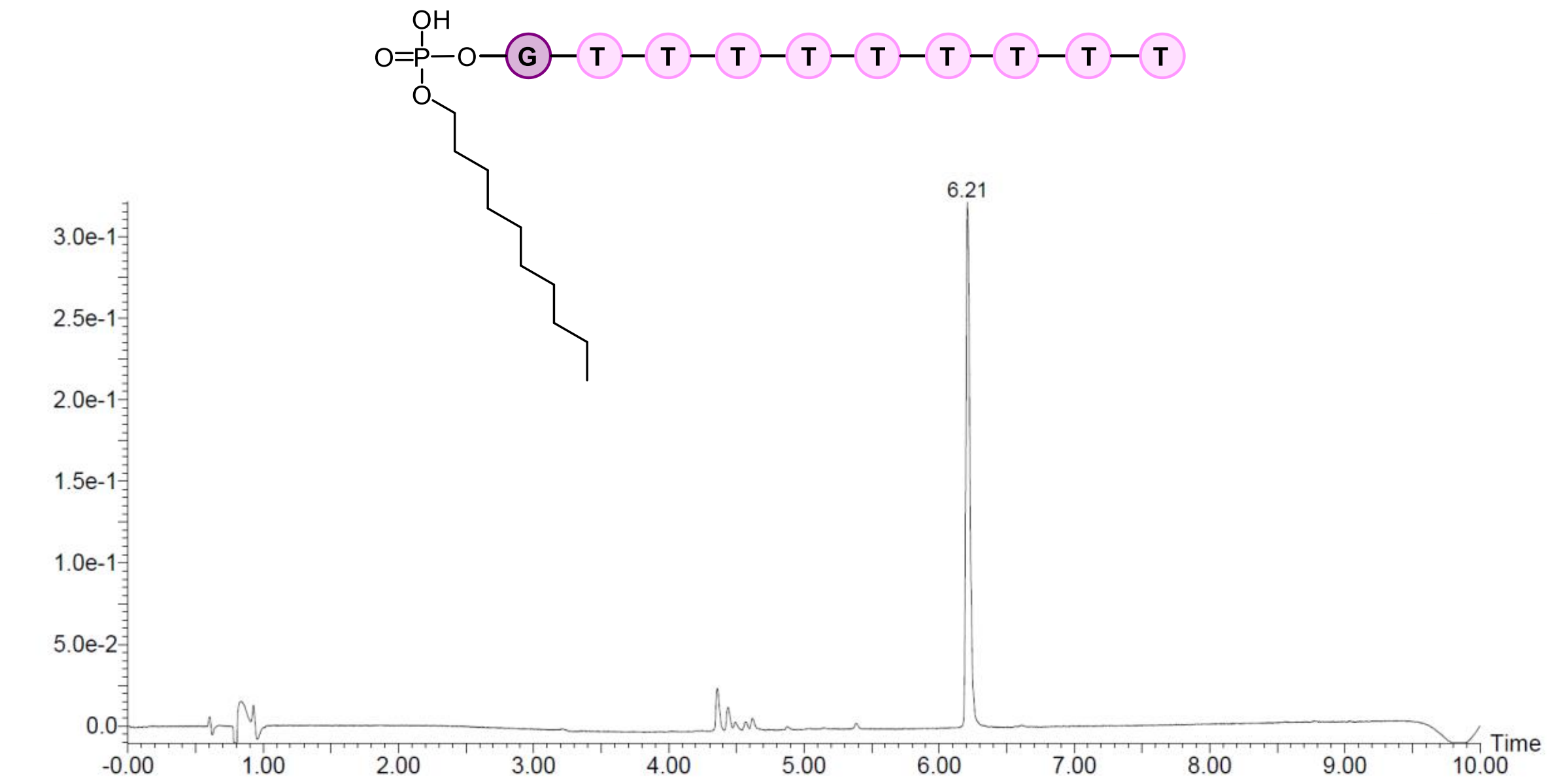

Mass spectrum (ES$^-$) of **4c** (y-axis = relative intensity [%], x-axis = deconvolved mass [Da]); calculated mass: 3223.2, found: 3223.7.

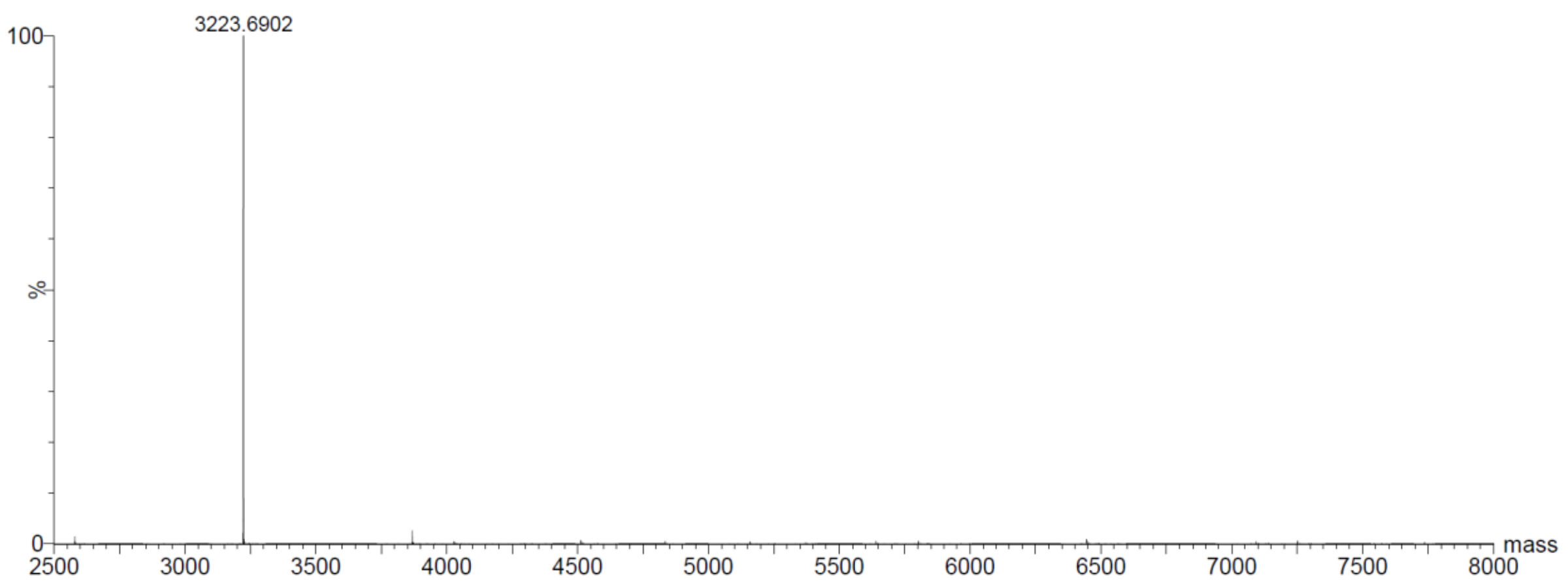

Entry 1 (4d)

Sequence	rt	Area (A)	Extinction coefficient (ε)	A/ε	Conversion
TTTTTTTTTT	4.334	1074.588	81000	0.013266519	
TTTTTTTTTT	4.418	678.726	81000	0.008379333	
TTTTTTTTTT	4.469	408.494	81000	0.005043136	
R-CTTTTTTTTT	6.277	15342.435	81000	0.189412778	88%

[R = $CH_3(CH_2)_9O$]

Ion-pair reversed-phase UHPLC chromatogram of the crude reaction mixture (UV absorbance at 260 nm vs. time in minutes).

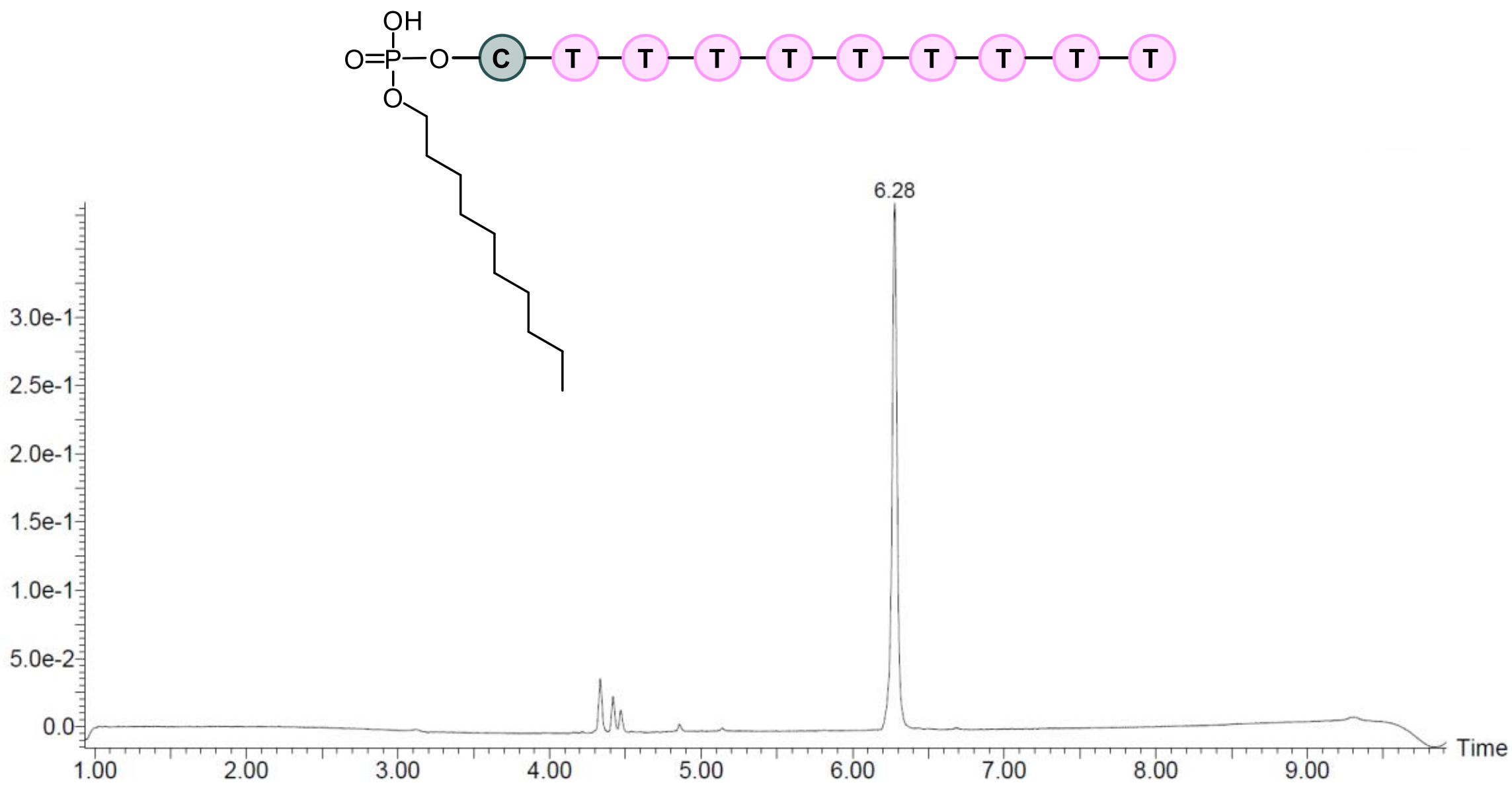

Mass spectrum (ES-) of **4d** (y-axis = relative intensity [%], x-axis = deconvolved mass [Da]); calculated mass: 3183.2, found: 3183.3.

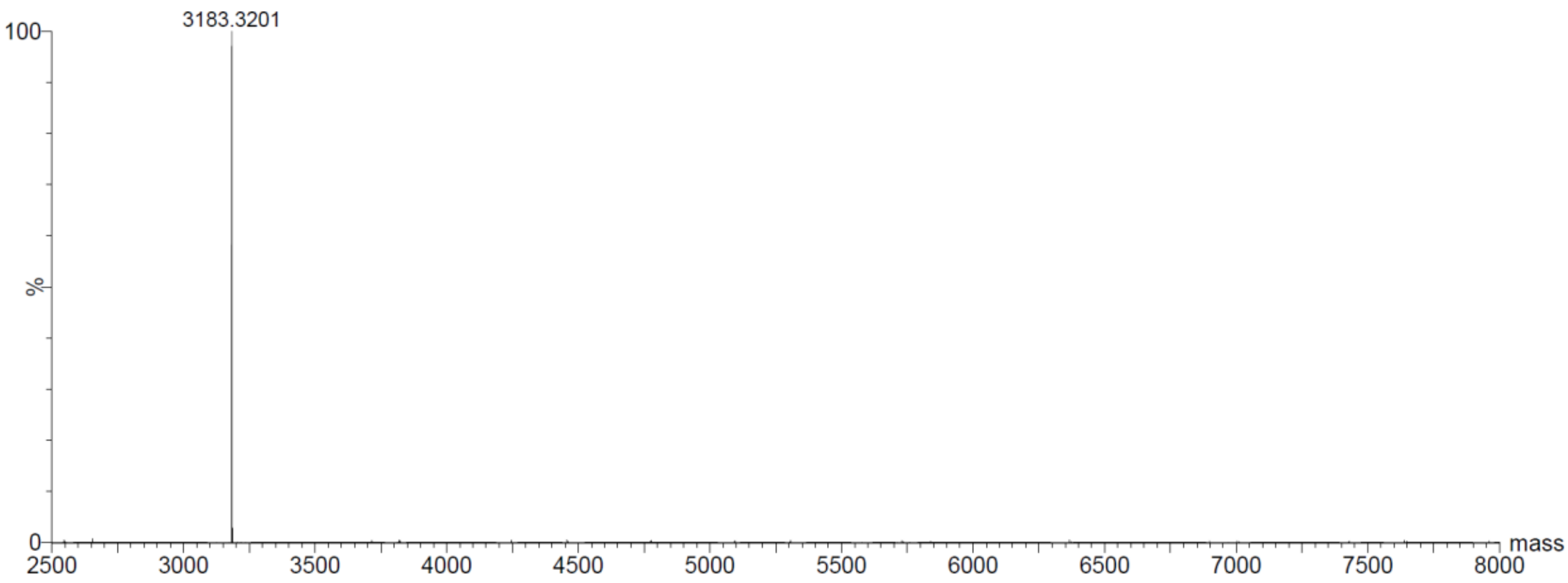

4.2 Functionalization at the 5'-position of a polystyrene supported mixed sequence

Entry 2 (4e)

Sequence	rt	Area (A)	Extinction coefficient (ε)	A/ε	Conversion
SSQVYSPRPR	4.734	3519.71	110270	0.031919	
R-SSQVYSPRPR	6.713	42804.832	110270	0.388182	92%

Ion-pair reversed-phase UHPLC chromatogram of the crude reaction mixture (UV absorbance at 260 nm vs. time in minutes). [R = $CH_3(CH_2)_9O$]

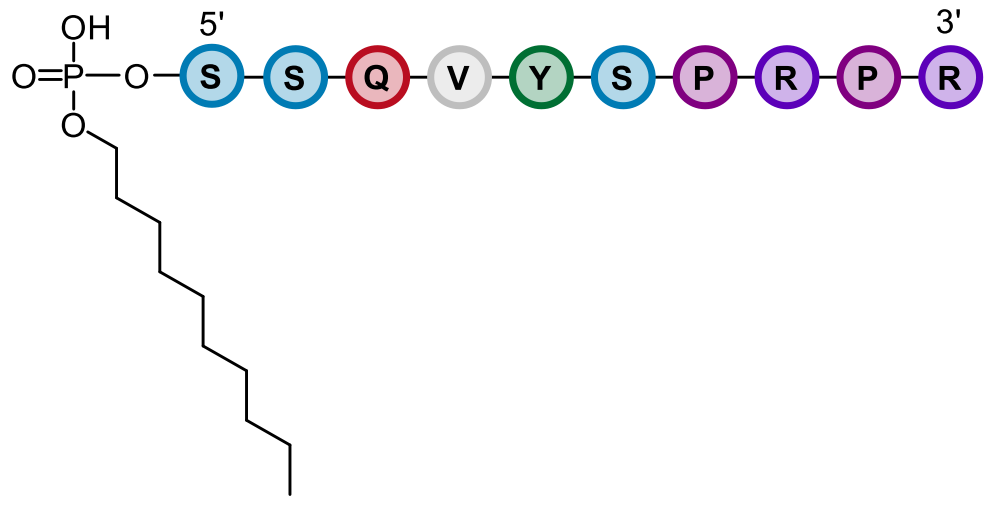

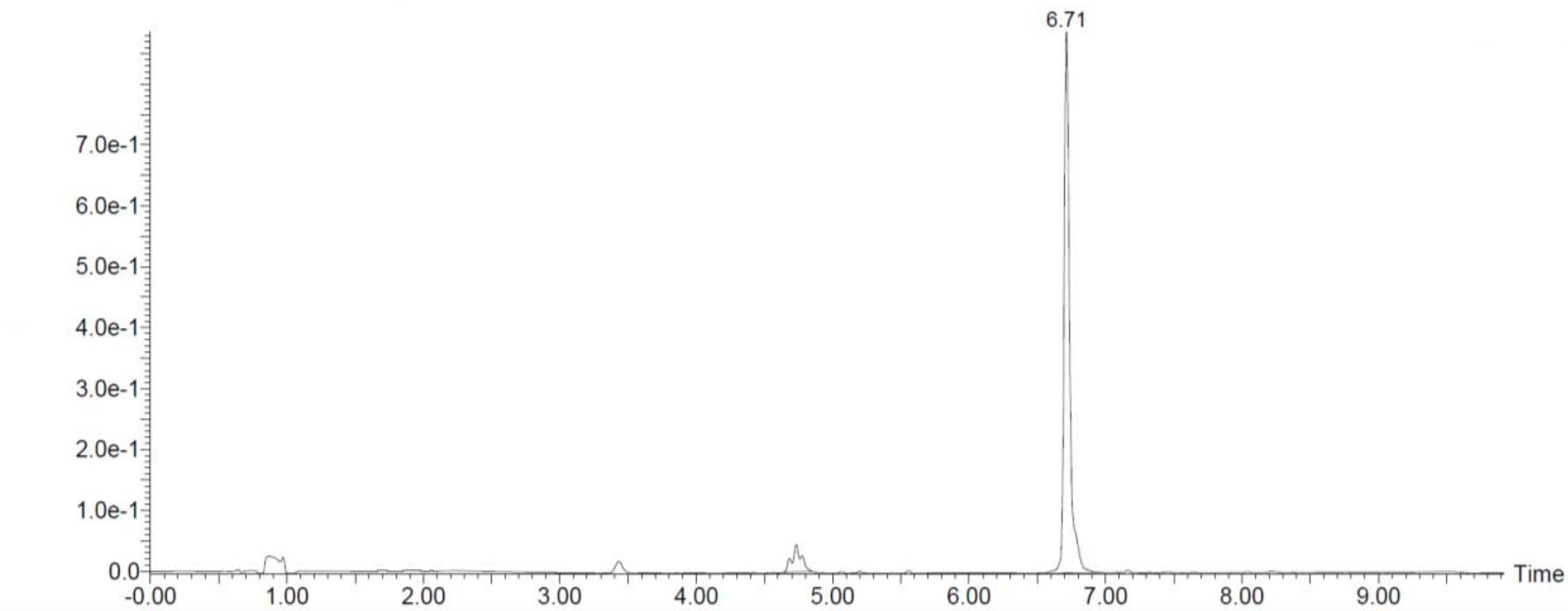

Mass spectrum (ES-) of **4e** (y-axis = relative intensity [%], x-axis = deconvolved mass [Da]); calculated mass: 3482.3, found: 3480.7.

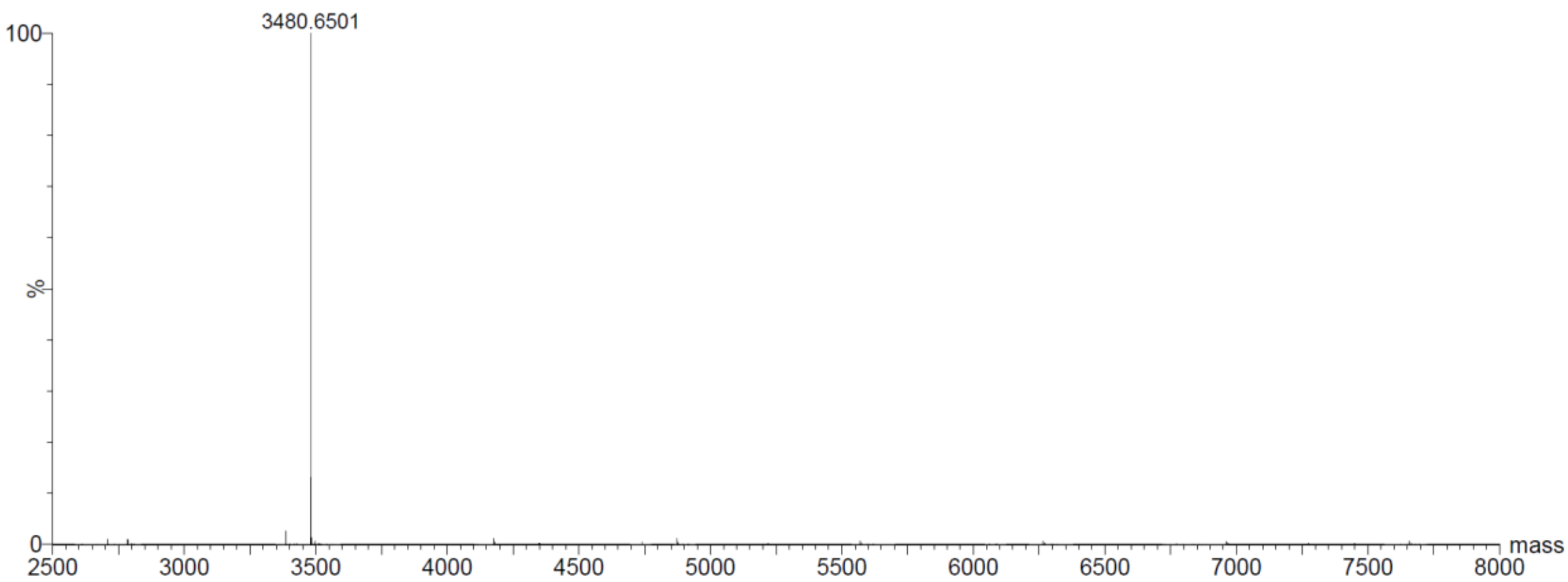

Entry 3 (4f)

Sequence	rt	Area (A)	Extinction coefficient (ε)	A/ε	Conversion
SSQVYSPRPR	4.734	2636.038	110270	0.0239053	
R-SSQVYSPRPR	6.043	22628.316	110270	0.2052083	90%

[R = $CH_3(CH_2)_6O$]

Ion-pair reversed-phase UHPLC chromatogram of the crude reaction mixture (UV absorbance at 260 nm vs. time in minutes).

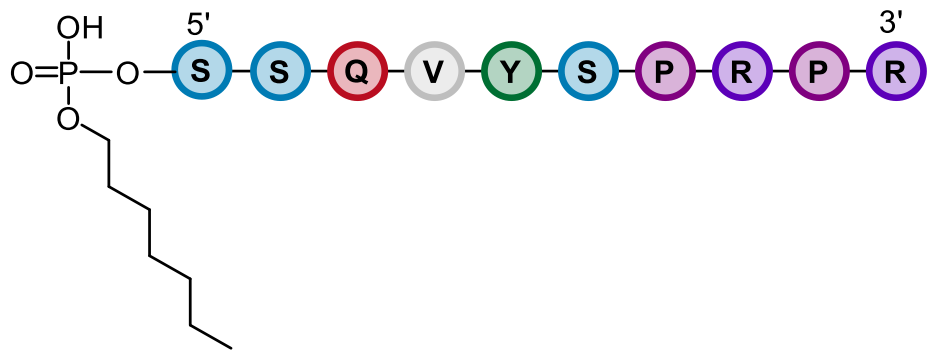

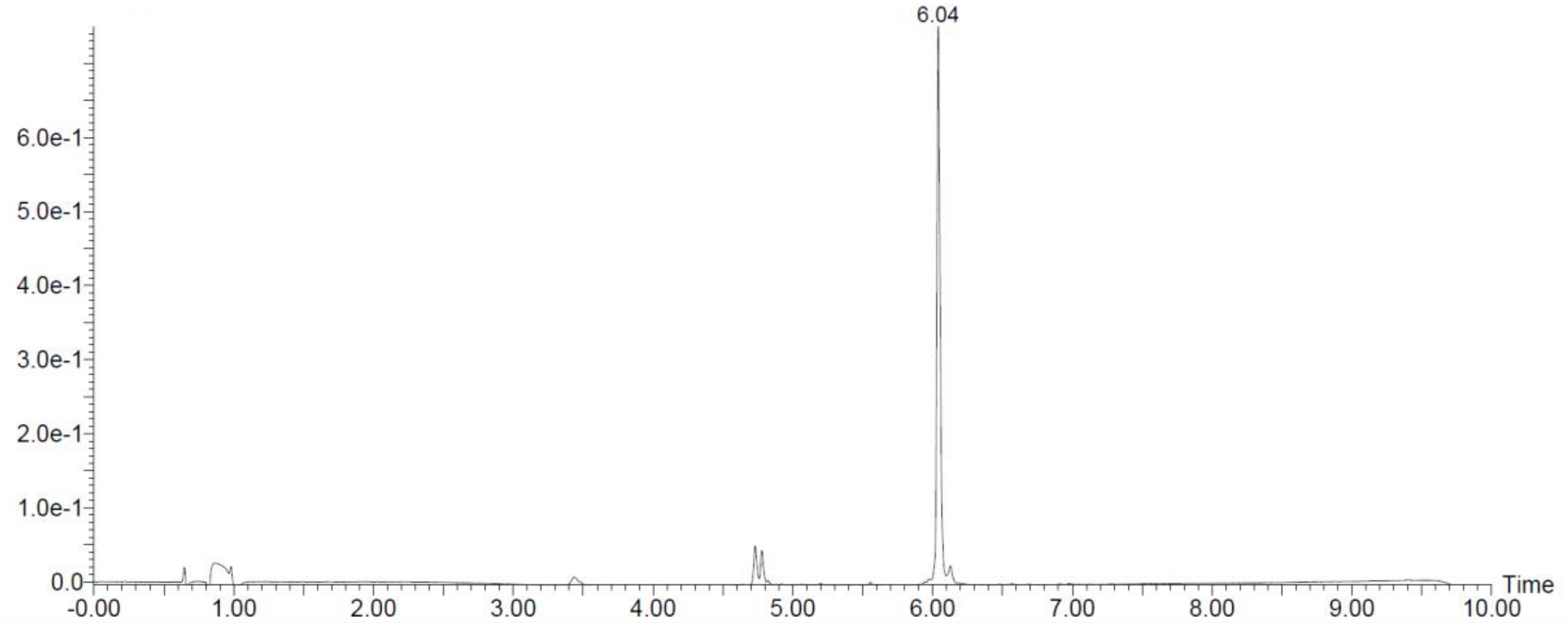

Mass spectrum (ES-) of **4f** (y-axis = relative intensity [%], x-axis = deconvolved mass [Da]); calculated mass: 3440.2, found: 3439.9.

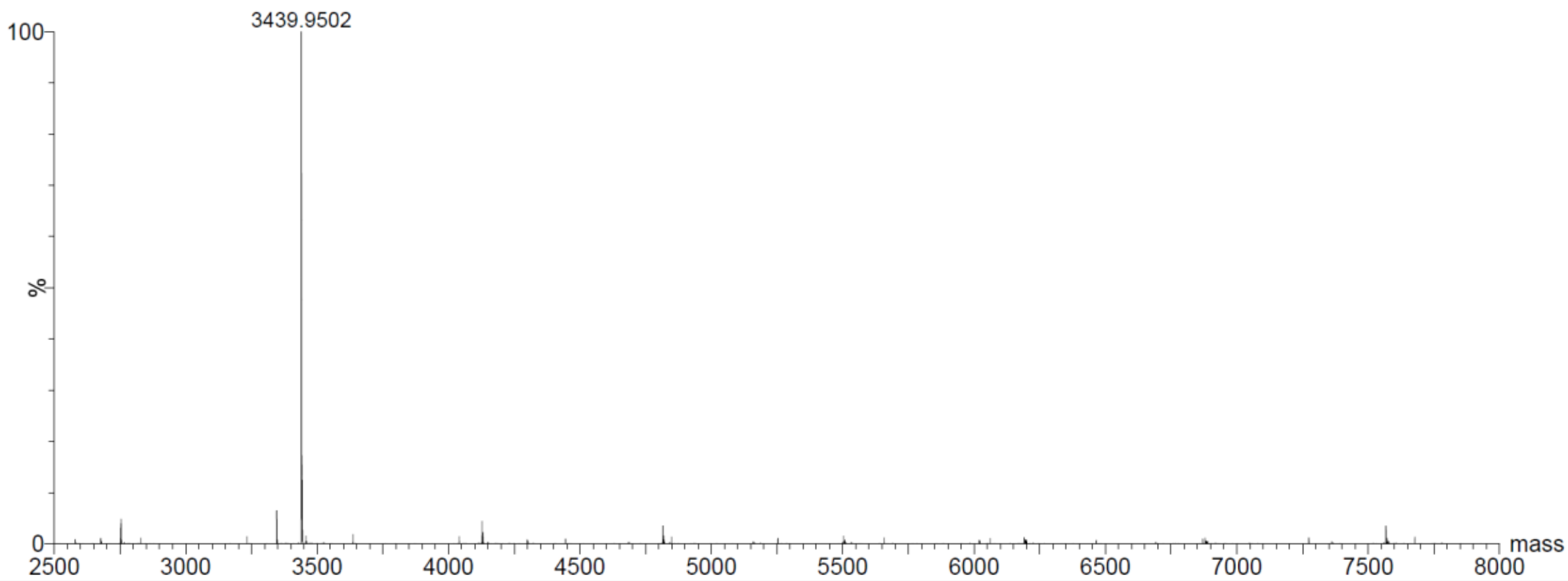

Entry 4 (4f')

Sequence	rt	Area (A)	Extinction coefficient (ε)	A/ε	Conversion
SSQVYSPRPR	4.657	1979.636	110270	0.017952625	
SSQVYSPRPR	4.754	2898.045	110270	0.026281355	
**R-**SSQVYSPRPR	6.087	44618.32	110270	0.404627913	90%

[R = $CH_3(CH_2)_6O$]

Ion-pair reversed-phase UHPLC chromatogram of the crude reaction mixture (UV absorbance at 260 nm vs. time in minutes).

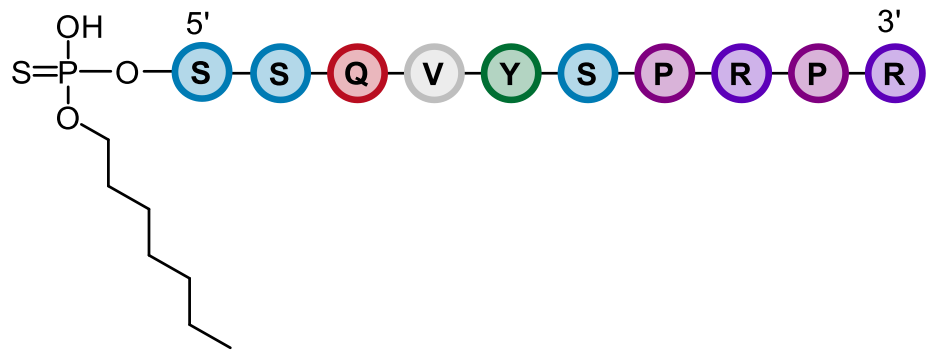

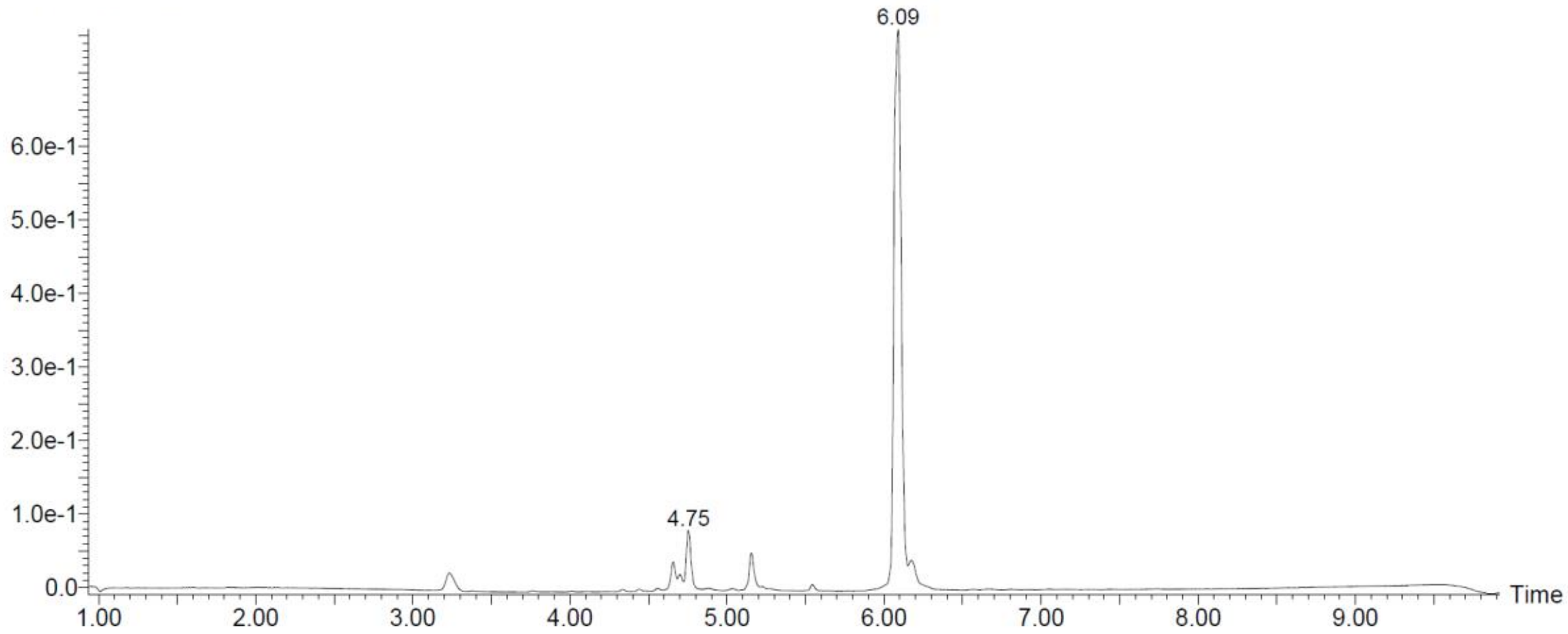

Mass spectrum (ES-) of **4f'** (y-axis = relative intensity [%], x-axis = deconvolved mass [Da]); calculated mass: 3456.2, found: 3455.4.

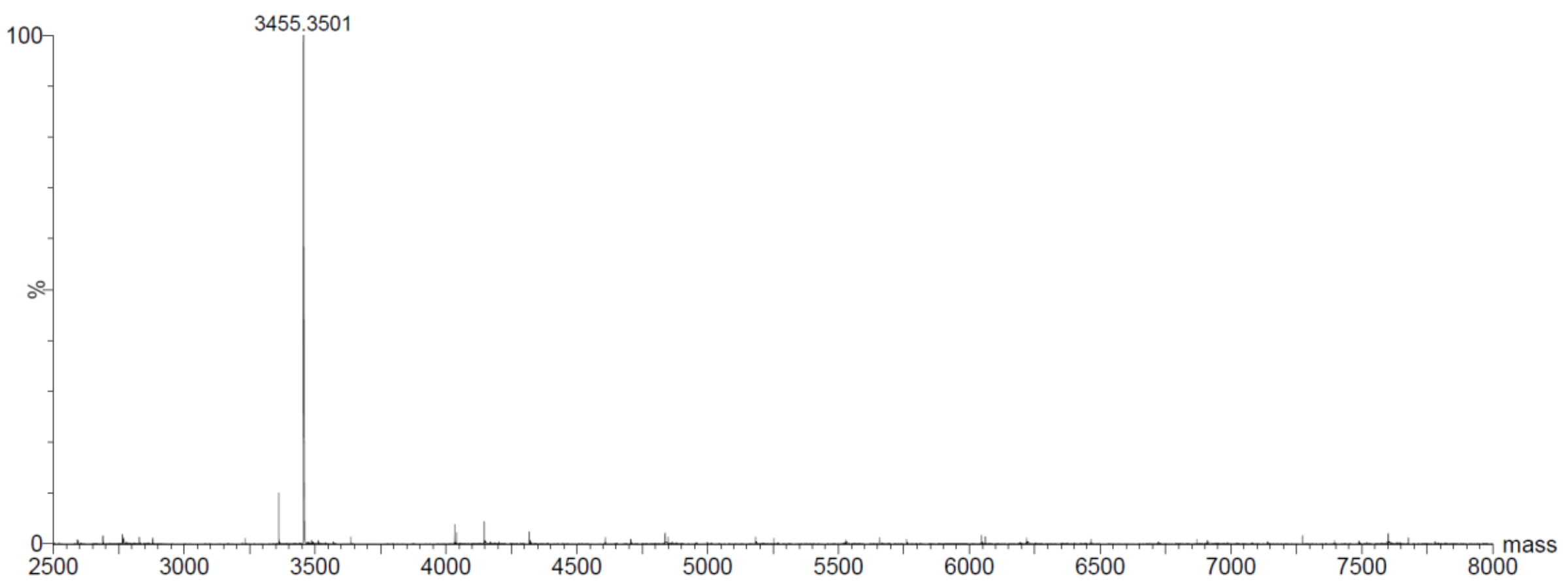

Entry 5 (4g)

Sequence	rt	Area (A)	Extinction coefficient (ε)	A/ε	Conversion
SSQVYSPRPR	4.563	2083.023	110270	0.01889021	
SSQVYSPRPR	4.656	3543.271	110270	0.03213268	
**R-**SSQVYSPRPR	8.927	30324.795	110270	0.27500494	84%

[R = $CH_3(CH_2)_{13}O$]

Ion-pair reversed-phase UHPLC chromatogram of the crude reaction mixture (UV absorbance at 260 nm vs. time in minutes).

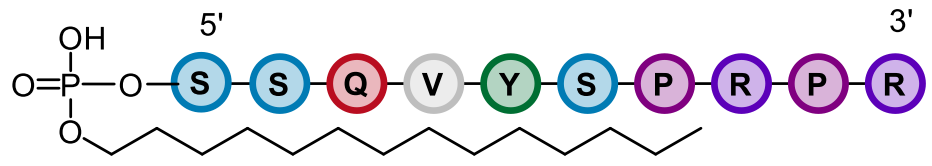

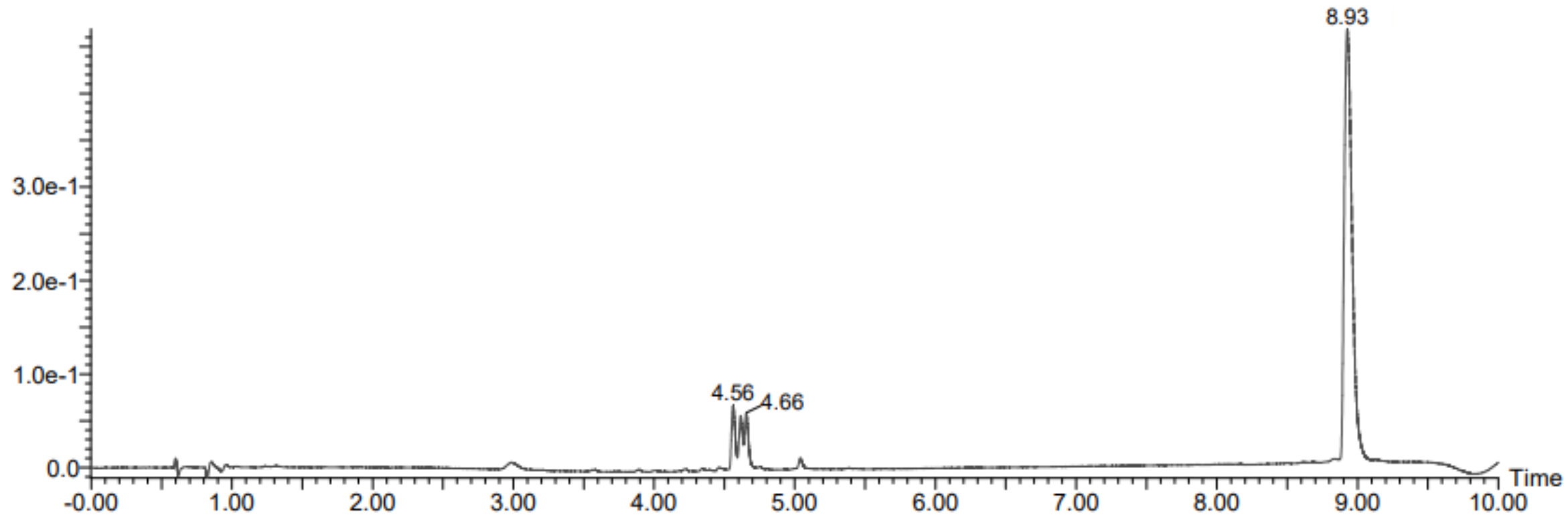

Mass spectrum (ES-) of **4g** (y-axis = relative intensity [%], x-axis = deconvolved mass [Da]); calculated mass: 3538.4, found: 3537.9.

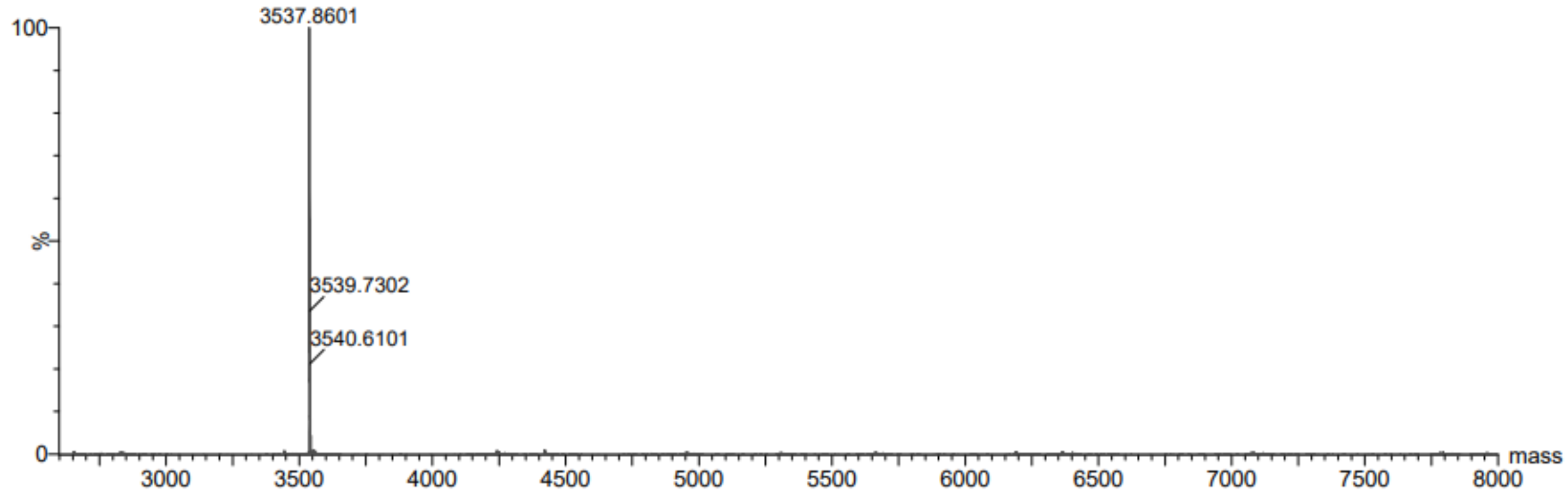

Entry 6 (4h)

Sequence	rt	Area (A)	Extinction coefficient (ε)	A/ε	Conversion
SSQVYSPRPR	4.072	9191.357	110270	0.083353197	
SSQVYSPRPR	4.133	4079.386	110270	0.036994523	
SSQVYSPRPR	4.371	1639.962	110270	0.014872241	
**R-**SSQVYSPRPR	8.208	65600.36	110270	0.594906675	81%

[R = $CH_3(CH_2)_{14}CH_2O$]

Ion-pair reversed-phase UHPLC chromatogram of the crude reaction mixture (UV absorbance at 260 nm vs. time in minutes).

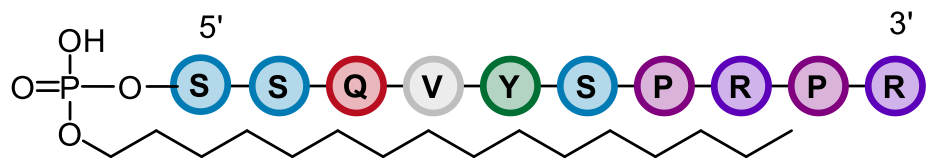

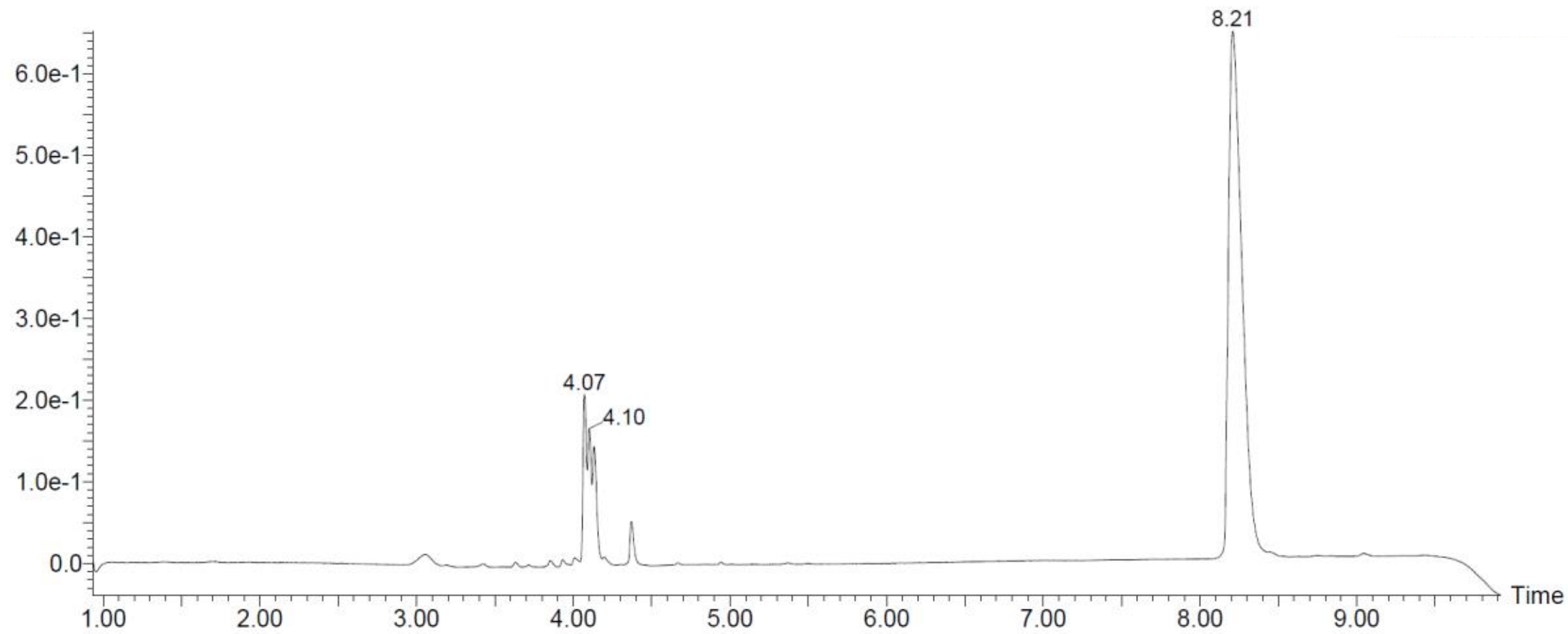

Mass spectrum (ES-) of **4h** (y-axis = relative intensity [%], x-axis = deconvolved mass [Da]); calculated mass: 3566.2, found: 3565.3.

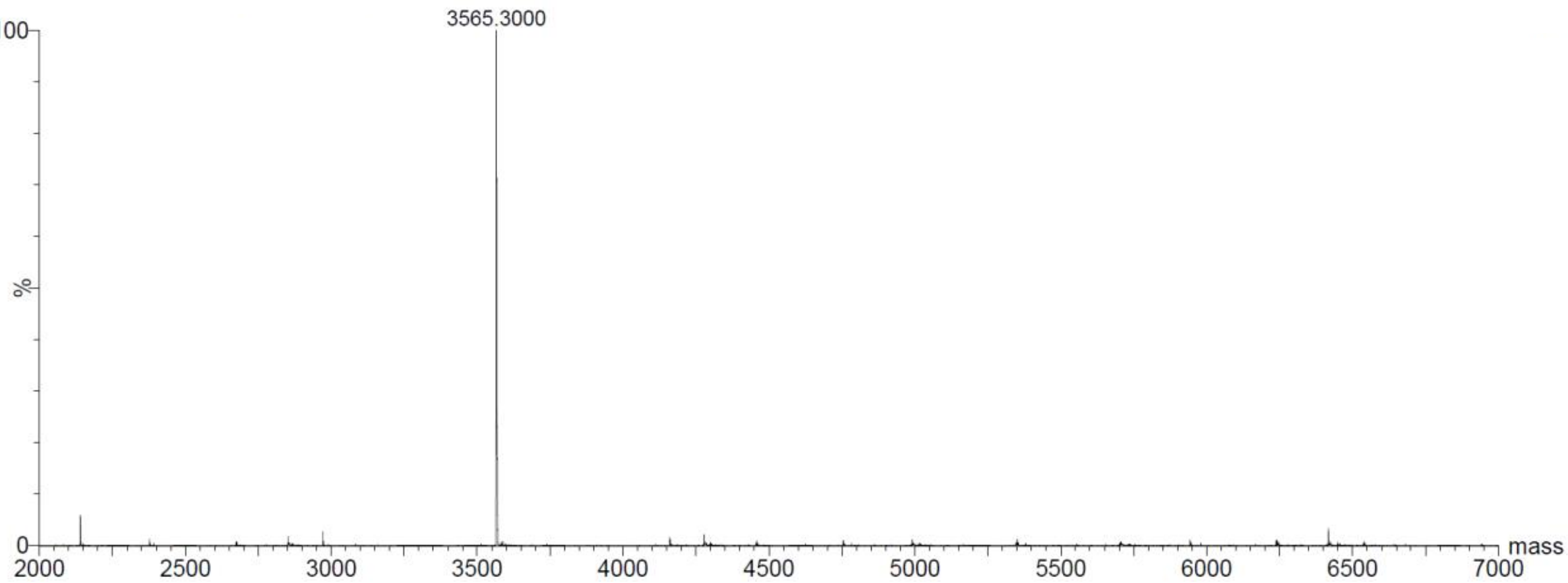

Entry 7 (4i)

Sequence	rt	Area (A)	Extinction coefficient (ε)	A/ε	Conversion
SSQVYSPRPR	4.138	4995.186	110270	0.04529959	
**R-**SSQVYSPRPR	8.153	35561.18	110270	0.32249188	88%

[R = $CH_3(CH_2)_7CH=CH(CH_2)_8O$]

Ion-pair reversed-phase UHPLC chromatogram of the crude reaction mixture (UV absorbance at 260 nm vs. time in minutes).

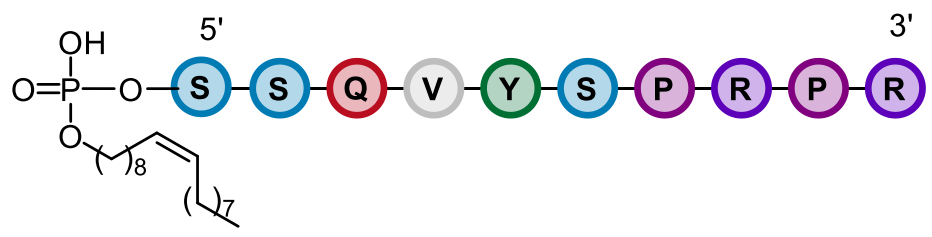

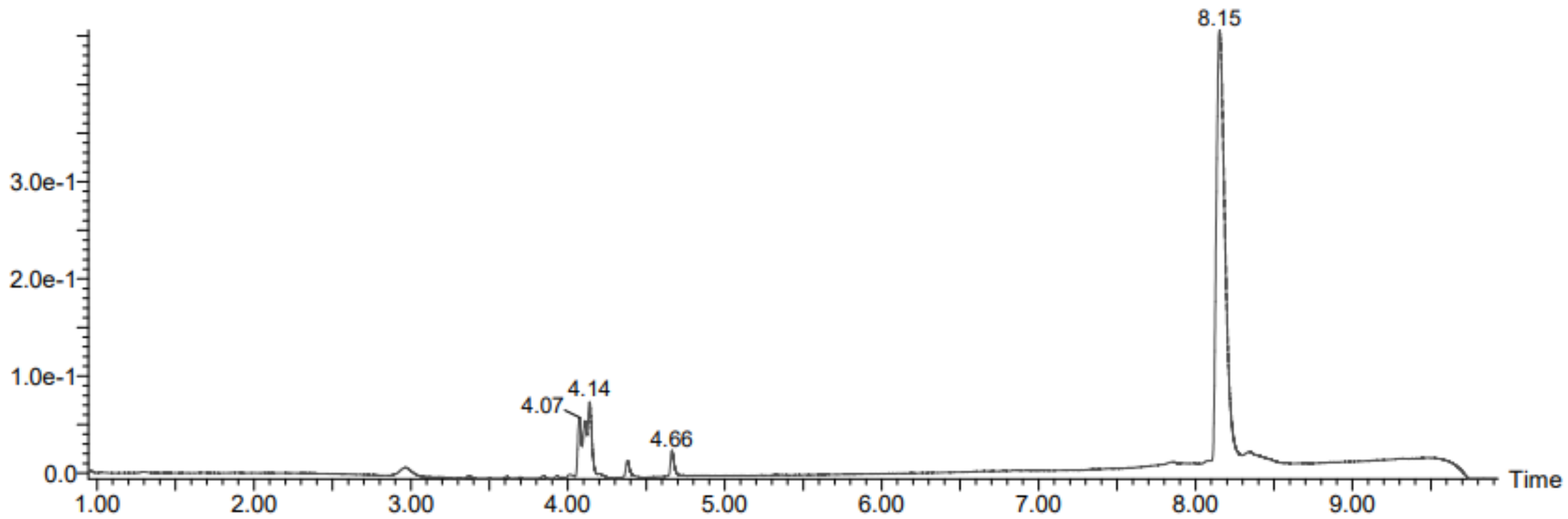

Mass spectrum (ES-) of **4i** (y-axis = relative intensity [%], x-axis = deconvolved mass [Da]); calculated mass: 3591.8, found: 3591.9.

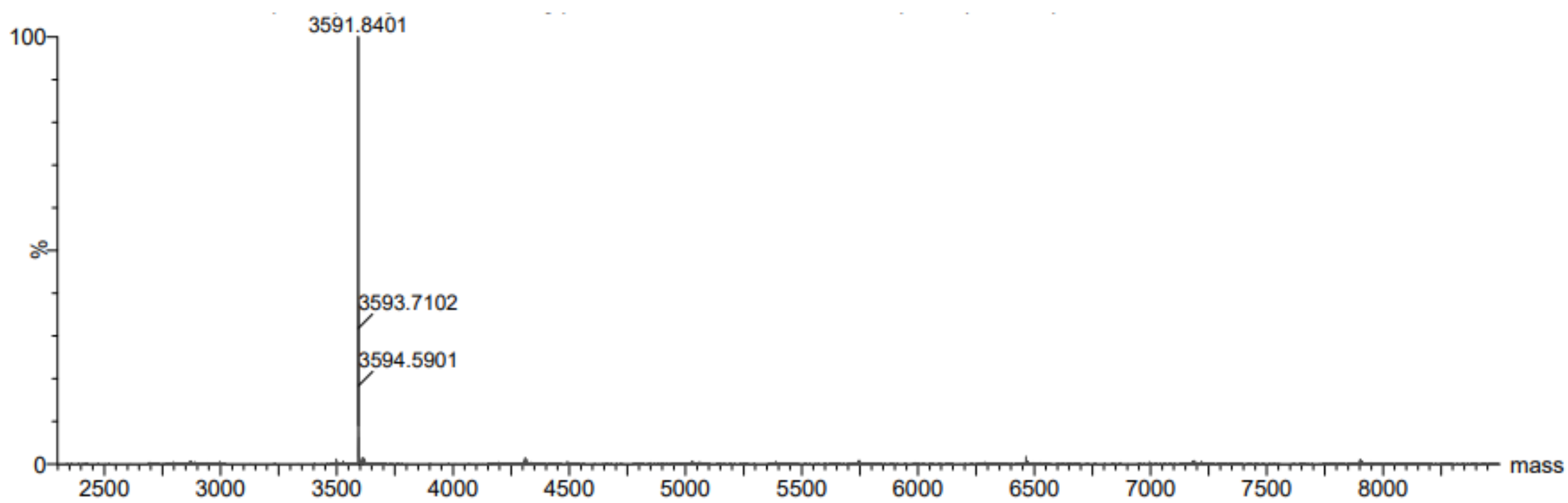

Entry 8 (4j)

Sequence	rt	Area (A)	Extinction coefficient (ε)	A/ε	Conversion
SSQVYSPRPR	4.732	1116.9	110270	0.0101288	
SSQVYSPRPR	4.779	892.494	110270	0.0080937	
R-SSQVYSPRPR	4.941	18028.783	110270	0.1634967	90%

[R = but-3-yn-1-ol]

Ion-pair reversed-phase UHPLC chromatogram of the crude reaction mixture (UV absorbance at 260 nm vs. time in minutes).

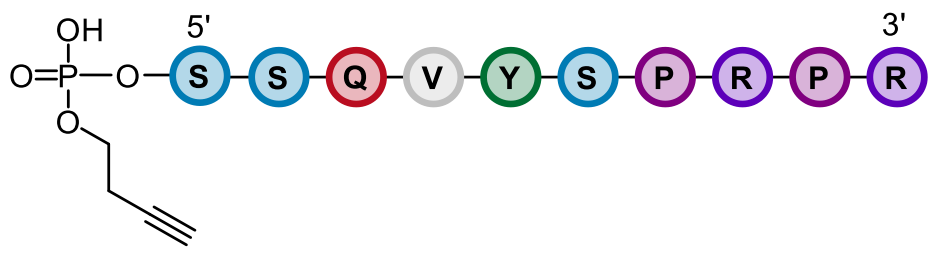

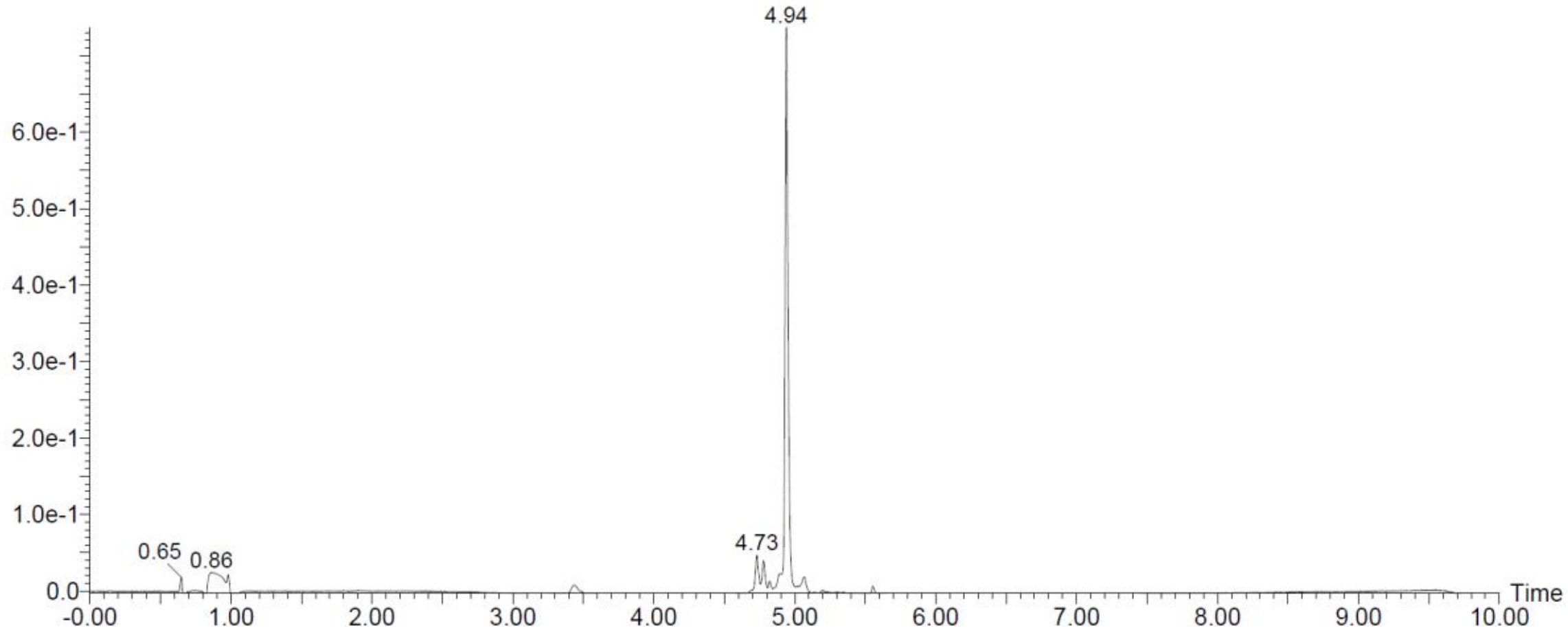

Mass spectrum (ES-) of **4j** (y-axis = relative intensity [%], x-axis = deconvolved mass [Da]); calculated mass: 3394.1, found: 3394.5.

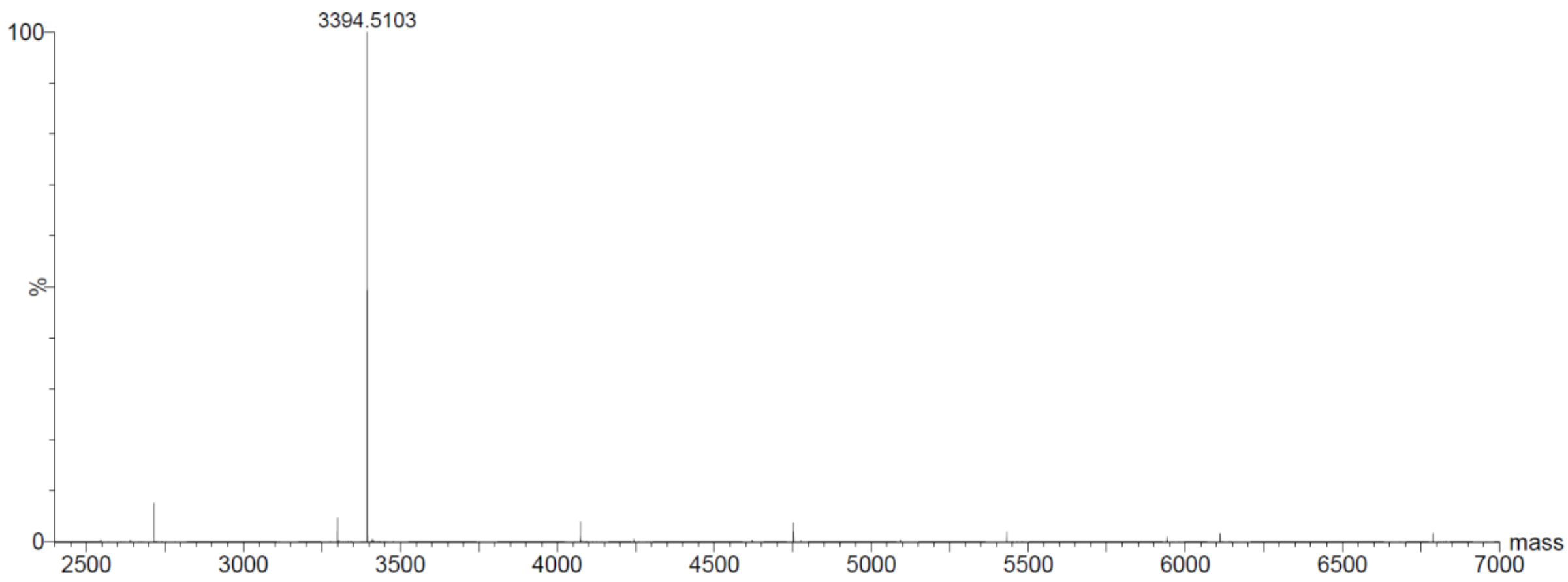

Entry 9 (4k)

Sequence	rt	Area (A)	Extinction coefficient (ε)	A/ε	Conversion
SSQVYSPRPR	4.737	1540.903	110270	0.0139739	
SSQVYSPRPR	4.783	1183.985	110270	0.0107371	
R-SSQVYSPRPR	5.047	32033.361	110270	0.2904993	92%

[R = pent-4-yn-1-ol]

Ion-pair reversed-phase UHPLC chromatogram of the crude reaction mixture (UV absorbance at 260 nm vs. time in minutes).

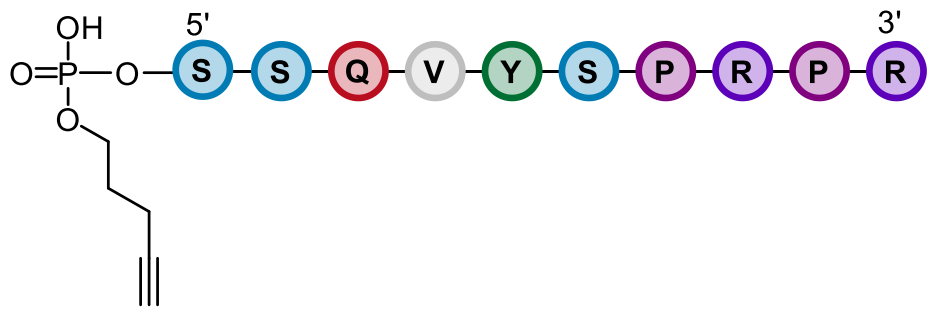

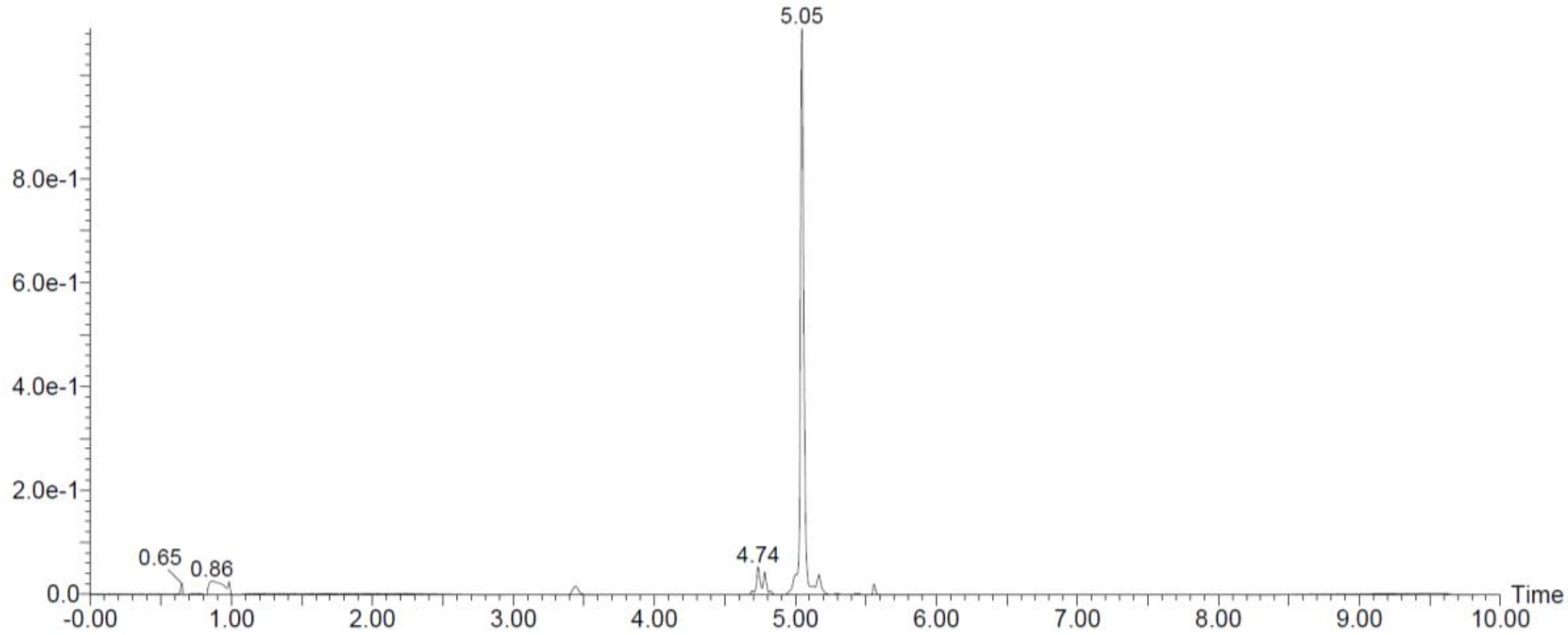

Mass spectrum (ES-) of **4k** (y-axis = relative intensity [%], x-axis = deconvolved mass [Da]); calculated mass: 3408.1, found: 3407.7.

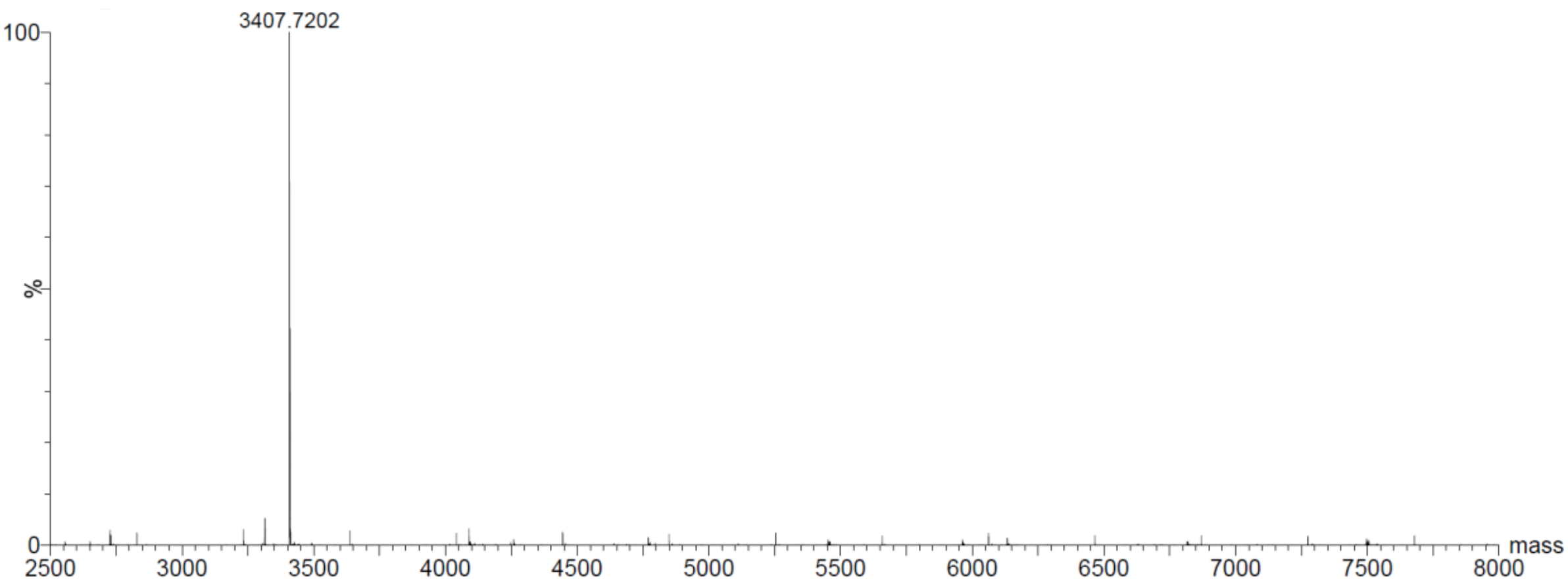

Entry 10 (4l)

Sequence	rt	Area (A)	Extinction coefficient (ε)	A/ε	Conversion
SSQVYSPRPR	4.722	502.15	110270	0.0045538	
SSQVYSPRPR	4.771	340.943	110270	0.0030919	
R-SSQVYSPRPR	5.176	12004.285	110270	0.1088627	93%

[R = hex-5-yn-1-ol]

Ion-pair reversed-phase UHPLC chromatogram of the crude reaction mixture (UV absorbance at 260 nm vs. time in minutes).

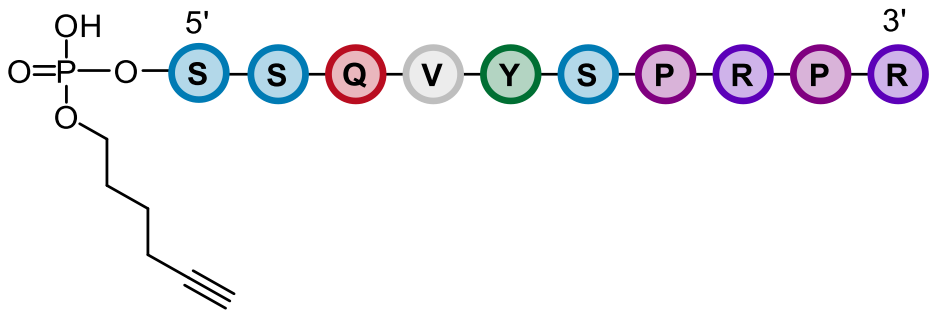

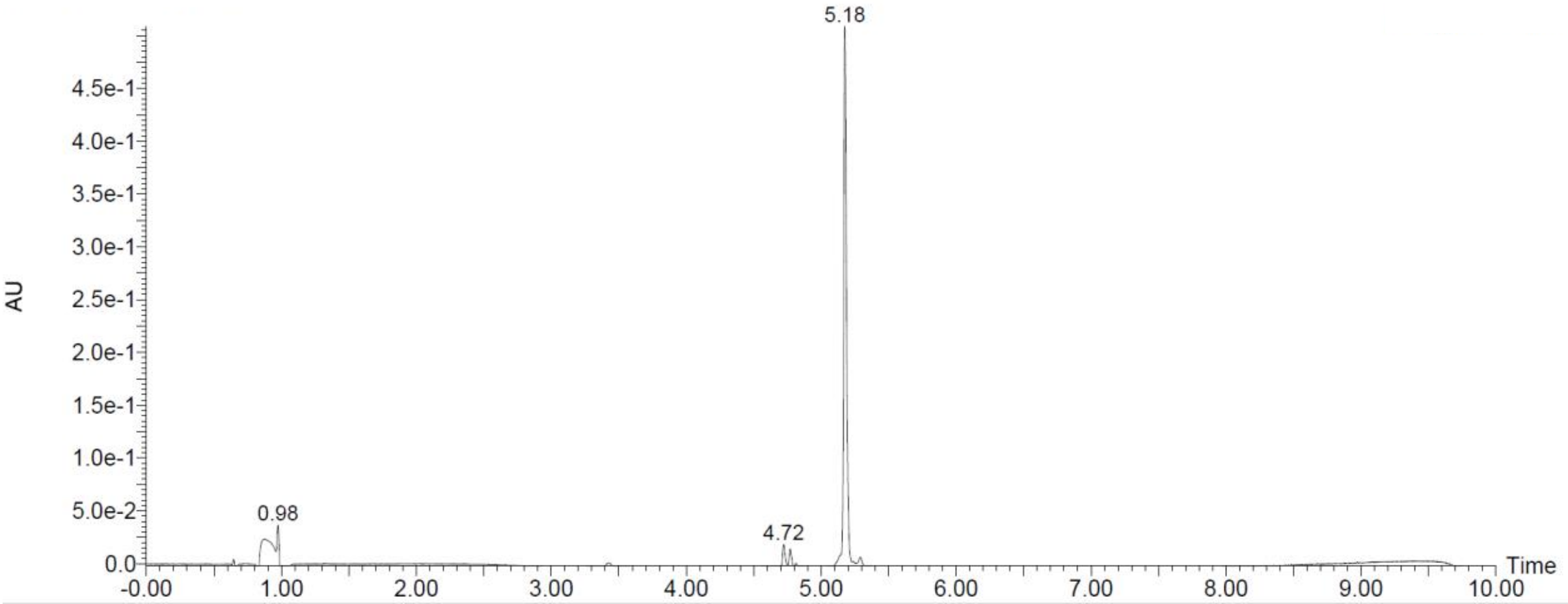

Mass spectrum (ES-) of **4l** (y-axis = relative intensity [%], x-axis = deconvolved mass [Da]); calculated mass: 3422.1, found: 3421.7.

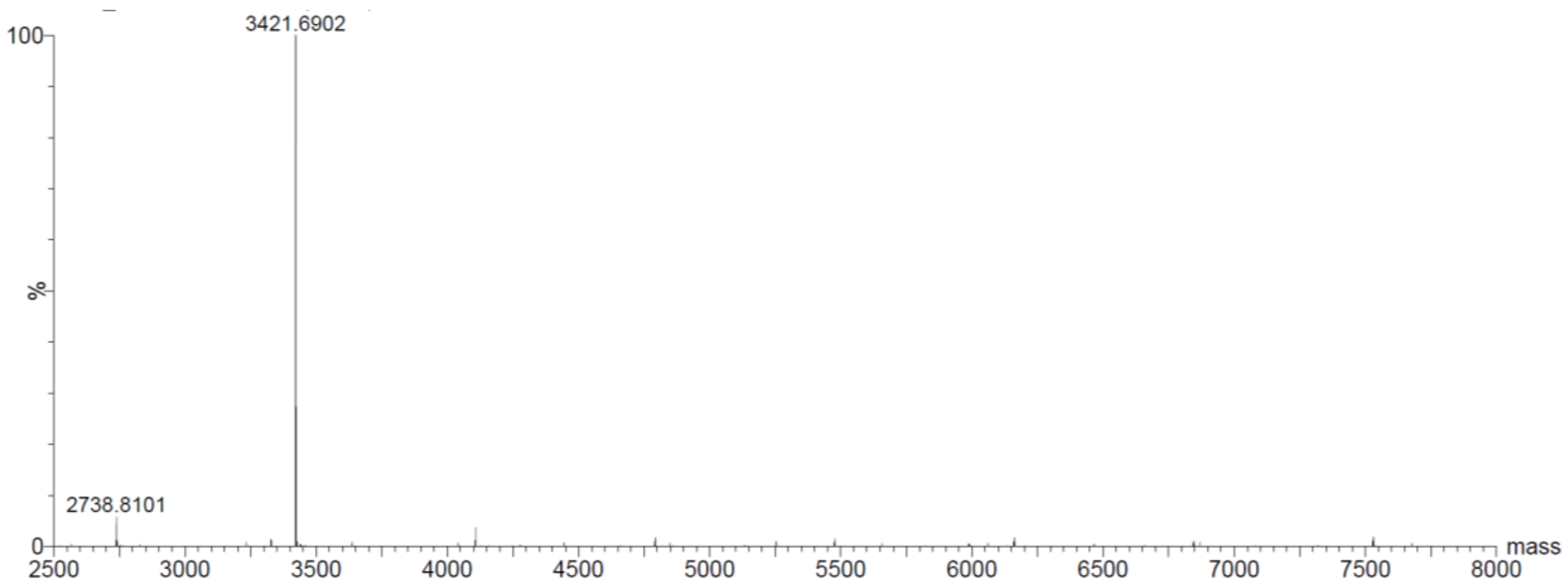

Entry 11 (4m)

Sequence	rt	Area (A)	Extinction coefficient (ε)	A/ε	Conversion
SSQVYSPRPR	4.73	1093.916	110270	0.031919	
**R-**SSQVYSPRPR	5.288	16658.859	110270	0.388182	94%

[R = pent-4-en-1-ol]

Ion-pair reversed-phase UHPLC chromatogram of the crude reaction mixture (UV absorbance at 260 nm vs. time in minutes).

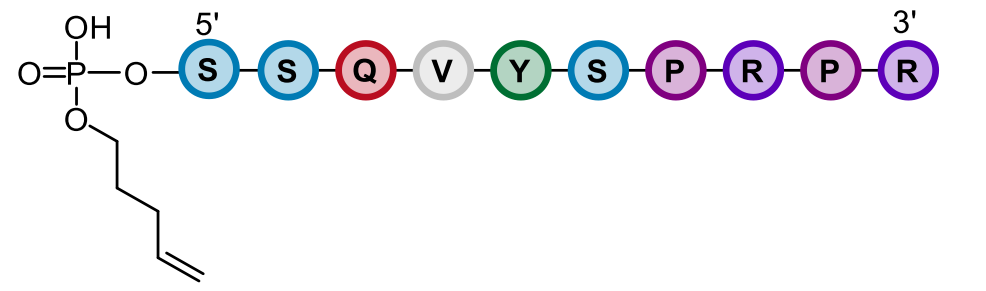

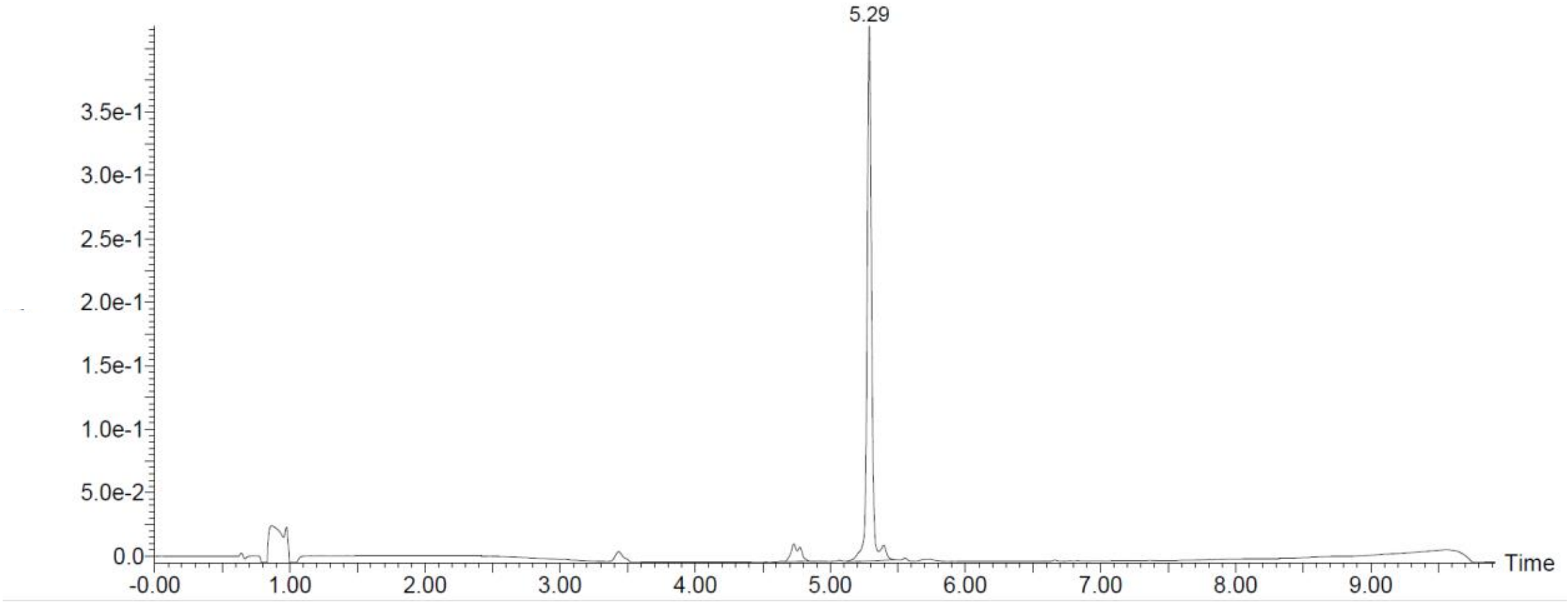

Mass spectrum (ES-) of **4m** (y-axis = relative intensity [%], x-axis = deconvolved mass [Da]); calculated mass: 3410.1, found: 3409.5.

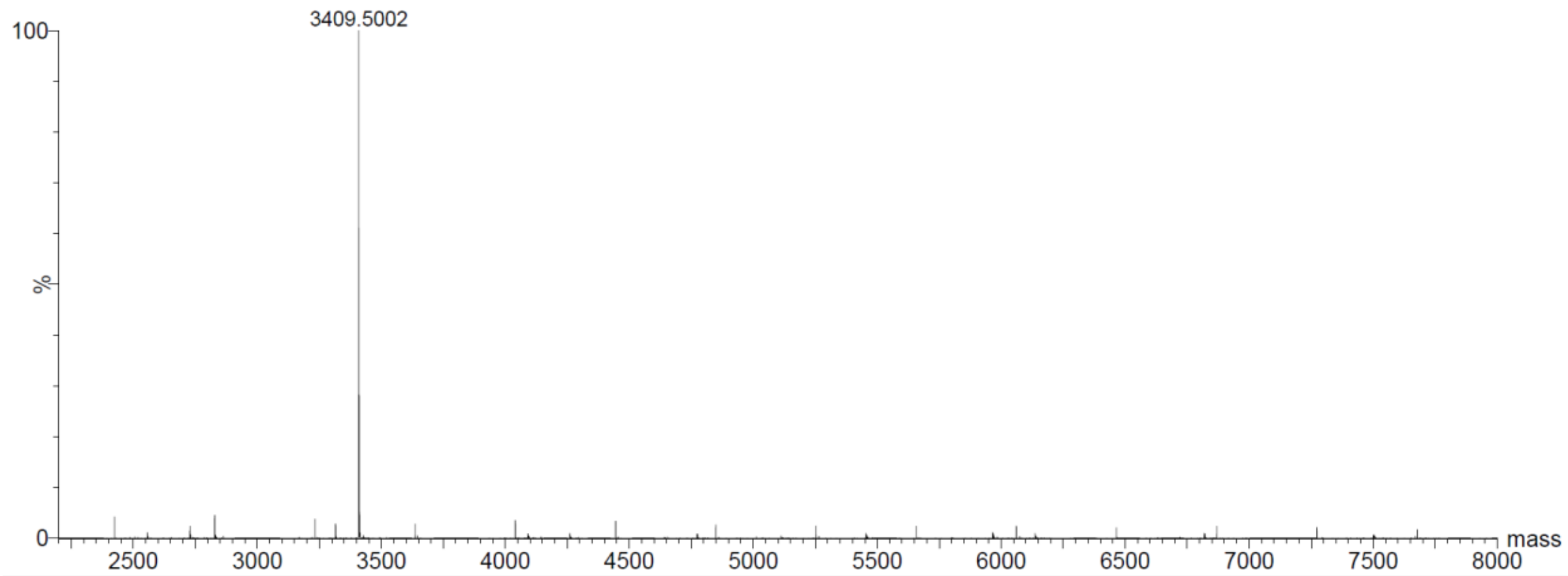

Entry 12 (4n)

Sequence	rt	Area (A)	Extinction coefficient (ε)	A/ε	Conversion
TTTTTTTTTT	4.291	795.418	81600	0.00974777	
TTTTTTTTTT	4.337	1513.403	81600	0.01854661	
TTTTTTTTTT	4.374	703.766	81600	0.00862458	
TTTTTTTTTT	4.469	771.724	81600	0.0094574	
R-TTTTTTTTTT	6.456	27182.768	81600	0.33312216	88%

[R = $H_2C=CH(CH_2)_7CH_2O$]

Ion-pair reversed-phase UHPLC chromatogram of the crude reaction mixture (UV absorbance at 260 nm vs. time in minutes).

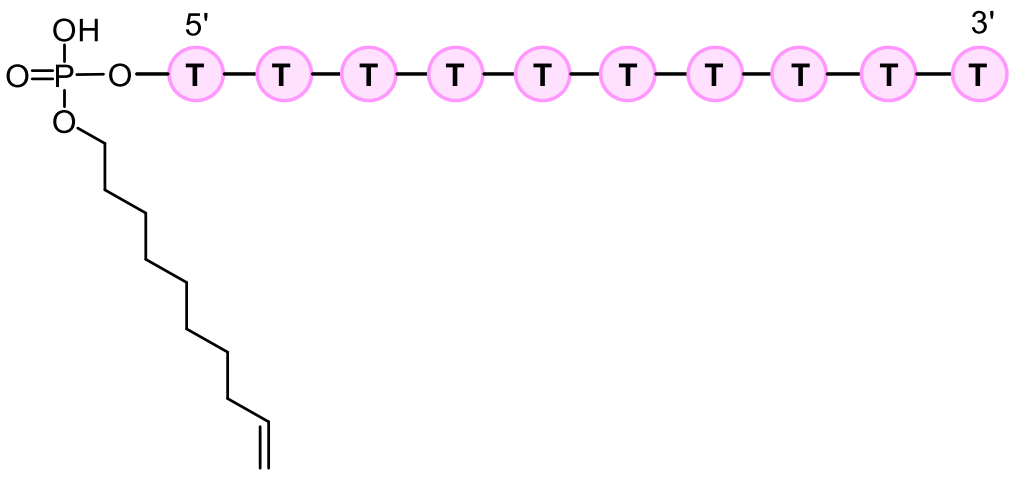

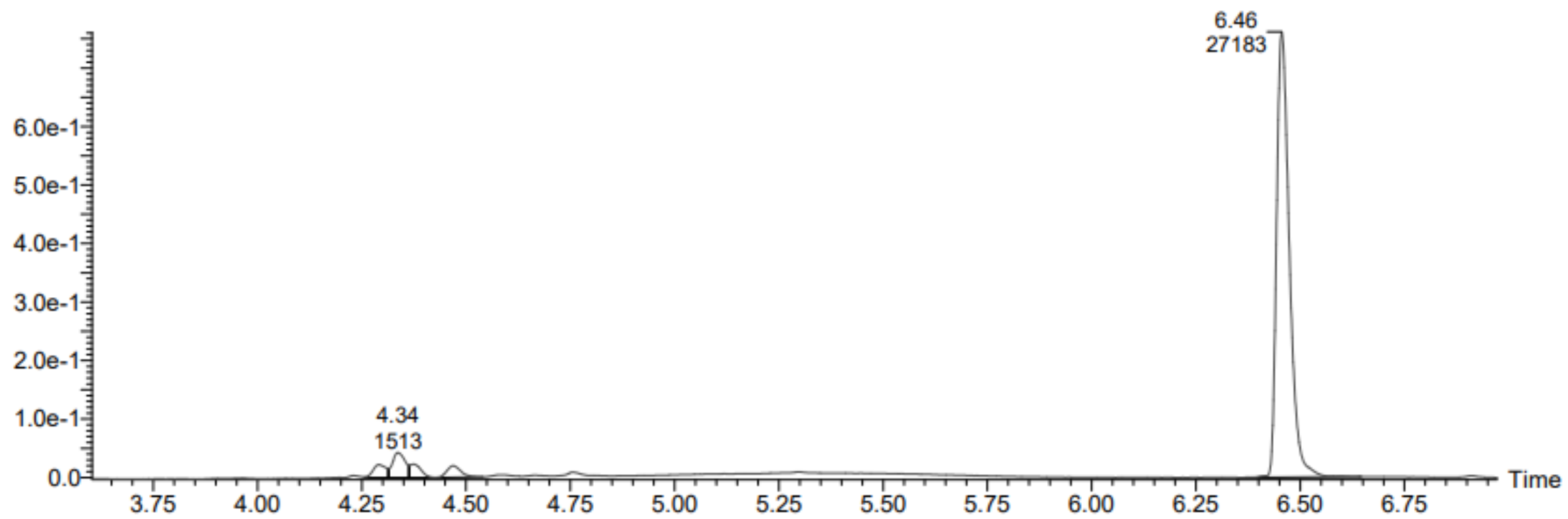

Mass spectrum (ES-) of **4n** (y-axis = relative intensity [%], x-axis = deconvolved mass [Da]); calculated mass: 3198.2, found: 3197.8.

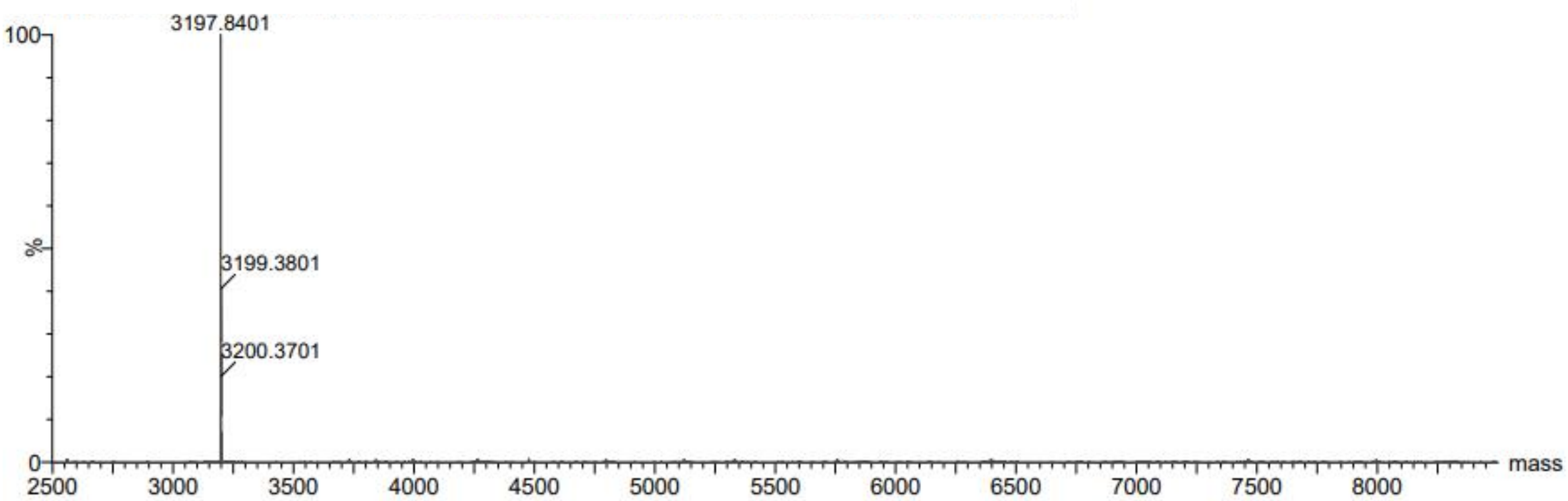

Entry 13 (4o)

Sequence	rt	Area (A)	Extinction coefficient (ε)	A/ ε	Conversion
SSQVYSPRPR	4.752	5625.413	110270	0.0510149	
R-SSQVYSPRPR	5.215	15747.031	110270	0.1428043	74%

[R = 2-(2-(2-azidoethoxy)ethoxy) ethan-1-ol]

Ion-pair reversed-phase UHPLC chromatogram of the crude reaction mixture (UV absorbance at 260 nm vs. time in minutes).

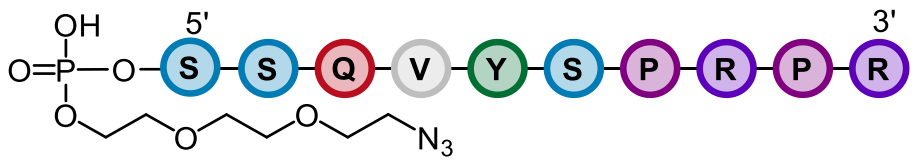

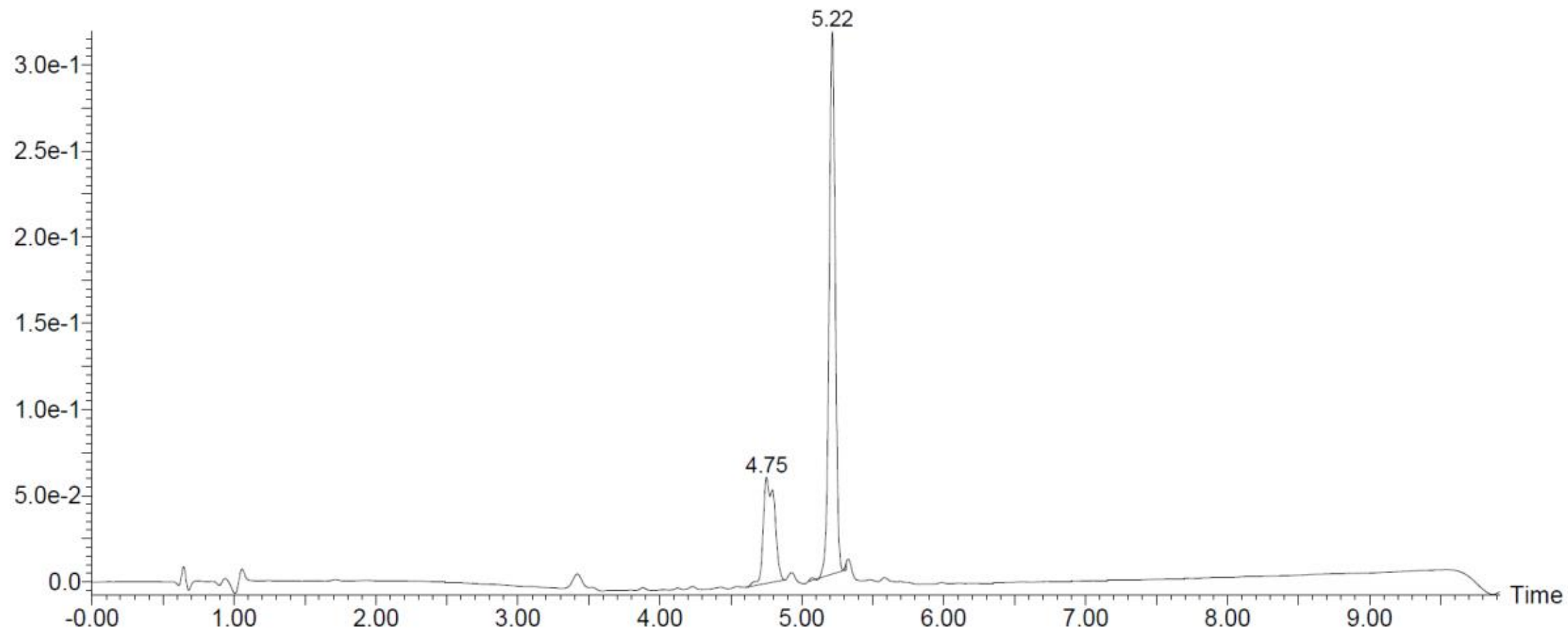

Mass spectrum (ES-) of **4o** (y-axis = relative intensity [%], x-axis = deconvolved mass [Da]); calculated mass: 3499.2, found: 3499.7.

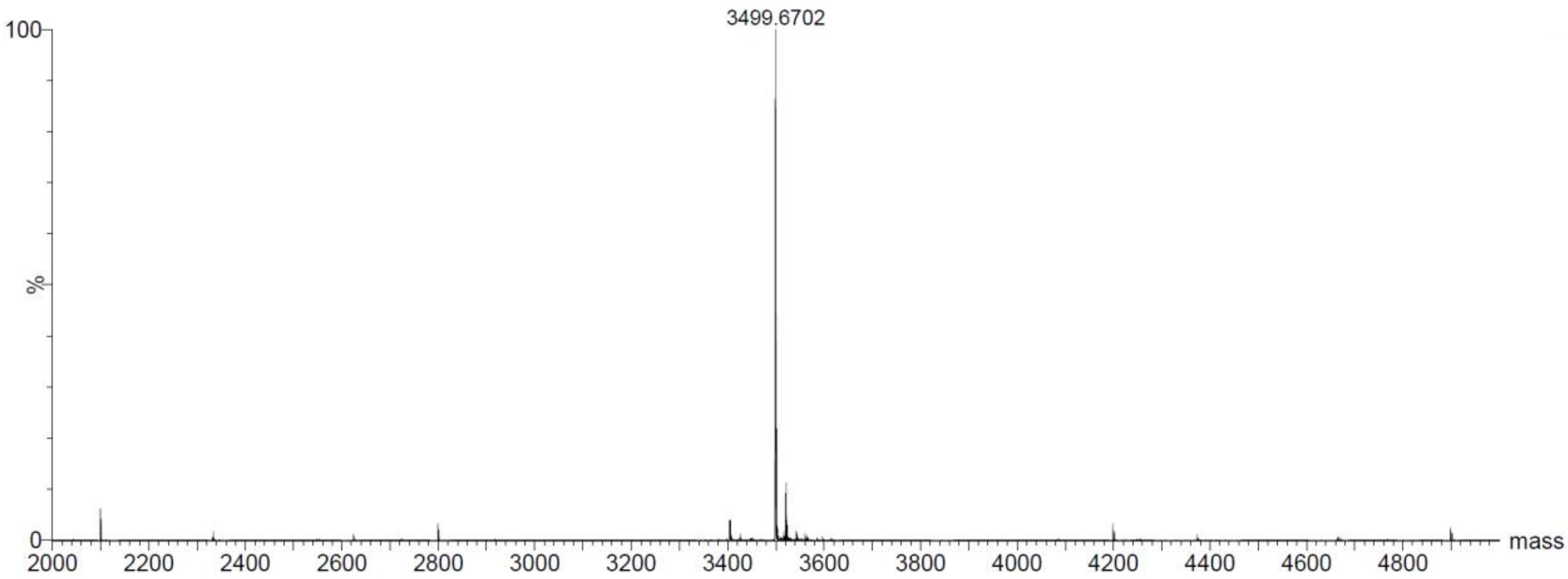

Entry 14 (4p)

Sequence	rt	Area (A)	Extinction coefficient (ε)	A/ε	Conversion
SSQVYSPRPR	4.614	4016.517	110270	0.036424386	
SSQVYSPRPR	4.699	8705.492	110270	0.078947057	
R-SSQVYSPRPR	5.486	30706.936	110270	0.278470445	71%

[R = 3-(dimethylamino)propan-1-ol]

Ion-pair reversed-phase UHPLC chromatogram of the crude reaction mixture (UV absorbance at 260 nm vs. time in minutes).

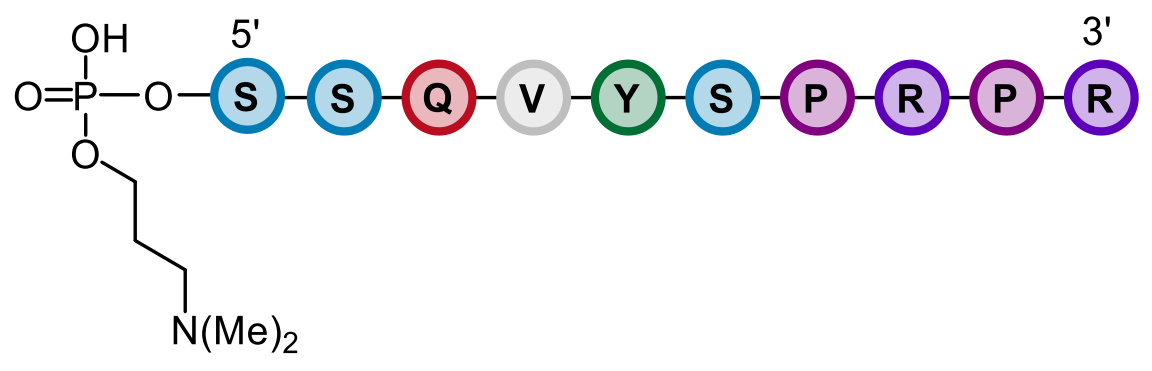

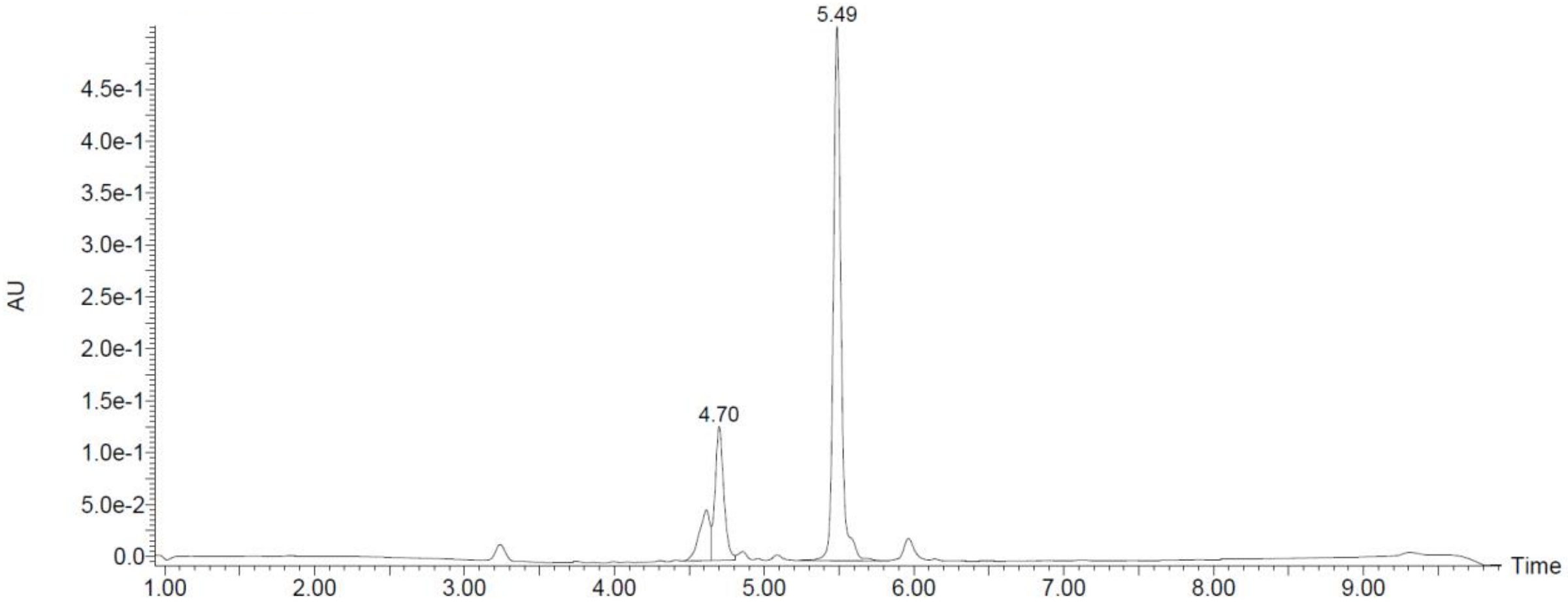

Mass spectrum (ES-) of **4p** (y-axis = relative intensity [%], x-axis = deconvolved mass [Da]); calculated mass: 3425.2, found: 3425.7.

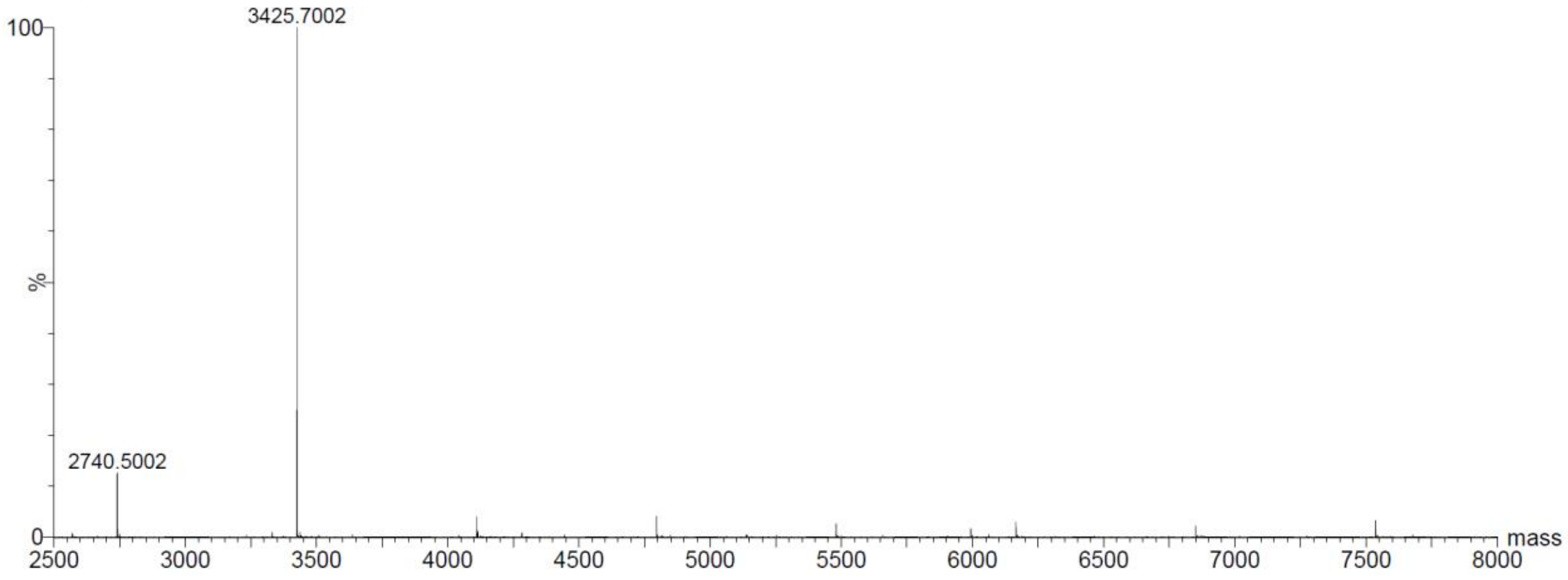

Entry 15 (4q)

Sequence	rt	Area (A)	Extinction coefficient (ε)	A/ε	Conversion
R-SSQVYSPRPR	4.88	29390.262	110270	0.26653	90%
SSQVYSPRPR	5.682	3250.866	110270	0.029481	

[R = 6-aminohexan-1-ol]

Ion-pair reversed-phase UHPLC chromatogram of the crude reaction mixture (UV absorbance at 260 nm vs. time in minutes).

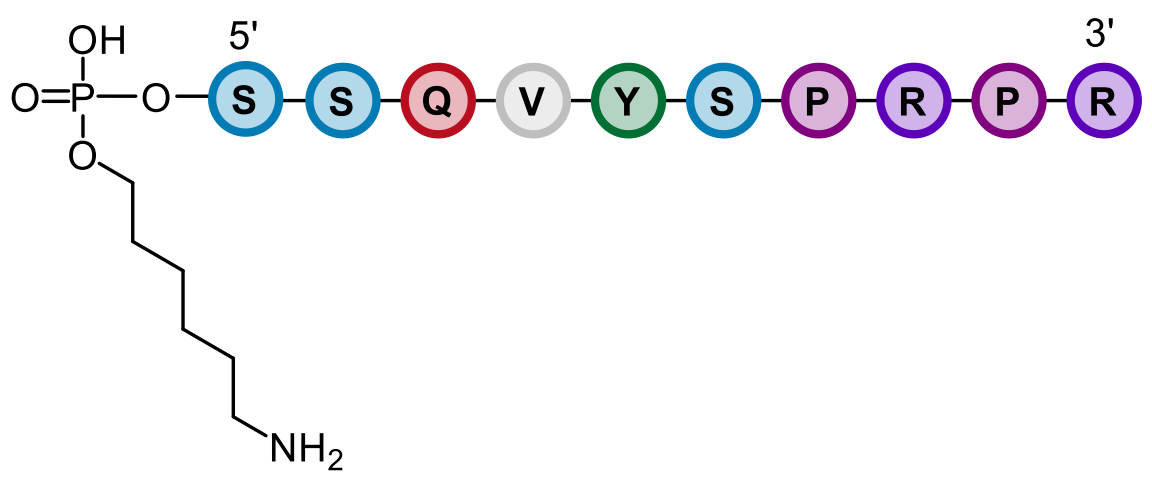

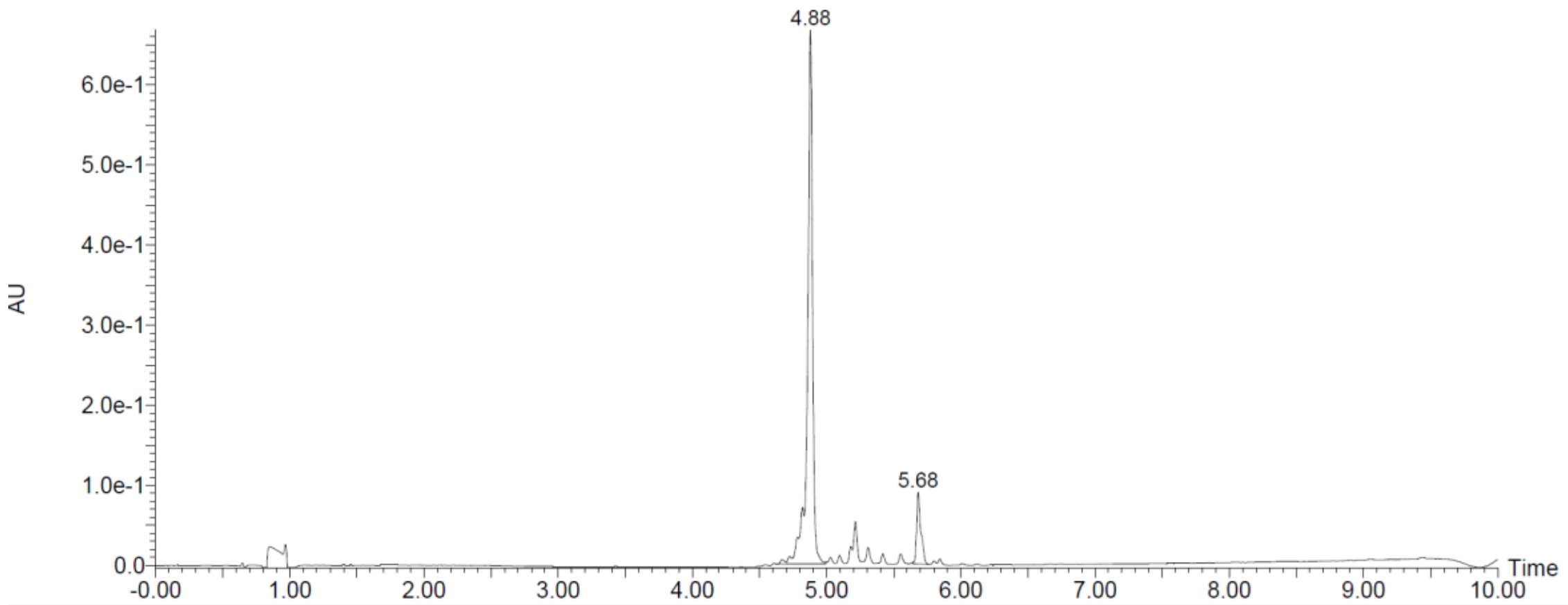

Mass spectrum (ES-) of **4q** (y-axis = relative intensity [%], x-axis = deconvolved mass [Da]); calculated mass: 3441.2, found: 3441.8.

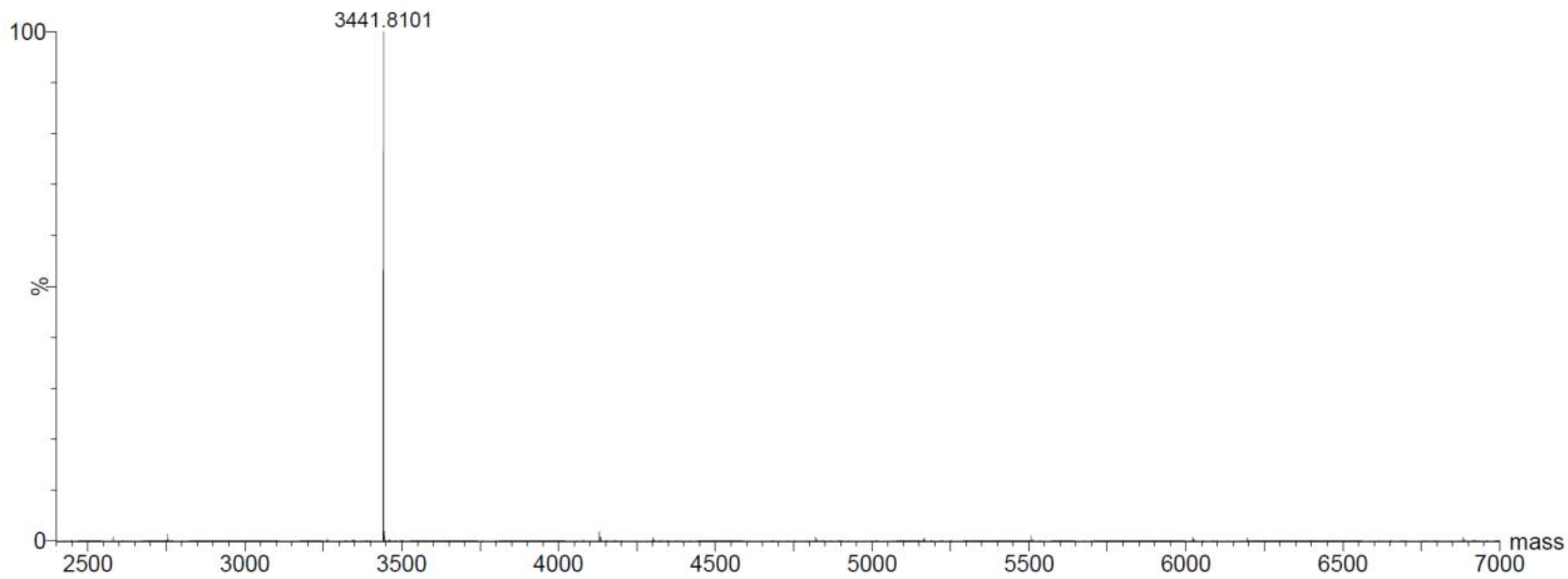

Entry 16 (4r)

Sequence	rt	Area (A)	Extinction coefficient (ε)	A/ε	Conversion
SSQVYSPRPR	4.805	1096133.875	110270	9.940454113	
R-SSQVYSPRPR	11.65	757377.438	110270	6.868390659	41%

Ion-pair reversed-phase UHPLC chromatogram of the crude reaction mixture (UV absorbance at 260 nm vs. time in minutes). [R = cyanine-3]

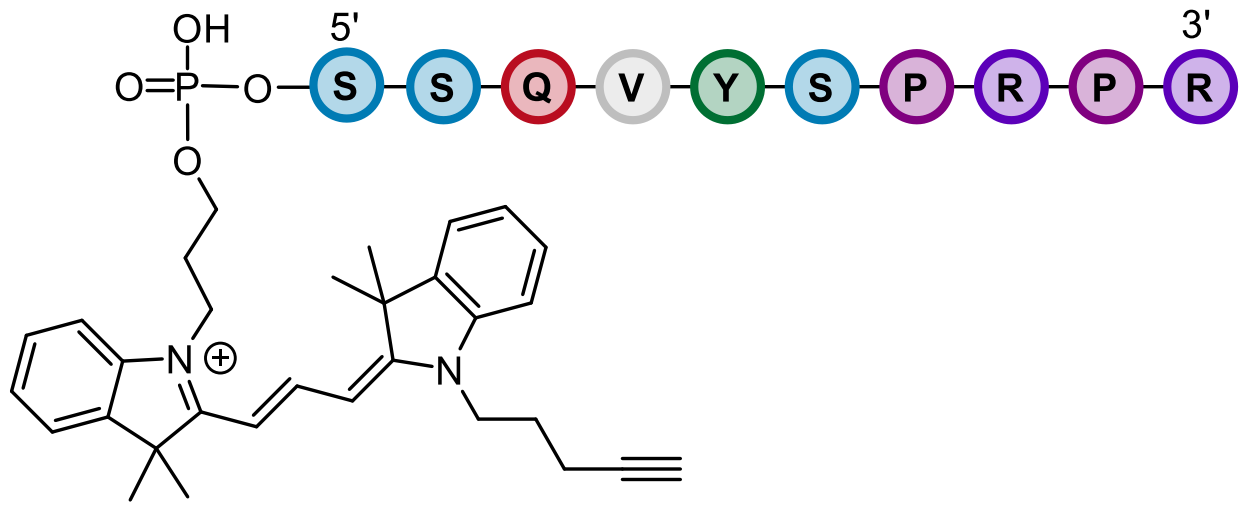

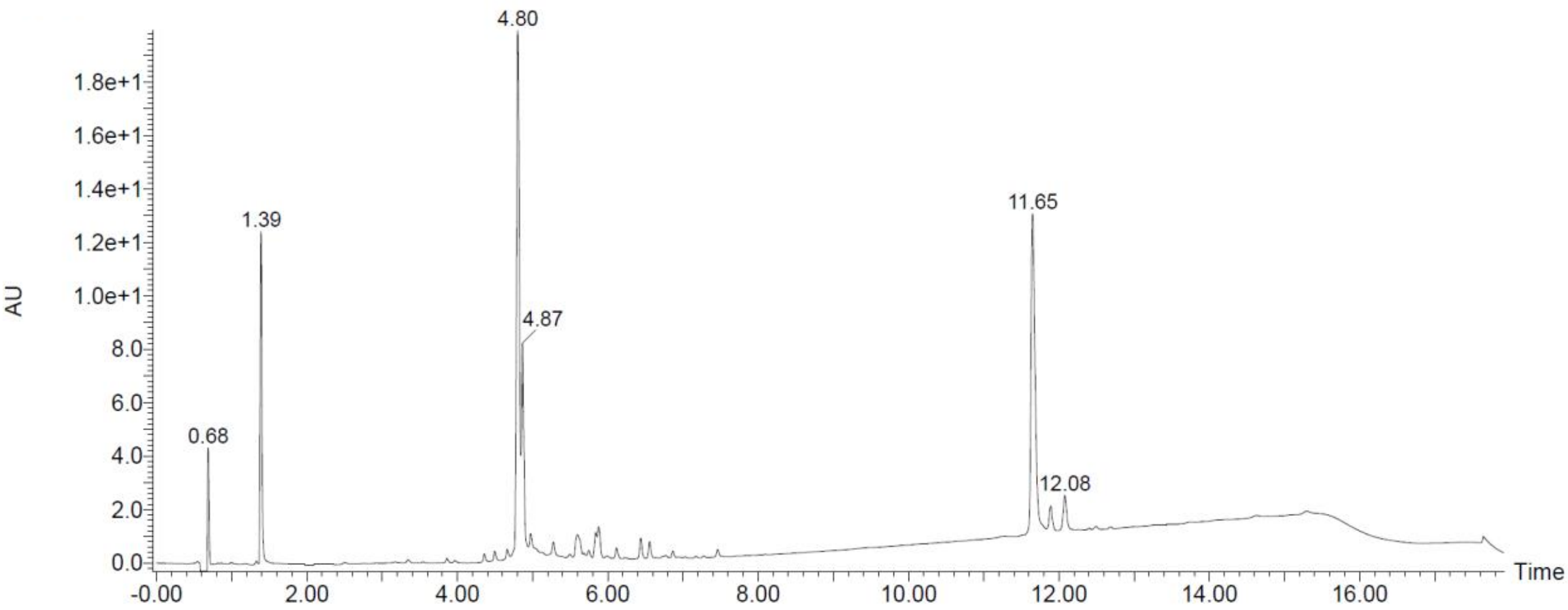

Mass spectrum (ES-) of **4r** (y-axis = relative intensity [%], x-axis = deconvolved mass [Da]); calculated mass: 3775.7, found: 3776.8.

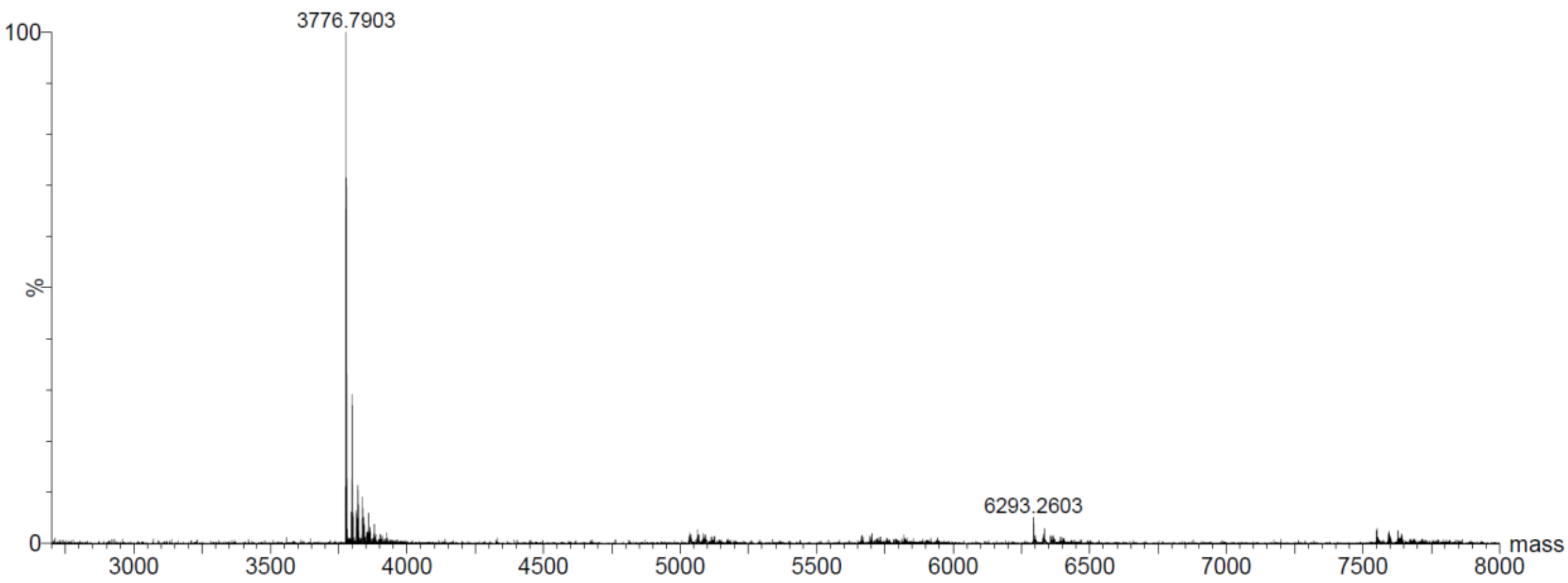

Entry 17 (4s)

Ion-pair reversed-phase UHPLC chromatogram of the crude reaction mixture (UV absorbance at 260 nm vs. time in minutes). [R = 6-mercaptohexan-1-ol]

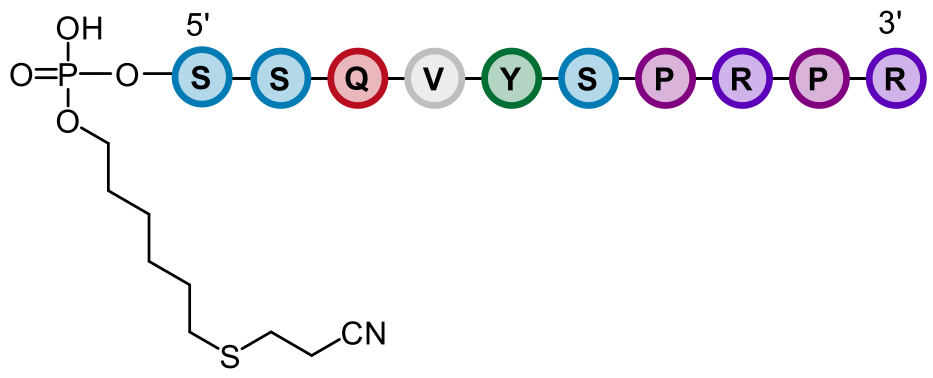

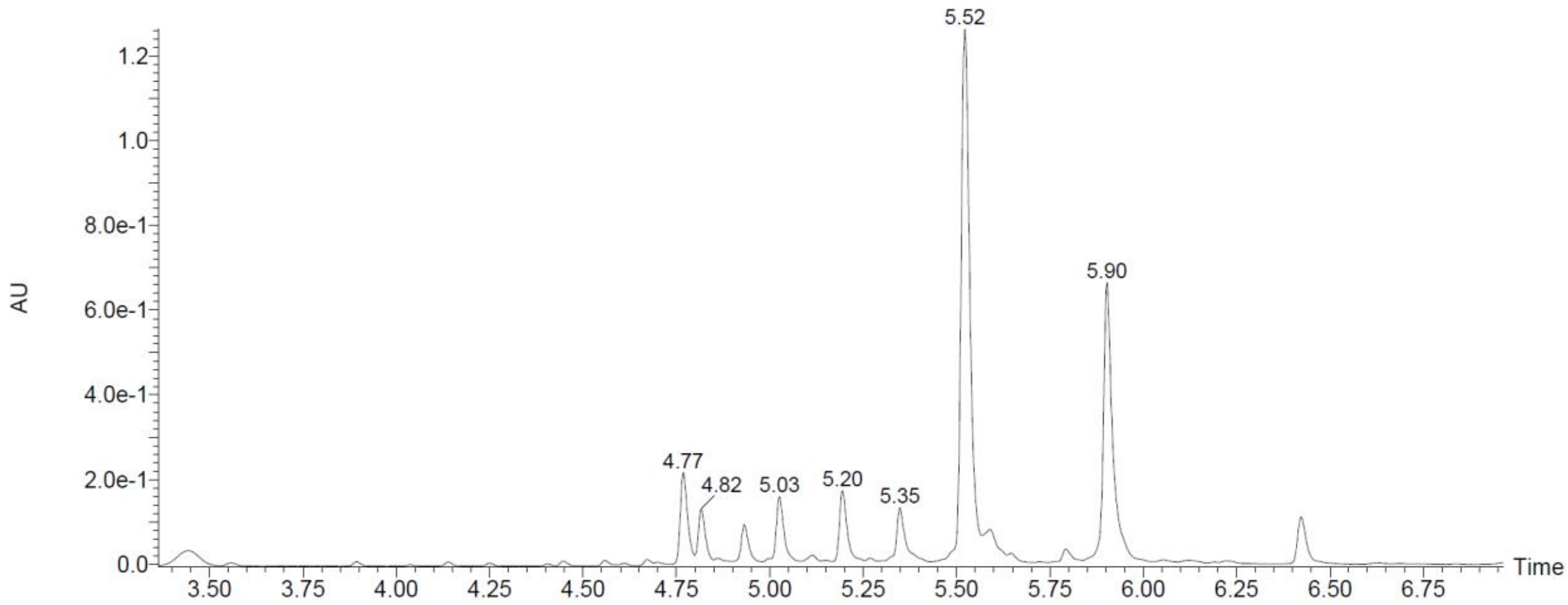

Mass spectrum (ES-) of **4s** (y-axis = relative intensity [%], x-axis = deconvolved mass [Da]); calculated mass: 3511.3 found 3511.7

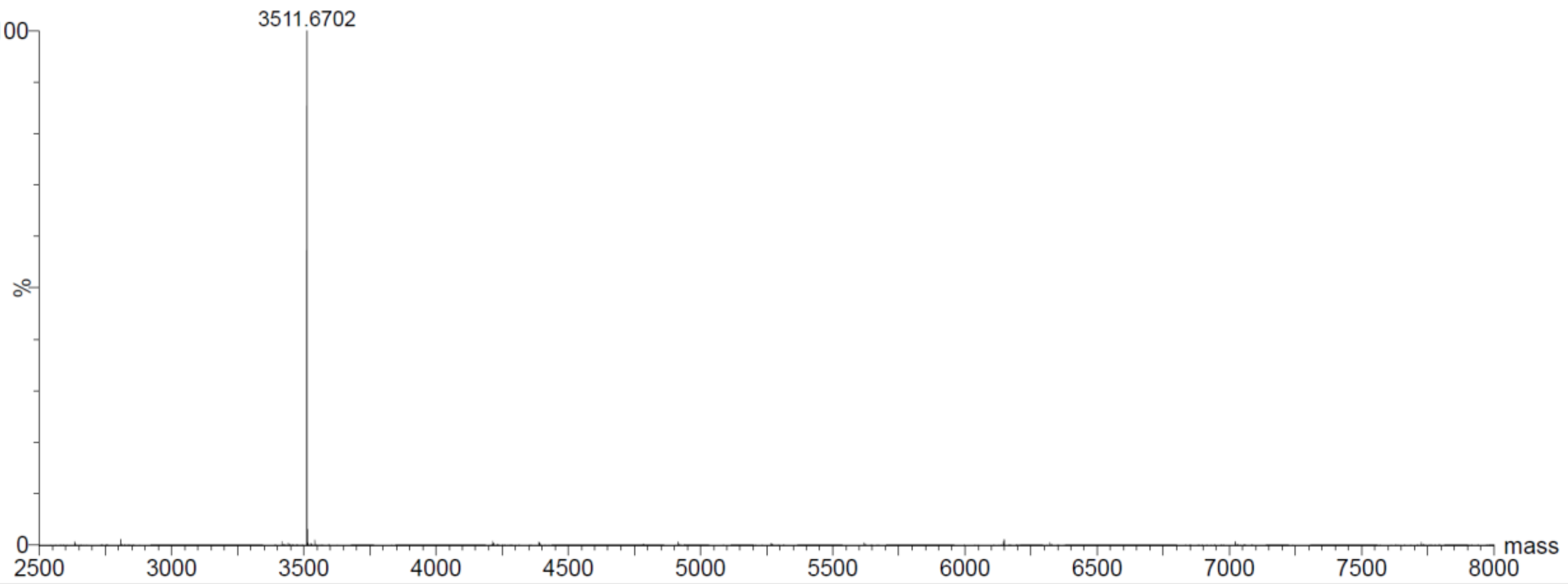

Mass spectrum (ES-) of **4s**-dimer (y-axis = relative intensity [%], x-axis = deconvolved mass [Da]); calculated mass: dimeric (S–S) 6914.4 found 6913.2.

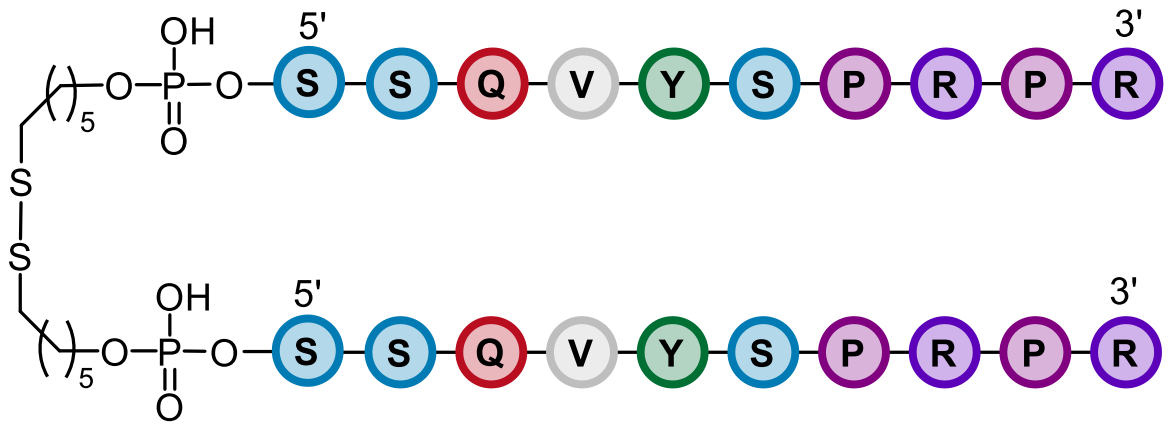

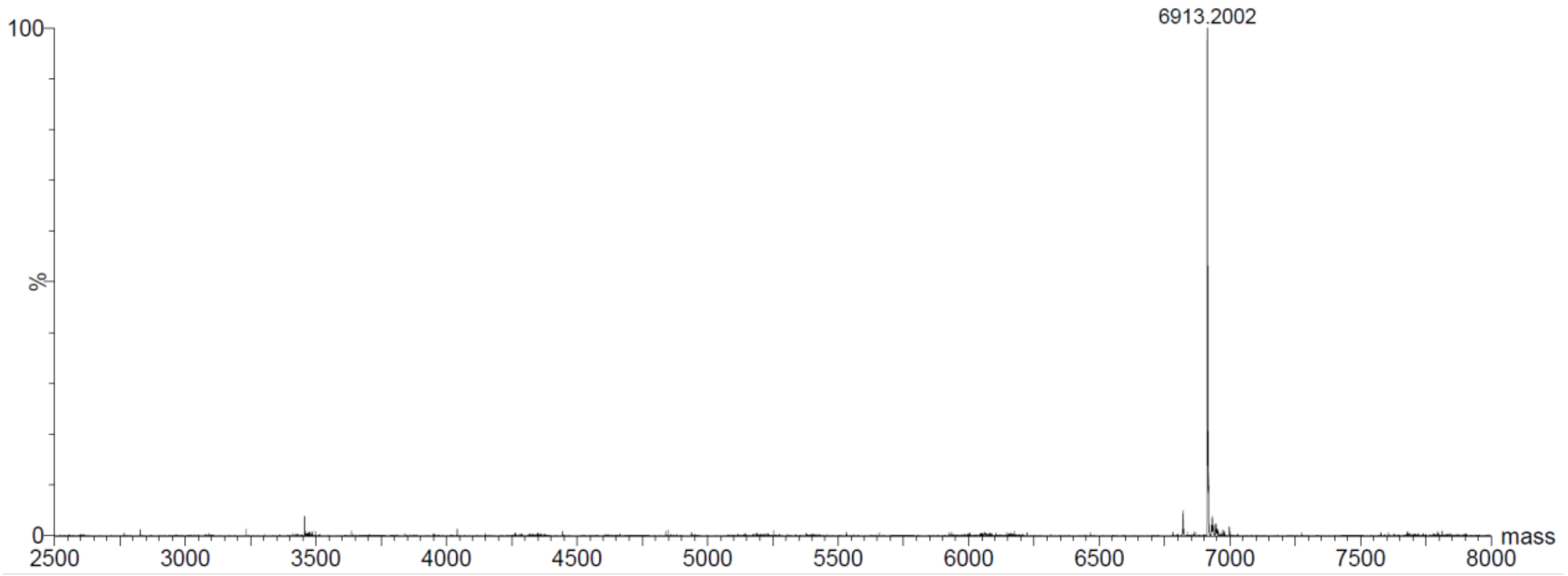

Entry 18

Sequence	rt	Area (A)	Extinction coefficient (ε)	A/ε	Conversion
SSQVYSPRPR	4.687	2172.367	110270	0.01970043	
SSQVYSPRPR	4.803	4595.325	110270	0.04167339	
(**R-**SSQVYSPRPR)$_2$	5.968	20629.36	220540	0.18708043	25%
**R-**SSQVYSPRPR	6.545	23591.318	110270	0.21394139	58%

[R = 6, 6′-disulfanediylbis(hexan-1-ol)]

Ion-pair reversed-phase UHPLC chromatogram of the crude reaction mixture (UV absorbance at 260 nm vs. time in minutes).

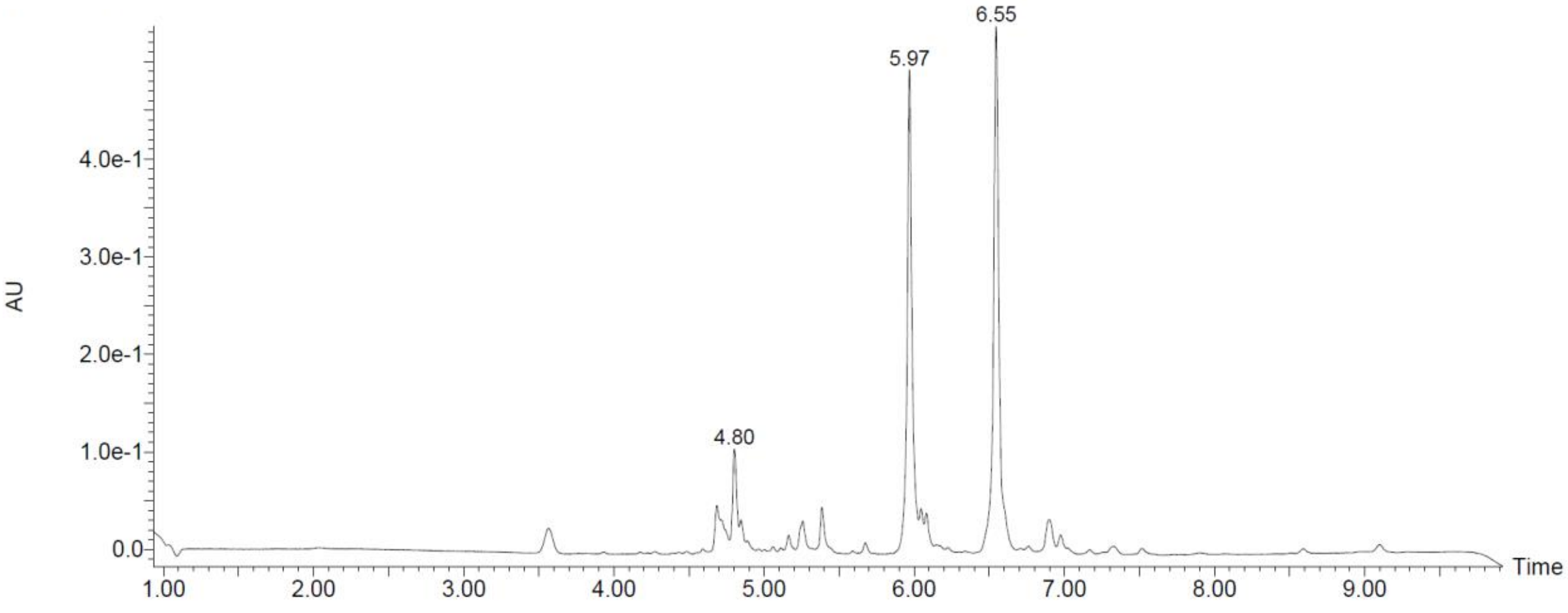

Mass spectrum (ES-) of **4t-**dimer (y-axis = relative intensity [%], x-axis = deconvolved mass [Da]); calculated mass: 6914.4, found: 6912.8.

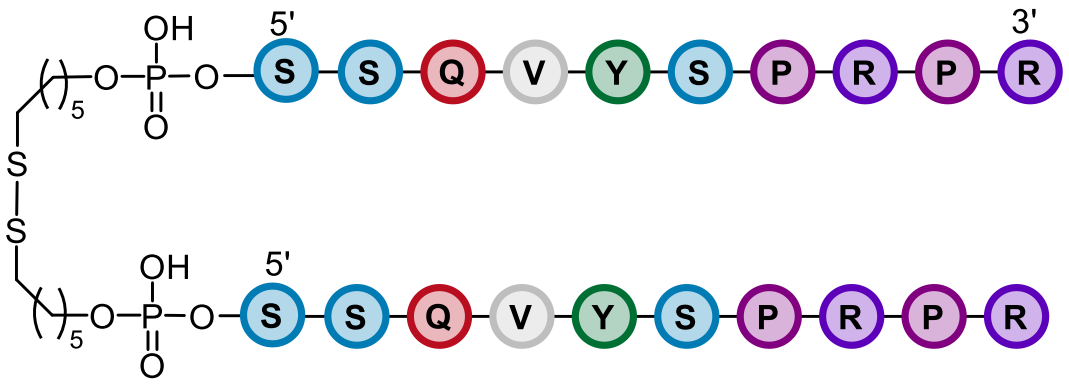

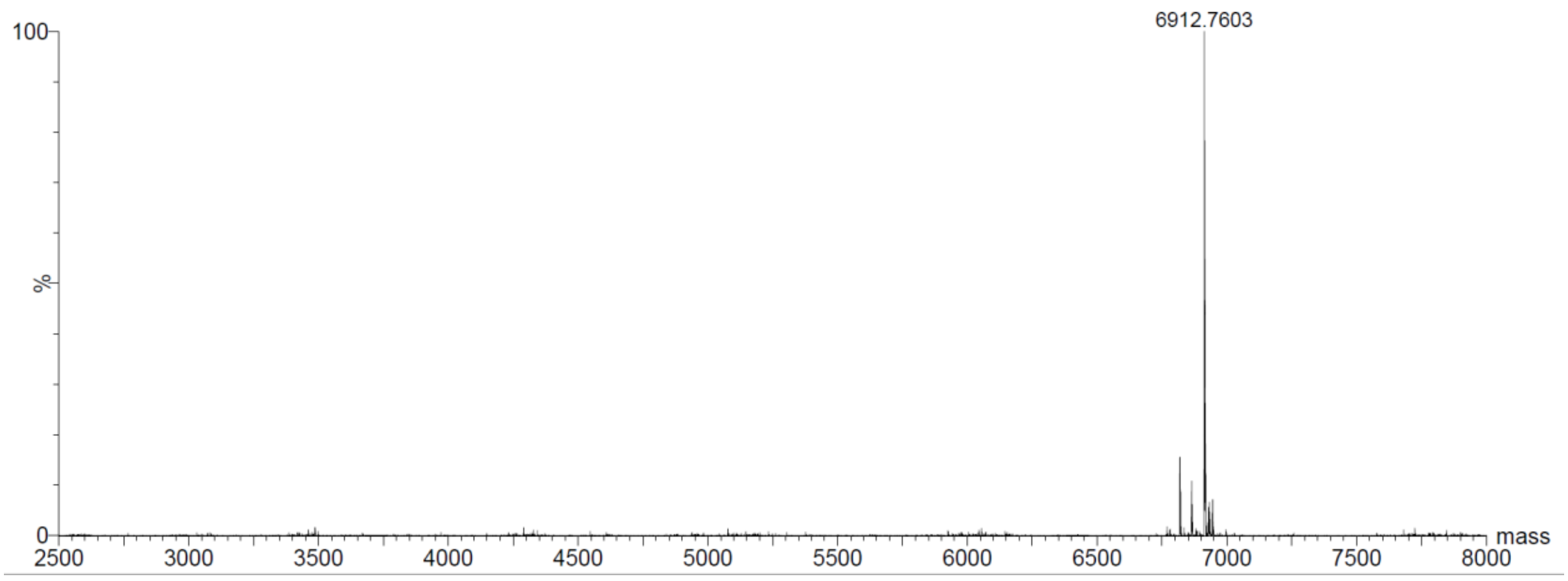

Mass spectrum (ES-) of **4t** (S-S) (y-axis = relative intensity [%], x-axis = deconvolved mass [Da]); calculated mass: 3591.5, found: 3590.5.

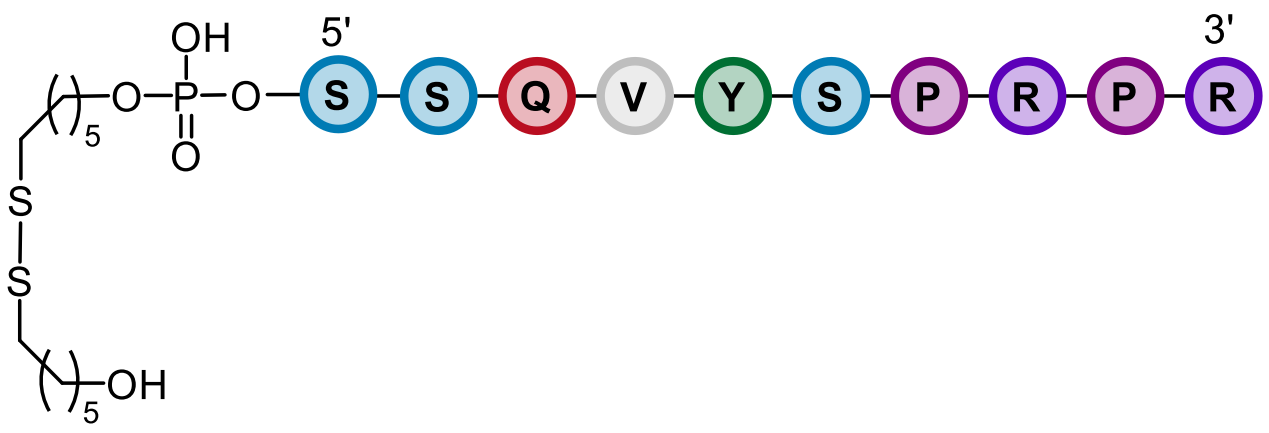

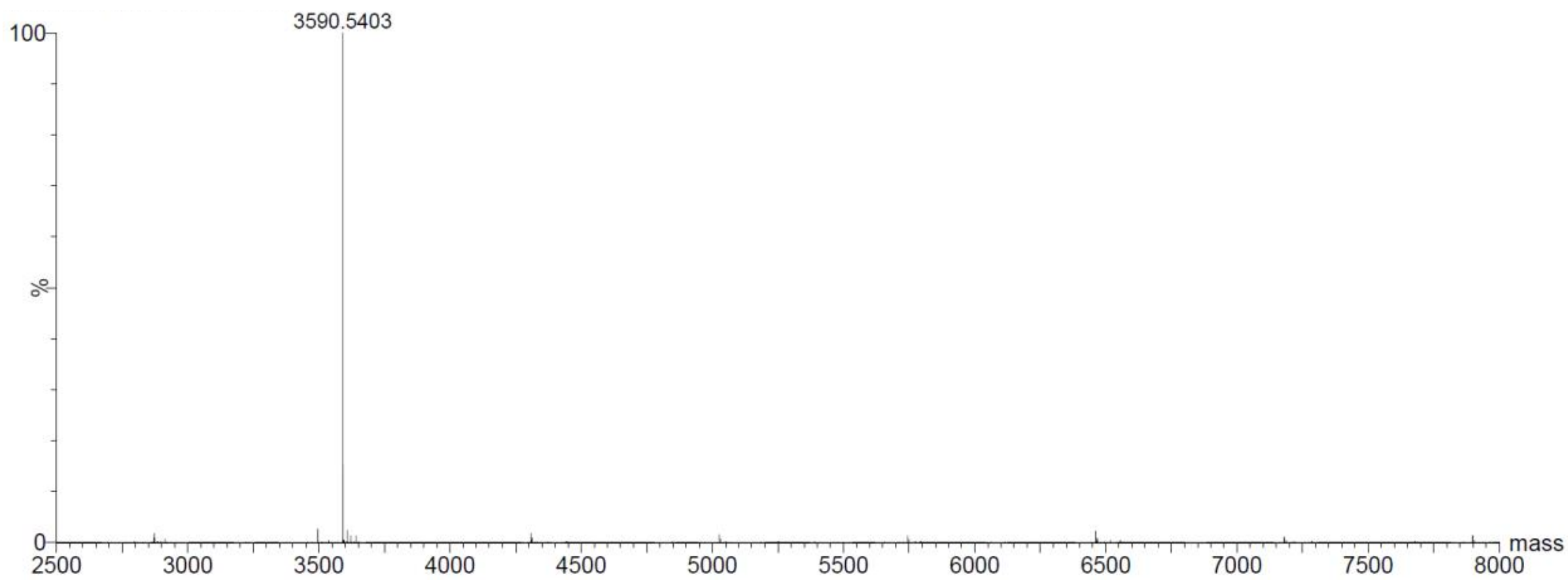

Entry 18 (4t)

Sequence	rt	Area (A)	Extinction coefficient (ε)	A/ε	Conversion
SSQVYSPRPR	4.593	2997.189	110270	0.02718045	
R-SSQVYSPRPR	5.497	17154.93	110270	0.1555721	85%

Ion-pair reversed-phase UHPLC chromatogram of the crude reaction mixture (UV absorbance at 260 nm vs. time in minutes).

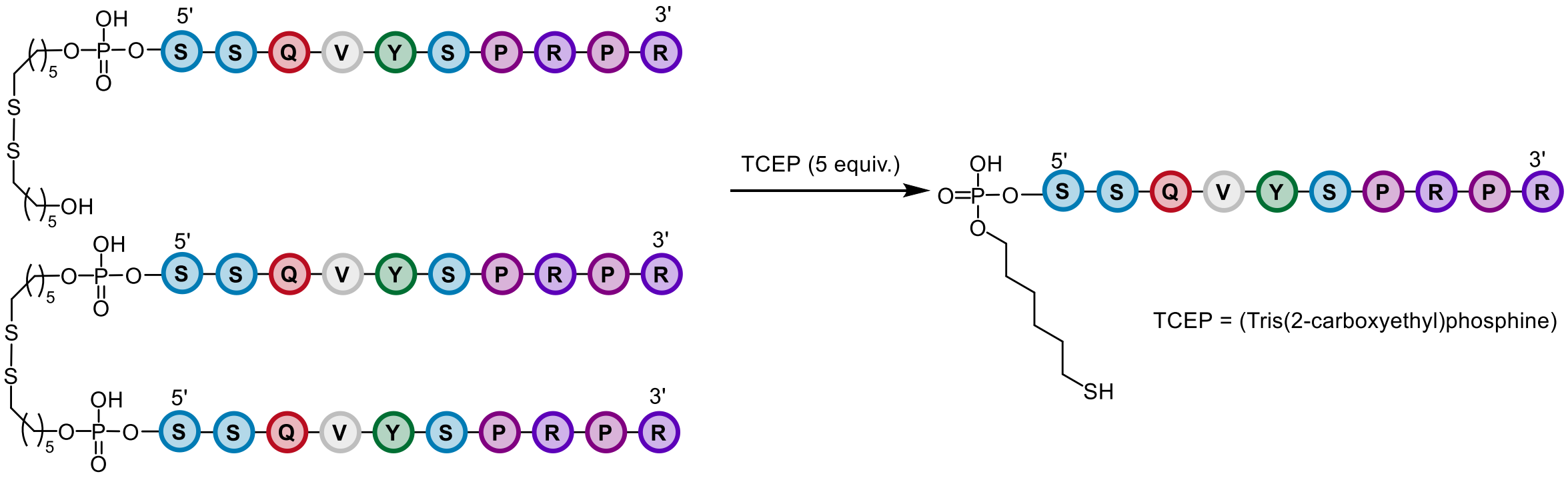

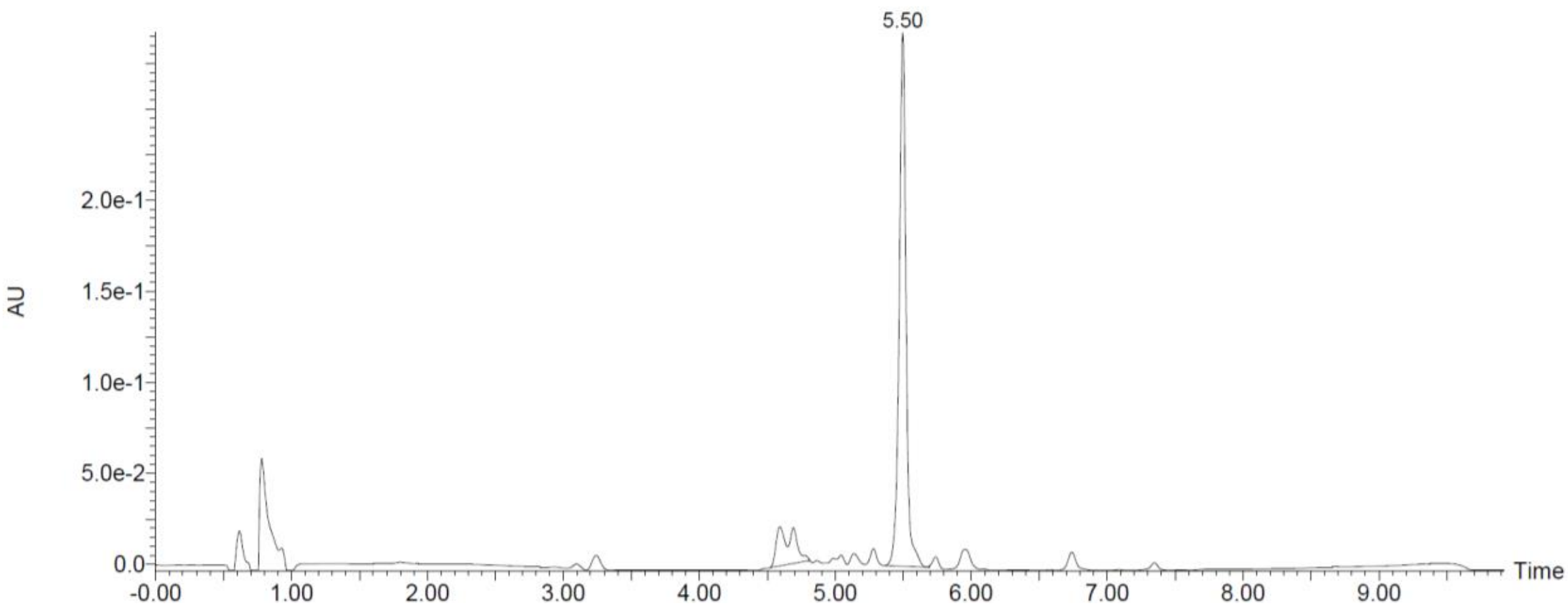

Mass spectrum (ES-) of **4t** (y-axis = relative intensity [%], x-axis = deconvolved mass [Da]); calculated mass: 3456.2, found: 3547.2.

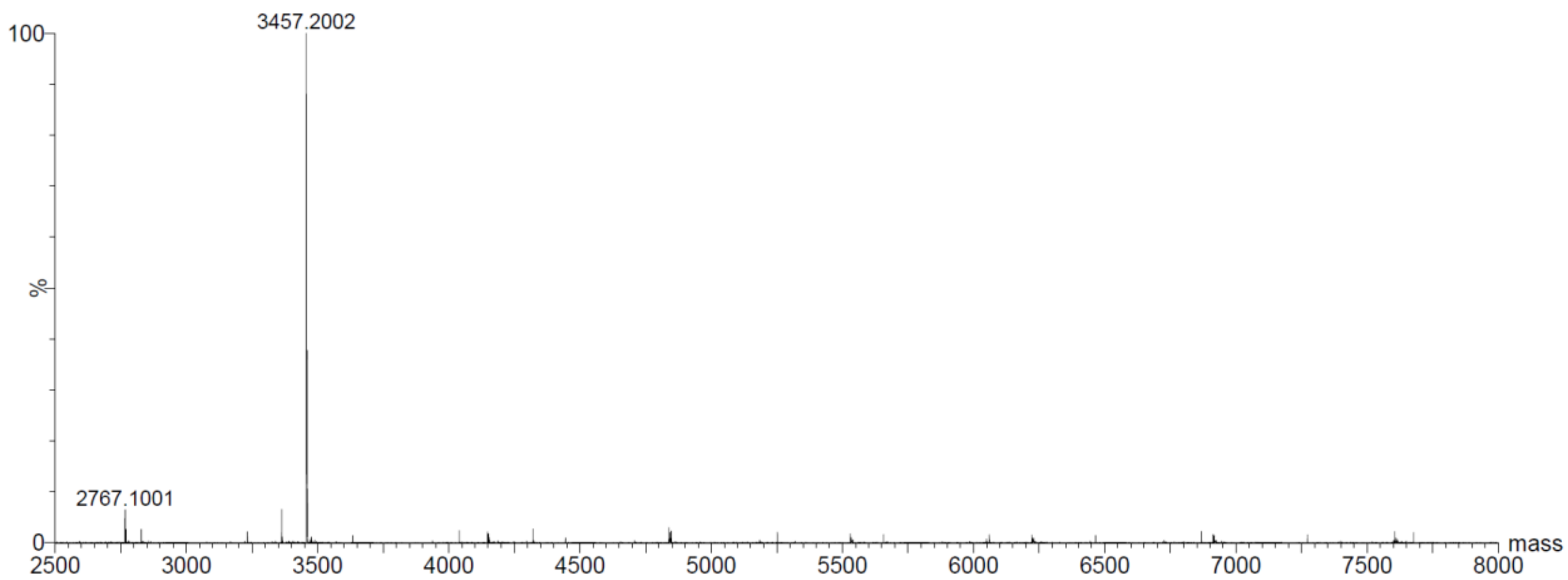

Entry 19 (4u) (PS supported mixed sequence; loading : 350 µmol g^{-1})

Sequence	rt	Area (A)	Extinction coefficient (ε)	A/ε	Conversion
SSQVYSPRPR	4.813	14377.582	110270	0.1303853	
R-SSQVYSPRPR-R	5.257	46160.59	110270	0.4186142	53%
R(SSQVYSPRPR)$_2$	5.338	30186.652	220540	0.13687608	35%
SSQVYSPRPR	5.615	10910.318	110270	0.098941852	

[R = hexaethylene glycol]

Ion-pair reversed-phase UHPLC chromatogram of the crude reaction mixture (UV absorbance at 260 nm vs. time in minutes).

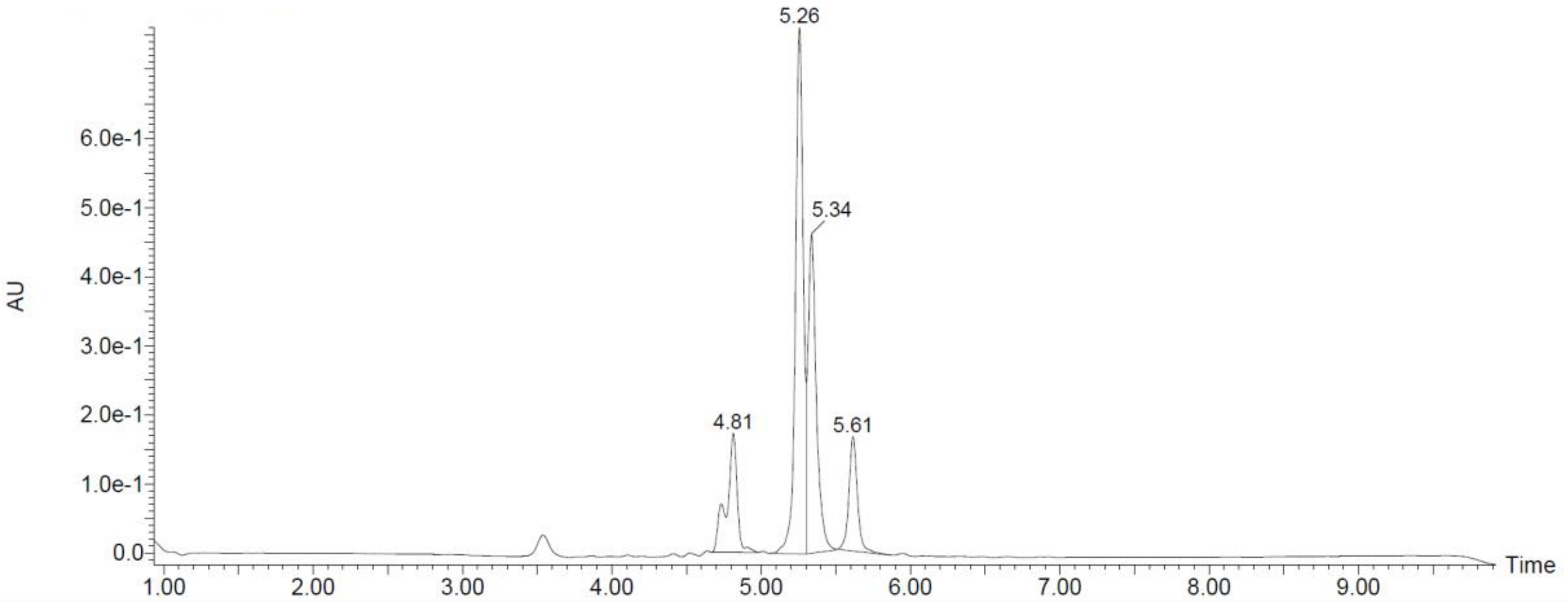

Mass spectrum (ES-) of **4u**-dimer (y-axis = relative intensity [%], x-axis = deconvolved mass [Da]); calculated mass: 6930.3 found 6929.1.

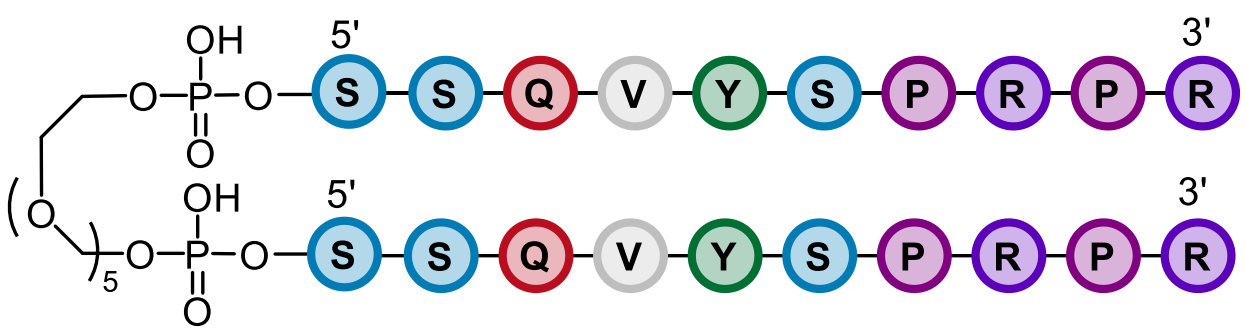

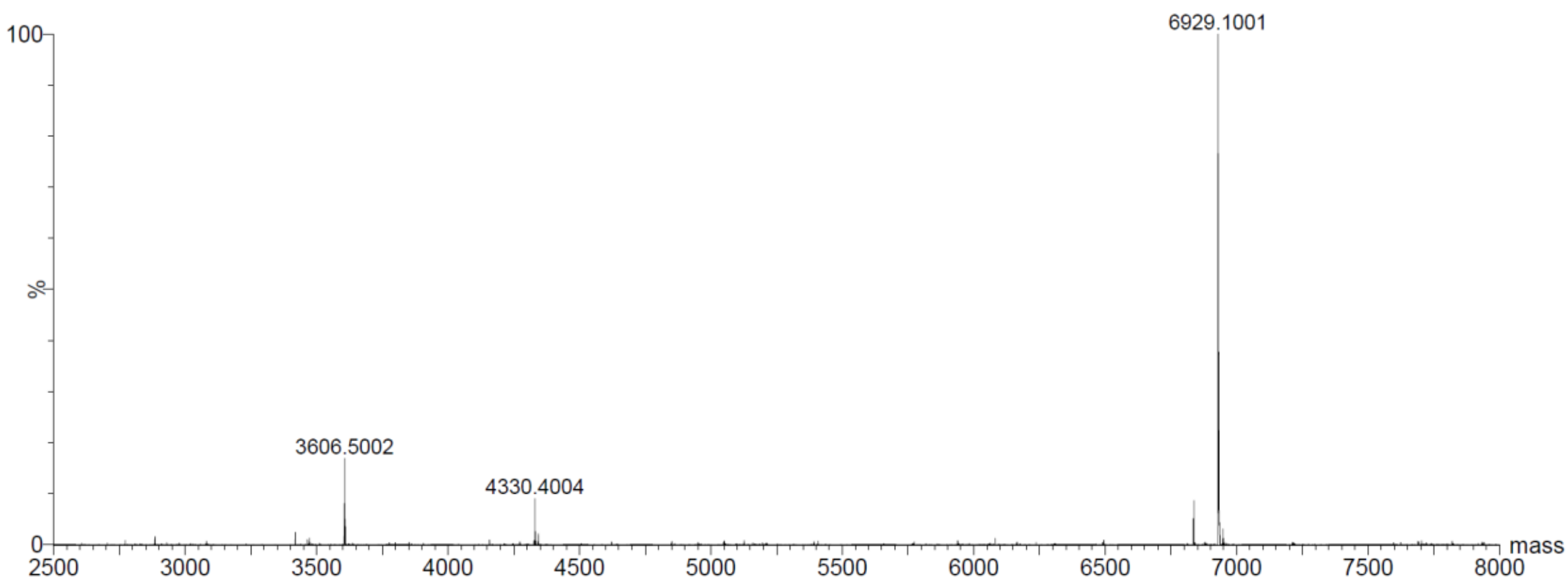

Mass spectrum (ES-) of **4u** (y-axis = relative intensity [%], x-axis = deconvolved mass [Da]); calculated mass: 3606.3, found: 3605.6.

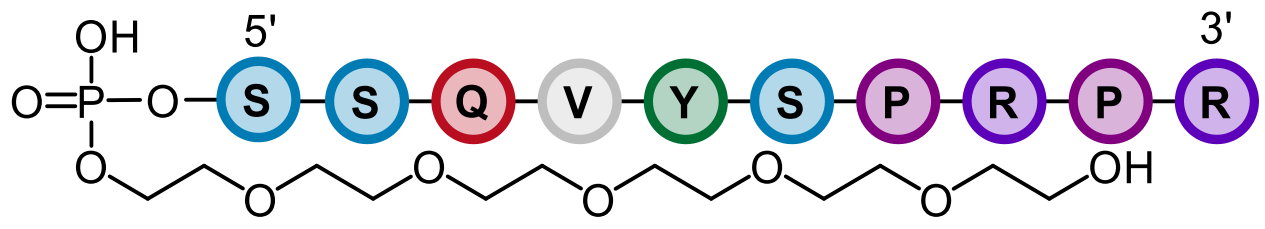

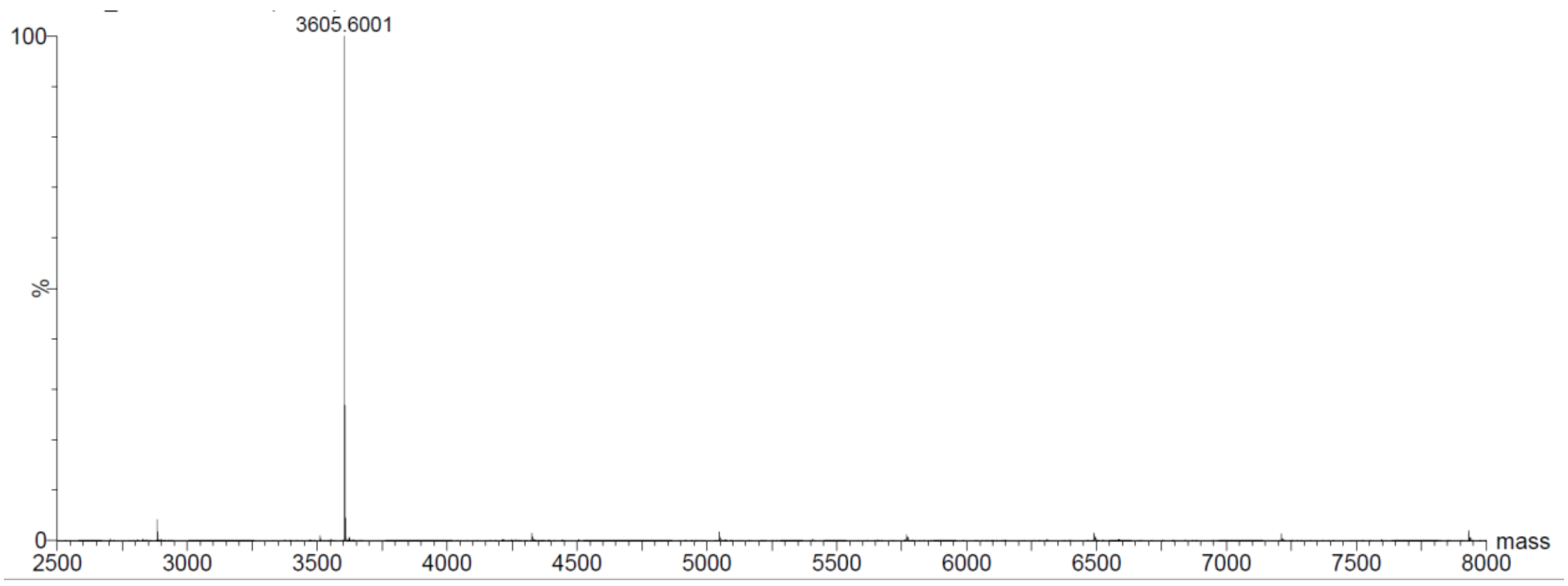

Entry 19 (**4u**) (CPG supported mixed sequence; loading : 35 µmol g^{-1})

Sequence	rt	Area (A)	Extinction coefficient (ε)	A/ε	Conversion
**R-**SSQVYSPRPR	4.971	11580.483	110270	0.10501934	59%
**R**$(SSQVYSPRPR)_2$	5.063	8758.836	220540	0.03971541	23%
SSQVYSPRPR	5.337	3537.372	110270	0.03207919	

[R = hexaethylene glycol]

Ion-pair reversed-phase UHPLC chromatogram of the crude reaction mixture (UV absorbance at 260 nm vs. time in minutes).

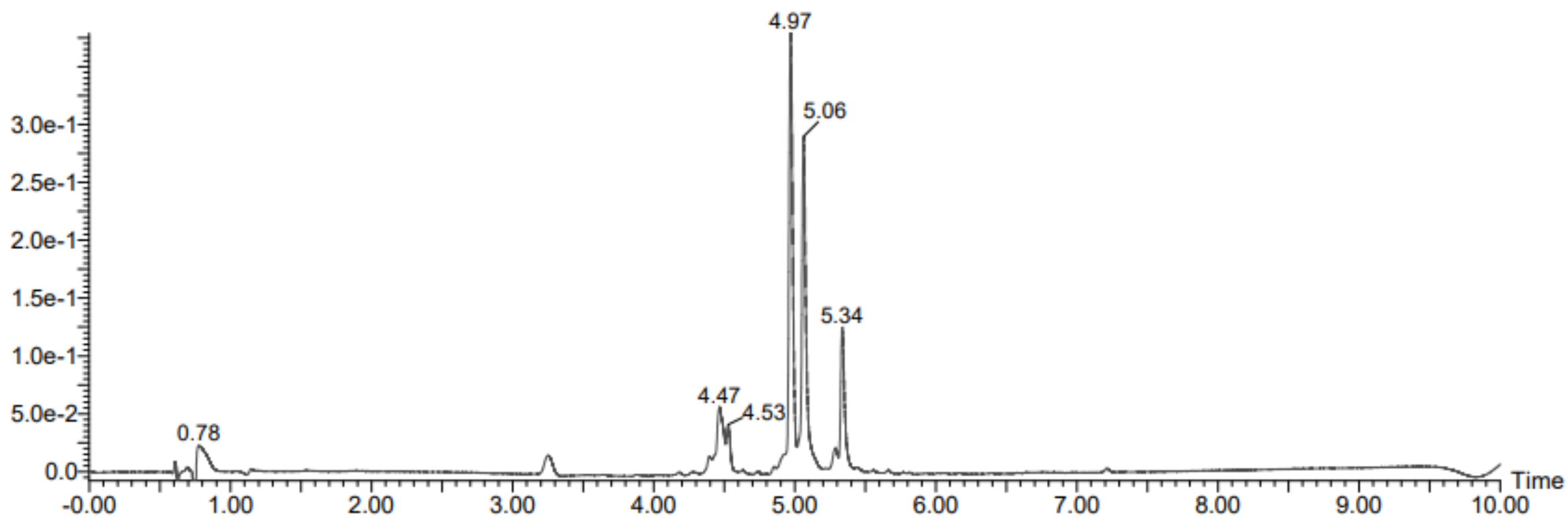

Mass spectrum (ES-) of **4u** (y-axis = relative intensity [%], x-axis = deconvolved mass [Da]); calculated mass: 3606.3, found: 3606.5.

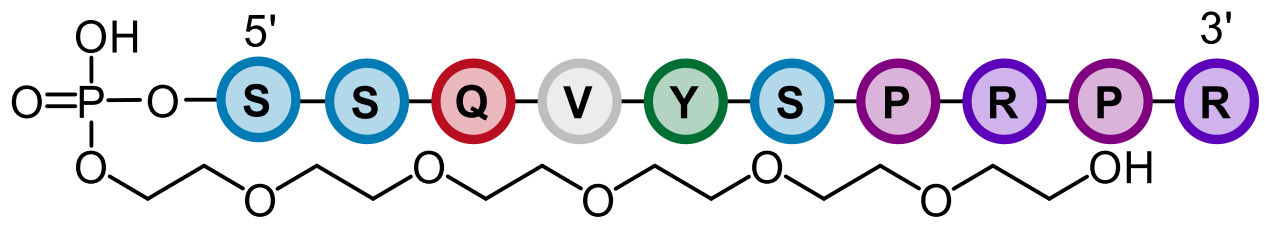

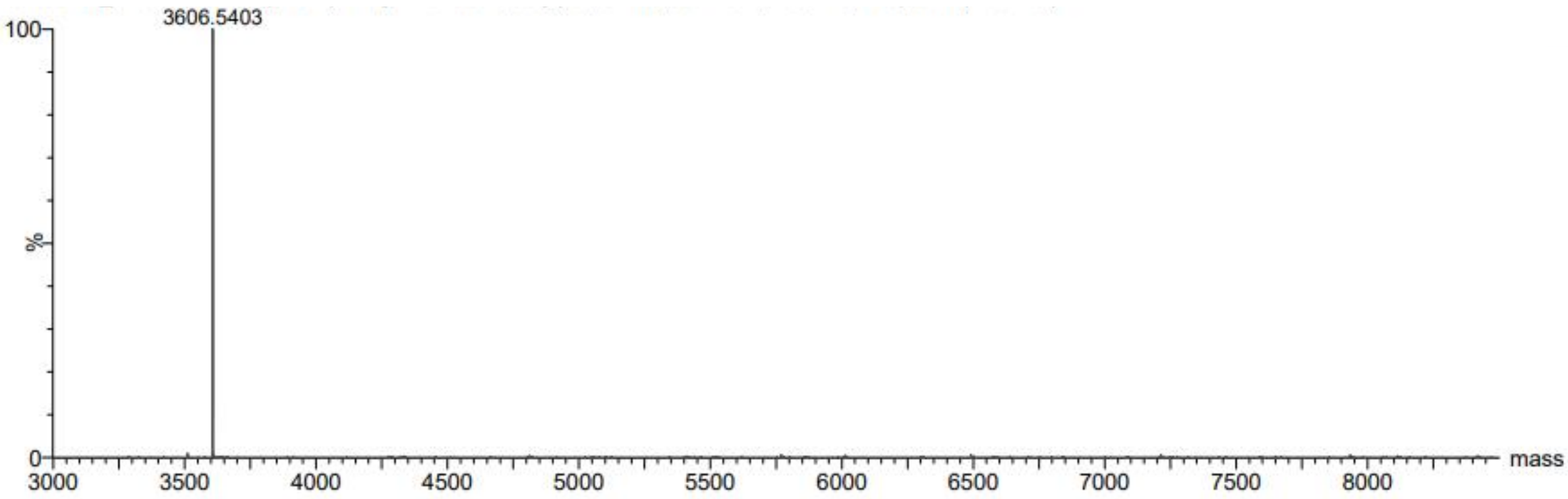

Mass spectrum (ES-) of **4u**-dimer (y-axis = relative intensity [%], x-axis = deconvolved mass [Da]); calculated mass: 6930.3, found: 6930.9.

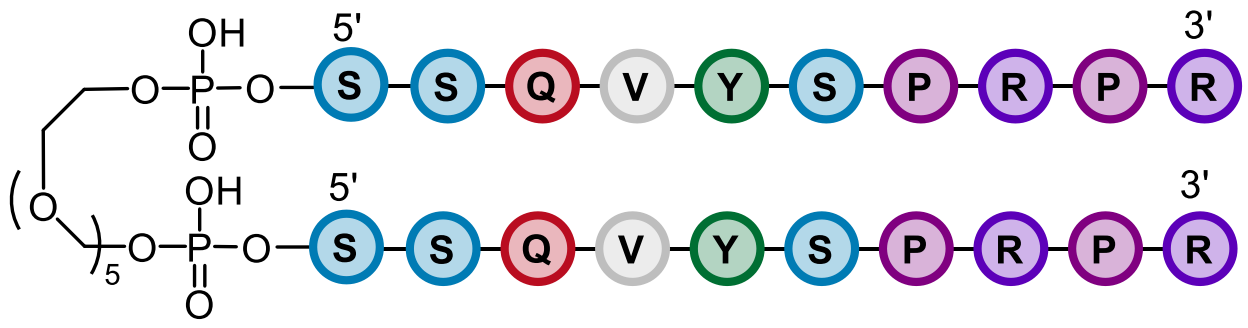

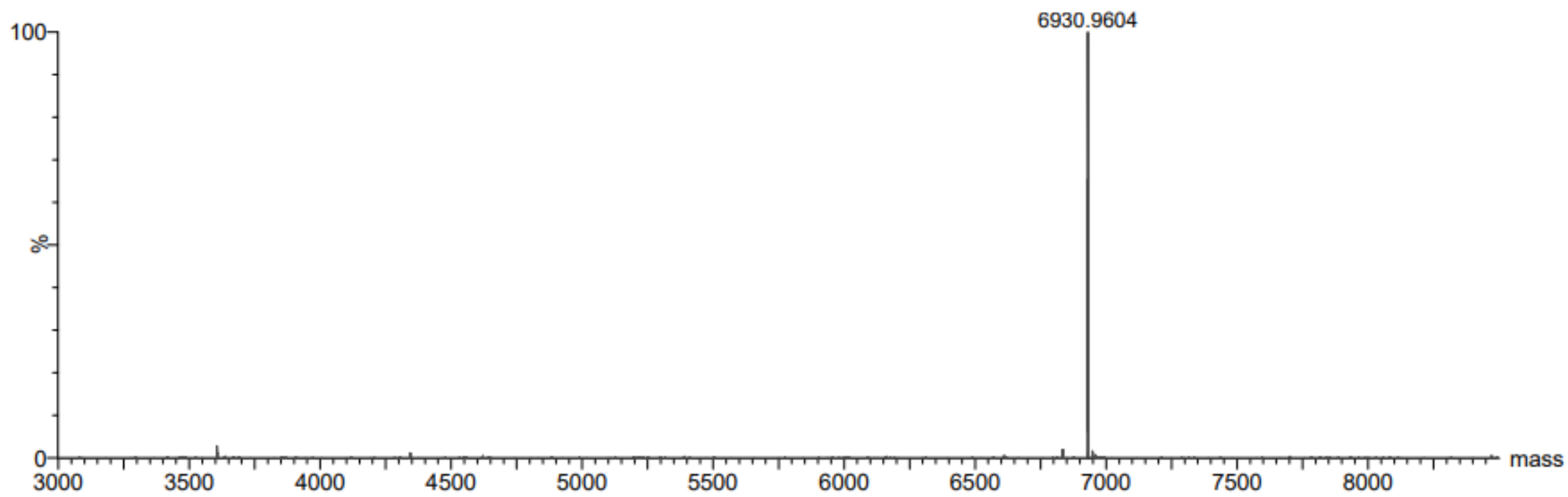

Consistent trends were observed when comparing different solid supports. For the high-loading PS support (350 µmol g^{-1}), coupling with a 0.25 M solution of hexaethylene glycol in acetonitrile afforded 53% of the desired oligonucleotide **4u** and 35% of the corresponding dimer. Performing the same reaction on the lower-loading CPG support (35 µmol g^{-1}) under identical conditions gave 59% of **4u** and 23% dimer. These results indicate that the overall distribution of monomeric and dimeric products is largely maintained across different supports, with the lower-loading CPG providing a slight improvement in the ratio of desired monomer to dimer.

To evaluate the influence of support loading and alcohol excess on product distribution, comparative coupling experiments were performed using lower-loading CPG-supported oligonucleotide sequences (35 µmol g^{-1}). Couplings with hexaethylene glycol were carried out in acetonitrile using alcohol concentrations ranging from 0.25 to 3.0 M. UHPLC analysis of the crude reaction mixtures demonstrated that increasing alcohol concentration progressively suppressed formation of the dimeric product, consistent with increased intermolecular coupling events with the external nucleophile under conditions of high nucleophile excess. However, elevated alcohol concentrations resulted in a marked decrease in overall coupling efficiency of **4u**, accompanied by increased levels of unreacted phosphoramidite.

Representative product distributions are summarized below.

For the CPG supported mixed sequence (loading 35 µmol g^{-1})

Entry	alcohol (M)	**4u** (%)	**4u**- difunctionalized (%)	Phosphoramidate
1	0.25	59	22	18
2	1	51	4	44
3	2	36	1	62
4	3	24	-	75

Stacked ion pair reversed-phase UHPLC chromatogram of the crude reaction mixture (UV absorbance at 260 nm *vs*. time in minutes).

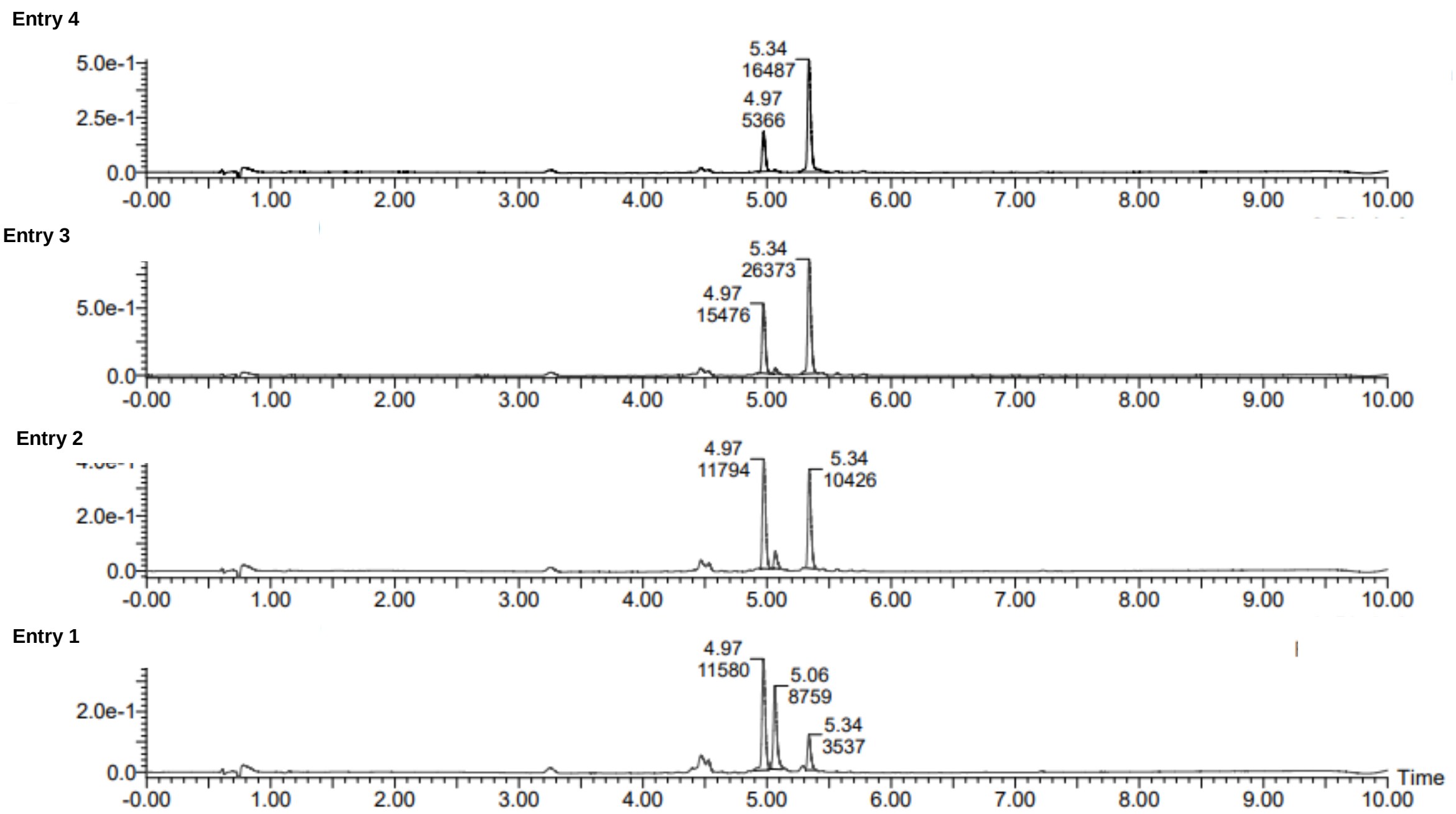

Entry 20 (4v)

Sequence	rt	Area (A)	Extinction coefficient (ε)	A/ε	Conversion
TTTTTTTTTT	4.305	1587.723	81600	0.01945739	
TTTTTTTTTT-**Ar**	4.779	29495.584	81600	0.36146549	95%

[Ar = phenol]

Ion-pair reversed-phase UHPLC chromatogram of the crude reaction mixture (UV absorbance at 260 nm vs. time in minutes).

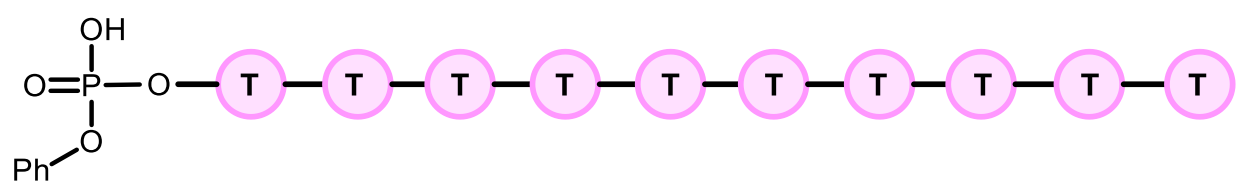

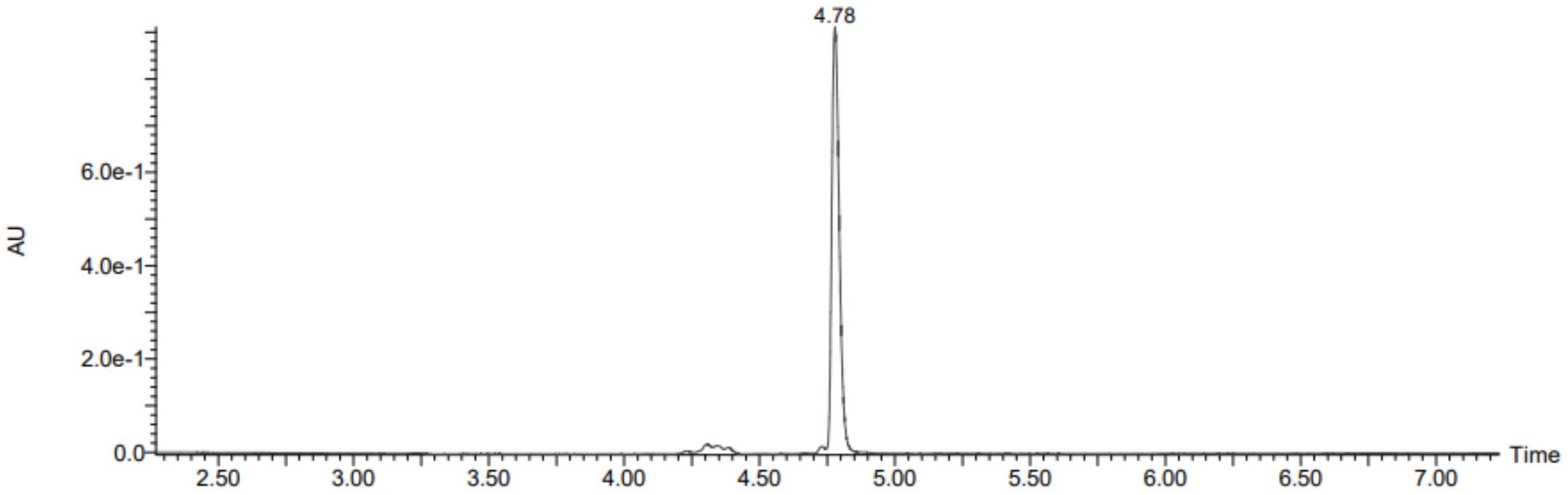

Mass spectrum (ES-) of **4v** (y-axis = relative intensity [%], x-axis = deconvolved mass [Da]); calculated mass: 3136.1, found: 3136.9.

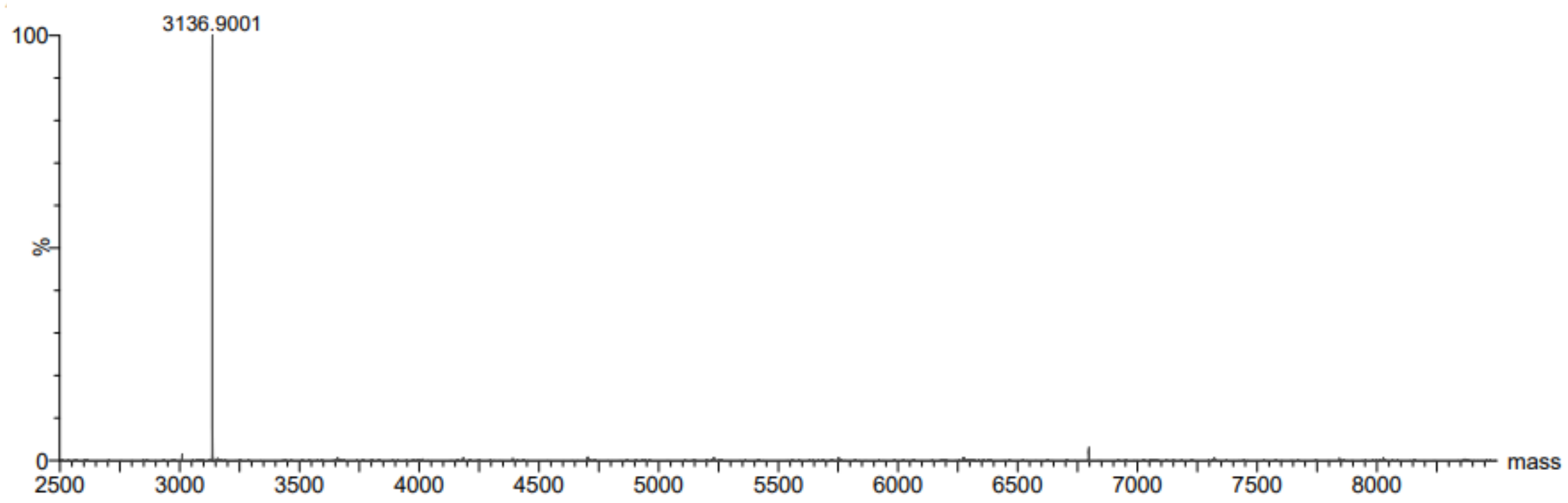

Entry 20 (4v')

Sequence	rt	Area (A)	Extinction coefficient (ε)	A/ε	Conversion
SSQVYSPRPR	4.454	1264.93	110270	0.01147121	
SSQVYSPRPR	4.509	612.009	110270	0.0055501	
Ar-SSQVYSPRPR	4.871	30406.381	110270	0.27574482	94%

[Ar = phenol]

Ion-pair reversed-phase UHPLC chromatogram of the crude reaction mixture (UV absorbance at 260 nm vs. time in minutes).

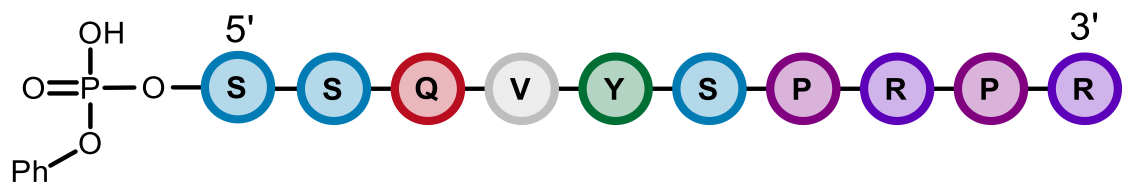

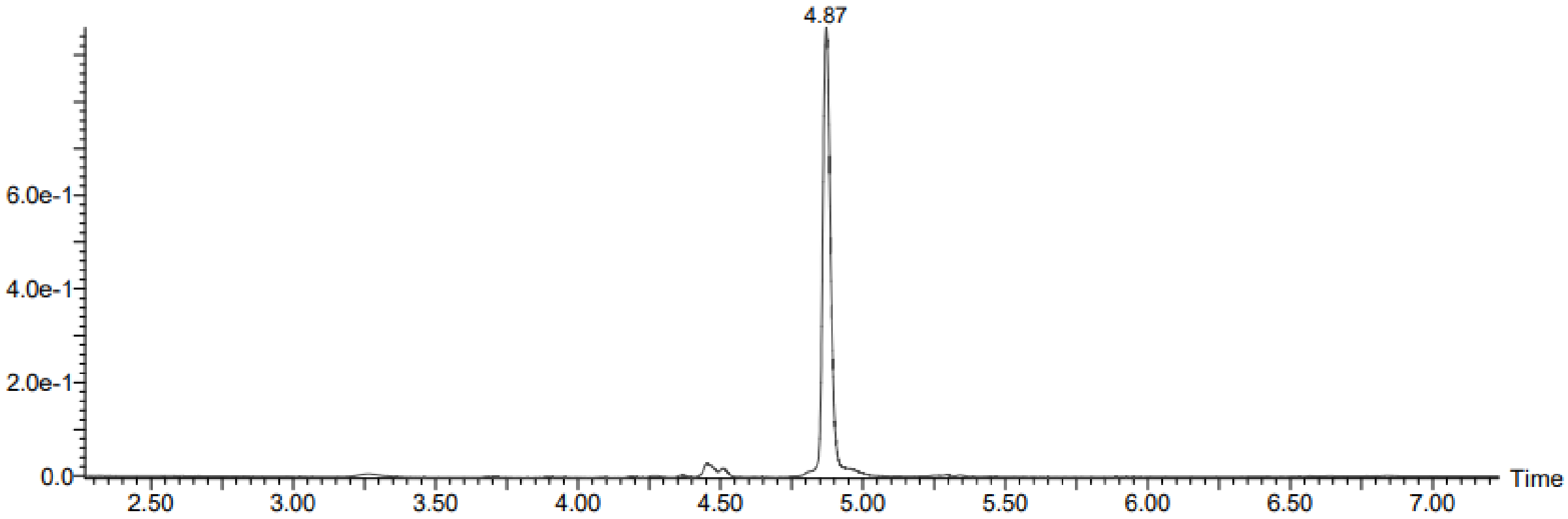

Mass spectrum (ES-) of **4v'** (y-axis = relative intensity [%], x-axis = deconvolved mass [Da]); calculated mass: 3418.1, found: 3418.1.

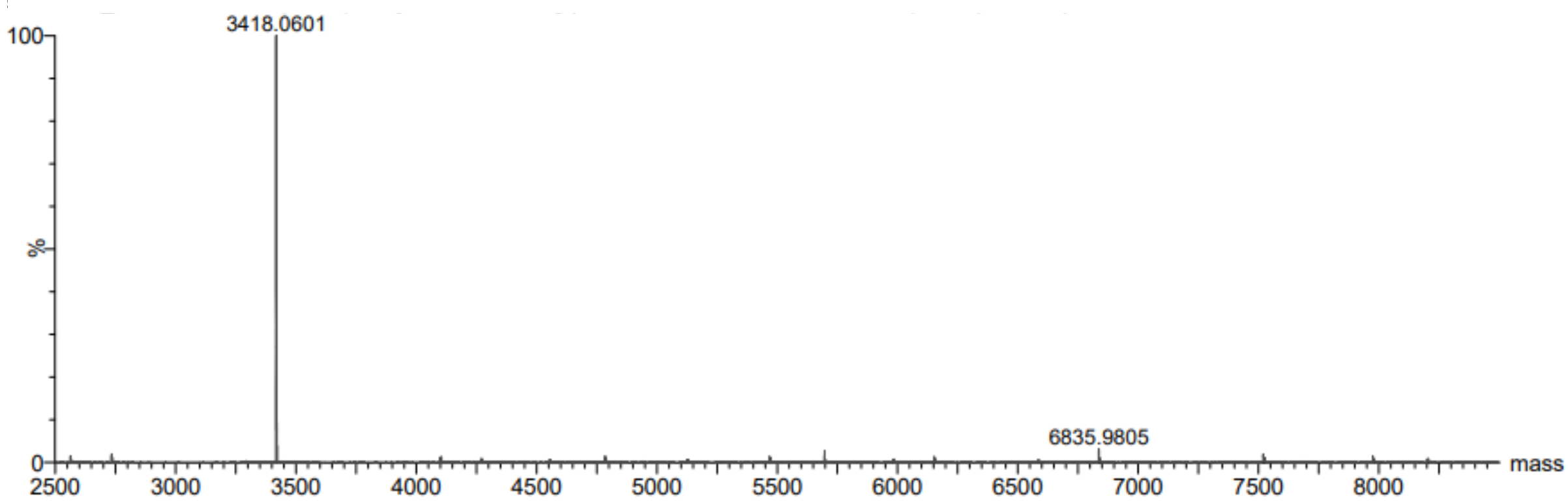

Entry 21 (4w)

Sequence	rt	Area (A)	Extinction coefficient (ε)	A/ε	Conversion
SSQVYSPRPR	2.173	203774.297	110270	1.84795771	
Ar-SSQVYSPRPR	5.305	1950089.25	110270	17.6846762	90%

[Ar = δ-Tocopherol]

Ion-pair reversed-phase UHPLC chromatogram of the crude reaction mixture (UV absorbance at 260 nm vs. time in minutes).

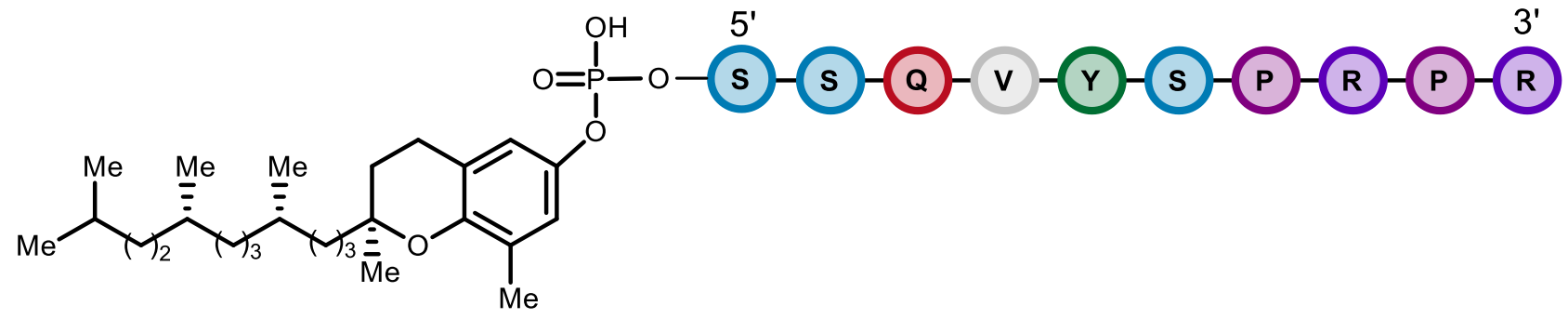

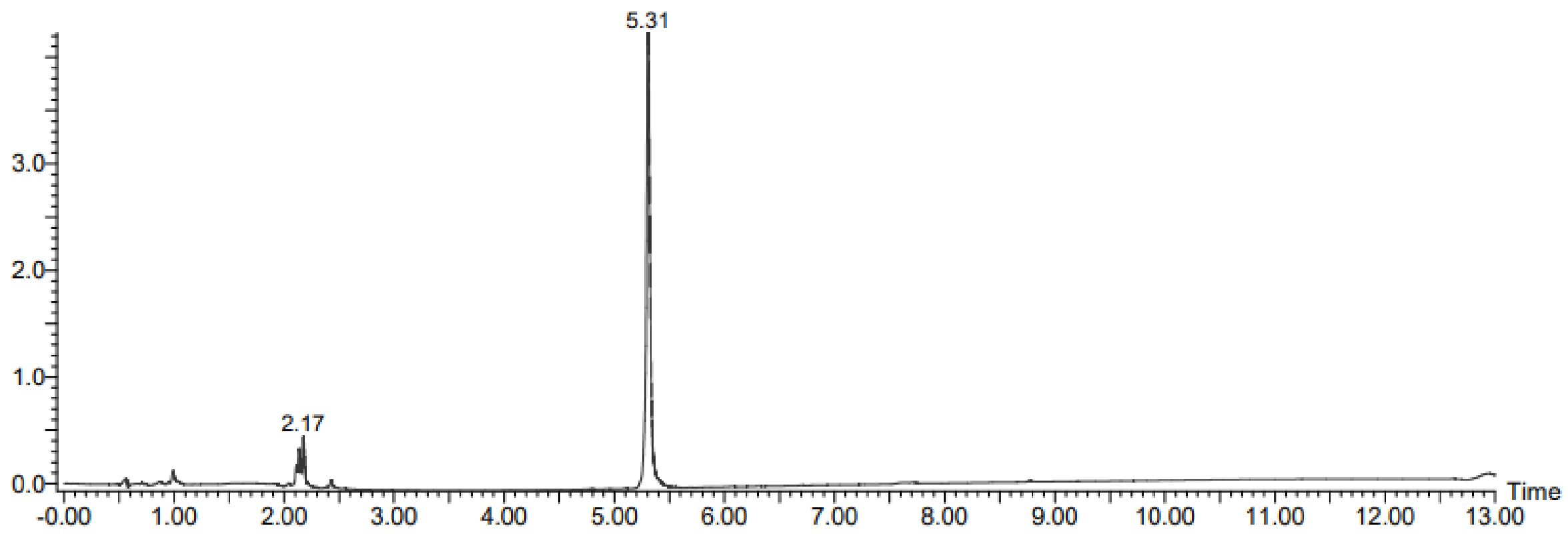

Mass spectrum (ES-) of **4w** (y-axis = relative intensity [%], x-axis = deconvolved mass [Da]); calculated mass: 3726.6, found: 3726.0.

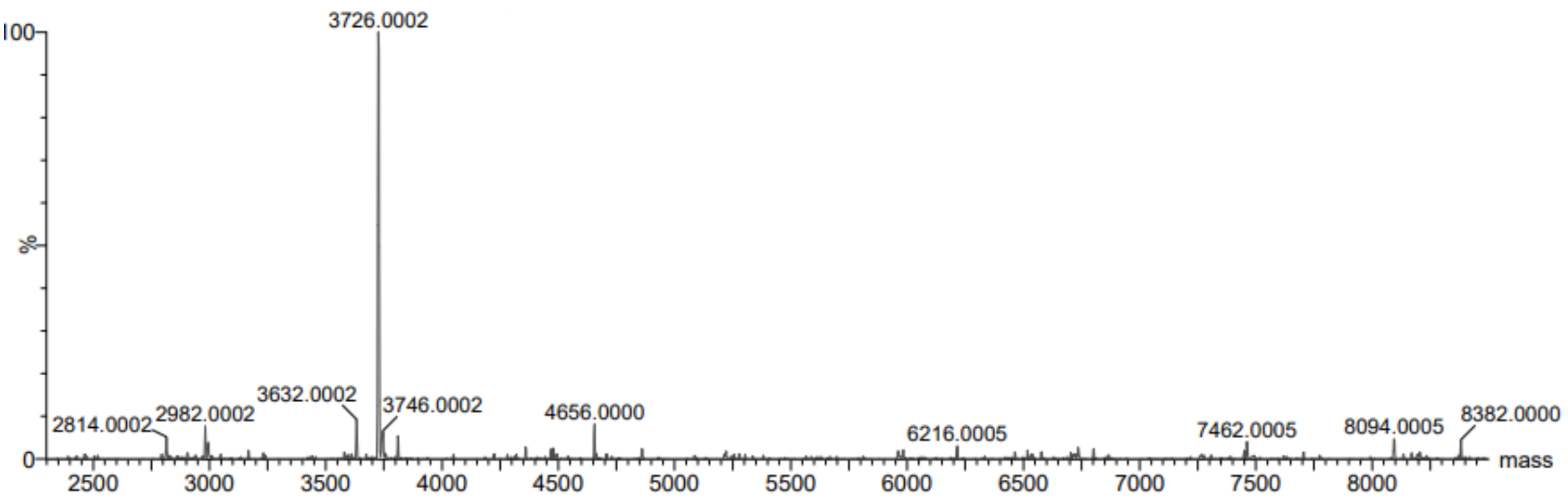

Entry 22 (4x)

Sequence	rt	Area (A)	Extinction coefficient (ε)	A/ε	Conversion
AKQVYSKRPR	4.374	2773.21	110270	0.02514927	
**R-**AKQVYSKRPR	6.779	24472.252	110270	0.22193028	90%

[R = $CH_3(CH_2)_8CH_2O$]

Ion-pair reversed-phase UHPLC chromatogram of the crude reaction mixture (UV absorbance at 260 nm vs. time in minutes).

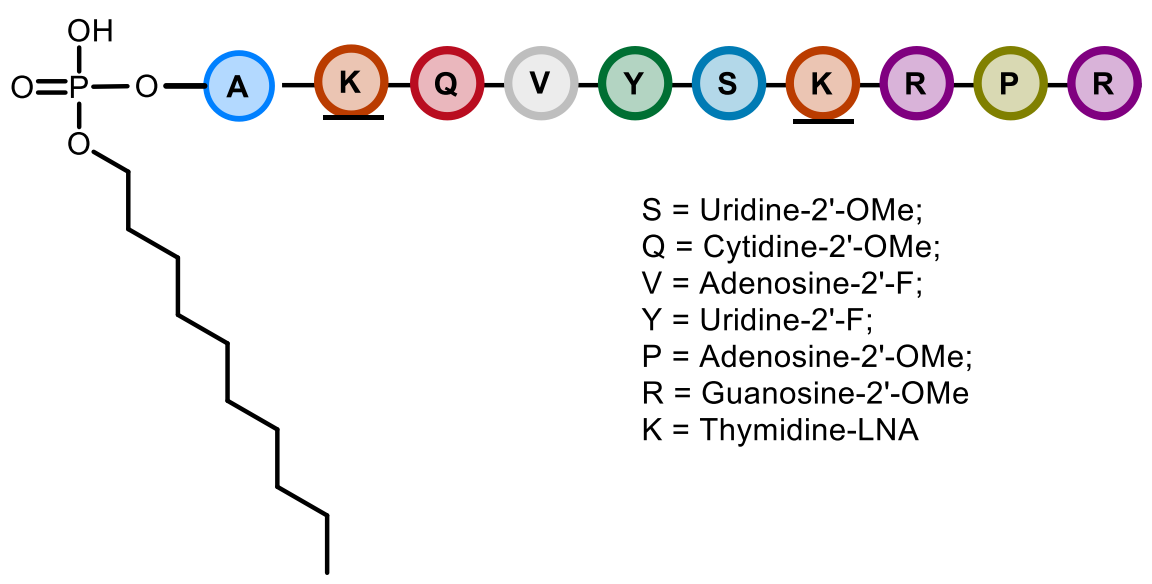

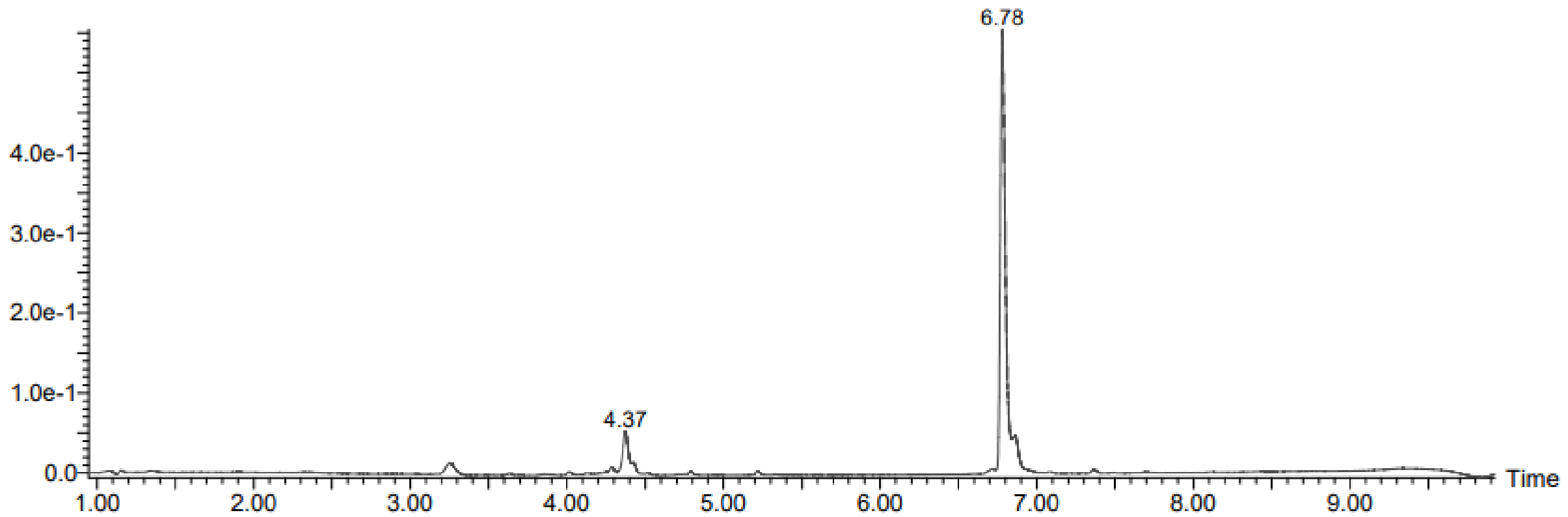

Mass spectrum (ES-) of **4x** (y-axis = relative intensity [%], x-axis = deconvolved mass [Da]); calculated mass: 3476.3, found: 3475.5.

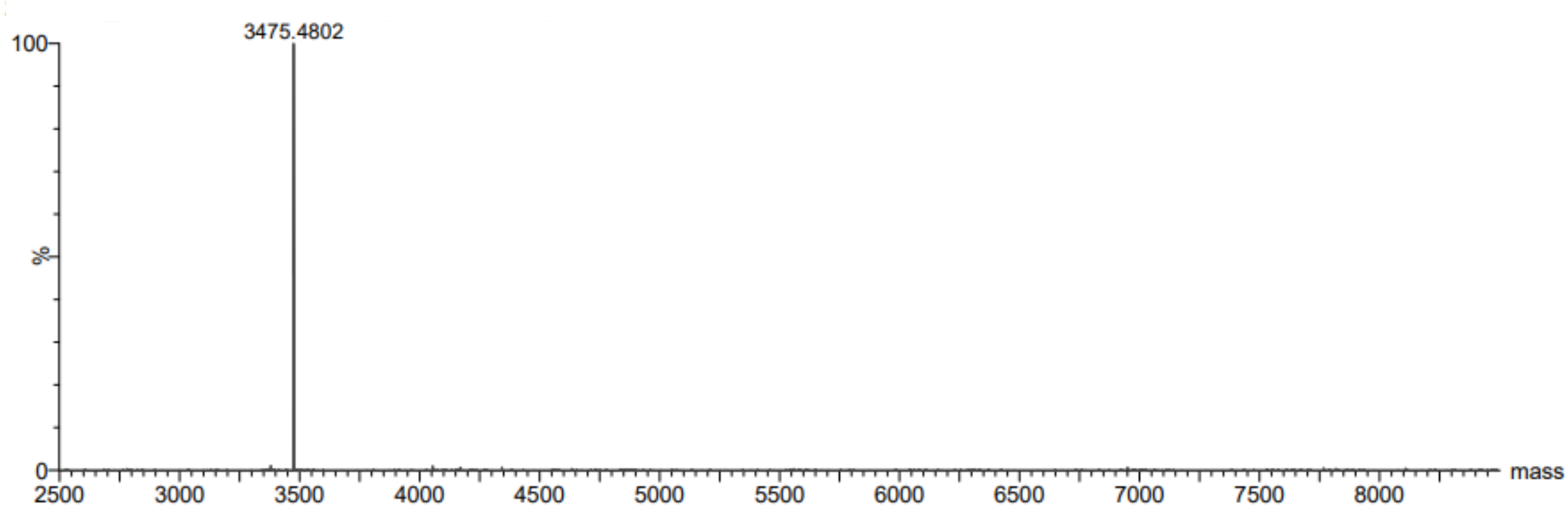

Entry 22 (4y)

Sequence	rt	Area (A)	Extinction coefficient (ε)	A/ε	Conversion
TKQVYSKRPR	4.378	3507.6	103470	0.03389968	
**R-**TKQVYSKRPR	6.923	33859.105	103470	0.32723596	91%

[R = $CH_3(CH_2)_8CH_2O$]

Ion-pair reversed-phase UHPLC chromatogram of the crude reaction mixture (UV absorbance at 260 nm vs. time in minutes).

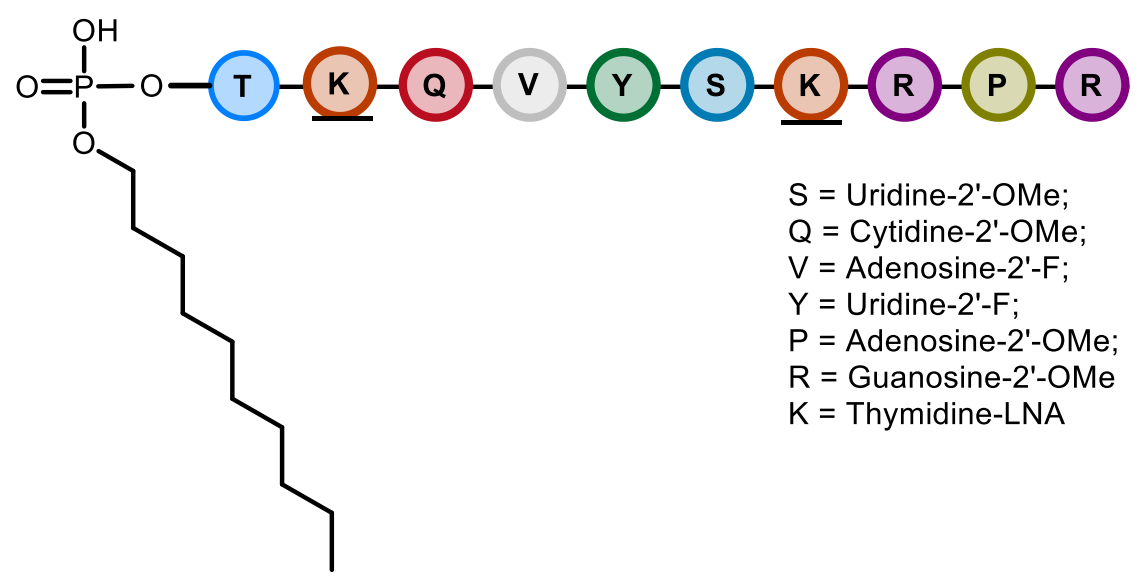

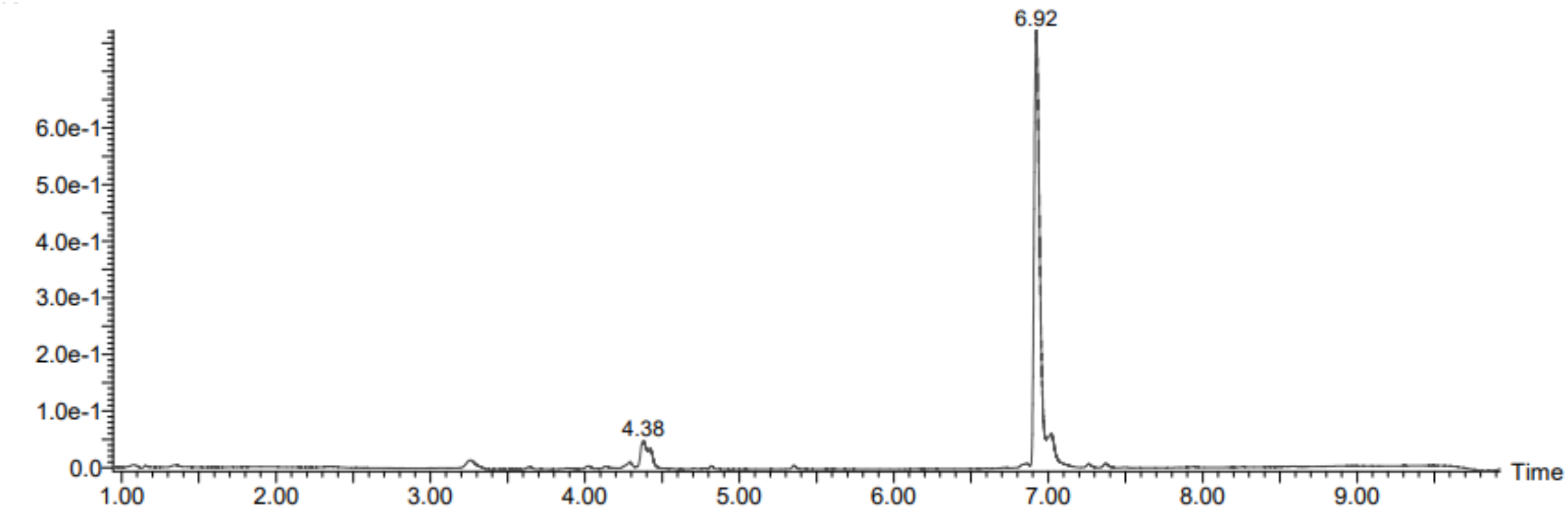

Mass spectrum (ES-) of **4y** (y-axis = relative intensity [%], x-axis = deconvolved mass [Da]); calculated mass: 3467.3, found: 3465.8.

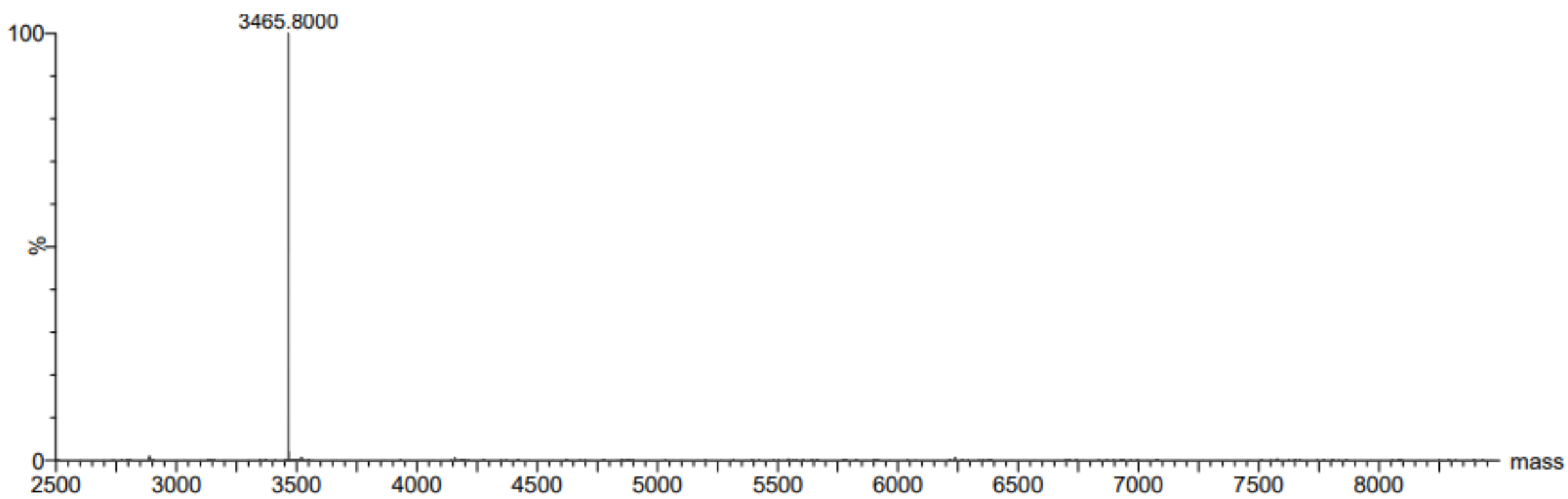

Entry 22 (4z)

Sequence	rt	Area (A)	Extinction coefficient (ε)	A/ε	Conversion
GKQVYSKRPR	4.27	3315.775	107080	0.0309654	
**R-**GKQVYSKRPR	6.572	34906.98	107080	0.32598973	91%

[R = $CH_3(CH_2)_8CH_2O$]

Ion-pair reversed-phase UHPLC chromatogram of the crude reaction mixture (UV absorbance at 260 nm vs. time in minutes).

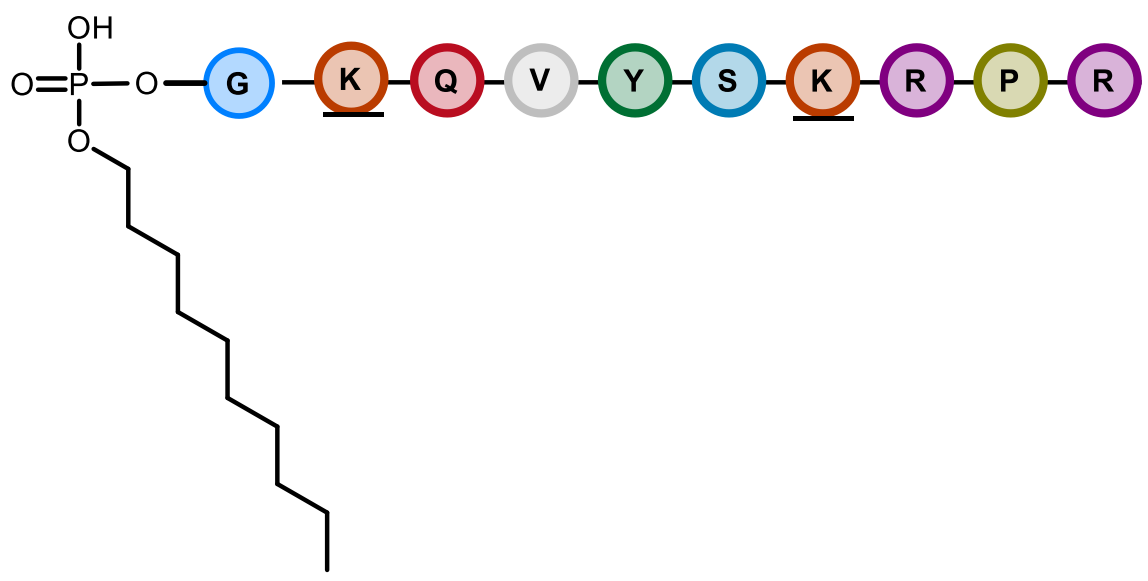

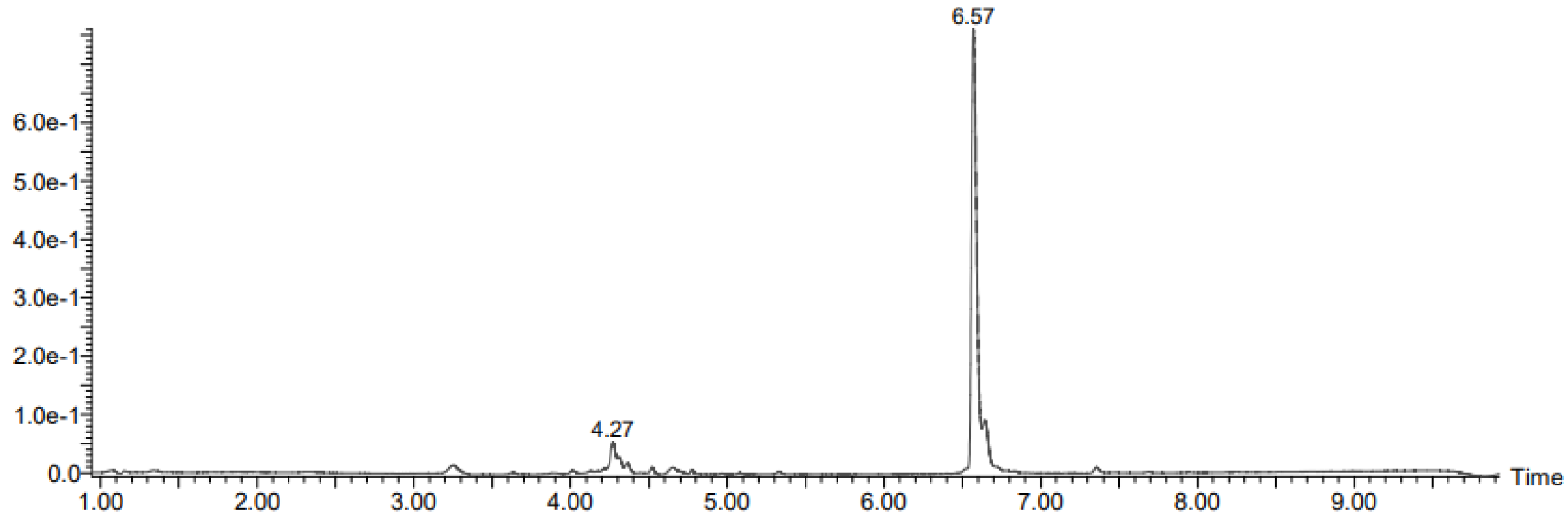

Mass spectrum (ES-) of **4z** (y-axis = relative intensity [%], x-axis = deconvolved mass [Da]); calculated mass: 3492.3, found: 3490.4.

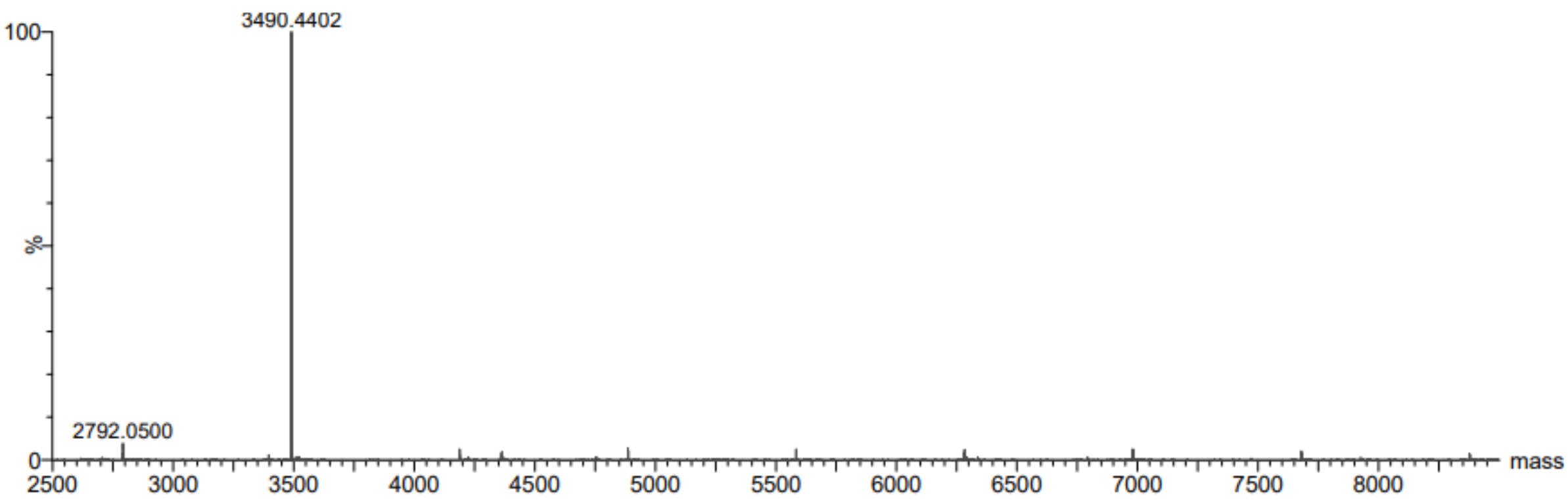

Entry 22 (4aa)

Sequence	rt	Area (A)	Extinction coefficient (ε)	A/ε	Conversion
CKQVYSKRPR	4.399	2022.2	102120	0.01980219	
CKQVYSKRPR	4.466	498.406	102120	0.00488059	
R-CKQVYSKRPR	6.818	23460.027	102120	0.22972999	90%

[R = $CH_3(CH_2)_8CH_2O$]

Ion-pair reversed-phase UHPLC chromatogram of the crude reaction mixture (UV absorbance at 260 nm vs. time in minutes).

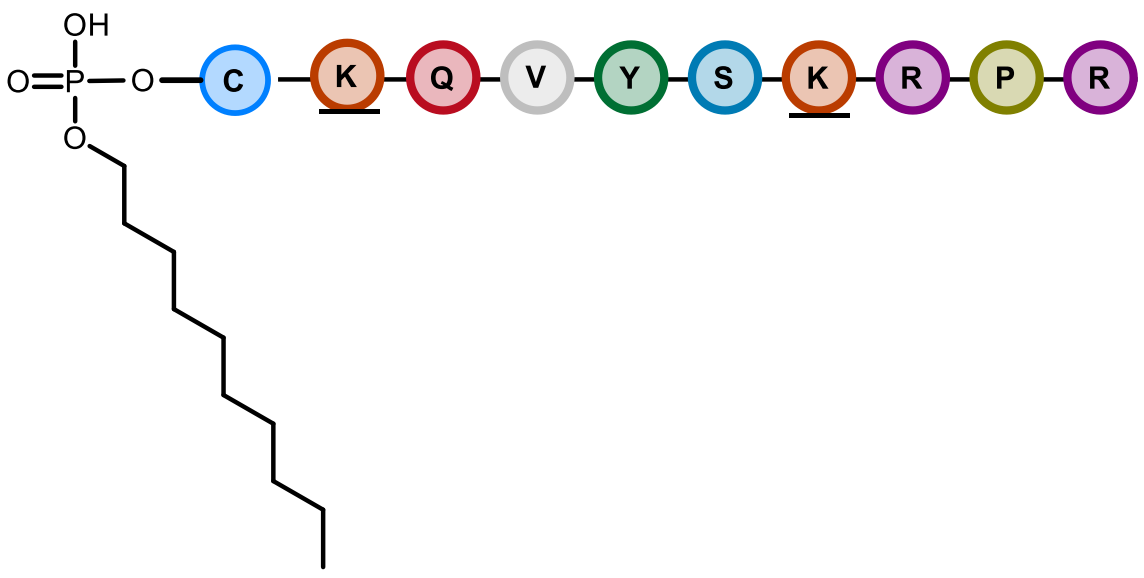

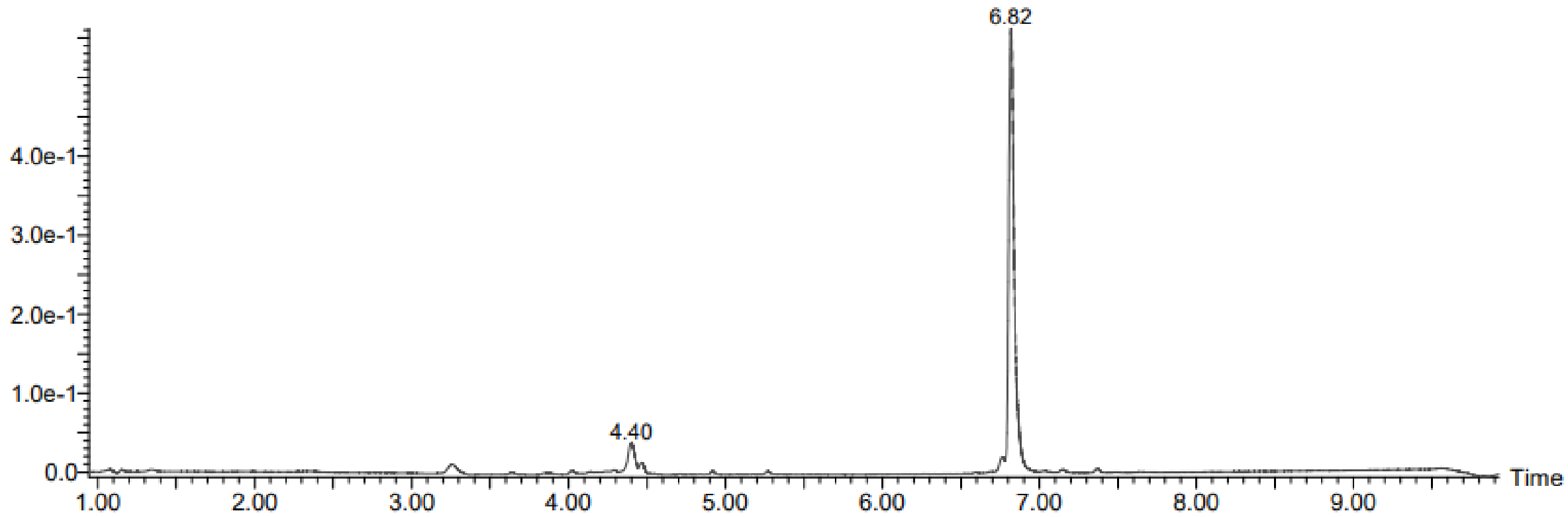

Mass spectrum (ES-) of **4aa** (y-axis = relative intensity [%], x-axis = deconvolved mass [Da]); calculated mass: 3452.3, found: 3451.3.

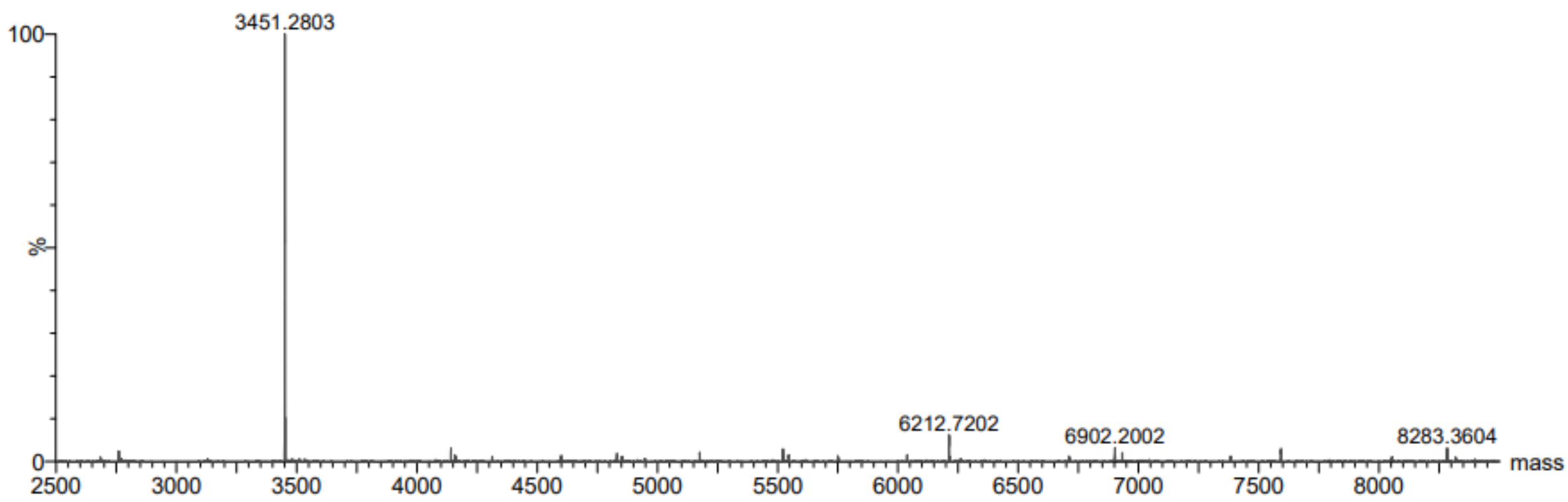

4.3 Labelling of the NH_2 group

The crude reaction mixture **4q**, was purified according to the general procedure described in Section 2 and was subsequently used for the labelling studies.

Two separate reactions were performed using different fluorescent dyes.

BODIPY-FL NHS ester (5 equiv.) or sulfo-Cyanine 3 NHS ester (5 equiv.) in dimethyl sulfoxide (20 mM, 3.75 µL) was mixed with a water solution of the amino-modified oligonucleotide (15 mM, 1.00 µL, 1 equiv.), sodium bicarbonate buffer (pH 8.3, 1 M, 1.00 µL) and dimethyl sulfoxide (4.25 µL). The mixtures were left to react for 1 h at an ambient temperature (23 ^{o}C). Mass spectrometric analysis showed new peaks at the expected molecular masses, confirming successful labelling of the NH_2 functionality.

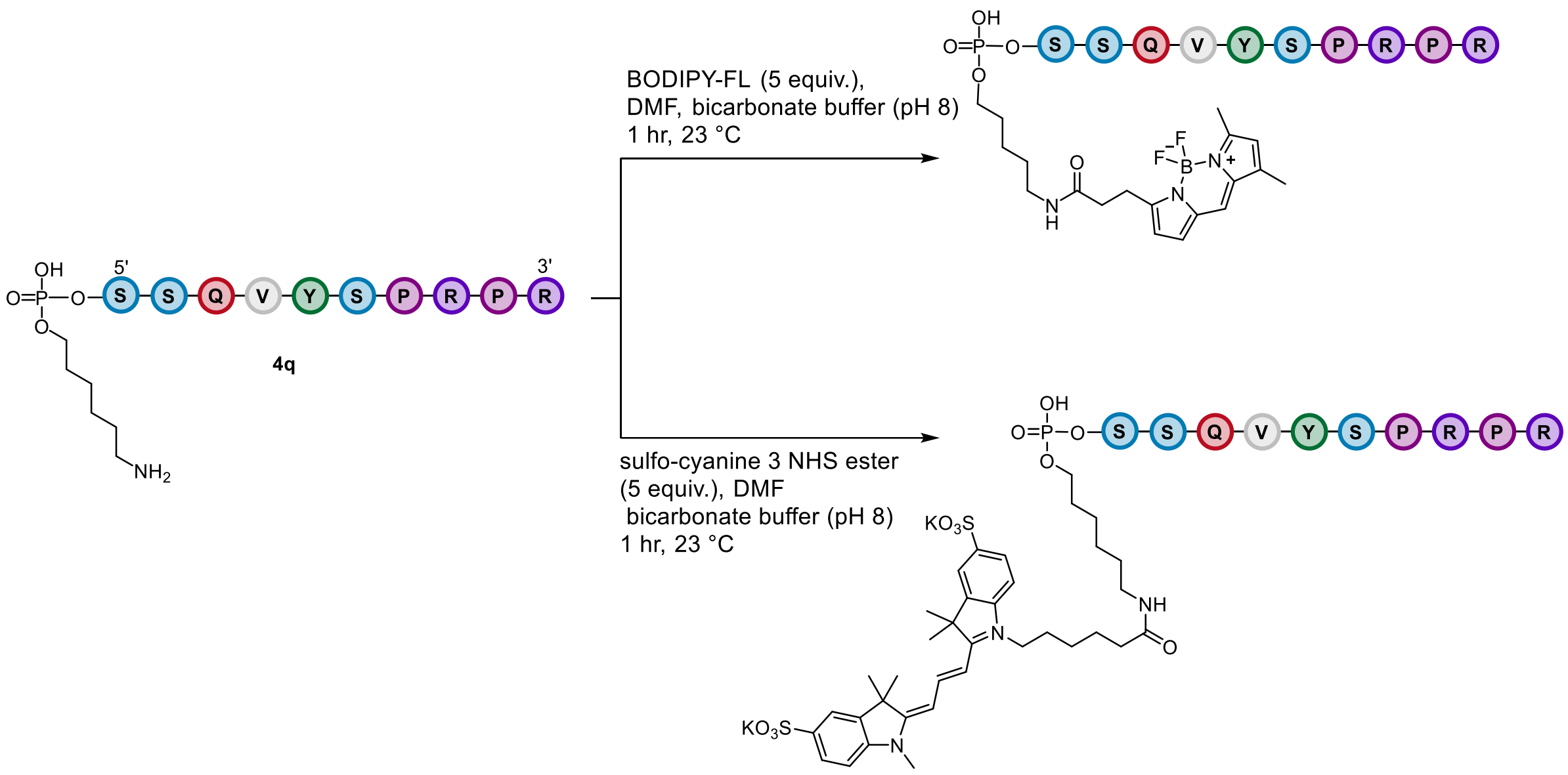

Ion-pair reversed-phase UHPLC chromatogram of the crude reaction mixture obtained from the BODIPY-FL NHS labelled modified oligonucleotide (UV absorbance at 260 nm vs. time in minutes).

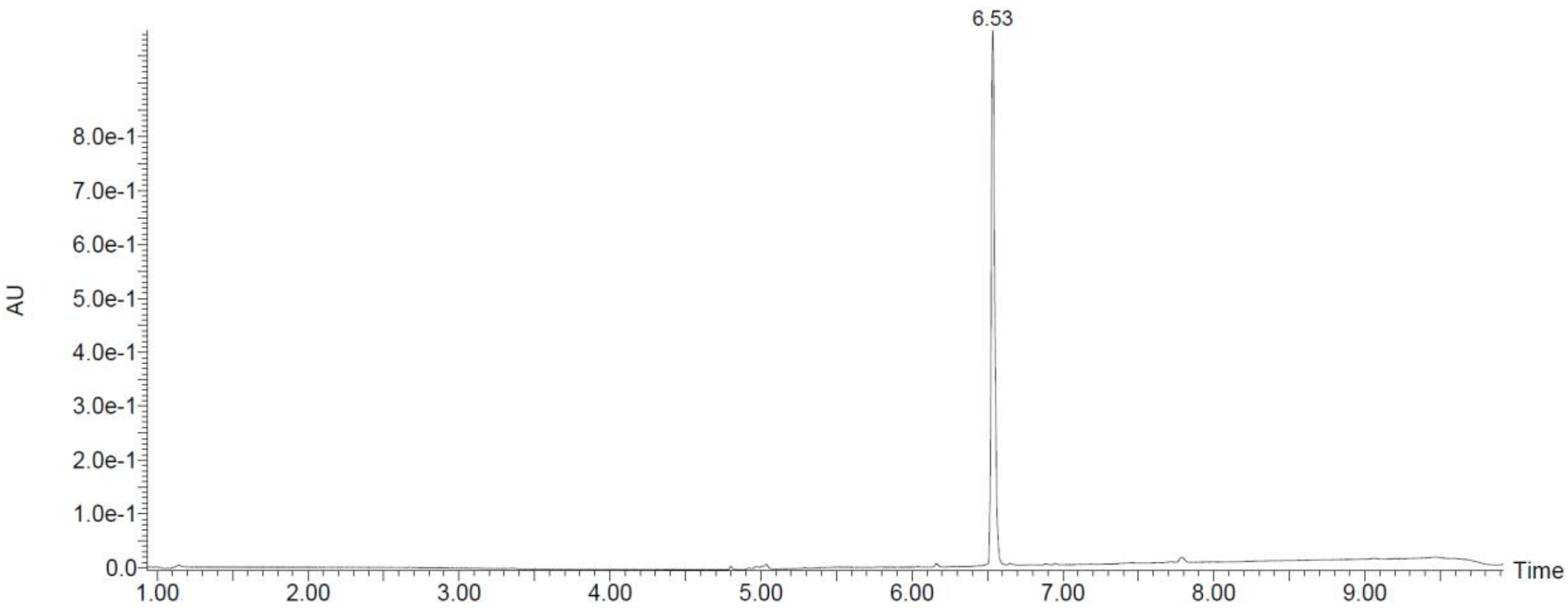

Mass spectrum (ES-) of BODIPY-FL NHS labelled modified oligonucleotide (y-axis = relative intensity [%], x-axis = deconvolved mass [Da]); calculated mass: 3715.08, found: 3714.7.

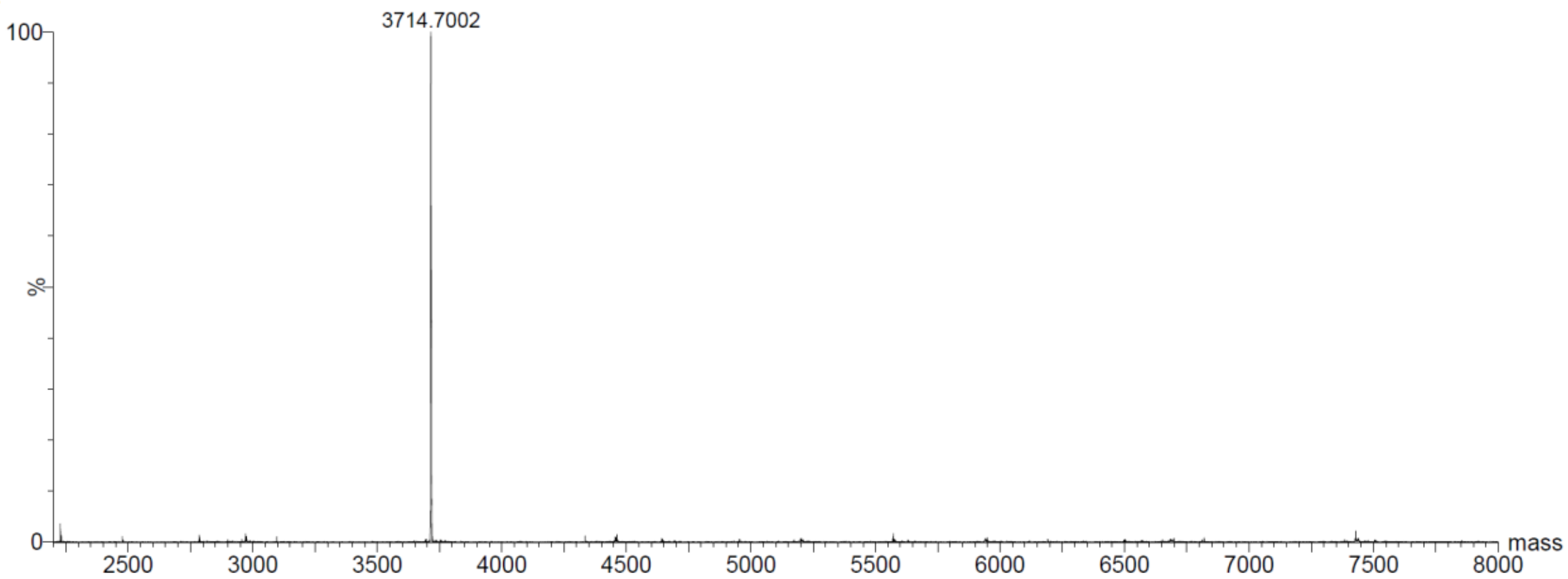

Ion-pair reversed-phase UHPLC chromatogram of the crude reaction mixture obtained from the sulfo-Cyanine3-labelled oligonucleotide (UV absorbance at 260 nm vs. time in minutes).

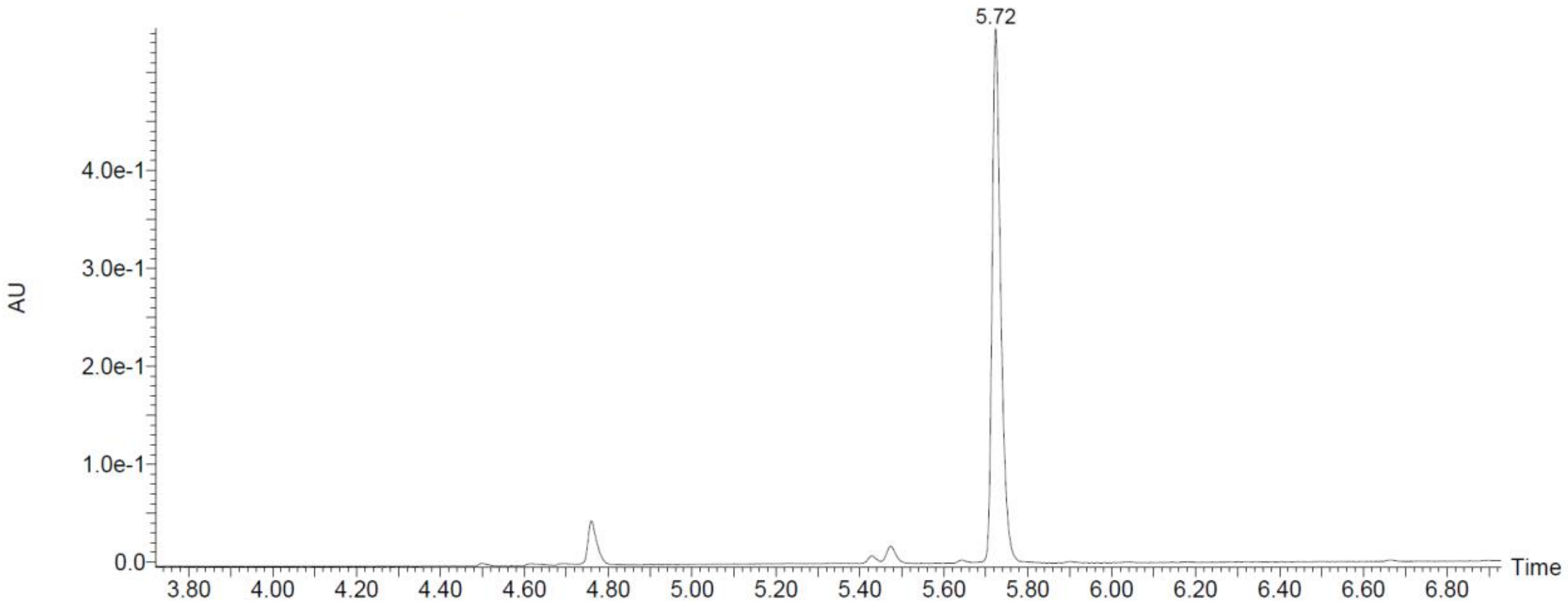

Mass spectrum (ES$^-$) of the sulfo-cyanine 3- labelled oligonucleotide (y-axis = relative intensity [%], x-axis = deconvolved mass [Da]); calculated mass: 4039.7, found: 4038.9.

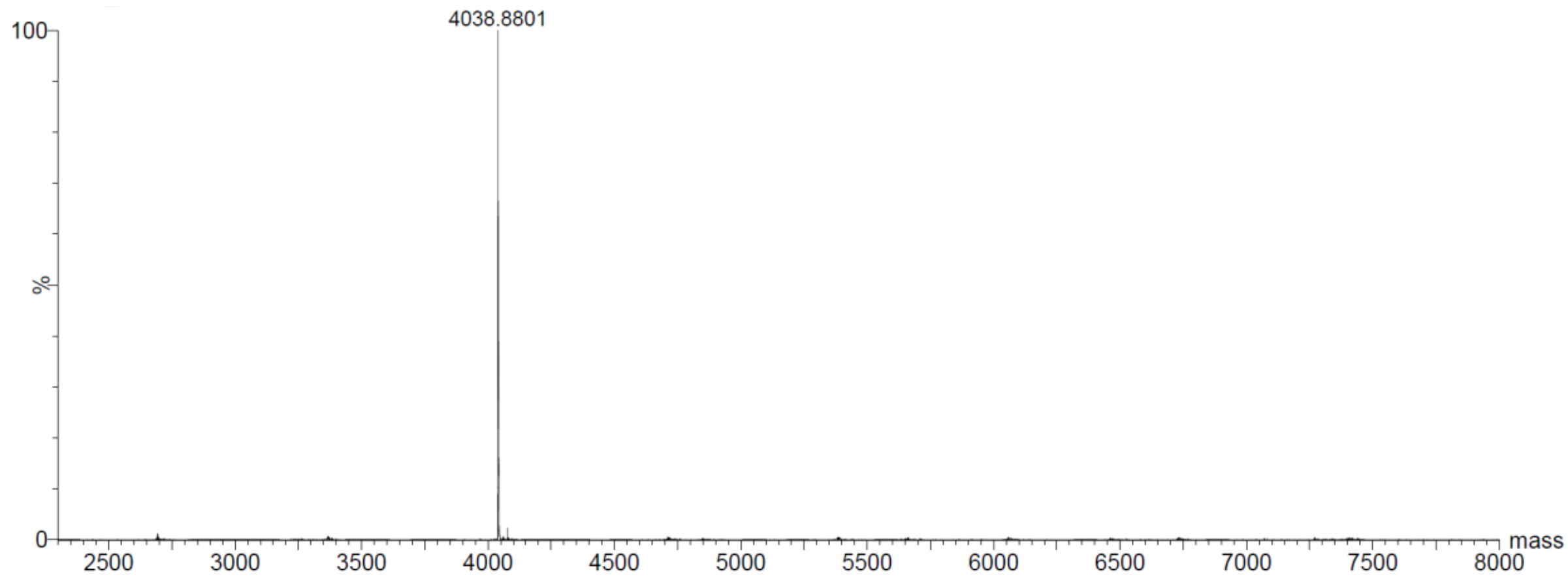

4.4 Characterization of cyanine 3 dye

Cyanine 3 dye was synthesized according to the literature report[1].

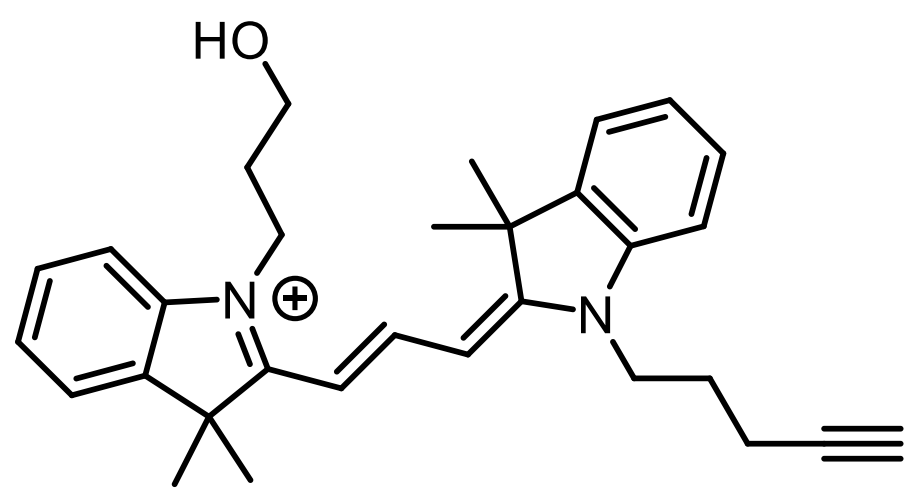

^1H NMR (500 MHz, DMSO-d$_6$ 298 K) δ 8.37 (t, *J* = 13.4 Hz, 1H), 7.65 (dd, *J* = 7.4, 4.0 Hz, 2H), 7.46 (m, 4H), 7.39–7.14 (m, 2H), 6.52 (m, 2H), 4.79 (t, *J* = 4.8 Hz, 1H), 4.19 (q, *J* = 7.2 Hz, 4H), 3.53 (q, *J* = 5.8 Hz, 2H), 2.96 (t, *J* = 2.6 Hz, 1H), 2.38–2.35 (m, 2H), 1.93 (m, 4H), 1.71 (s, 12H); ^{13}C NMR (126 MHz, DMSO-d$_6$ 298 K) δ 174.2, 173.9, 149.9, 141.9, 140.7, 140.6, 128.7, 128.6, 125.3, 125.2, 122.6, 122.5, 111.6, 111.2, 102.7, 102.4, 83.5, 72.2, 57.7, 49.0, 48.9, 42.8, 41.3, 30.1, 27.5, 27.39, 26.0, 15.4; IR $υ_{max}$ (cm^{-1}): 3585, 1555, 1427, 1355, 1230, 1153, 1045; HRMS (ESI+) $[M+H]^+$ $[C_{31}H_{38}N_2O]^+$: calculated 454.2979, found 454.2952.

^1H NMR (500 MHz, DMSO-d$_6$, 298 K) spectrum of Cy3 dye.

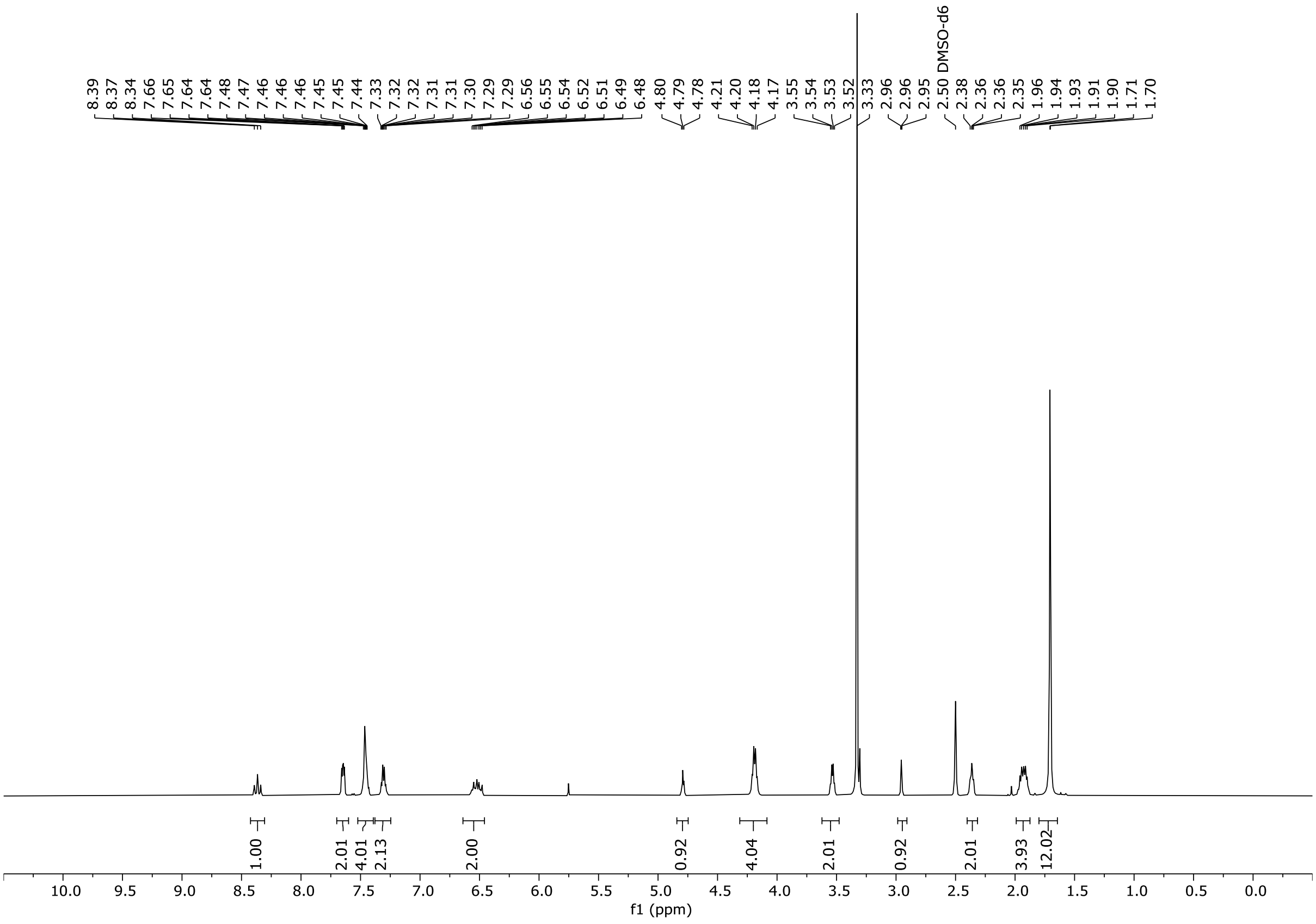

^{13}CNMR (126 MHz, DMSO-d_6, 298 K) spectrum of Cy3 dye.

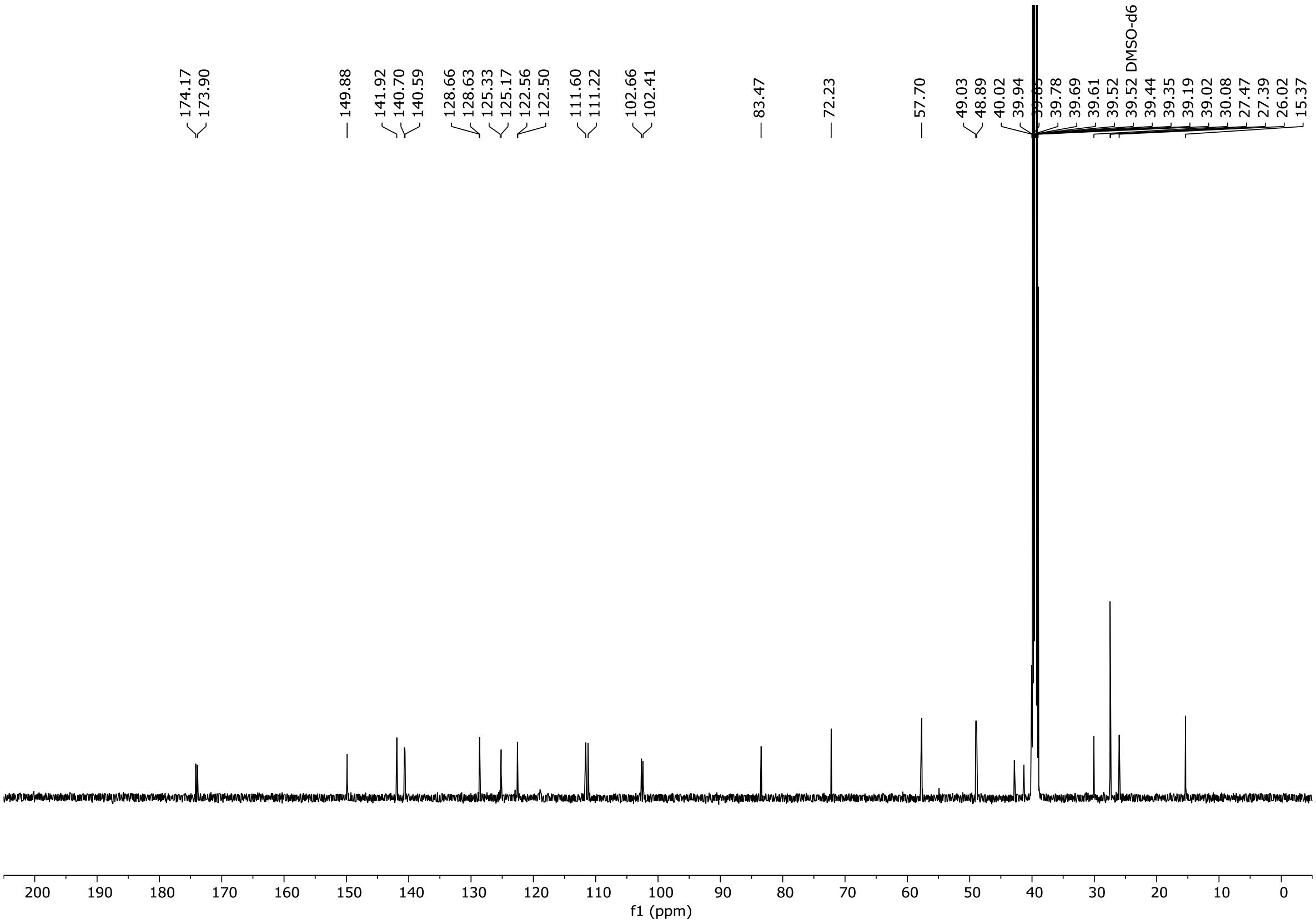

4.5 'Reverse' (5'→3') modified oligonucleotide synthesis of the $(dT)_{10}$ sequence

Sequence	rt	Area (A)	Extinction coefficient (ε)	A/ ε	Conversion
TTTTTTTTTT	5.144	1363.786	81600	0.0167131	
TTTTTTTTTT	5.302	1758.579	81600	0.021551213	
TTTTTTTTTT-**R**	6.863	23746.186	81600	0.291007181	88%

[R = $CH_3(CH_2)_5CH_2O$]

Ion-pair reversed-phase UHPLC chromatogram of the crude reaction mixture (UV absorbance at 260 nm vs. time in minutes).

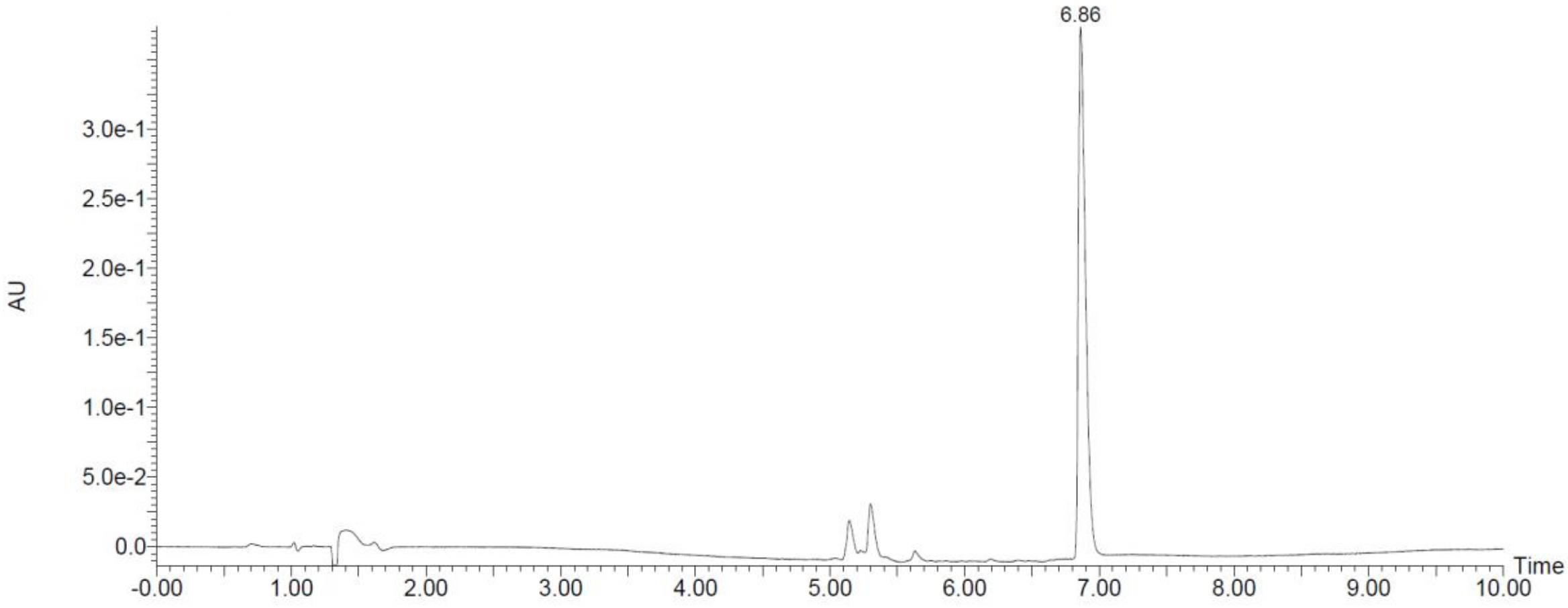

Mass spectrum of **5a** (ES-) of modified oligonucleotide (y-axis = relative intensity [%], x-axis = deconvolved mass [Da]); calculated mass: 3156.2, found: 3157.4.

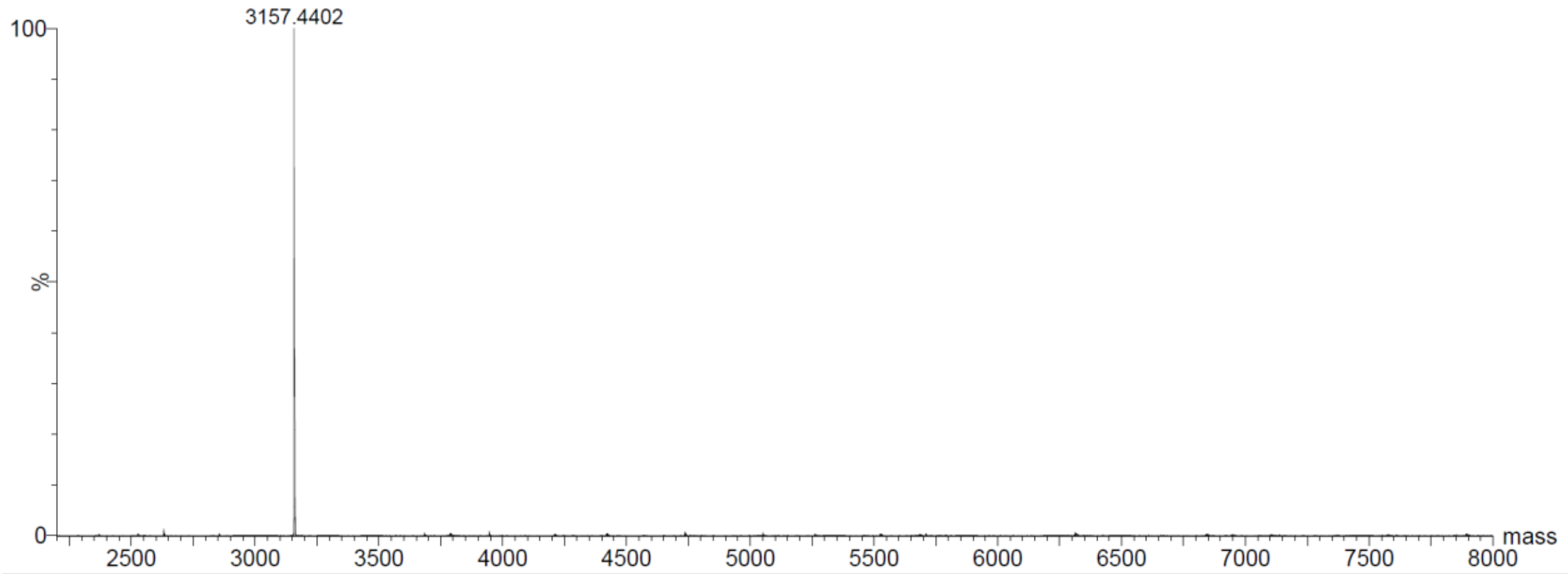

4.6 Sulfurization of the 3′-modified heptanol-coupled oligonucleotide

Sequence	rt	Area (A)	Extinction coefficient (ε)	A/ ε	Conversion
TTTTTTTTTT	4.302	1851.655	81600	0.02269185	
TTTTTTTTTT	4.406	6833.863	81600	0.083748321	
TTTTTTTTTT	4.490	3033.014	81600	0.037169289	
TTTTTTTTTT	4.575	4092.863	81600	0.050157635	
TTTTTTTTTT-**R**	5.970	85774.211	81600	1.051154547	84%

[R = $CH_3(CH_2)_6O$]

Ion-pair reversed-phase UHPLC chromatogram of the crude reaction mixture (UV absorbance at 260 nm vs. time in minutes).

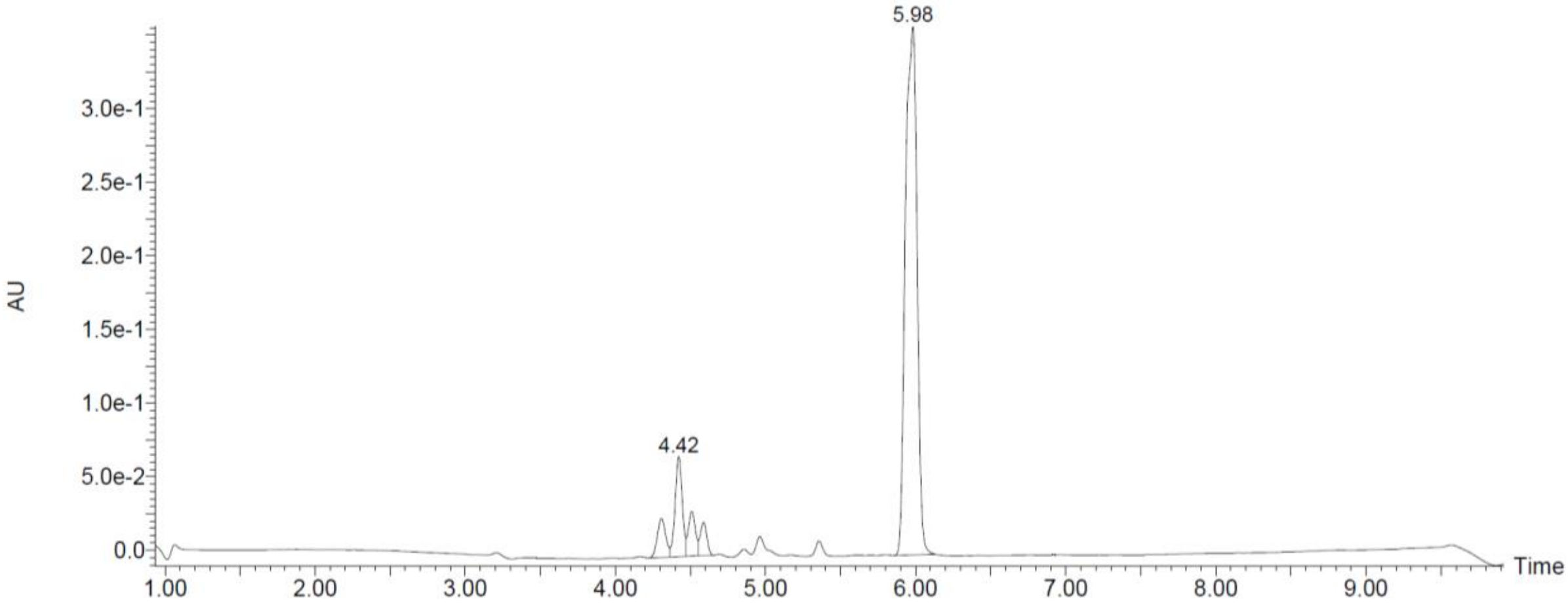

Mass spectrum of **5a'** (ES-) (y-axis = relative intensity [%], x-axis = deconvolved mass [Da]); calculated mass: 3174.2, found: 3174.4.

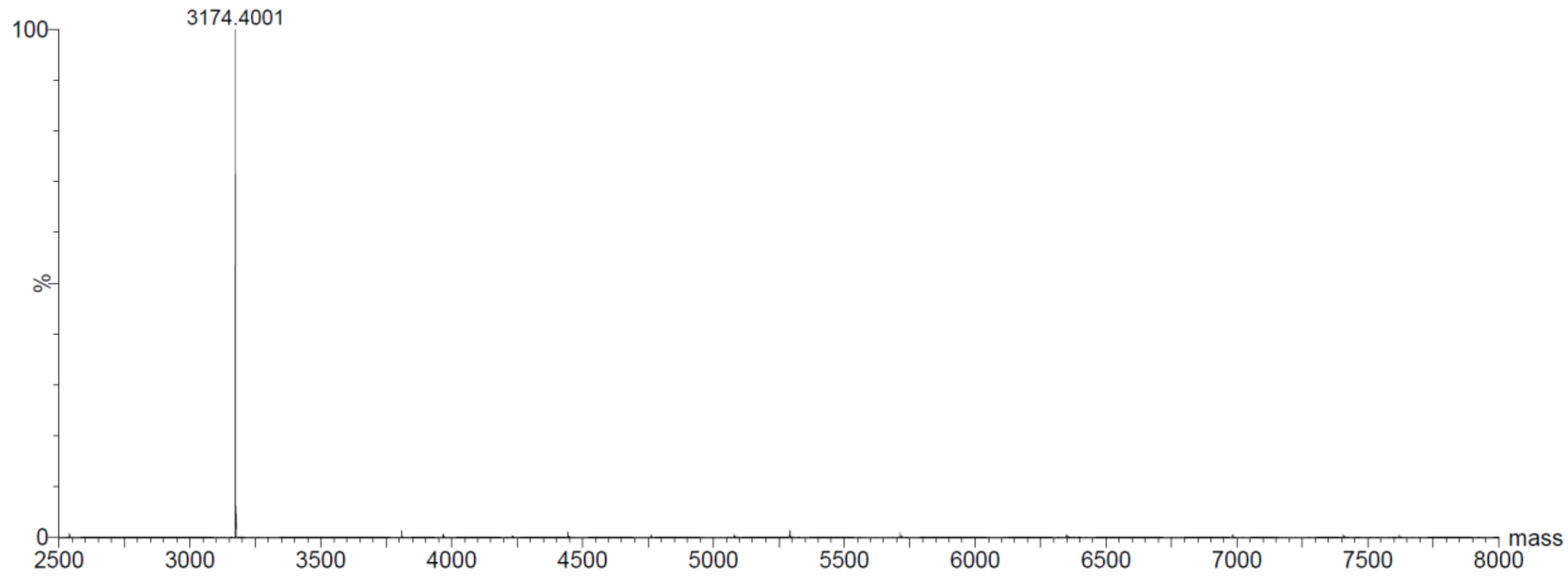

4.6 Oligonucleotide phosphoramidate (**8**) synthesis

A 0.3 M solution of the phosphitylating reagent [P–N] in MeCN was prepared prior to the reaction. Commercial 5-benzylthio-1*H*-tetrazole (BTT) solution (0.3 M in MeCN) was used as the activator for the phosphitylation step. The phosphitylating solution (0.9 mL) and the BTT solution (0.9 mL) were loaded into separate 2 mL syringes and passed back and forth through the synthesis column for 10 minutes. All reactions were performed at ambient temperature under an argon atmosphere. Following the coupling step, the resin was washed with 1 mL of MeCN, flushed with argon, and treated with 0.02 M iodine solution (Sigma-Aldrich, product number L060020) for 3 minutes at ambient temperature to oxidize P(III) to P(V). Global deprotection was carried out using an AMA [35% NH_3 (aq.) and 40% methyl amine (aq.) 1:1., v/v] solution at 65 ^oC for 10 min, The solution was concentrated using a rotary evaporator, and the resulting crude product was dissolved in 1 mL of deionised water to prepare the stock solution.

Sequence	rt	Area (A)	Extinction coefficient (ε)	A/ε	Conversion
SSQVYSPRP	4.53	1358.419	110270	0.01231903	
SSQVYSPRP	4.68	4313.024	110270	0.0391133	
SSQVYSPRP	5.383	30594.764	110270	0.2774532	84%

Ion-pair reversed-phase UHPLC chromatogram of the crude reaction (UV absorbance at 260 nm vs. time in minutes).

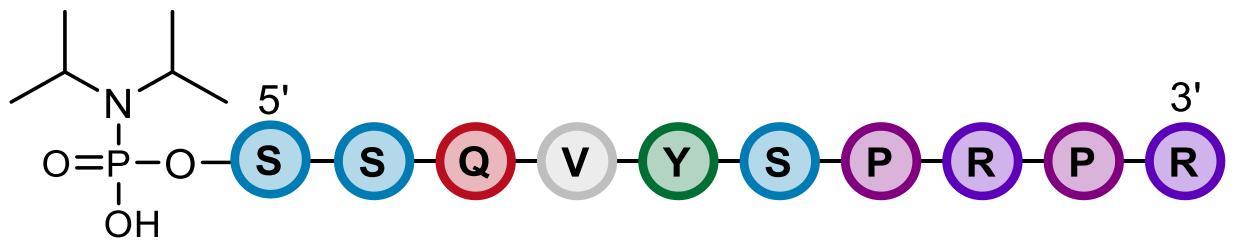

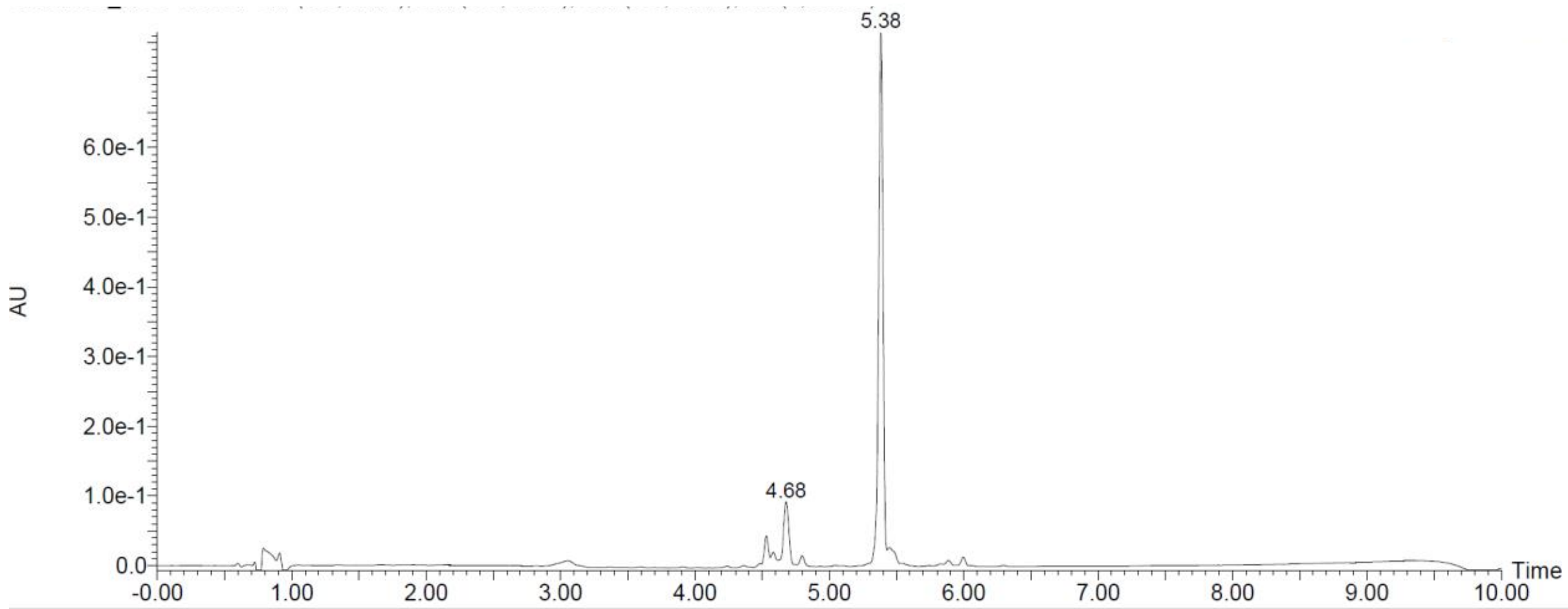

Mass spectrum (ES^{-}) of **8** (y-axis = relative intensity [%], x-axis = deconvolved mass [Da]); calculated mass: 3425.2, found: 3425.4.

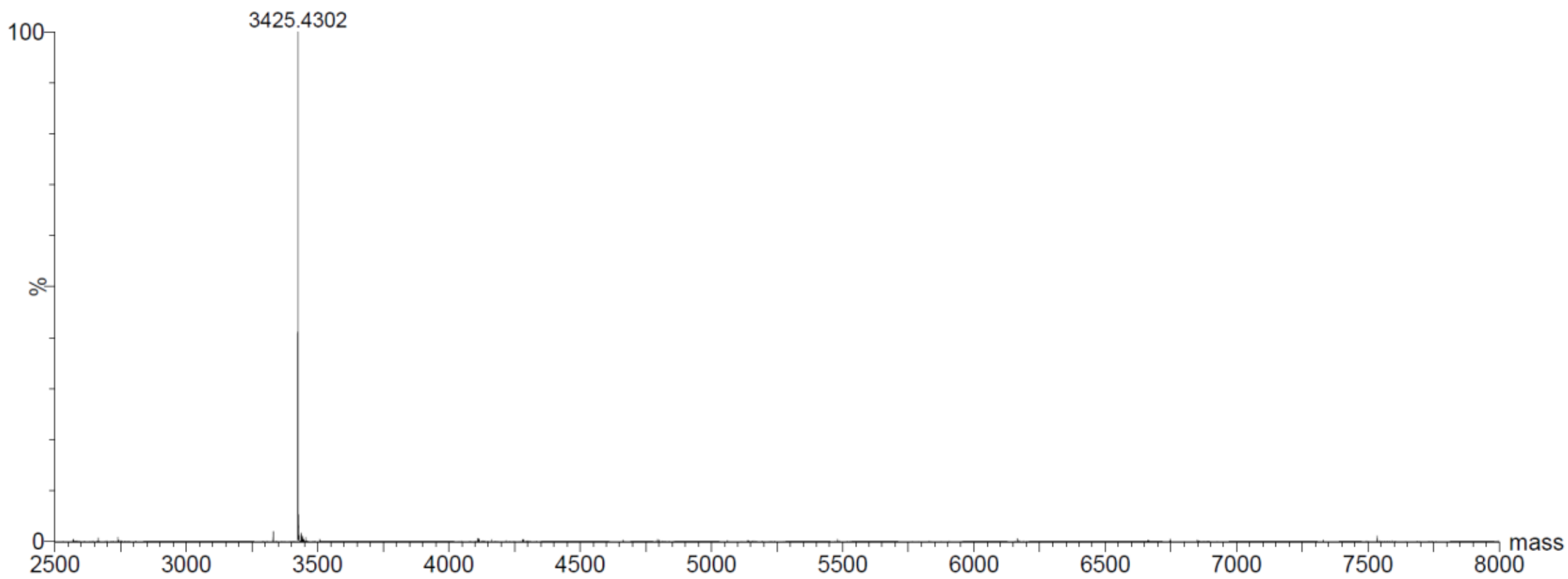

5. Internal modifications

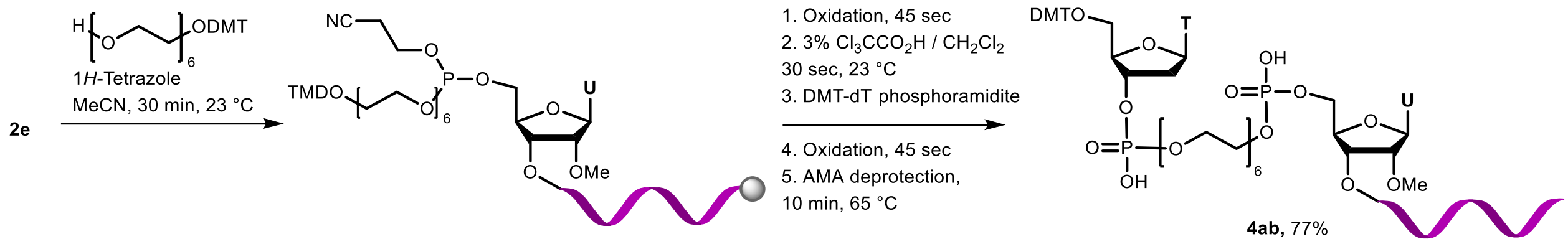

Sequence	rt	Area (A)	Extinction coefficient (ε)	A/ε	Conversion
SSQVYSPRPR	4.528	13828.692	118670	0.11653065	
SSQVYSPRPR	4.903	2853.852	118670	0.02404864	
SSQVYSPRPR	4.975	4839.565	118670	0.04078171	
T-R-SSQVYSPRPR	7.668	69925.945	118670	0.58924703	77%

[R = DMTO-PEG_6-O]

Ion-pair reversed-phase UHPLC chromatogram of the crude reaction mixture (UV absorbance at 260 nm vs. time in minutes).

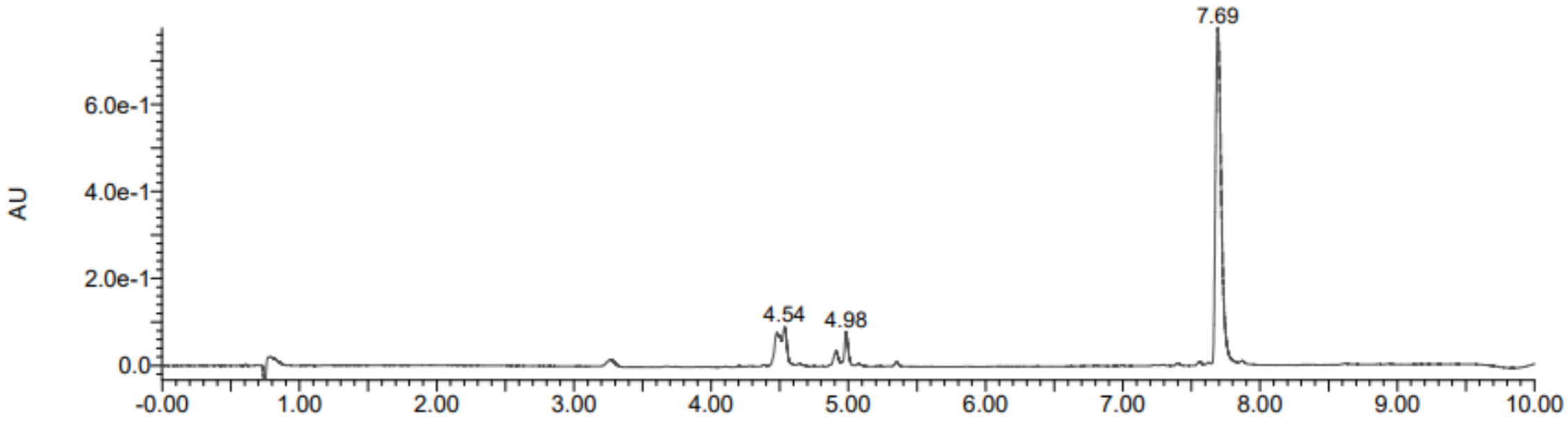

Mass spectrum (ES-) of **4ab** (y-axis = relative intensity [%], x-axis = deconvolved mass [Da]); calculated mass: 4212.8, found: 4212.8.

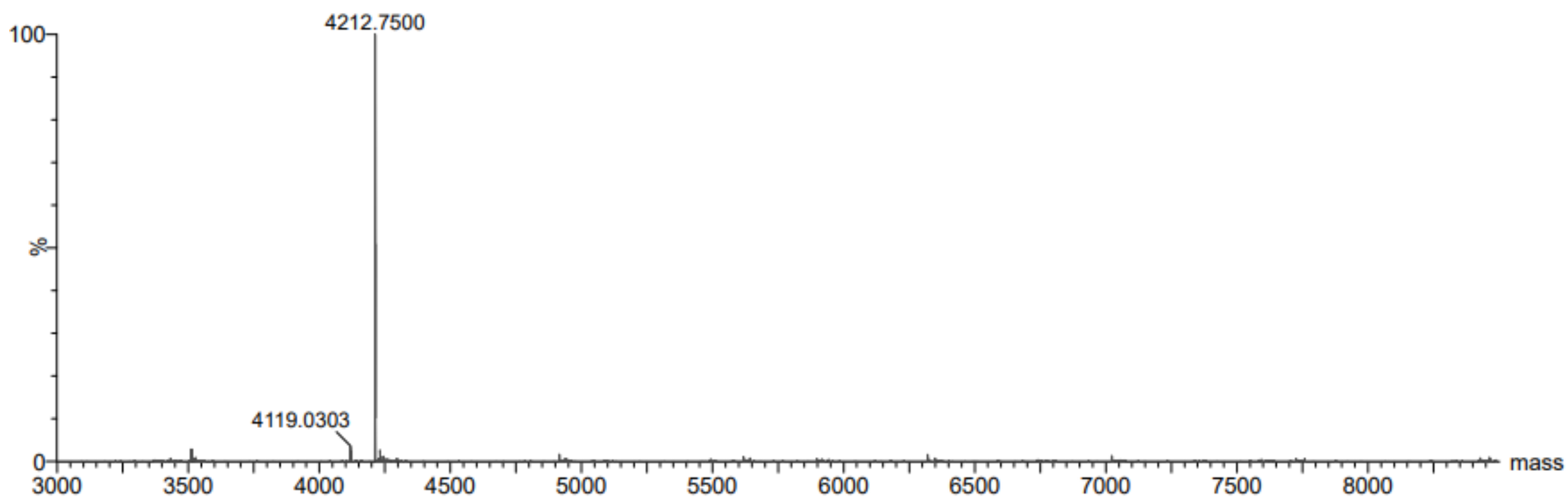

6. Nucleoside Coupling

6.1 Nucleoside (3'-OH) coupling with 5'-OH of (dT)10 sequence on CPG solid support

Sequence	rt	Area (A)	Extinction coefficient (ε)	A/ε	Conversion
TTTTTTTTTT	4.449	733.317	81600	0.008987	
TTTTTTTTTT	4.508	713.733	81600	0.008747	
T-TTTTTTTTTT	4.546	1301.905	81600	0.015955	
	7.013	6816.649	89700	0.075994	69%

Ion-pair reversed-phase UHPLC chromatogram of the crude reaction mixture (UV absorbance at 260 nm vs. time in minutes).

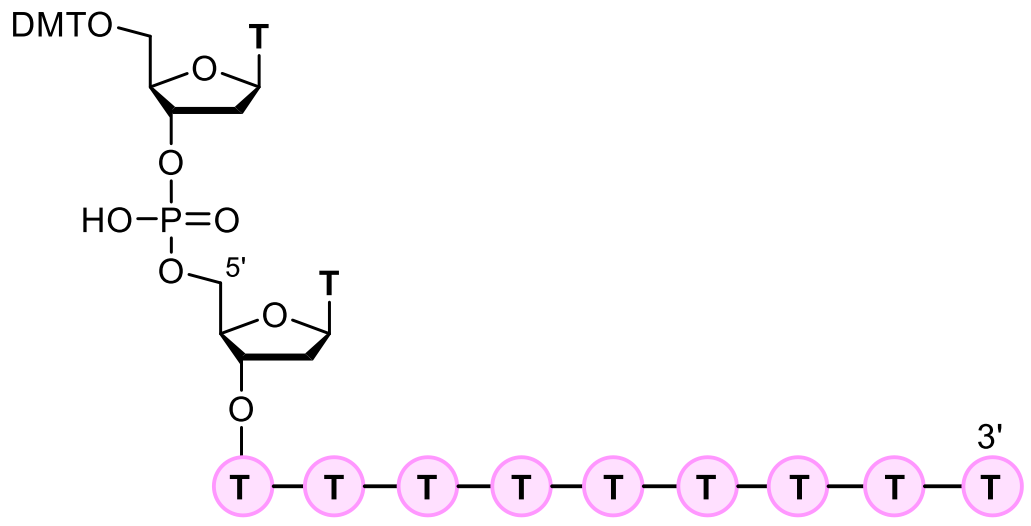

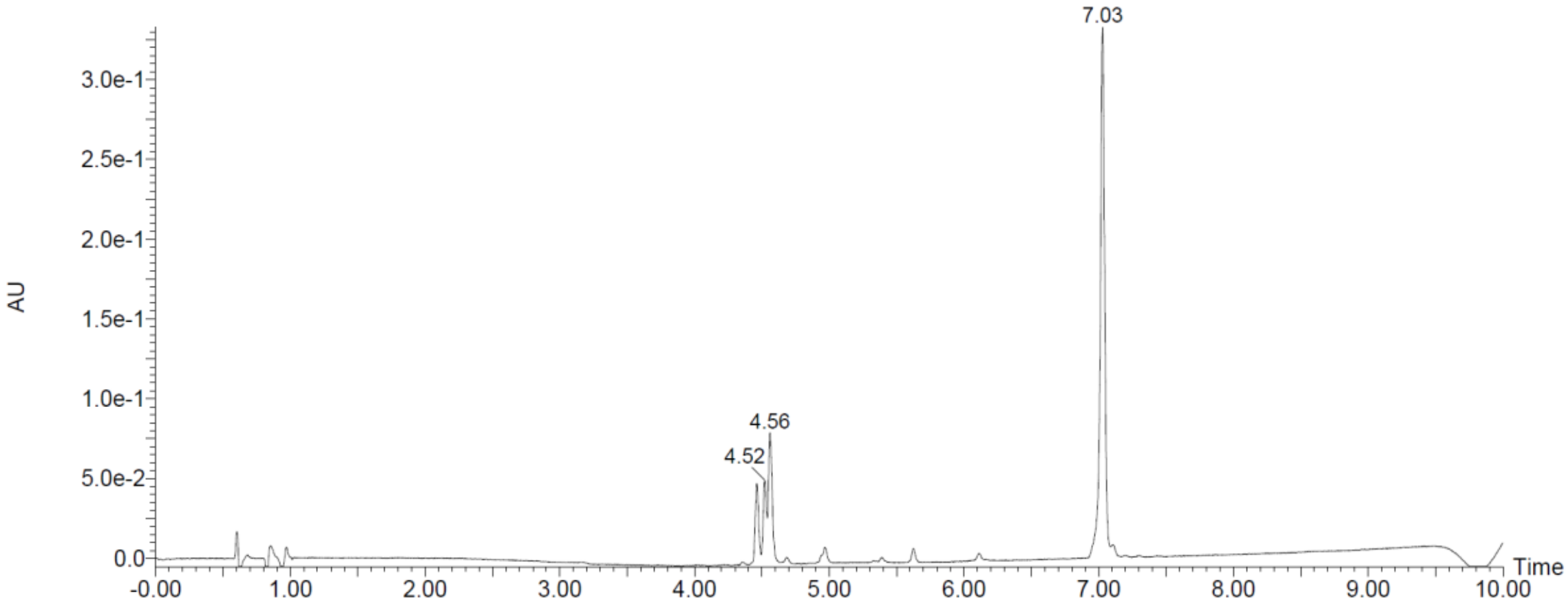

Mass spectrum of **4ac** (ES-) (y-axis = relative intensity [%], x-axis = deconvolved mass [Da]); calculated mass: 3586.6, found: 3585.7.

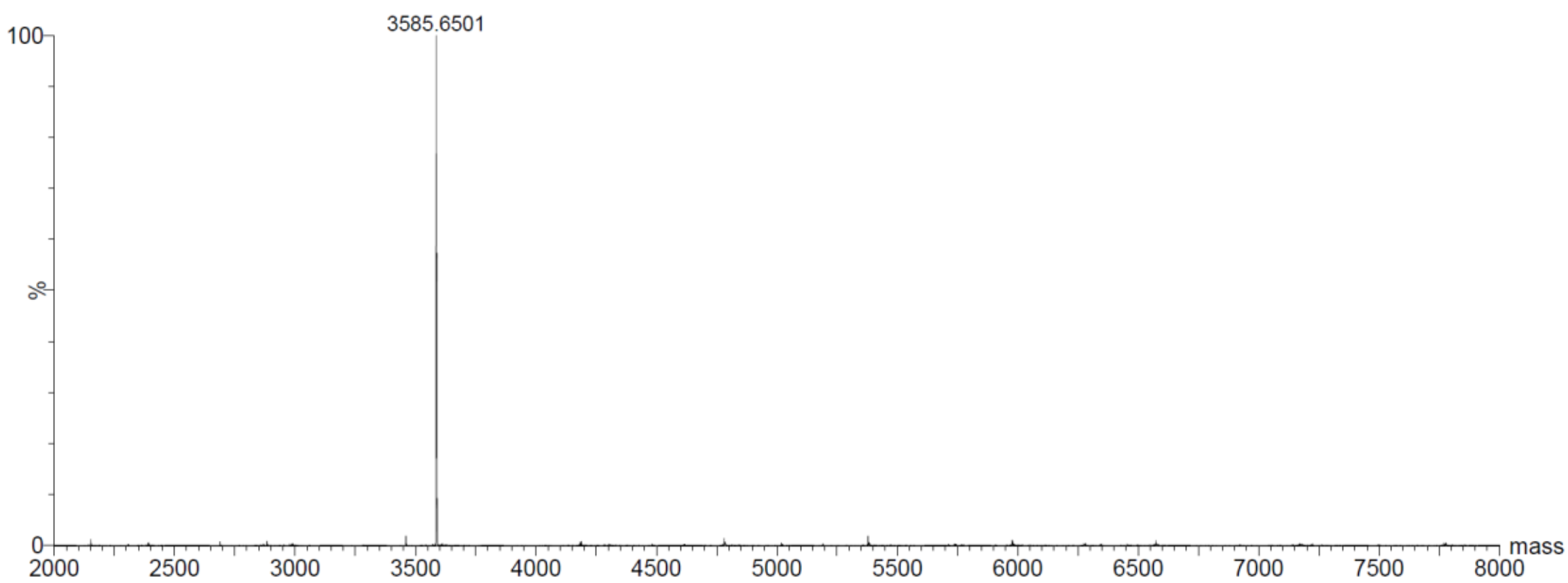

6.2 Nucleoside (3'-OH) coupling with 5'-OH of SSQVYSPRPR sequence on PS solid support

Sequence	rt	Area (A)	Extinction coefficient (ε)	A/ε	Conversion
SSQVYSPRP	4.718	447.007	110270	0.004054	
SSQVYSPRP	4.81	3156.274	110270	0.028623	
T-SSQVYSPRP	7.312	17170.002	118670	0.144687	82%

Ion-pair reversed-phase UHPLC chromatogram of the crude reaction mixture (UV absorbance at 260 nm vs. time in minutes).

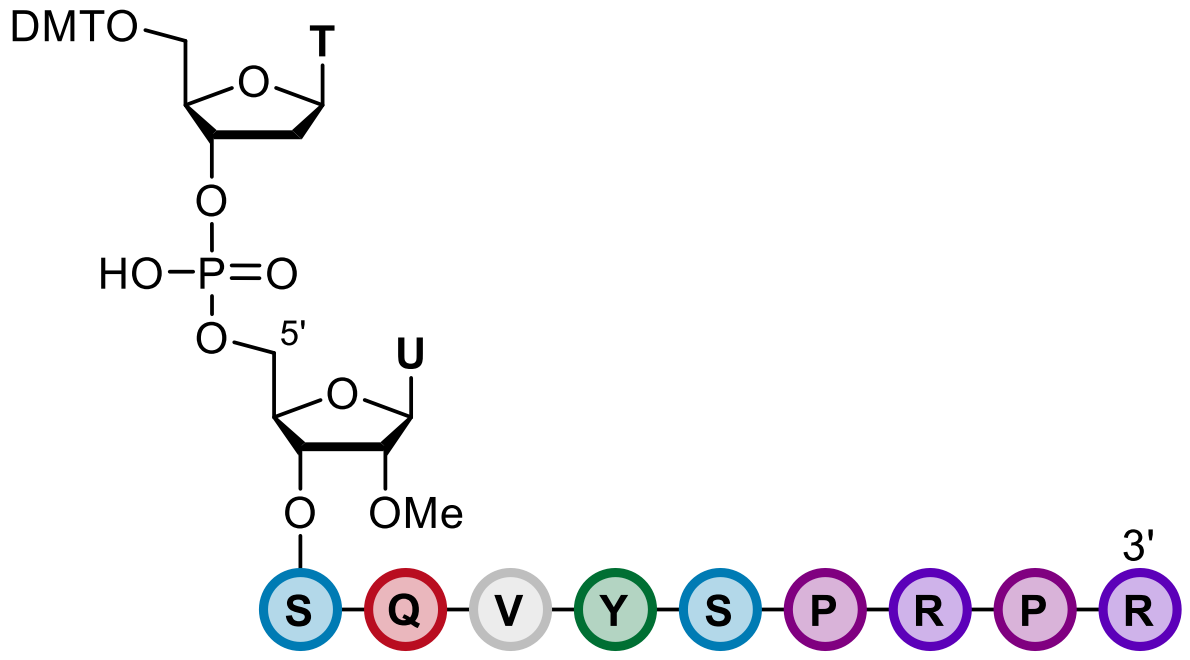

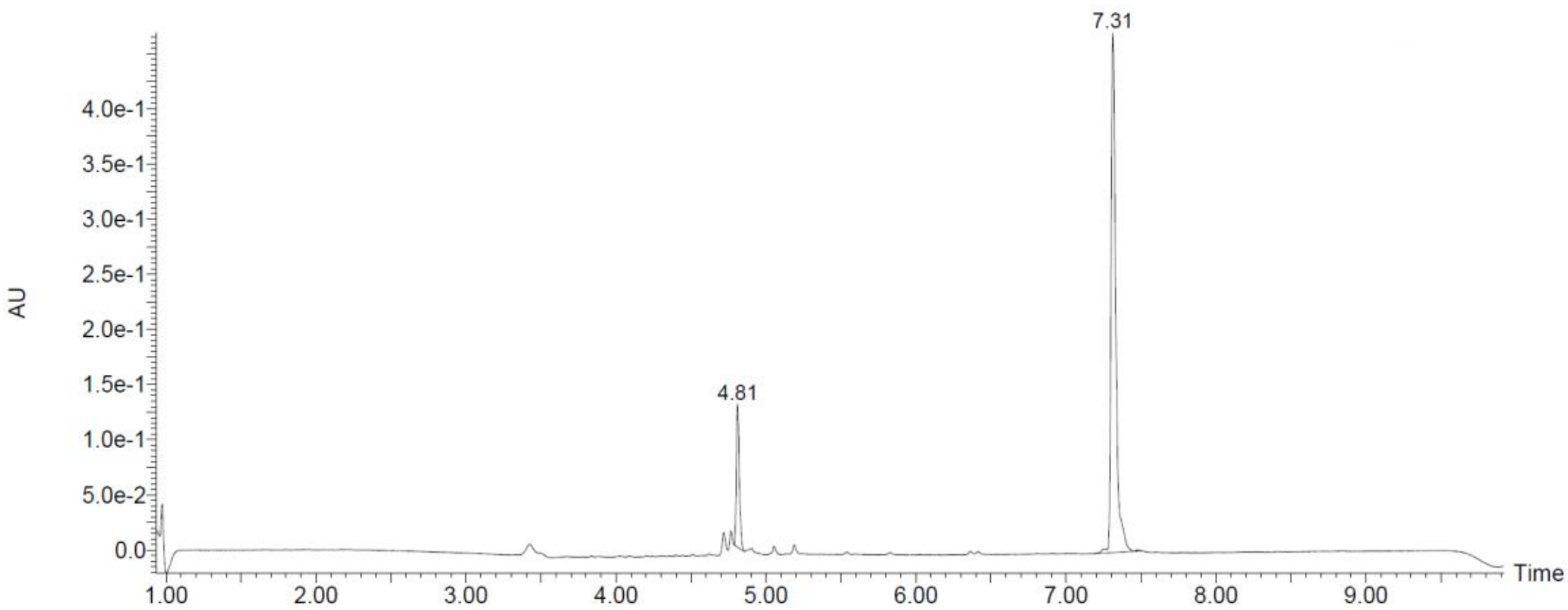

Mass spectrum of **4ad** (ES-) (y-axis = relative intensity [%], x-axis = deconvolved mass [Da]); calculated mass: 3868.6, found: 3867.4.

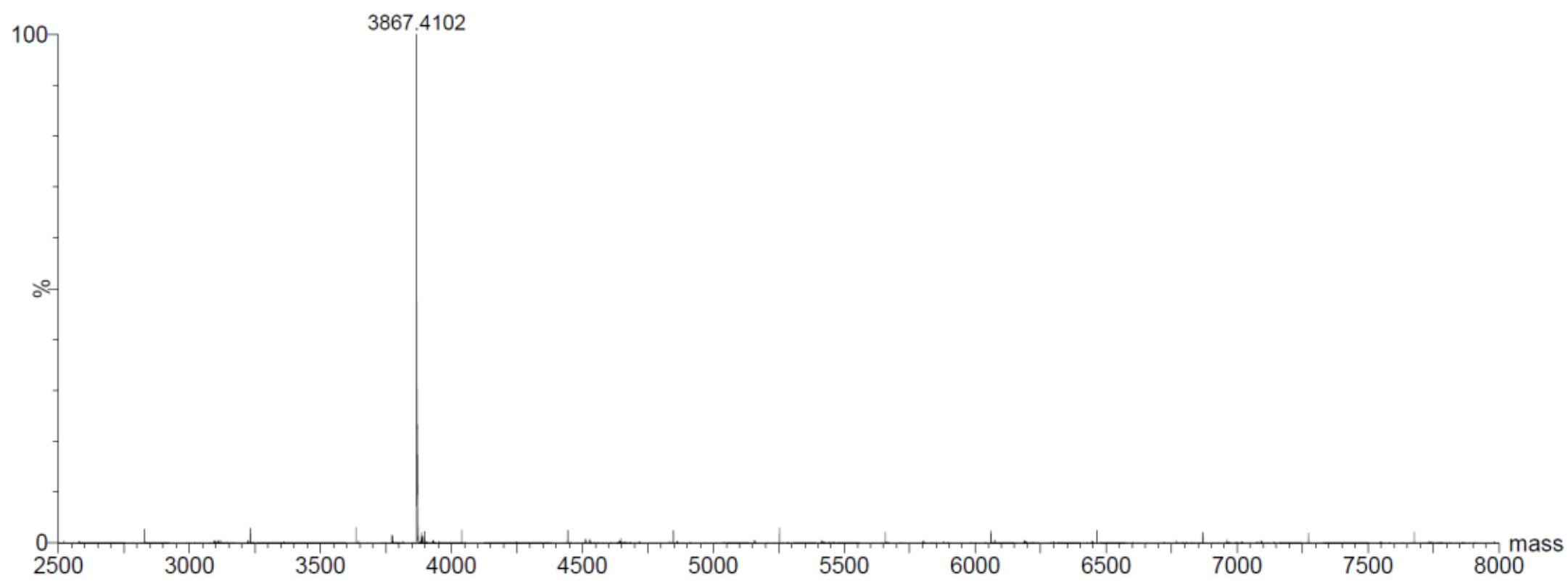

6.3 Nucleoside (5'-OH) coupling with 3'-OH of reverse $(dT)_{10}$ sequence on CPG solid support

Sequence	rt	Area (A)	Extinction coefficient (ε)	A/ε	Conversion
TTTTTTTTTT	5.119	1739.235	81600	0.021314	
TTTTTTTTTTT	5.277	5258.57	81600	0.064443	
TTTTTTTTTT-**T(3'*O*-TBS)**	7.441	19440.05	89700	0.216723	72%

Ion-pair reversed-phase UHPLC chromatogram of the crude reaction mixture (UV absorbance at 260 nm vs. time in minutes).

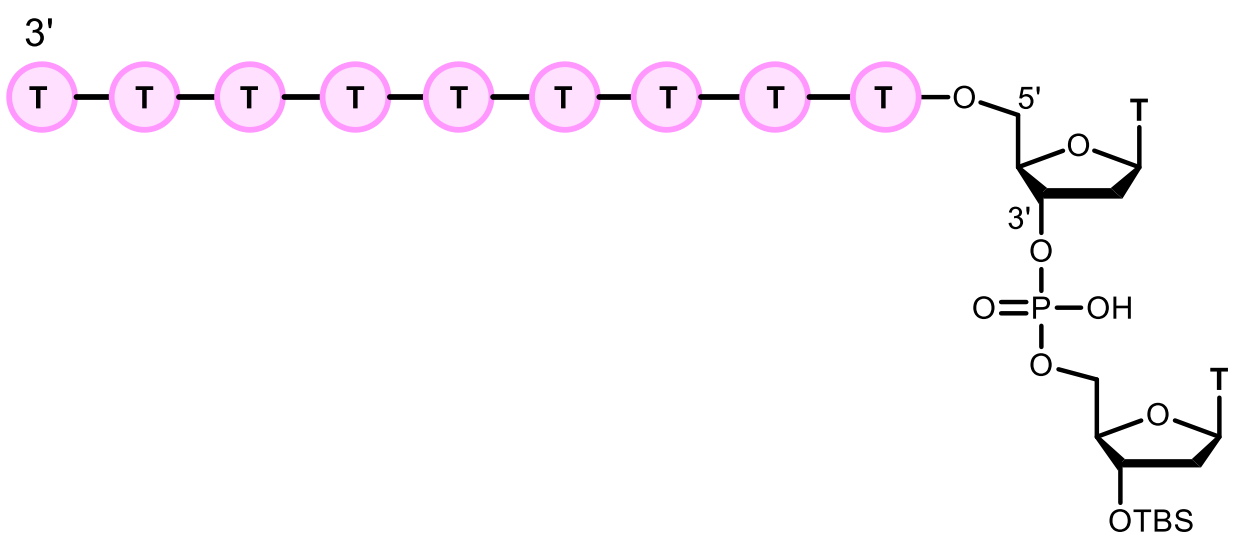

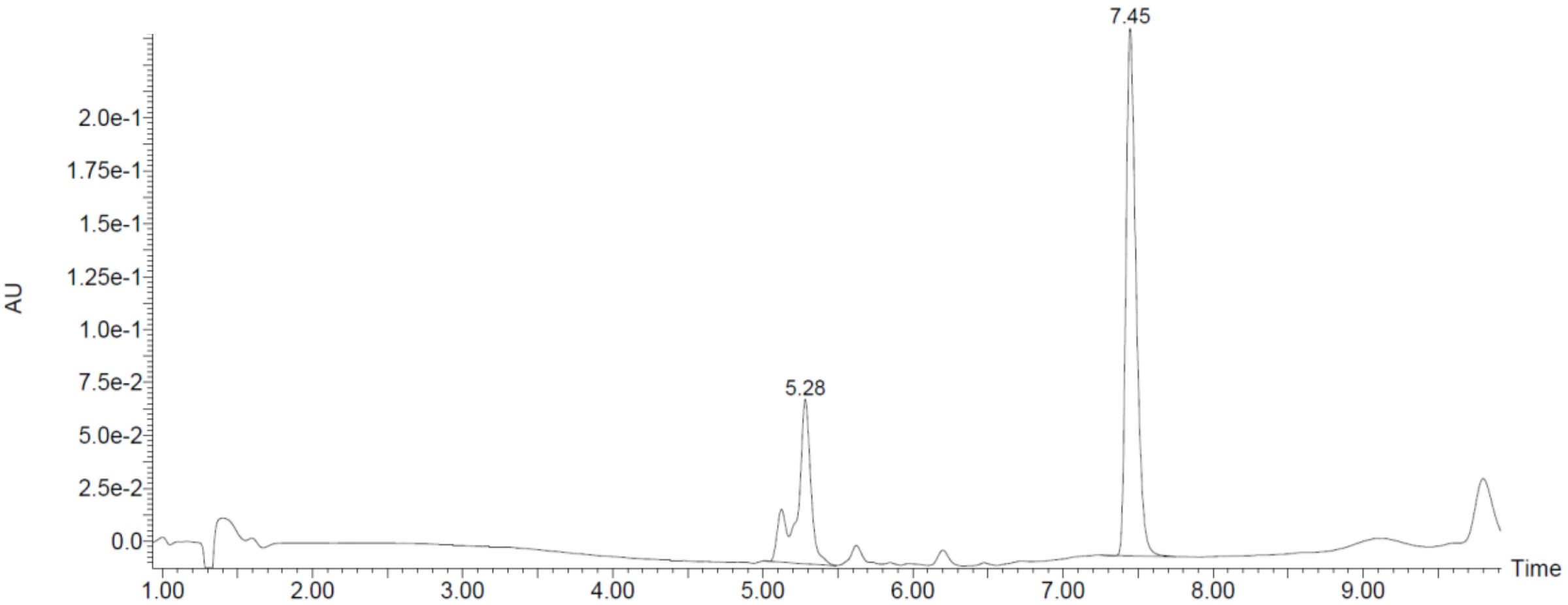

Mass spectrum of **5b** (ES-) (y-axis = relative intensity [%], x-axis = deconvolved mass [Da]); calculated mass: 3397.4, found: 3397.7.

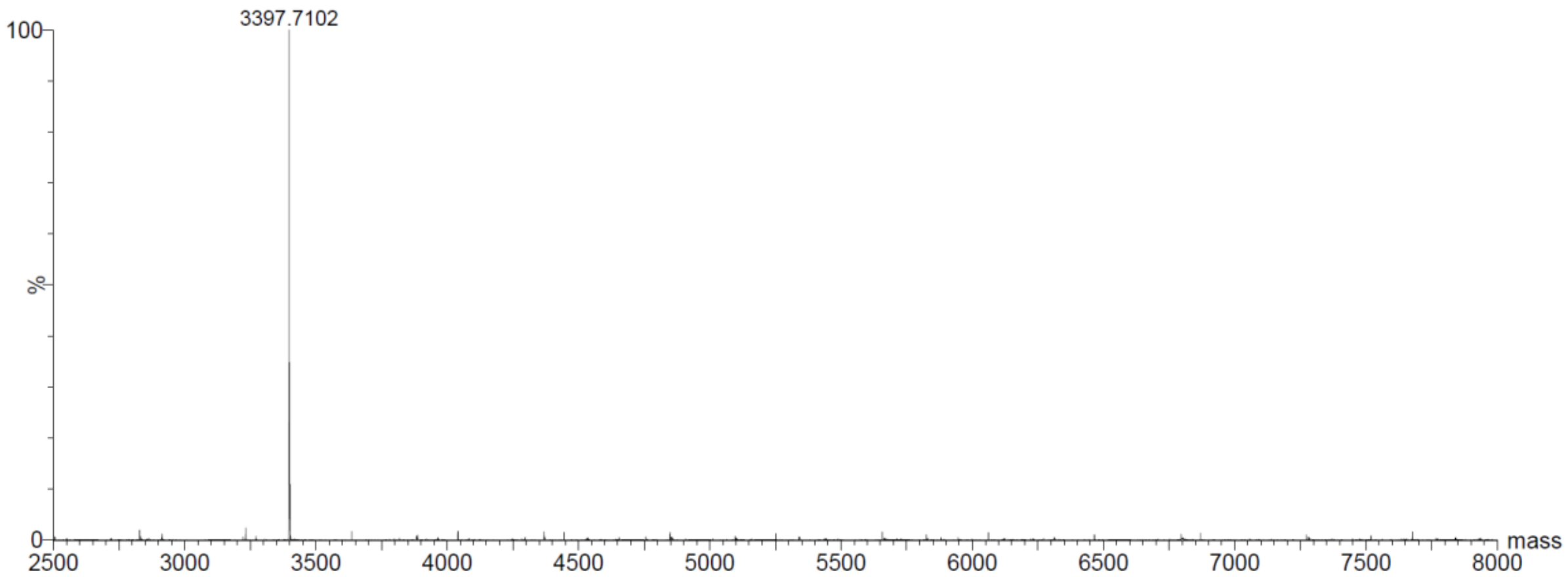

6.4 Nucleoside (5'-OH) coupling with 5'-OH of mixed SSQVYSPRPR sequence on PS solid support

Sequence	rt	Area (A)	Extinction coefficient (ε)	A/ε	Conversion
SSQVYSPRPR	4.696	3066.127	110270	0.027805632	
SSQVYSPRPR	4.776	14433.757	110270	0.130894686	
**(2'*O*-TBS)T-** SSQVYSPRPR	6.56	36953.50	118670	0.311397219	70%

Ion-pair reversed-phase UHPLC chromatogram of the crude reaction mixture (UV absorbance at 260 nm vs. time in minutes).

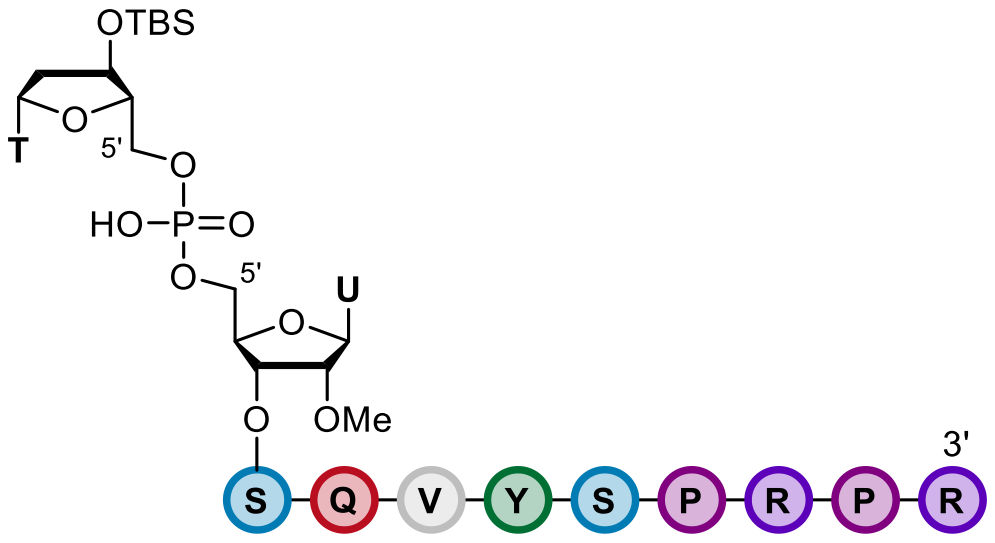

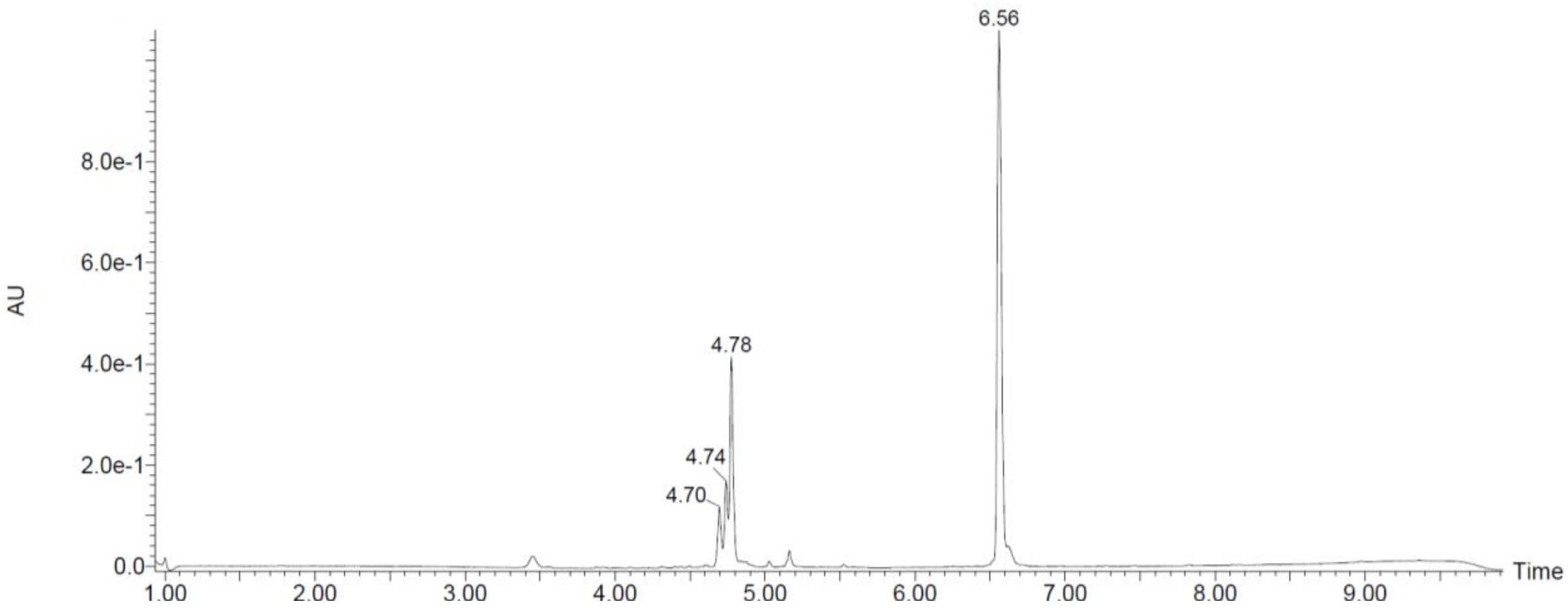

Mass spectrum of **4ae** (ES-) (y-axis = relative intensity [%], x-axis = deconvolved mass [Da]); calculated mass: 3679.5, found: 3679.2.

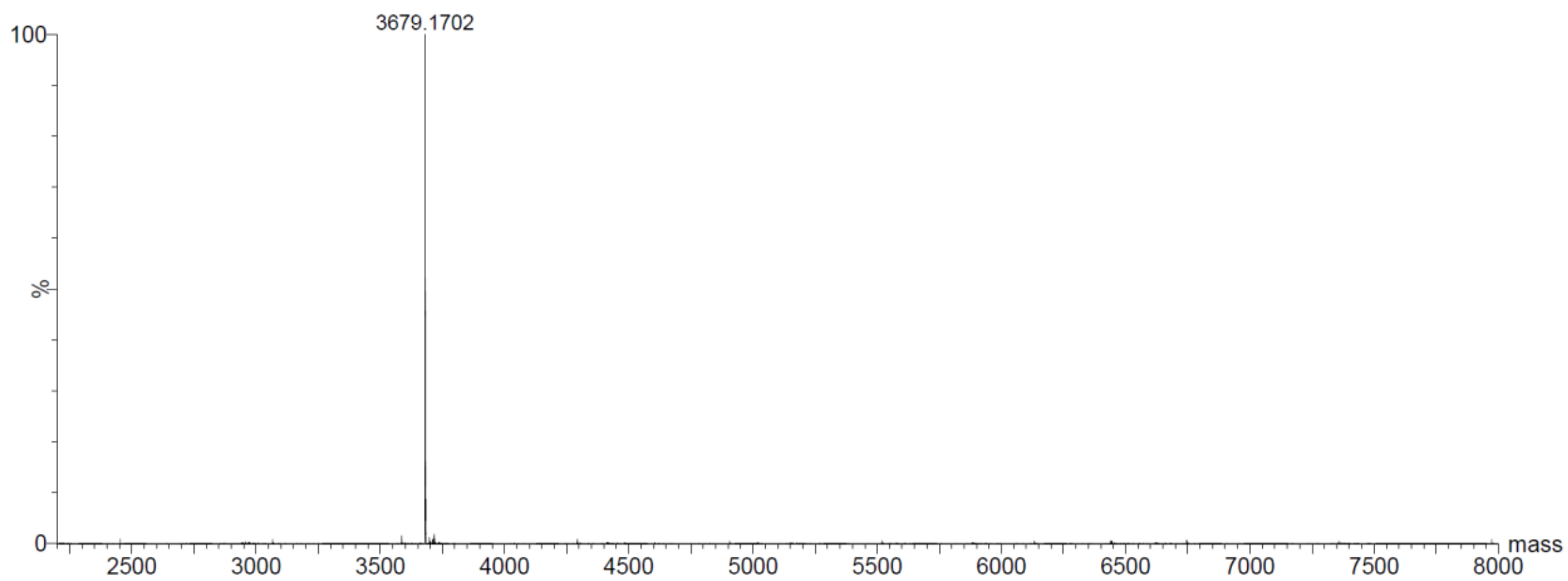

7. **Recovery of the DMT-dT nucleoside**

After the DMT-dT coupling reaction, the mixture of tetrazole and DMT-dT solutions was removed from the syringe. The synthesis column was washed with dry MeCN (2 × 1 mL), and the combined organic fractions were diluted with 5 mL of CH_2Cl_2. The organic layer was washed twice with 5 mL of 5 M aqueous Na_2CO_3, followed by a wash with saturated brine solution (5 mL). The organic phase was then dried over $MgSO_4$, filtered, and concentrated to afford pure DMT-dT in 92% recovery.

^{1}H NMR (500 MHz, DMSO-d$_6$, 298 K) δ 11.33 (s, 1H), 7.51 (d, *J* = 1.4 Hz, 1H), 7.39 (d, *J* = 7.3 Hz, 2H), 7.31 (t, *J* = 7.6 Hz, 2H), 7.25 (t, *J* = 8.0 Hz, 5H), 6.89 (dd, *J* = 8.9, 1.9 Hz, 4H), 6.21 (t, *J* = 6.8 Hz, 1H), 5.34 (d, *J* = 4.5 Hz, 1H), 4.32 (dd, *J* = 6.9, 3.6 Hz, 1H), 3.88 (q, *J* = 3.6 Hz, 1H), 3.73 (s, 6H), 3.19 (qd, *J* = 10.5, 3.8 Hz, 2H), 2.32–2.08 (m, 2H), 1.50–1.23 (s, 3H).

^{1}H NMR (500 MHz, DMSO-d$_6$, 298 K) spectrum of recovered DMT-d(T).

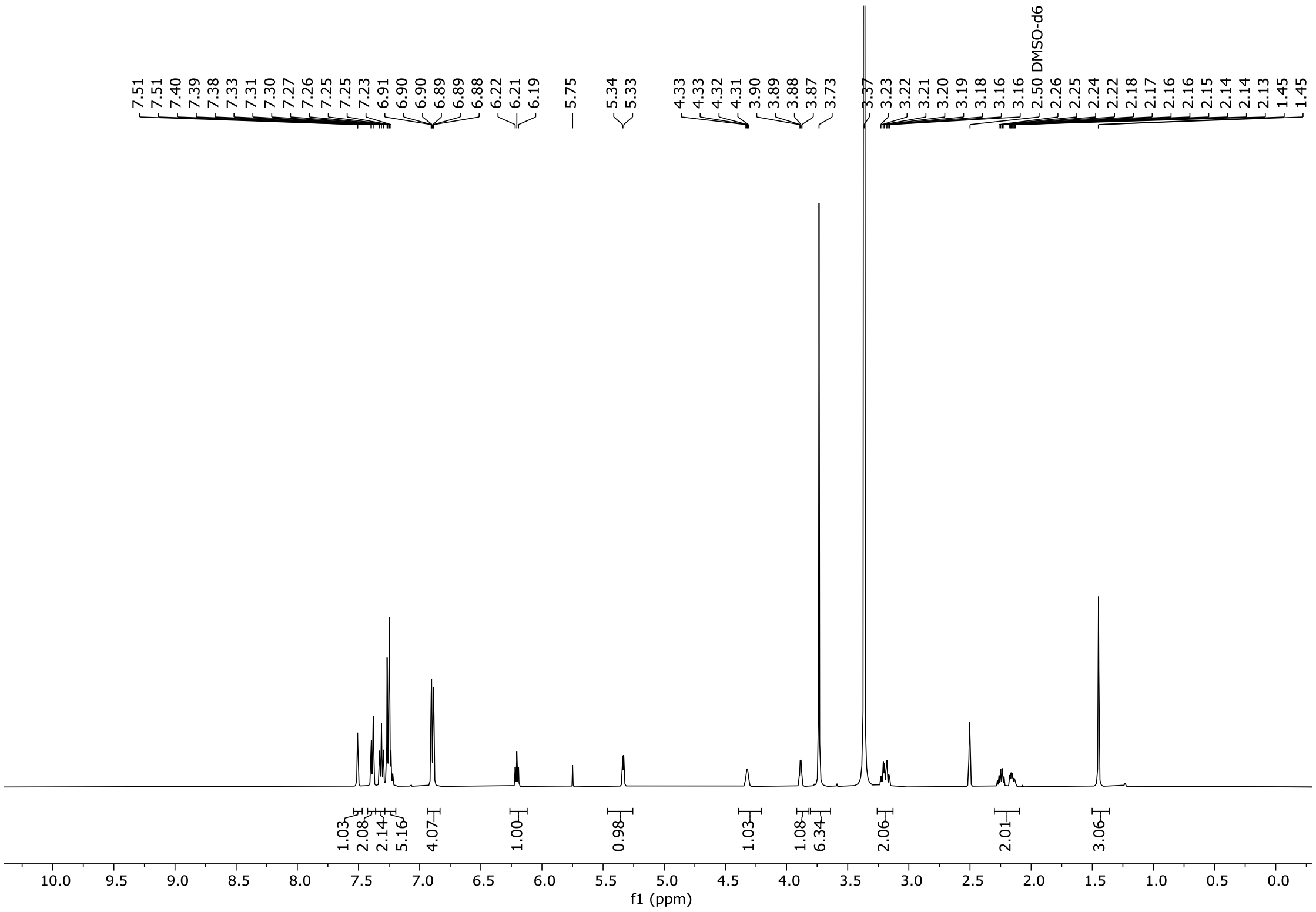

Stacked ^{1}H NMR (500 MHz, DMSO-d_6, 298 K) spectrum of tetrazole (top), recovered DMT-dT (middle) and pure DMT-dT (bottom)

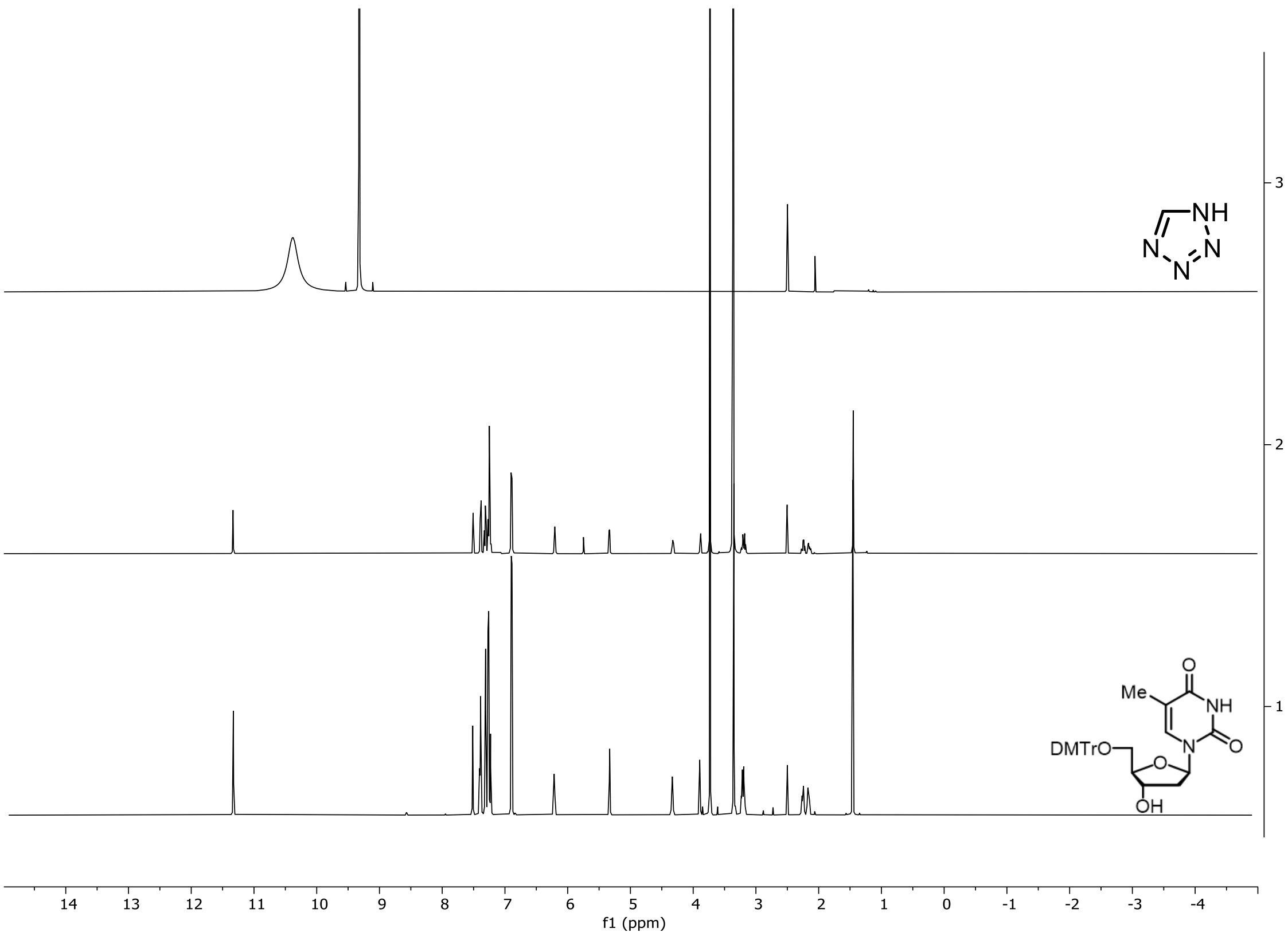

8. Phosphotriester backbone synthesis

8.1 Spectral characterization of the phosphonamidites **6a** and **6b**

All listed phosphoramidites *N*,*N*,*N'*,*N'*-tetraisopropyl-1-((tetrahydro-2*H*-pyran-4-yl)oxy)phosphanediamine (**6a**), and 1-isopropoxy-*N*,*N*,*N'*,*N'*-tetraisopropyl phosphanediamine (**6b**), and were synthesized according to literature procedures[2] and employed in the synthesis of modified oligonucleotides with a phosphotriester backbone.

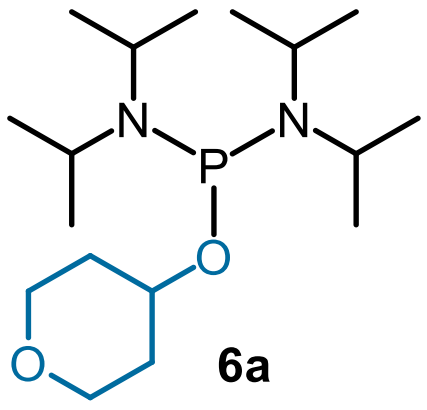

6a

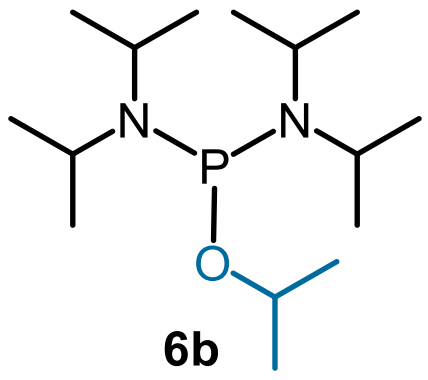

6b

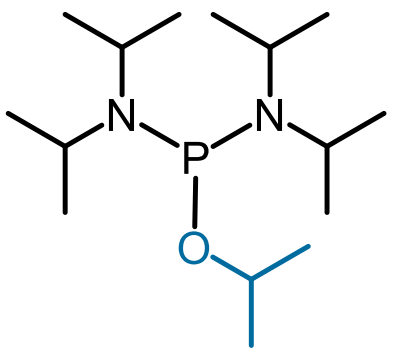

^{1}H NMR (500 MHz, C_6D_6, 298 K) δ 3.94 (m, 1H), 3.54 (m, 4H), 1.30–1.16 (m, 30H).

^{31}P NMR (203 MHz, C_6D_6) δ 113.3.

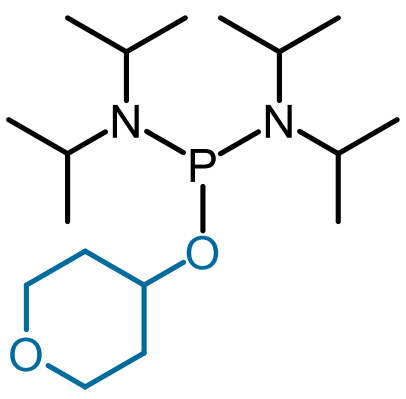

^{1}H NMR (500 MHz, C_6D_6, 298 K) δ ^{1}H NMR 4.01 (m, 2H), 3.92 (m, 1H), 3.61 (m, 4H), 3.51 (ddd, *J* = 11.3, 7.9, 3.3 Hz, 2H), 1.91 (m, 2H), 1.78 (m, 2H), 1.30 (d, *J* = 6.8 Hz, 24H).

^{31}P NMR (203 MHz, C_6D_6) δ 111.3.

^{1}H NMR (500 MHz, C_6D_6, 298 K) spectrum of **6a**

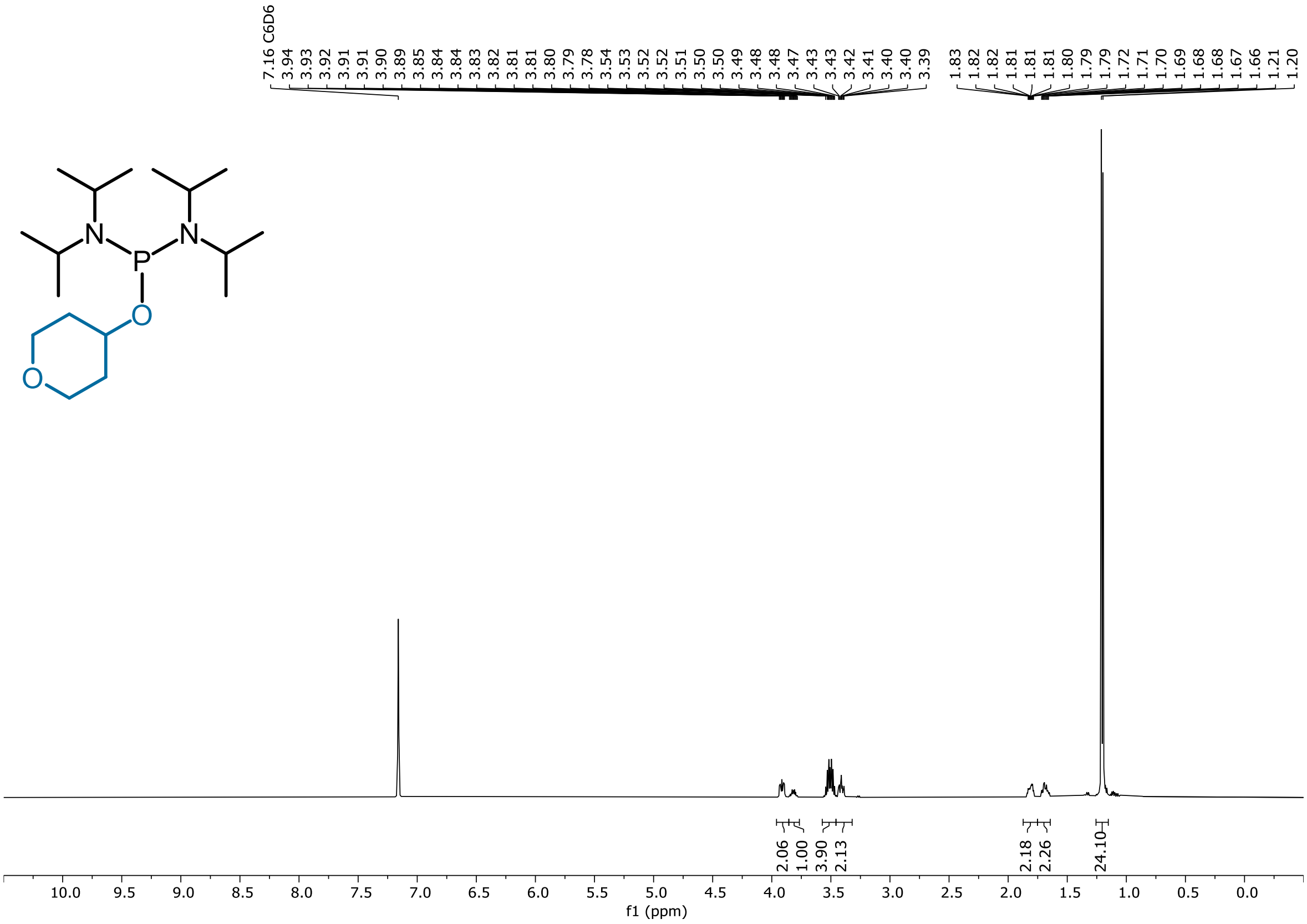

^{31}P NMR (203 MHz, C_6D_6, 298 K) spectrum of **6a**

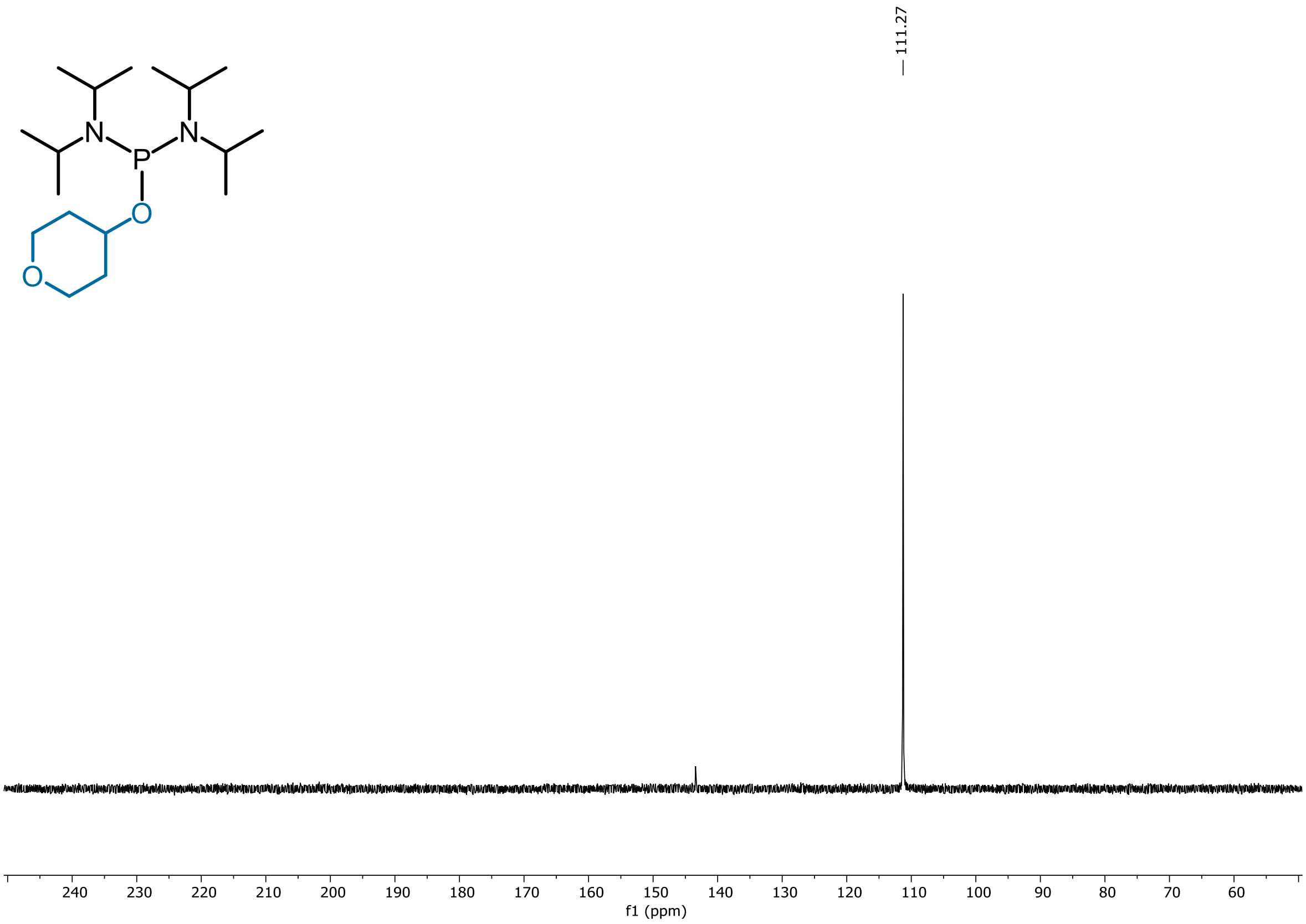

^{1}H NMR (500 MHz, C_6D_6, 298 K) spectrum of **6b**

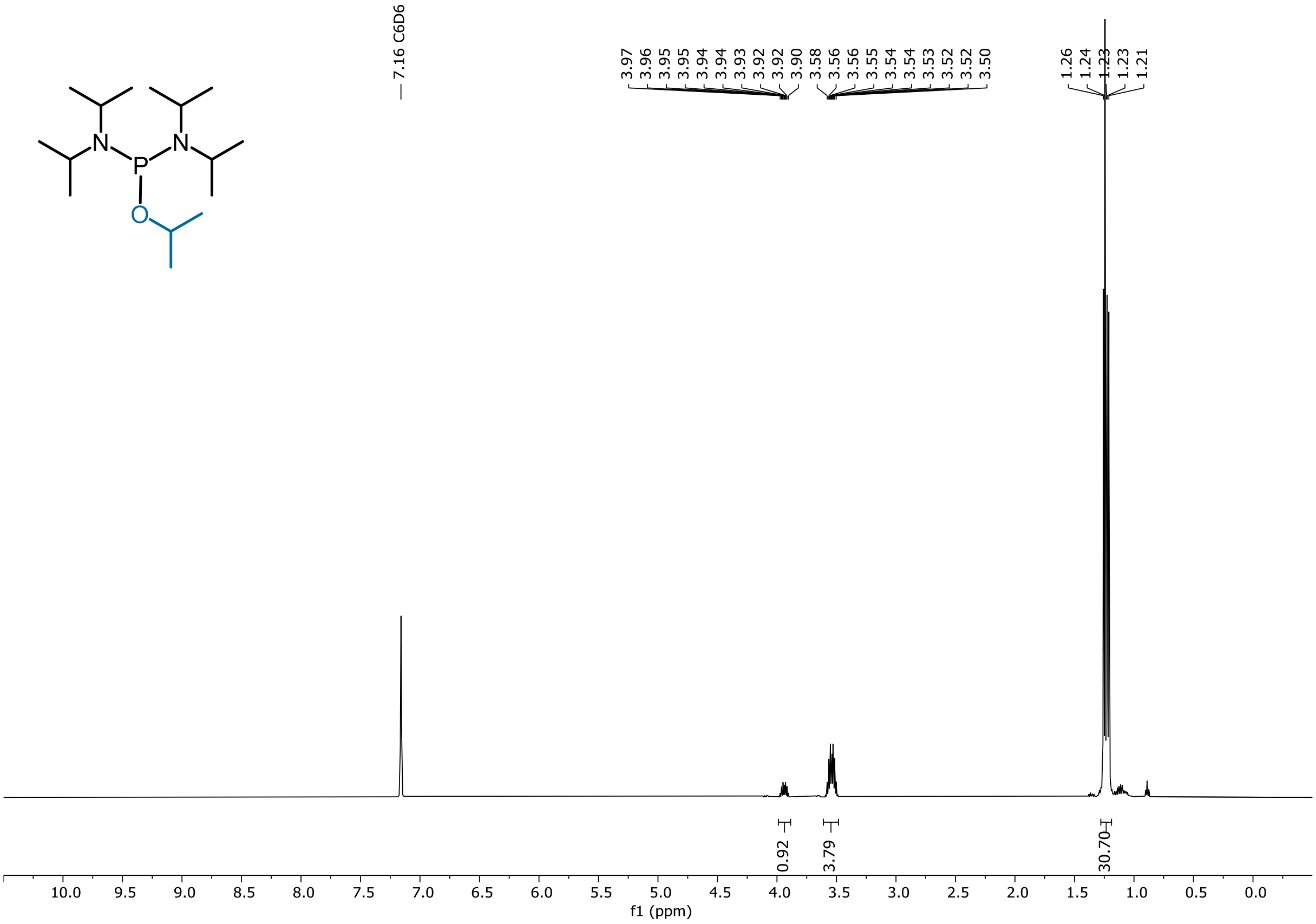

^{31}P NMR (203 MHz, C_6D_6, 298 K) spectrum of **6b**

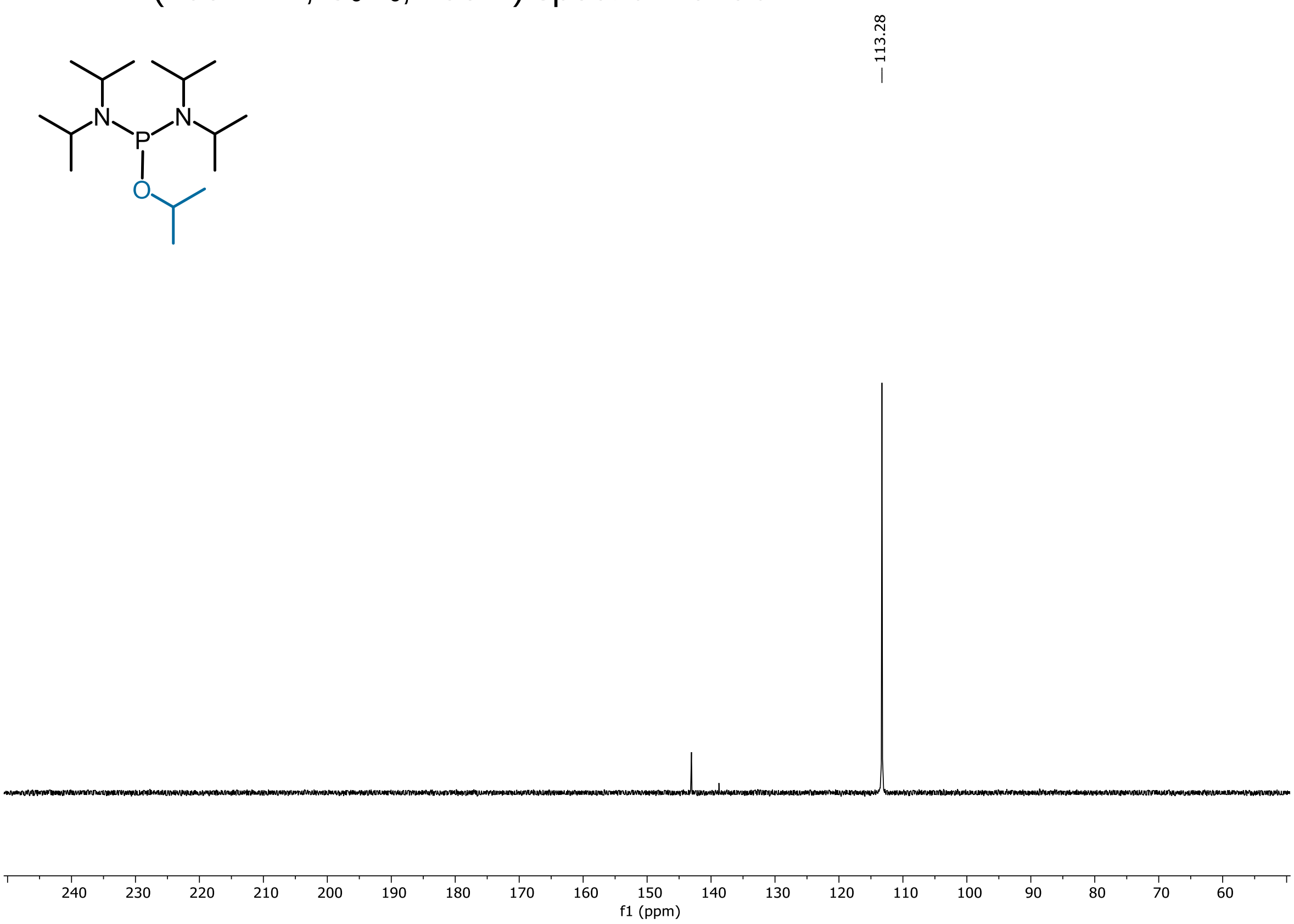

8.2 Reaction conditions for charge neutral phosphotriester backbone synthesis

15 mg of the polystyrene-supported SSQVYSPRPR oligonucleotide was loaded into an empty synthesis column (TWIST 40nm, 0.2um or 1um), flushed with argon, and washed with 1 mL of dry MeCN prior to synthesis. A 0.3 M solution of each phosphitylating reagent (**6a** and **6b**) in CH_2Cl_2 was prepared. 5-Benzylthio-1*H*-tetrazole (BTT, 0.3 M in MeCN) was used as the activator. 0.9 mL of the phosphitylating solution and 0.9 mL of the BTT solution were loaded into separate 2 mL syringes and pushed back and forth through the synthesis column for 10 minutes. Reactions were conducted at ambient temperature, with syringe push–pull mixing performed every 1–2 minutes to ensure complete consumption of the starting material. After phosphitylation, the column was washed with 1 mL MeCN, flushed with argon, and subjected to the coupling step using a premixed nucleoside solution (0.25 M in MeCN:CH_2Cl_2, 8:2 v/v). Following coupling, the resin was washed with 1 mL MeCN, flushed with argon, and treated with a 0.02 M iodine solution for oxidation of P(III) to P(V) at room temperature for 3 minutes. The resin was then thoroughly washed with MeCN and flushed with argon. Deprotection was carried out using ethylenediamine/toluene 1:1 (v/v) for 2 h at ambient temperature. The solution was concentrated under reduced pressure, and the resulting crude product was dissolved in 1 mL deionised water to prepare the stock solution.

8.3 UHPLC Analysis of crude phosphotriester-modified oligonucleotides (**7a-d**)

**Sequence**	**rt**	**Area (A)**	**Extinction coefficient (ε)**	**A/ε**	**Conversion**
SSQVYSPRPR	4.738	9047.847	110270	0.082051755	
SSQVYSPRPR	4.787	1172.697	110270	0.010634778	
SSQVYSPRPR	4.915	3277.246	110270	0.029720196	
T-SSQVYSPRPR	8.671	36374.59	118670	0.306518834	71%

Ion-pair reversed-phase UHPLC chromatogram of the crude reaction mixture (UV absorbance at 260 nm vs. time in minutes).

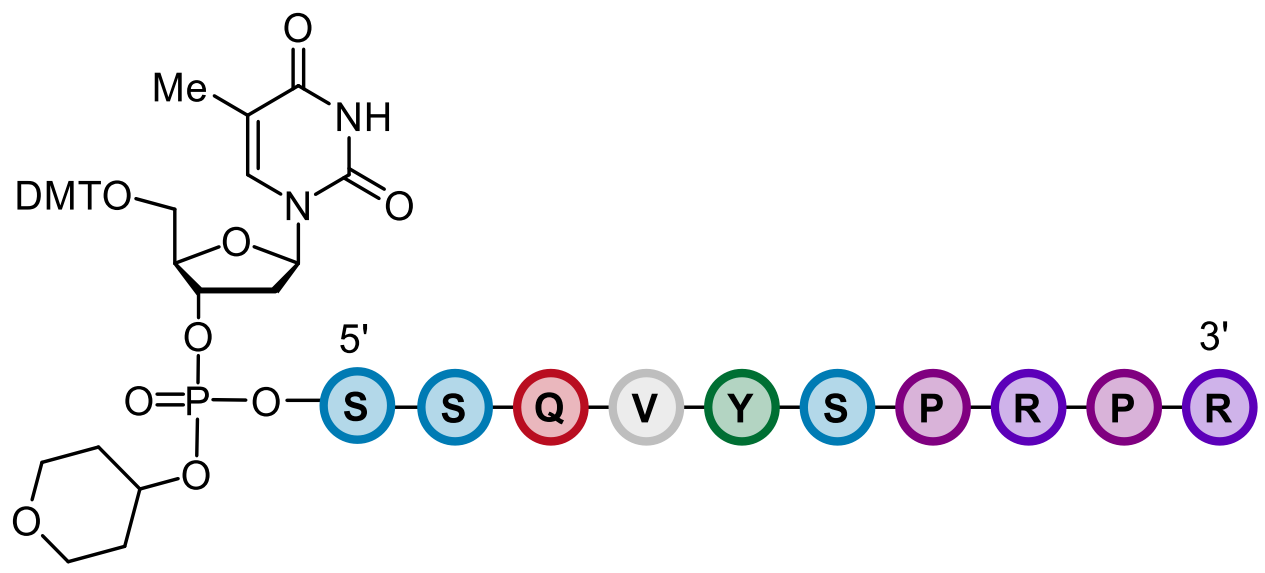

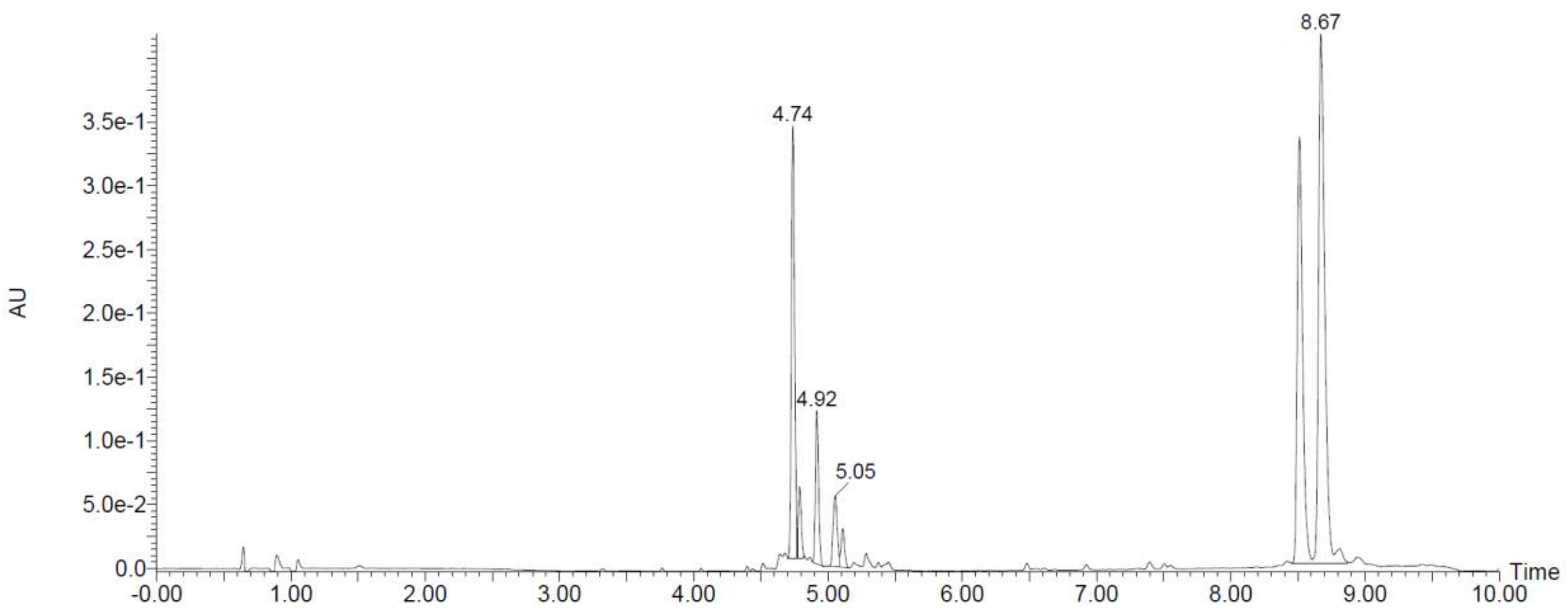

Mass spectrum (ES-) of **7a** (y-axis = relative intensity [%], x-axis = deconvolved mass [Da]); calculated mass: 3952.2, found: 3952.5.

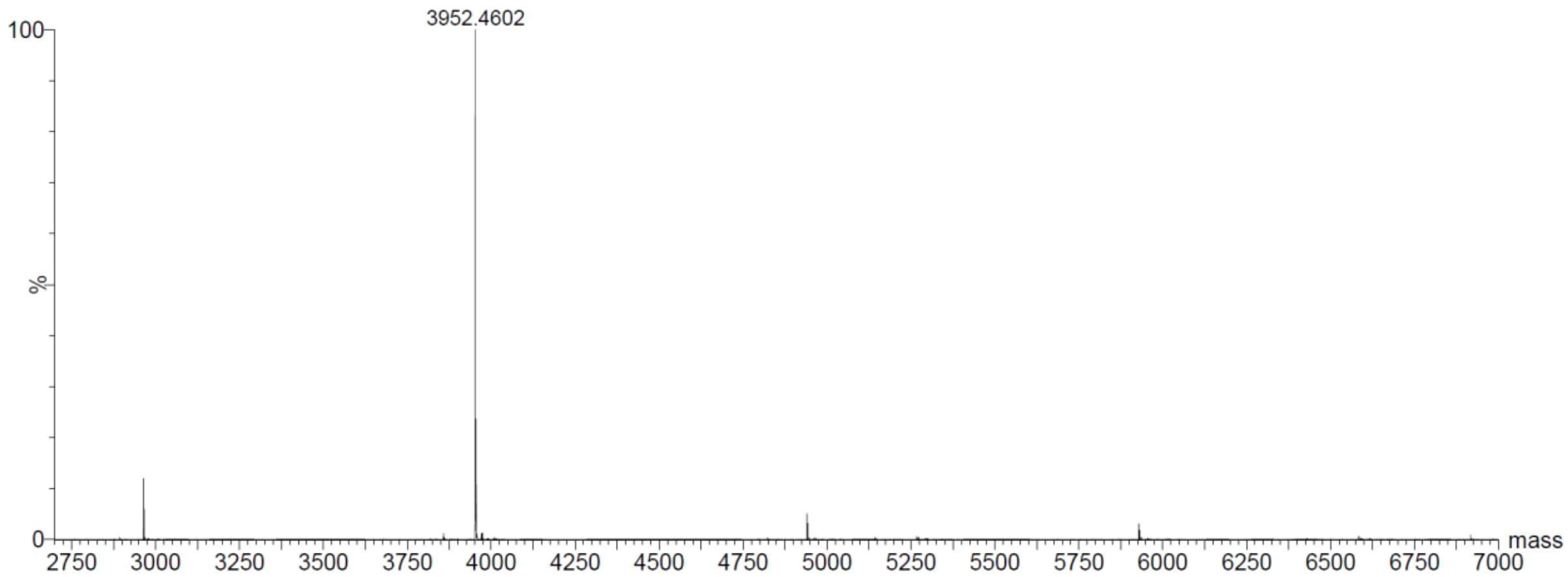

Sequence	rt	Area (A)	Extinction coefficient (ε)	A/ε	Conversion
SSQVYSPRPR	4.572	327.524	110270	0.0029702	
SSQVYSPRPR	4.745	381.053	110270	0.003455636	
T-SSQVYSPRPR	6.717	1747.01	110270	0.015843022	71%

Ion-pair reversed-phase UHPLC chromatogram of the crude reaction mixture (UV absorbance at 260 nm vs. time in minutes).

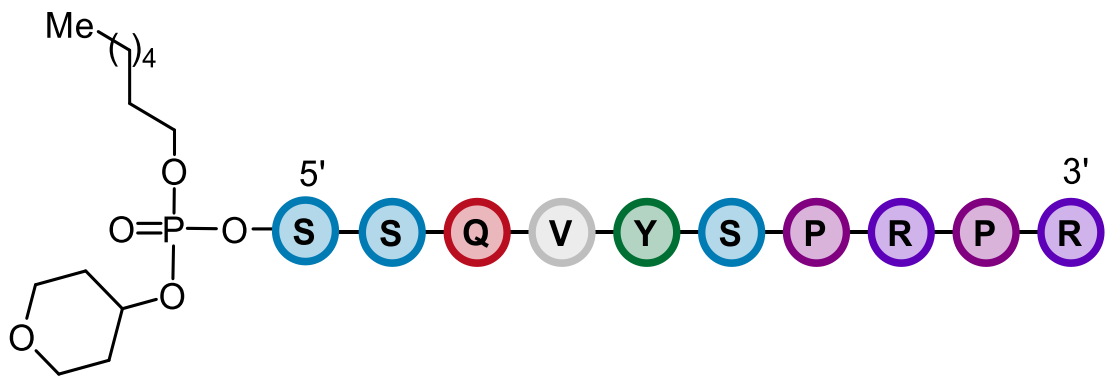

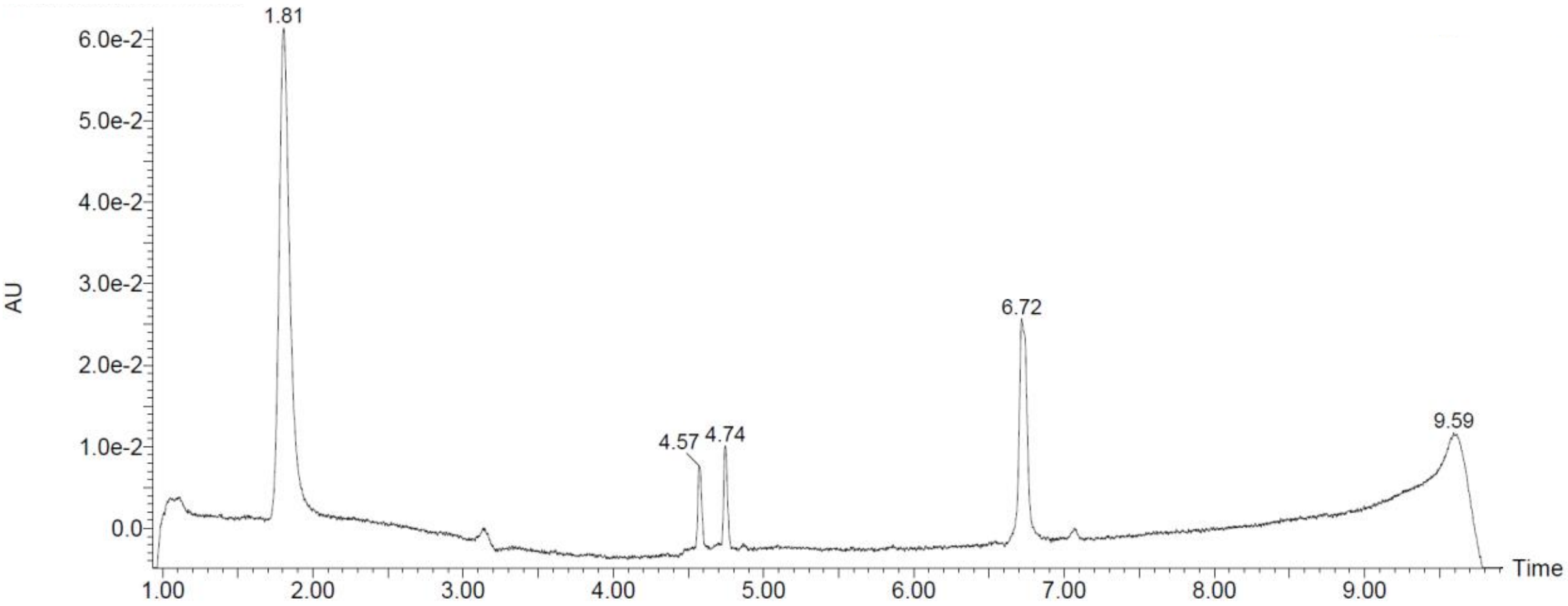

Mass spectrum (ES-) of **7b** (y-axis = relative intensity [%], x-axis = deconvolved mass [Da]); calculated mass: 3524.3, found: 3523.8.

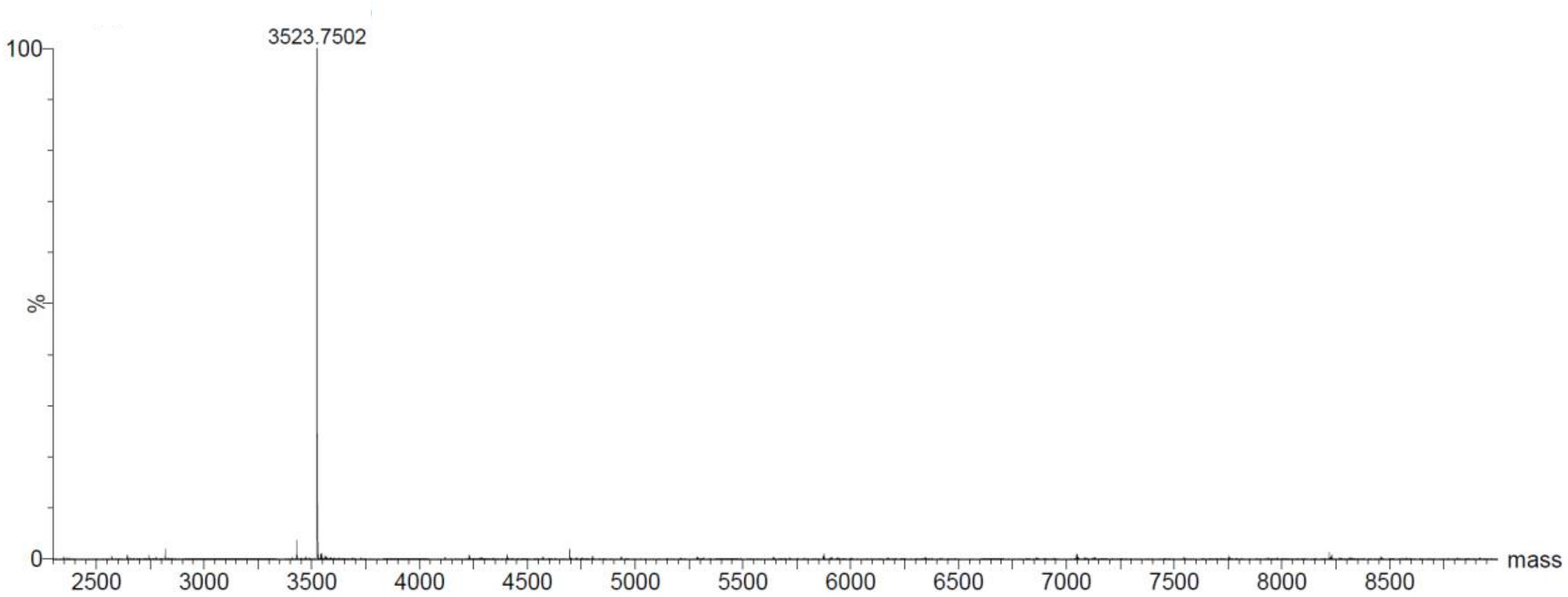

Sequence	rt	Area (A)	Extinction coefficient (ε)	A/ε	Conversion
SSQVYSPRPR	4.818	7426.528	110270	0.067348581	
SSQVYSPRPR	5.154	1525.579	110270	0.013834942	
T-SSQVYSPRPR	9.191	17689.074	118670	0.149061043	65%

Ion-pair reversed-phase UHPLC chromatogram of the crude reaction mixture (UV absorbance at 260 nm vs. time in minutes).

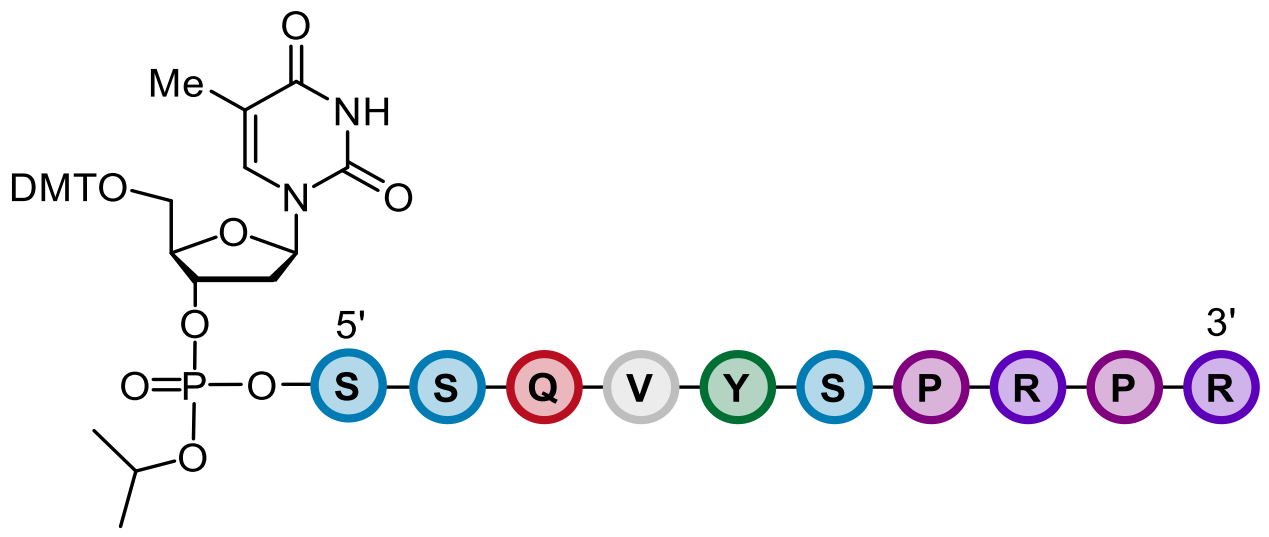

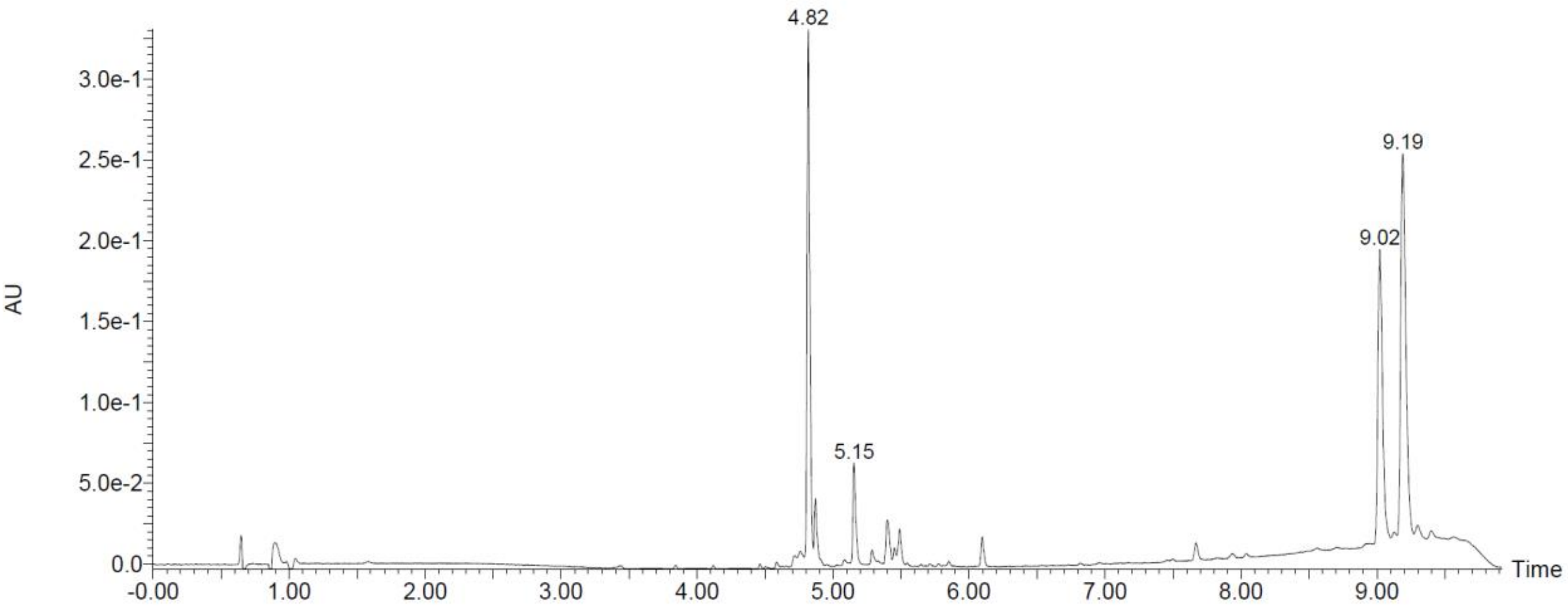

Mass spectrum (ES-) of **7c** (y-axis = relative intensity [%], x-axis = deconvolved mass [Da]); calculated mass: 3910.7, found: 3909.7.

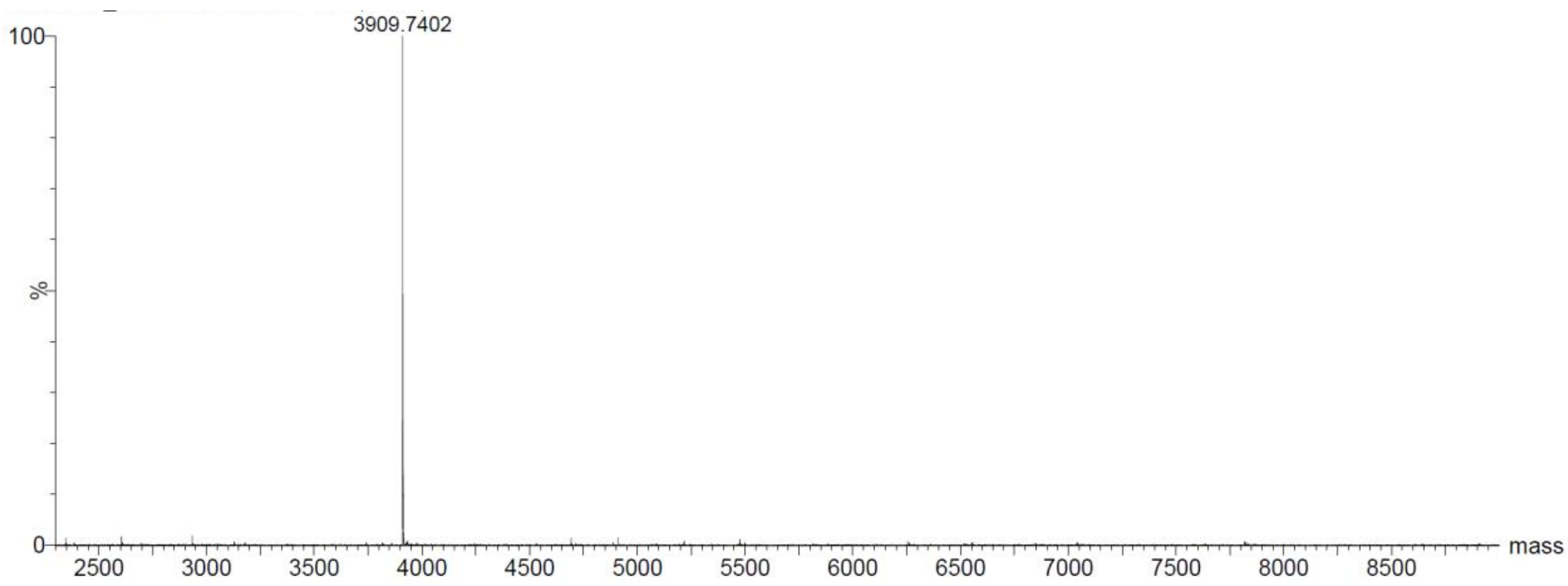

Sequence	rt	Area (A)	Extinction coefficient (ε)	A/ε	Conversion
SSQVYSPRPR	4.571	812.803	110270	0.007371026	
SSQVYSPRPR	4.88	222.264	110270	0.002015634	
**T-**SSQVYSPRPR	7.1	1763.376	110270	0.015991439	63%

Ion-pair reversed-phase UHPLC chromatogram of the crude reaction mixture (UV absorbance at 260 nm vs. time in minutes).

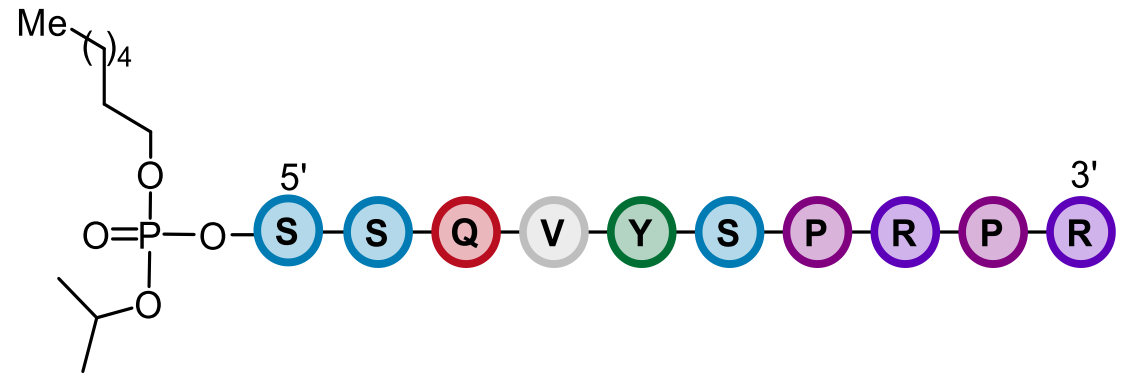

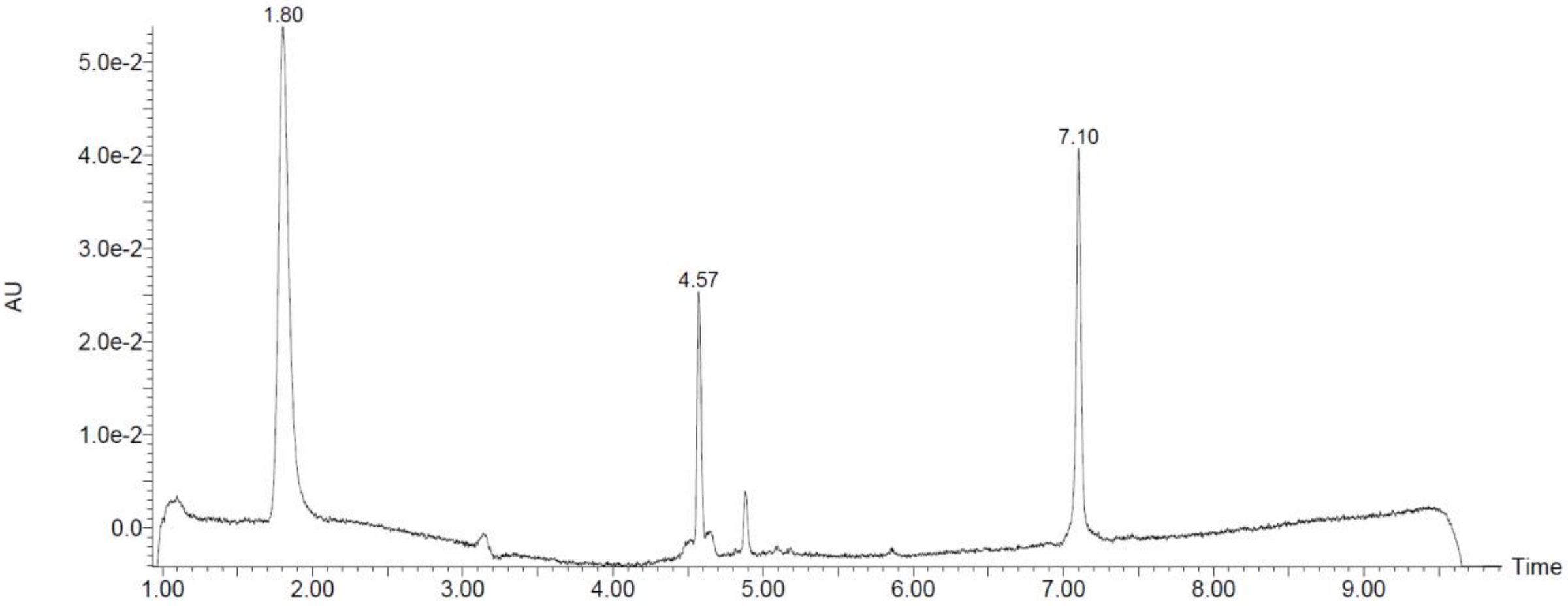

Mass spectrum of **7d** (ES-) (y-axis = relative intensity [%], x-axis = deconvolved mass [Da]); calculated mass: 3482.3 found: 3482.5.

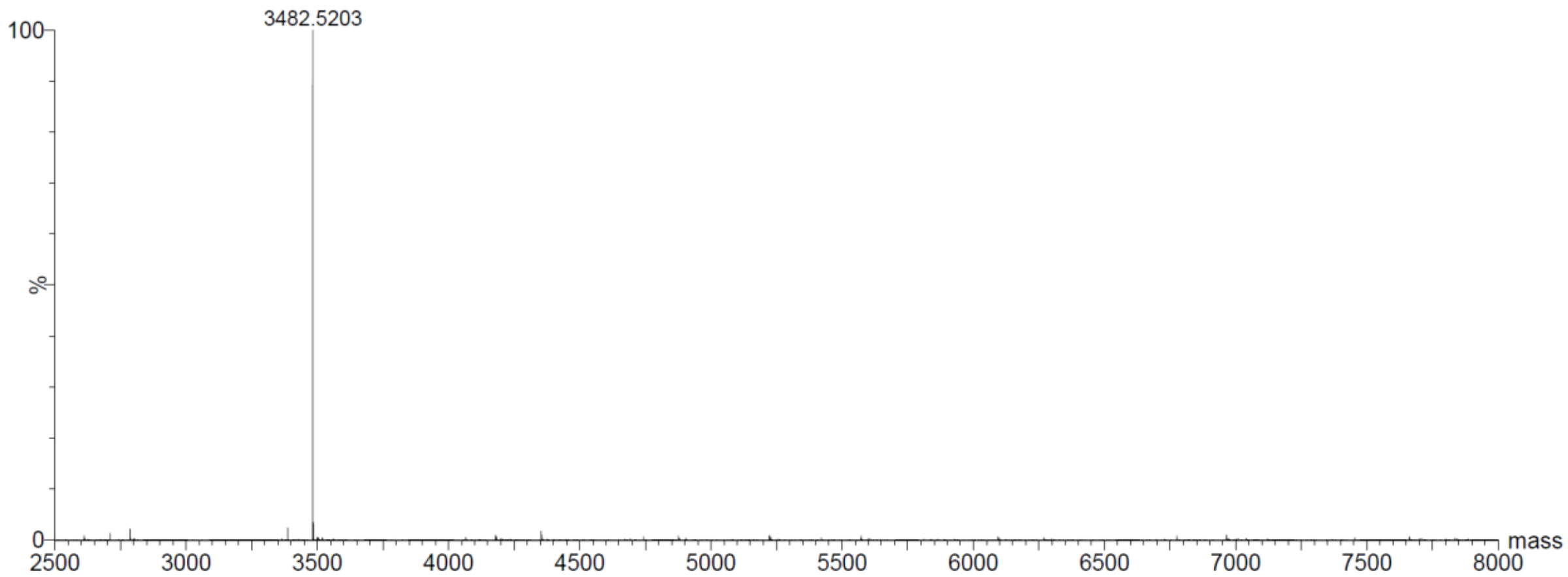

9. **5' Modification of the MALAT1 LNA/PS gapmer: installation of the azide group**

The modified 5'-OH oligonucleotide (MALAT1 LNA/PS Gapmer) was synthesized on an Applied Biosystems 394 automated DNA/RNA synthesizer using a general reaction protocol described in section 2.

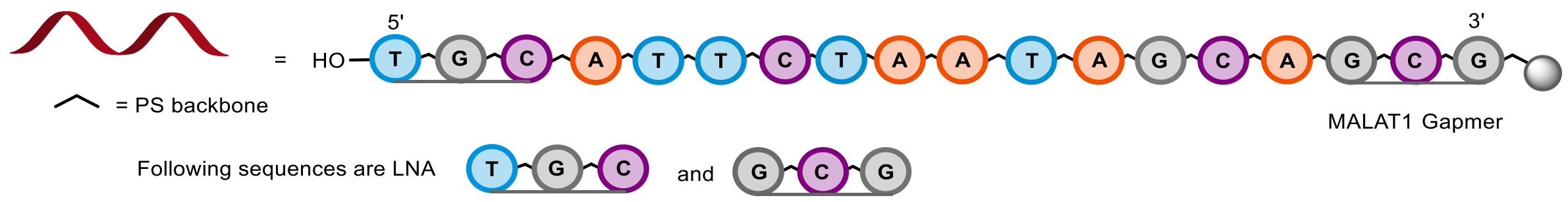

Ion-pair reversed-phase UHPLC chromatogram of the MALAT1 gapmer sequence (UV absorbance at 260 nm vs. time in minutes).

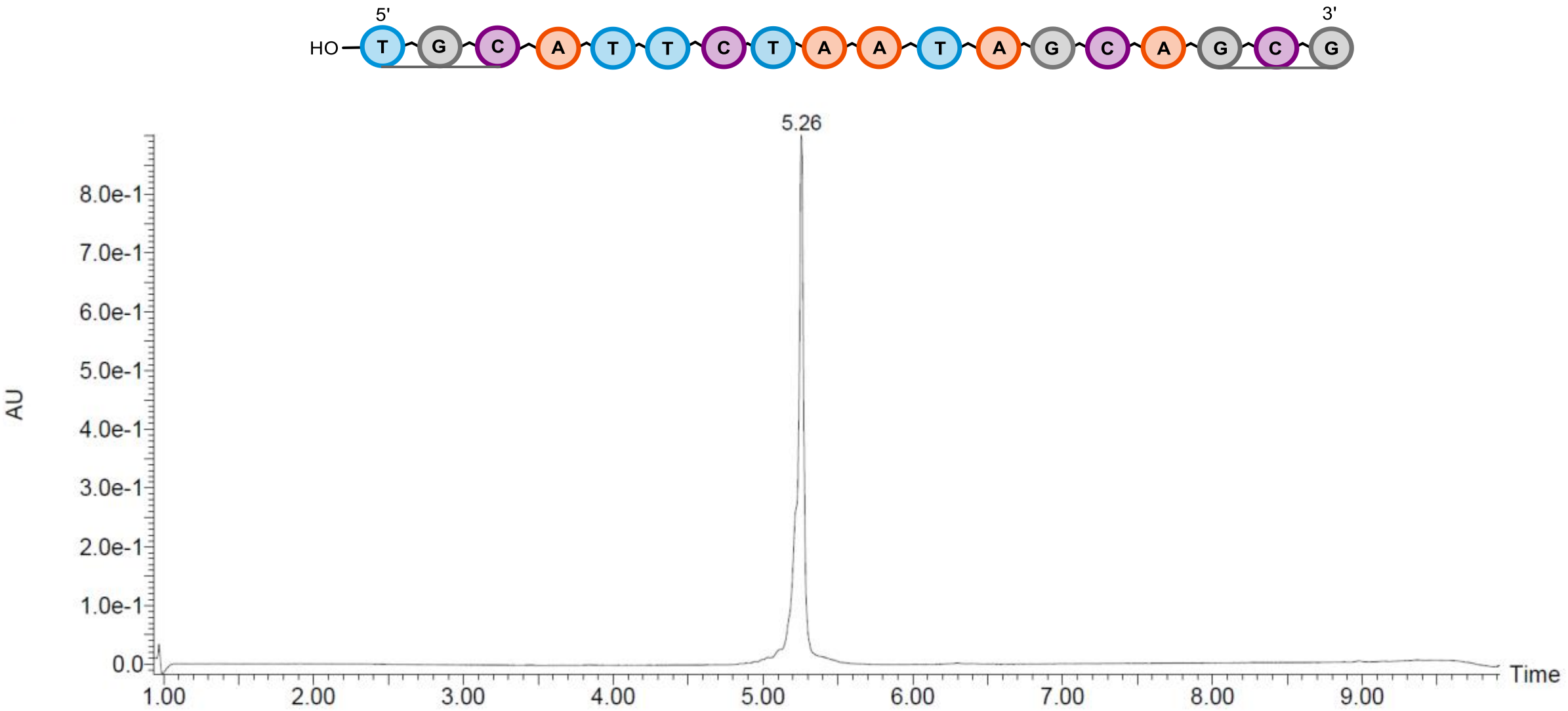

Mass spectrum (ES-) of the MALAT1 gapmer sequence (y-axis = relative intensity [%], x-axis = deconvolved mass [Da]); calculated mass: 5997.7, found: 5996.6.

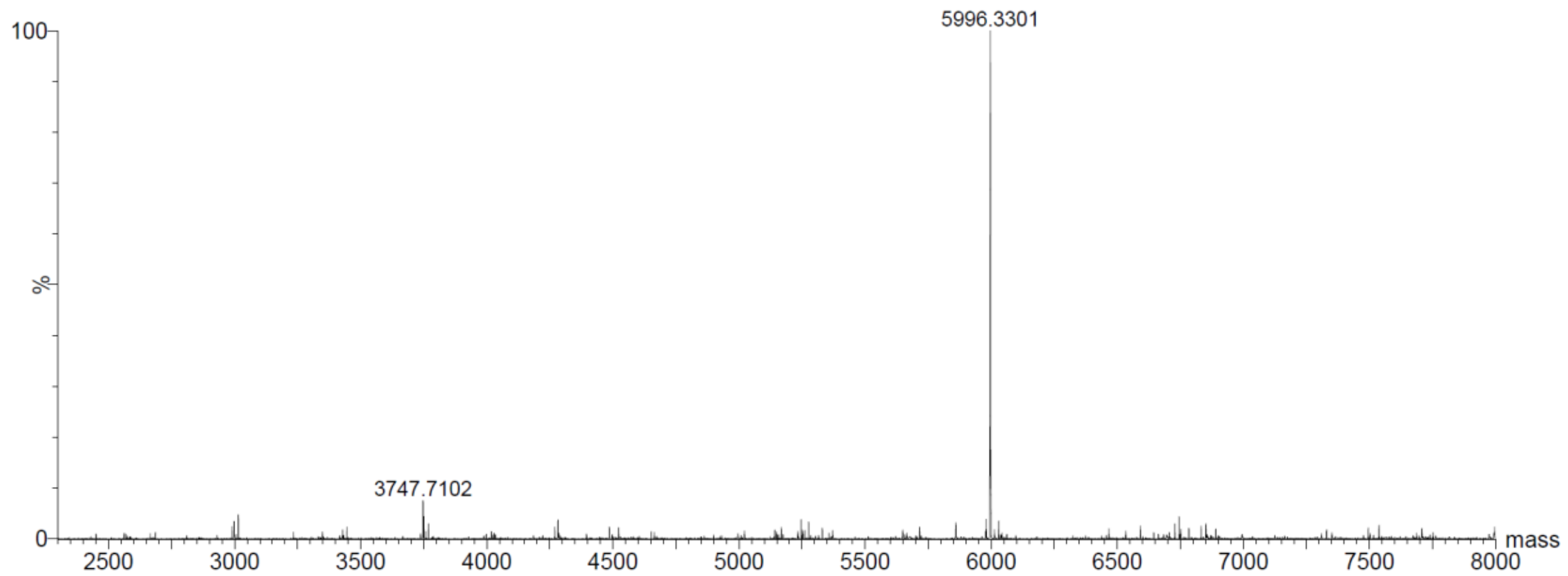

9.1 Attempted installation of the 5'-azide functionalities of a LNA sequences

The resin-bound sequences-MALAT1 Gapmer, was investigated for conversion of the 5'-OH group into an azide functionality following reported procedures[3]. 15 mg of the resin bounded sequence was loaded into an empty synthesis column (TWIST 40 nm, 0.2 µm, or 1 µm), flushed with argon, and washed with 1 mL of dry MeCN prior to the reaction. A 0.5 M solution of methyltriphenoxyphosphonium iodide in DMF was charged to the column and allowed to react for 1 h at ambient temperature, with syringe push–pull operations every 10 min to ensure efficient mixing. After 1 h, the column was thoroughly washed with DMF (2 × 2 mL) and MeCN (2 × 2 mL), flushed with argon, and dried. Subsequently, a 0.5 M solution of NaN_3 in DMF was introduced (2 mL) and the reaction was carried out for 5 h at 55 °C. Final deprotection was performed at 55 °C using 35% NH_3 solution (aq.).

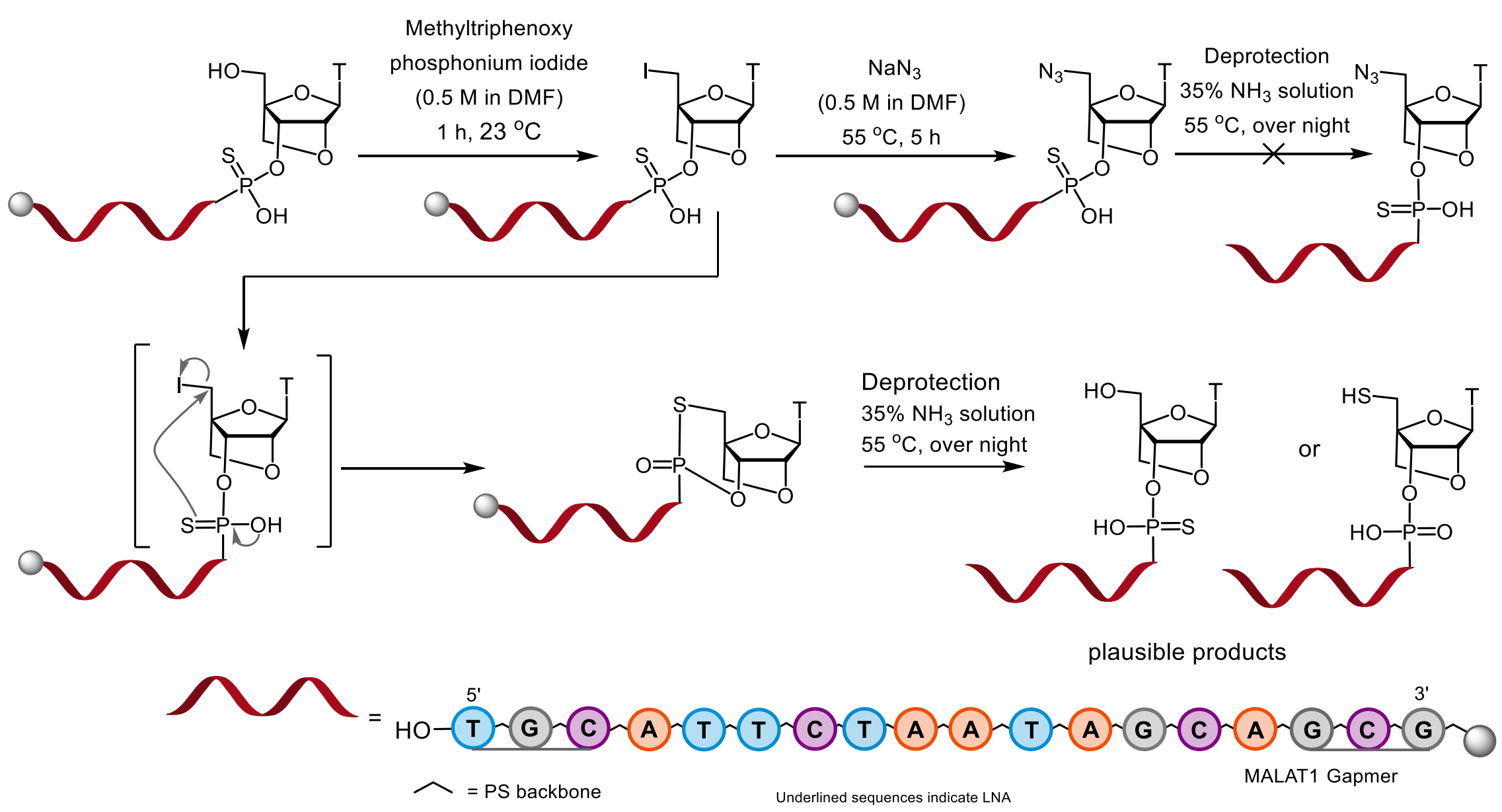

Ion-pair reversed-phase UHPLC chromatogram of the attempted reaction to install N_3 group at the 5' position of the MALAT1 gapmer sequence immobilised on polystyrene support (UV absorbance at 260 nm vs. time in minutes).

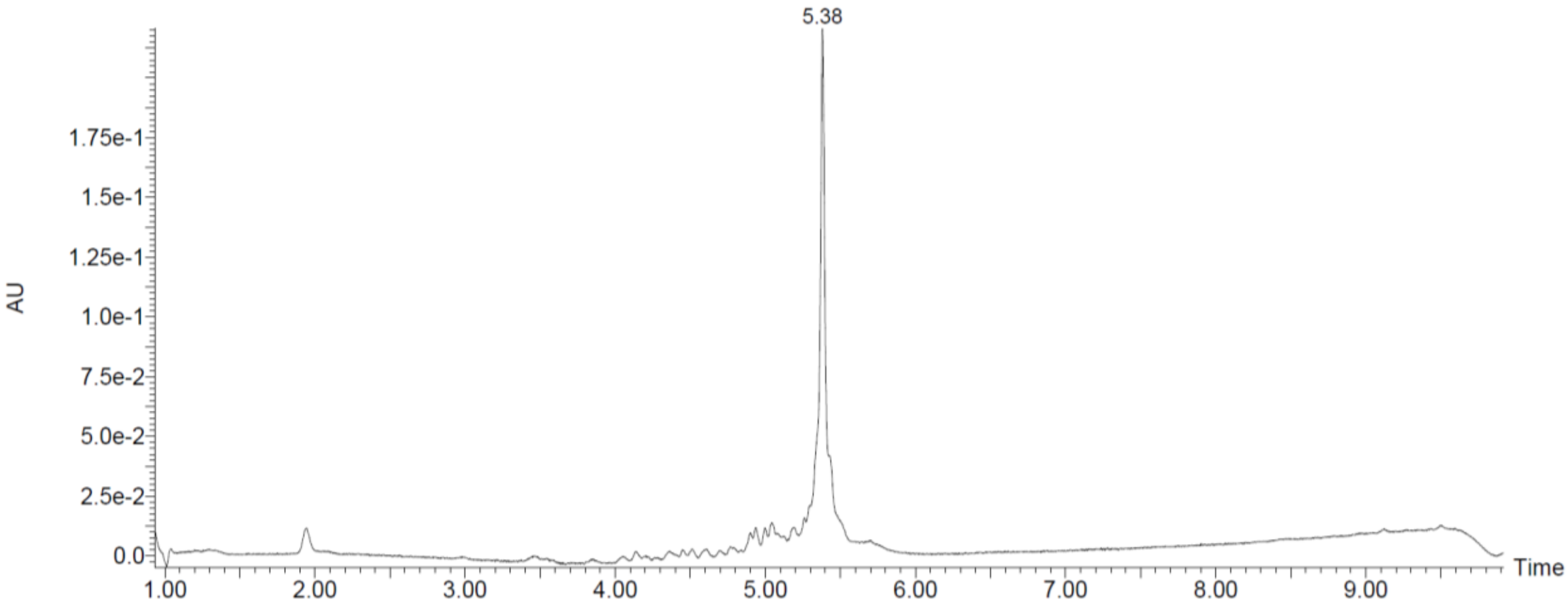

Mass spectrum (ES-) of the product (y-axis = relative intensity [%], x-axis = deconvolved mass [Da]).

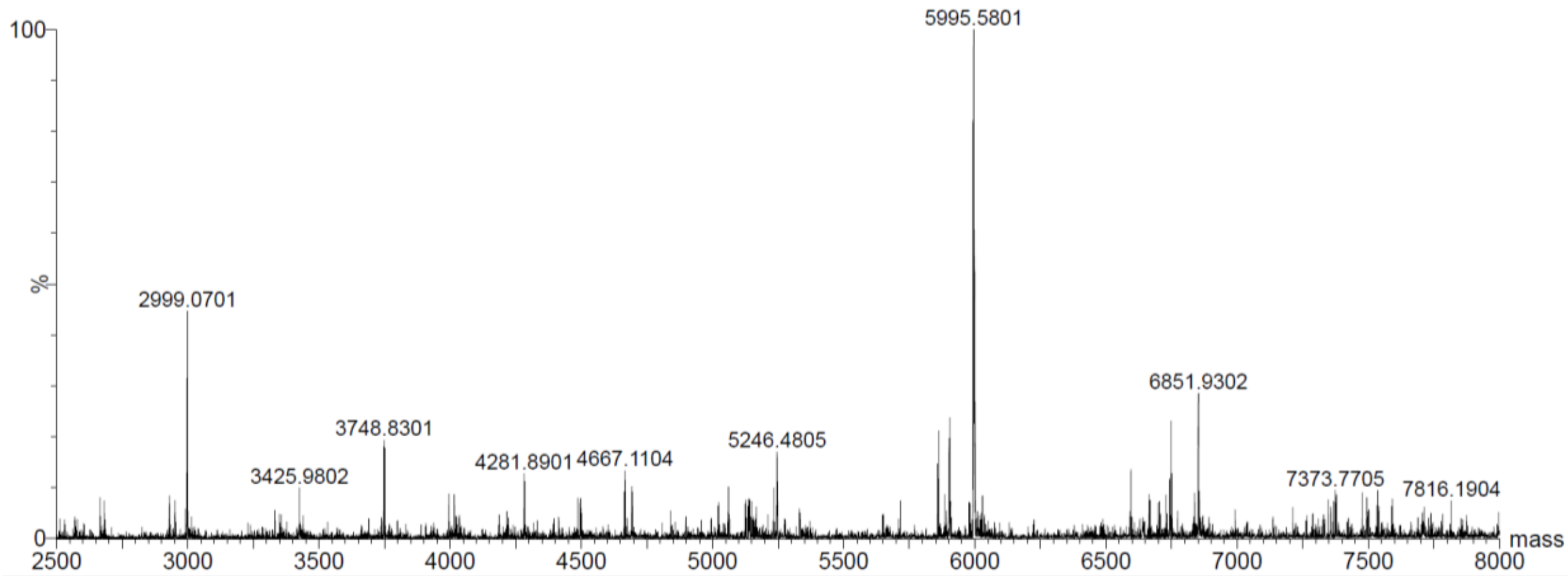

9.2 Synthesis and chromatogram of the azido-modified MALAT1 gapmer (**9**)

Synthesis of azido-modified oligonucleotide

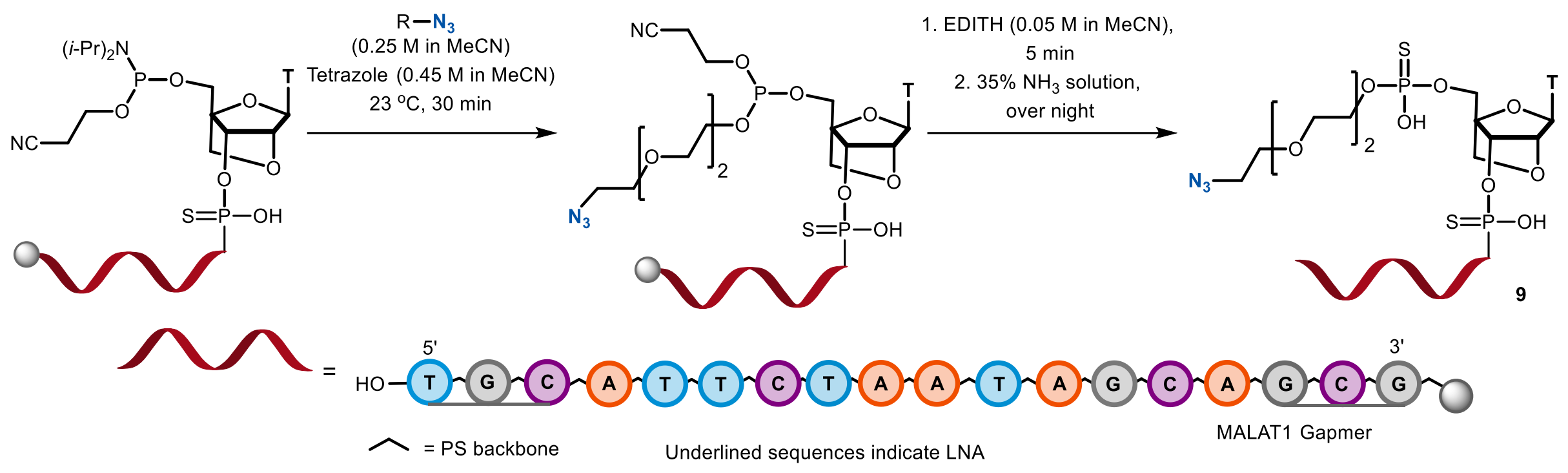

Ion-pair reversed-phase UHPLC chromatogram of the **R-**MALAT1 gapmer sequence **9** (UV absorbance at 260 nm vs. time in minutes). [R = Azido-PEG3-OH]

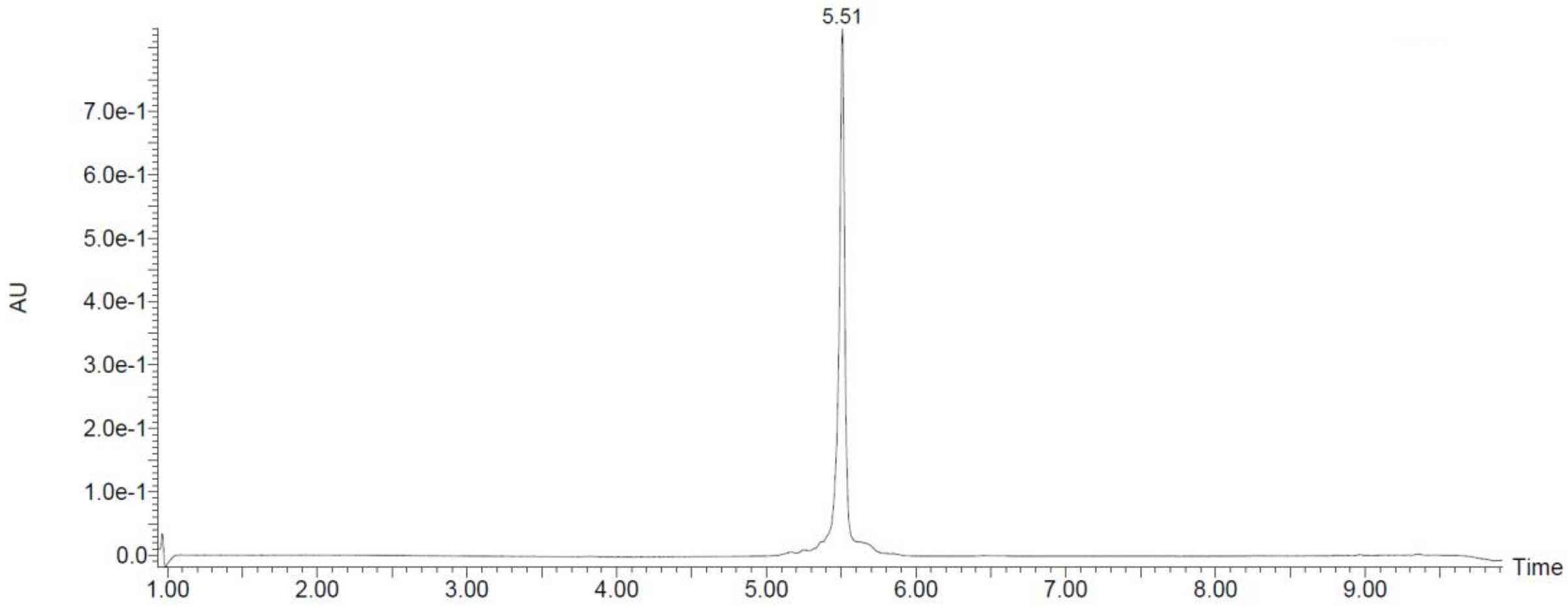

Mass spectrum (ES-) of **9** of the MALAT1 gapmer sequence (y-axis = relative intensity [%], x-axis = deconvolved mass [Da]); calculated mass: 6250.8, found: 6249.4.

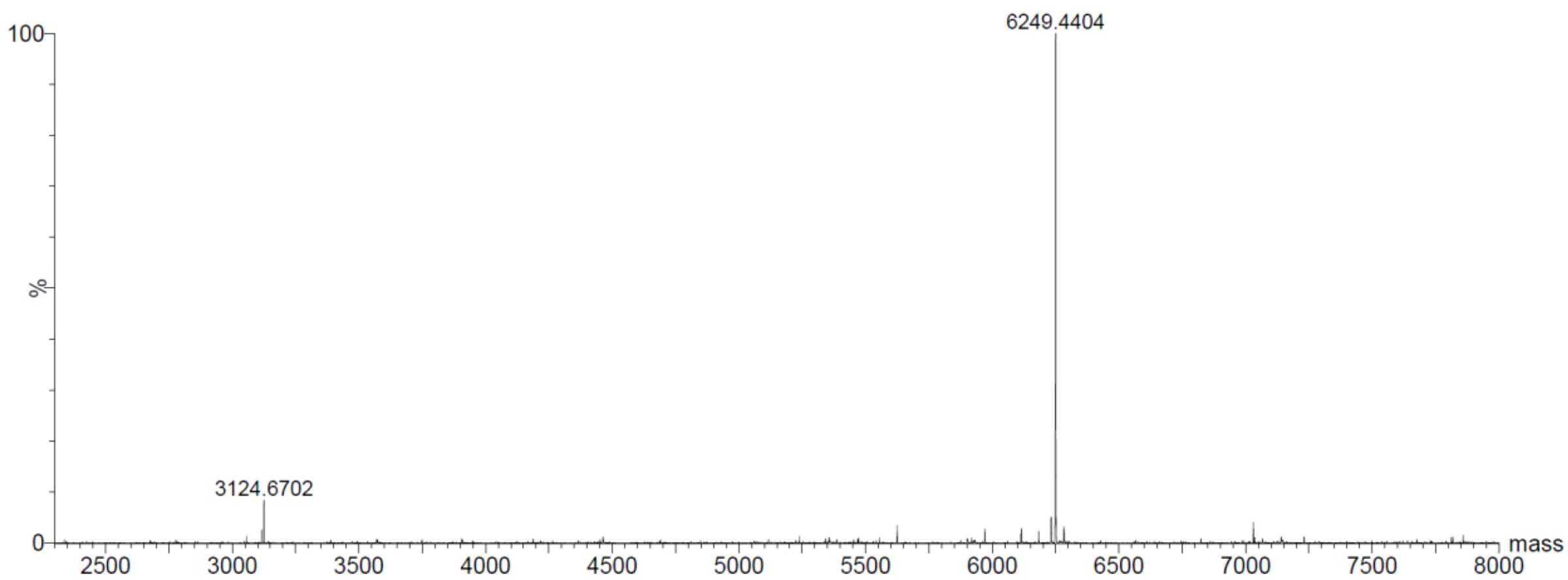

10. Statistics & reproducibility

Information on study design and sample sizes is provided in protocols listed in the Methods. No statistical method was used to predetermine sample size. No data were excluded from the analyses. The experiments were not randomised. The Investigators were not blinded to allocation during experiments and outcome assessment.

11. Cell culture

HeLa (ATCC #CCL-2) cells were a gift from Katherine A. Vallis (Oxford Institute for Radiation Oncology, Department of Oncology, University of Oxford) and were authenticated by ECACC. HeLa cells were cultured in DMEM, high glucose, GlutaMAX™ Supplement, pyruvate (#31966021, Thermo Fisher Scientific) medium containing glucose (4.5 g/L), GlutaMAX™ Supplement (862 mg/L), sodium pyruvate (110 mg/L), sodium bicarbonate (3.7 g/L), and phenol red (15 mg/L), supplemented with 10% (v/v) fetal bovine serum (#A5209402, Thermo Fisher Scientific) at 37 °C and 5% CO_2. For microscopy experiments, the medium was exchanged with FluoroBrite™ DMEM (#A1896701, Thermo Fisher Scientific) containing glucose (4.5 g/L) and sodium bicarbonate (3.7 g/L), supplemented with 10% (v/v) fetal bovine serum (#A5209402, Thermo Fisher Scientific), sodium pyruvate (#11360070, Thermo Fisher Scientific, 1X, 110 mg/L), and GlutaMAX™ Supplement (#35050061, Thermo Fisher Scientific, 1X, 434 mg/L). In regular subcultures, TrypLE™ Express Enzyme (1X), no phenol red (#12604013, Thermo Fisher Scientific) was used for cell dissociation. Regular mycoplasma testing was performed using a MycoAlert® PLUS Mycoplasma Detection Kit (#LT07-701, Lonza).

12. Microscopy

To assess the applicability of the 5'-PEG$_3$-N$_3$ handle for oligonucleotide detection in HeLa cells, oligonucleotide treatment, fixation, SPAAC-based staining, and confocal imaging were performed.

6 h prior to the treatment, 1 × 10^4 HeLa cells per well were seeded in a poly-D-lysine (#A3890401, Thermo Fisher Scientific) treated Coverslips for Chambers, removable (#10811, Ibidi) with attached 8 Well Chamber (#80841, Ibidi) . To prepare the mixtures for gymnotic delivery, each oligonucleotide (250 µM, 24 µL) was added to 276 µL of FluoroBrite™ DMEM (#A1896701, Thermo Fisher Scientific) containing glucose (4.5 g/L) and sodium bicarbonate (3.7 g/L), supplemented with 10% (v/v) fetal bovine serum

(#A5209402, Thermo Fisher Scientific), sodium pyruvate (#11360070, Thermo Fisher Scientific, 1X, 110 mg/L), and GlutaMAX™ Supplement (#35050061, Thermo Fisher Scientific, 1X, 434 mg/L). Subsequently, the cell culture medium was exchanged with the prepared gymnosis solutions in medium (300 µL/well), and the cells were incubated for 72 h.

Next, cells were washed 3 times with 300 µL of HBSS, calcium, magnesium, no phenol red (#14025092, Thermo Fisher Scientific), fixed by incubating with 300 µL of 4% (w/v) formaldehyde (#10630813, Fisher Scientific) and 1% (v/v) glutaraldehyde (#A17876.AP, Thermo Fisher Scientific) solution in PBS (#10010023, Thermo Fisher Scientific) for 1 h at room temperature, washed 3 times with PBS (300 µL), permeabilised by incubating with 300 µL of 0.1% (v/v) Triton X-100 (#T8787-50ML, Sigma-Aldrich) solution in PBS for 5 min at room temperature, washed 3 times with PBS (300 µL), incubated with 300 µL of ethanolamine (#398136-25ML, Sigma-Aldrich, 100 mM) solution in PBS for 1 h at room temperature, washed 3 times with PBS (300 µL), incubated with 3% (w/v) bovine serum albumin (#A3156-5G, Sigma-Aldrich) in PBS for 1 h at room temperature, and washed 3 times with 300 µL of 0.1% (v/v) TWEEN® 20 (#P1379-100ML, Sigma-Aldrich) solution in PBS. Next, the cells were incubated with 300 µL of CellTracker™ Deep Red (#C34565, Thermo Fisher Scientific, 250 nM) solution in PBS for 30 min at room temperature. Afterwards, the cells were washed 3 times with 300 µL of 0.1% (v/v) TWEEN® 20 solution in PBS, incubated with 300 µL of iodoacetamide (#F001085-10G, Fluorochem, 100 mM) solution in PBS for 1 h at room temperature, washed 3 times with 300 µL of 0.1% (v/v) TWEEN® 20 solution in PBS, incubated with 300 µL of DBCO-AF488 (#CLK-1278-1, Jena Bioscience, 15 µM) in 0.1% (v/v) TWEEN® 20, 0.1% (w/v) bovine serum albumin solution in PBS for 1 h at room temperature, washed 3 times with 300 µL of 0.1% (v/v) TWEEN® 20 solution in PBS, incubated with 300 µL of DAPI (#D8417-5MG, Sigma-Aldrich, 1 µM) solution in PBS for 5 min at room temperature, and washed 1 time with 300 µL of PBS. Afterwards, the coverslip was mounted on imaging slide (#80841, Ibidi) with ProLong™ Glass Antifade Mountant (#P36982, Thermo Fisher Scientific) and left for 48 h at room temperature to cure.

The imaging of the stained cells was performed collecting 5 µm *z*-stacks of 69.33 µm x 69.33 µm tiles with 100 nm spacing. Spinning disk confocal microscopy was carried out on an Olympus IXplore SpinSR Super Resolution Microscope System consisting of the IX-83 inverted microscope frame equipped with a Yokogawa CSU-W1 SoRa confocal scanner unit, Teledyne Photometrics Prime BSI (used in the experiment) and Prime 95B sCMOS

cameras, SD-MGCA motorised magnification changer with 1x and 3.2x magnification (used in the experiment), SoRa (used in the experiment) and 50 µm pinhole disks, IX3 DIC optics, IX3-ZDC2 TruFocus Z-Drift compensation module, Coherent OBIS LX 405 nm (used for DAPI), 488 nm (used for AF488), 561 nm, 640 nm (used for CellTracker™ Deep Red) lasers, B447/60 (used for DAPI), B525/50 (used for AF488), B617/73, and B685/40 (used for CellTracker™ Deep Red) bandpass filters, and Olympus UPLAPO60XOHR 60x/1.50 (used in the experiment) oil immersion objective. In all experiments, the disk speed was set to 4000 rpm. Fixed cell imaging was carried out at room temperature and atmospheric CO_2 level. For instrument control and data acquisition, cellSens Dimension 3.2 software (Olympus) was used.

Microscopy images were processed (cropping, adjusting levels, applying LUTs, adding scale bars, exporting) and analysed (profiles) using Fiji 2.16.0 (National Institutes of Health, USA). Whole cells and nuclei were segmented based on intensities in CellTracker™ Deep Red and DAPI channels.

13. Supplementary scripts

The following software/package versions were used for data analysis: R v4.3.1, ggplot2 v4.0.0, dplyr v1.1.4, readr v2.1.5, Python v3.12.11.

ABI 394 cycle parser

```
#!/usr/bin/env python3
"""
OligoNET cycle decode/encode

# Decode ABIF → CSV
python cycle.py decode "<input>" "<output.csv>"

# Encode CSV → ABIF
python cycle.py encode "<input.csv>" "<output>"
"""

from __future__ import annotations
import struct, csv
from dataclasses import dataclass
from pathlib import Path
from typing import Dict, List, Tuple, Iterable, Optional

# mappings
NUM_TO_NAME: Dict[int, str] = {
    # CONTROL
    103: "Wait",
    104: "Interrupt",
    105: "Start Detrityl",
    106: "Begin",
    107: "End",
    108: "Block to Col",
    109: "Waste - Port",
    110: "Waste - Bottle",
    112: "Trityl Advance",
    113: "End Advance",
    120: "Advance FC",
    121: "Relay 1 On",
```

```
122: "Relay 1 Off",
123: "Relay 1 Pulse",
124: "Relay 2 On",
125: "Relay 2 Off",
126: "Relay 2 Pulse",
127: "Relay 3 On",
128: "Relay 3 Off",
129: "Relay 3 Pulse",
130: "Relay 4 On",
131: "Relay 4 Off",
132: "Relay 4 Pulse",
133: "Relay 5 On",
134: "Relay 5 Off",
135: "Monitor Trityls",
136: "Monitor noise",
137: "Stop monitor",
167: "If Monitoring",
168: "If not Monitoring",
169: "End Monitoring",

# BOTTLE CONTROL
150: "Bottle01 On",
151: "Bottle01 Off",
152: "Bottle02 On",
153: "Bottle02 Off",
154: "Bottle03 On",
155: "Bottle03 Off",
156: "Bottle04 On",
157: "Bottle04 Off",
158: "Bottle05 On",
159: "Bottle05 Off",
160: "Bottle06 On",
161: "Bottle06 Off",
162: "Bottle07 On",
163: "Bottle07 Off",
164: "Bottle08 On",
165: "Bottle08 Off",

# PRIME DELIVERY LINES
50: "A to Waste",
51: "G to Waste",
52: "C to Waste",
53: "T to Waste",
54: "5 to Waste",
55: "6 to Waste",
56: "7 to Waste",
57: "8 to Waste",
58: "Tet to Waste",
59: "10 to Waste",
60: "11 to Waste",
61: "12 to Waste",
62: "14 to Waste",
63: "15 to Waste",
64: "18 to Waste",
65: "19 to Waste",
101: "Phos Prep",
102: "Cap Prep",
115: "Prep 10",
116: "Prep 14",
117: "Prep 15",
118: "Prep 18",
119: "Prep 18 + 19",

# ARGON FLUSHES
1: "Block flush",
2: "Reverse flush",
3: "Trityl Flush",
4: "Flush to Waste",
5: "Flush to Collect",
6: "Flush to A",
7: "Flush to G",
8: "Flush to C",
9: "Flush to T",
10: "Flush to 5",
```

```
    11: "Flush to 6",
    12: "Flush to 7",
    13: "Flush to 8",
    14: "Flush to Bases",
    15: "Flush to Tet",
    16: "Flush to 10",
    17: "Flush to 11+12",
    18: "Flush to 14+15",
    19: "Flush to 18",
    20: "Flush to 11",
    21: "Flush to 12",
    22: "Flush to 14",
    23: "Flush to 15",
    24: "Flush to 19",

    # REAGENTS TO RESERVOIRS
    70: "18 to A",
    71: "18 to G",
    72: "18 to C",
    73: "18 to T",
    74: "18 to 5",
    75: "18 to 6",
    76: "18 to 7",
    77: "18 to 8",
    78: "18 to Tet",
    79: "18 to 10",
    80: "18 to 11",
    81: "18 to 12",
    82: "18 to 11+12",
    83: "18 to 14",
    84: "18 to 15",
    85: "18 to 14+15",
    86: "18 to Bases",
    90: "15 to A",
    91: "15 to G",
    92: "15 to C",
    93: "15 to T",
    94: "15 to bases",

    # REAGENTS TO COLUMN
    25: "A to Column",
    26: "G to Column",
    27: "C to Column",
    28: "T to Column",
    29: "5 to Column",
    30: "6 to Column",
    31: "7 to Column",
    32: "8 to Column",
    33: "B+Tet to Column",
    34: "Tet to Column",
    35: "5+Tet to Column",
    36: "10 to Collect",
    37: "11 to Column",
    38: "12 to Column",
    39: "Cap to Column",
    40: "14 to Column",
    41: "15 to Column",
    42: "18 to Column",
    43: "Push to Column",
    44: "19 to Column",

    # VENTING
    100: "10 Vent",
    111: "Block Vent",
    114: "Vent Bases",

    # COLUMN CONTROL
    140: "Column 1 On",
    141: "Column 1 Off",
    142: "Column 2 On",
    143: "Column 2 Off",
    144: "Column 3 On",
    145: "Column 3 Off",
    146: "Column 4 On",
```

```
    147: "Column 4 Off",

    # AUTODILUTE
    170: "Dilute 'A'",
    171: "Dilute 'G'",
    172: "Dilute 'C'",
    173: "Dilute 'T'",
    174: "Dilute '5'",
    175: "Dilute '6'",
    176: "Dilute '7'",
    177: "Dilute '8'",
}

ACTIVE_BIT_LABELS: List[Tuple[int, str]] = [
    (1, "A"),
    (2, "G"),
    (4, "C"),
    (8, "T"),
    (16, "5"),
    (32, "6"),
    (64, "7"),
    (128, "8"),
]

SAFE_TO_TEXT: Dict[int, str] = {0: "No", 1: "Yes"}
TEXT_TO_SAFE: Dict[str, int] = {"no": 0, "yes": 1, "0": 0, "1": 1}

# ABIF structs

DIR_STRUCT = ">4sIHHIIII"  # name(4), number, etype, esize, numelem, datasize, dataoffset, handle
DIR_SIZE = struct.calcsize(DIR_STRUCT)

@dataclass
class AbifDir:
    tag: str
    number: int
    etype: int
    elem_size: int
    num_elem: int
    data_size: int
    data_offset: int
    handle: int

    @classmethod
    def from_bytes(cls, b: bytes) -> "AbifDir":
        name, num, et, es, ne, ds, do, dh = struct.unpack(DIR_STRUCT, b)
        return cls(name.decode('ascii', 'replace'), num, et, es, ne, ds, do, dh)

    def to_bytes(self) -> bytes:
        return struct.pack(DIR_STRUCT,
                           self.tag.encode('ascii'), self.number, self.etype,
                           self.elem_size, self.num_elem, self.data_size,
                           self.data_offset, self.handle)

@dataclass
class AbifFile:
    version: int
    root_dir: AbifDir
    dirs: List[AbifDir]
    blob: bytearray

    @classmethod
    def load(cls, path: Path) -> "AbifFile":
        data = bytearray(path.read_bytes())
        if data[:4] != b"ABIF":
            raise ValueError("Not an ABIF file")
        version = struct.unpack(">H", data[4:6])[0]
        root = AbifDir.from_bytes(data[6:6+DIR_SIZE])
        count = root.data_size // root.elem_size
        dirs = []
        for i in range(count):
            off = root.data_offset + i*root.elem_size
            entry = AbifDir.from_bytes(data[off:off+DIR_SIZE])
            dirs.append(entry)
```

```
        return cls(version, root, dirs, data)

    def find(self, tag: str, number: int = 1) -> Optional[AbifDir]:
        for d in self.dirs:
            if d.tag == tag and d.number == number:
                return d
        return None

    def read_block(self, d: AbifDir) -> bytes:
        return bytes(self.blob[d.data_offset : d.data_offset + d.data_size])

# CycS helpers

def active_bits_to_text(v: int) -> str:
    if v == 0:
        return ""
    out = []
    for bit, label in ACTIVE_BIT_LABELS:
        if v & bit:
            out.append(label)
    return "".join(out)

def active_text_to_bits(text: str) -> int:
    """Parse ACTIVE from CSV.
    - If the string is made ONLY of label chars (AGCT5678), treat it as a label mask.
      This makes '5' -> bit 16, '6' -> 32, etc.
    - Else, if it's purely digits (e.g., '255', '0', '16'), treat as numeric bitmask.
    - Else, accumulate any recognized label characters present.
    """
    if text is None:
        return 0
    t = str(text).strip()
    if t == "" or t in ("-", "None", "none"):
        return 0

    labels = [label for _, label in ACTIVE_BIT_LABELS]
    label_set = set(x.upper() for x in labels)
    char_to_bit = {label: bit for bit, label in ACTIVE_BIT_LABELS}

    if all(ch.upper() in label_set for ch in t):
        v = 0
        for ch in t.upper():
            v |= char_to_bit[ch]
        return v

    if t.isdigit():
        return int(t)

    v = 0
    for ch in t.upper():
        if ch in char_to_bit:
            v |= char_to_bit[ch]
    return v

def safe_to_text(v: int) -> str:
    return SAFE_TO_TEXT.get(int(v), str(v))

def text_to_safe(text: str) -> int:
    if text is None:
        return 0
    t = str(text).strip().lower()
    return TEXT_TO_SAFE.get(t, 1 if t in ("y","true","t") else 0)

def num_to_name(num: int) -> str:
    return NUM_TO_NAME.get(int(num), f"UNKNOWN {num}")

def read_cycs(dir_bytes: bytes) -> List[Tuple[int,int,int,int]]:
    out = []
    for i in range(0, len(dir_bytes), 8):
        if i + 8 > len(dir_bytes):
            break
        num, timex10, active, safe = struct.unpack(">4H", dir_bytes[i:i+8])
        out.append((num, timex10, active, safe))
    return out
```

```
def write_cycs(rows: Iterable[Tuple[int,int,int,int]]) -> bytes:
    buf = bytearray()
    for num, timex10, active, safe in rows:
        buf.extend(struct.pack(">4H", int(num), int(timex10), int(active), int(safe)))
    return bytes(buf)

# Public API

def decode_to_csv(abif_path: Path, csv_path: Path) -> None:
    abif = AbifFile.load(abif_path)
    cyc = abif.find("CycS", 1)
    if not cyc:
        raise RuntimeError("CycS:1 not found")
    data = abif.read_block(cyc)
    rows = read_cycs(data)

    out_rows: List[Dict[str, str]] = []
    for num, timex10, active, safe in rows:
        out_rows.append({
            "FUNCTION NAME": num_to_name(num),
            "NUM": str(num),
            "TIME": f"{timex10/10:.1f}",
            "ACTIVE": active_bits_to_text(active),
            "SAFE": safe_to_text(safe),
        })

    with csv_path.open("w", newline="", encoding="utf-8") as f:
        w = csv.DictWriter(f, fieldnames=["FUNCTION NAME","NUM","TIME","ACTIVE","SAFE"])
        w.writeheader()
        w.writerows(out_rows)

def _csv_to_rows(csv_path: Path) -> List[Tuple[int,int,int,int]]:
    rows_csv = []
    with csv_path.open("r", newline="", encoding="utf-8") as f:
        r = csv.DictReader(f)
        for i, row in enumerate(r, start=1):
            num_s = (row.get("NUM") or "").strip()
            if not num_s:
                fname = (row.get("FUNCTION NAME") or "").strip()
                inv = {v: k for k, v in NUM_TO_NAME.items()}
                if fname in inv:
                    num = inv[fname]
                else:
                    raise ValueError(f"Row {i}: NUM missing and FUNCTION NAME not recognized: {fname!r}")
            else:
                num = int(float(num_s))
            timex10 = int(round(float((row.get("TIME") or "0").strip()) * 10.0))
            active = active_text_to_bits(row.get("ACTIVE") or "")
            safe = text_to_safe(row.get("SAFE") or "No")
            rows_csv.append((num, timex10, active, safe))
    return rows_csv

def encode(csv_path: Path, out_path: Path) -> None:
    """Build a minimal ABIF containing only tdir + CycS.
    Layout:
      - Bytes 0..3: 'ABIF'
      - 4..5: version (101)
      - 6..33: root tdir (tdir:1) pointing to directory table at 128
      - 128..: directory table (32 entries, 28 bytes each)
      - 1024..: CycS data payload (8 bytes * row_count)
    """
    rows = _csv_to_rows(csv_path)
    payload = write_cycs(rows)

    version = 101
    DIR_COUNT = 32
    DIR_OFFSET = 128
    DATA_OFFSET = 1024

    blob = bytearray()
    blob.extend(b"ABIF")
    blob.extend(struct.pack(">H", version))
```

```
    root = AbifDir("tdir", 1, 1023, 28, 1, DIR_COUNT*DIR_SIZE, DIR_OFFSET, 0)
    blob.extend(root.to_bytes())

    if len(blob) < DIR_OFFSET:
        blob.extend(b"\x00" * (DIR_OFFSET - len(blob)))

    dir_table = bytearray(DIR_COUNT * DIR_SIZE)
    cyc = AbifDir("CycS", 1, 1024, 8, len(payload)//8, len(payload), DATA_OFFSET, 0)
    dir_table[0:DIR_SIZE] = cyc.to_bytes()
    blob.extend(dir_table)

    if len(blob) < DATA_OFFSET:
        blob.extend(b"\x00" * (DATA_OFFSET - len(blob)))

    blob.extend(payload)
    out_path.write_bytes(blob)

# CLI

def _main(argv: List[str]) -> int:
    import sys
    if len(argv) < 2 or argv[1] not in ("decode","encode"):
        print("Usage:\n  decode <input_abif> <output.csv>\n  encode <input.csv> <output_abif>")
        return 2
    cmd = argv[1]
    if cmd == "decode":
        if len(argv) != 4:
            print("Usage: decode <input_abif> <output.csv>")
            return 2
        decode_to_csv(Path(argv[2]), Path(argv[3]))
        print(f"Wrote CSV to {argv[3]}")
        return 0
    if cmd == "encode":
        if len(argv) != 4:
            print("Usage: encode <input.csv> <output_abif>")
            return 2
        encode(Path(argv[2]), Path(argv[3]))
        print(f"Wrote ABIF to {argv[3]}")
        return 0
    return 0

if __name__ == "__main__":
    import sys
    raise SystemExit(_main(sys.argv))
```

Extinction coefficient data processing

Fit Beer-Lambert Law function and plot the data (R)

```
library(ggplot2)
library(dplyr)
library(readr)
library(grid)

# Paths
file_path          <- "./input/data.csv"
export_pdf_path    <- "./output/extinction_coefficient.pdf"
export_csv_path    <- "./output/extinction_coefficient.csv"

# Style
LINE_WIDTH_PT      <- 0.5
TICK_LENGTH_MM     <- 0.72
TICK_LABEL_PAD_MM  <- 0.70
LABEL_TITLE_PAD_MM <- 1.00
PANEL_W_MM         <- 25
PANEL_H_MM         <- 25
X_LIMITS           <- c(0, 0.15)
Y_LIMITS           <- c(0, 1.5)

# Load data
dat <- read_csv(file_path)
```

```r
dat$sample <- factor(dat$sample, levels = unique(dat$sample))

# Fit linear models A = εlc (with l = 1 cm) for each sample
fit_one <- function(df) lm(A ~ c - 1, data = df)
fits    <- lapply(split(dat, dat$sample), fit_one)

# Extract extinction coefficients and standard errors (units: (M^-1)(cm^-1))
results <- bind_rows(lapply(names(fits), function(s){
  fit <- fits[[s]]
  coef_summary <- summary(fit)$coefficients
  tibble(sample = s, ε = round(coef_summary[1, "Estimate"], 0), se = round(coef_summary[1, "Std. Error"], 0))
}))
write_csv(results, export_csv_path)

# Generate lines for plotting across full axis limits
x_grid_mM <- seq(X_LIMITS[1], X_LIMITS[2], length.out = 200)
preds <- bind_rows(lapply(names(fits), function(s){
  tibble(sample = factor(s, levels = levels(dat$sample)), c_mM = x_grid_mM,
         pred = as.numeric(predict(fits[[s]], newdata = data.frame(c = x_grid_mM / 1000))))
}))

# Convert concentration in data to mM for plotting
dat <- dat |> mutate(c_mM = c * 1000)

# Plot
p <- ggplot() +
  geom_line(data = preds, aes(x = c_mM, y = pred, colour = sample), linewidth = LINE_WIDTH_PT * 0.752592) +
  geom_point(data = dat, aes(x = c_mM, y = A, fill = sample),
             size = 1.2, shape = 21, stroke = LINE_WIDTH_PT * 0.70423, colour = "#000000") +
  scale_x_continuous(limits = X_LIMITS, breaks = function(lims) pretty(lims, n = 4), expand = expansion(mult = 0)) +
  scale_y_continuous(limits = Y_LIMITS, breaks = function(lims) pretty(lims, n = 4), expand = expansion(mult = 0)) +
  labs(x = "Concentration (mM)", y = "Absorbance") +
  theme_classic(base_size = 6) +
  theme(axis.text.x       = element_text(size = 6, margin = margin(t = TICK_LABEL_PAD_MM, unit = "mm")),
        axis.text.y       = element_text(size = 6, margin = margin(r = TICK_LABEL_PAD_MM, unit = "mm")),
        axis.title.x      = element_text(size = 6, margin = margin(t = LABEL_TITLE_PAD_MM, unit = "mm")),
        axis.title.y      = element_text(size = 6, margin = margin(r = LABEL_TITLE_PAD_MM, unit = "mm")),
        legend.text       = element_text(size = 6),
        axis.line         = element_line(linewidth = LINE_WIDTH_PT * 0.47037),
        axis.ticks        = element_line(linewidth = LINE_WIDTH_PT * 0.47037),
        axis.ticks.length = unit(TICK_LENGTH_MM, "mm"),
        legend.key.size   = unit(3, "mm"))

# Export the plot to PDF
save_pdf <- function(p, path) {
  g <- ggplotGrob(p)
  g$widths [g$layout$l[g$layout$name == "panel"]] <- unit(PANEL_W_MM, "mm")
  g$heights[g$layout$t[g$layout$name == "panel"]] <- unit(PANEL_H_MM, "mm")
  ggsave(path, g, width = 180, height = 225, units = "mm", device = "pdf")
}
save_pdf(p, export_pdf_path); print(p)
```

14. Supplementary cycle method

FUNCTION NAME	NUM	TIME	ACTIVE	SAFE
Begin	106	0		Yes
18 to Waste	64	3	AGCT5678	Yes
18 to Column	42	10	AGCT5678	Yes
Reverse flush	2	10	AGCT5678	Yes
Block flush	1	4	AGCT5678	Yes
Phos Prep	101	3	AGCT5678	Yes
Column 1 On	140	0	AGCT5678	Yes
Block Vent	111	2	AGCT5678	Yes
5 to Waste	54	1.7		Yes
Tet to Column	34	0.5	AGCT5678	Yes
5 to Column	29	0.5	AGCT5678	Yes
Tet to Column	34	0.5	AGCT5678	Yes
5 to Column	29	0.5	AGCT5678	Yes
Tet to Column	34	0.5	AGCT5678	Yes
5 to Column	29	0.5	AGCT5678	Yes
Tet to Column	34	0.5	AGCT5678	Yes

5 to Column	29	0.5	AGCT5678	Yes
Tet to Column	34	0.5	AGCT5678	Yes
5 to Column	29	0.5	AGCT5678	Yes
Tet to Column	34	0.5	AGCT5678	Yes
5 to Column	29	0.5	AGCT5678	Yes
Tet to Column	34	0.5	AGCT5678	Yes
5 to Column	29	0.5	AGCT5678	Yes
Tet to Column	34	0.5	AGCT5678	Yes
5 to Column	29	0.5	AGCT5678	Yes
Wait	103	30	AGCT5678	Yes
Tet to Column	34	0.5	AGCT5678	Yes
5 to Column	29	0.5	AGCT5678	Yes
Tet to Column	34	0.5	AGCT5678	Yes
5 to Column	29	0.5	AGCT5678	Yes
Tet to Column	34	0.5	AGCT5678	Yes
5 to Column	29	0.5	AGCT5678	Yes
Tet to Column	34	0.5	AGCT5678	Yes
5 to Column	29	0.5	AGCT5678	Yes
Tet to Column	34	0.5	AGCT5678	Yes
5 to Column	29	0.5	AGCT5678	Yes
Tet to Column	34	0.5	AGCT5678	Yes
5 to Column	29	0.5	AGCT5678	Yes
Tet to Column	34	0.5	AGCT5678	Yes
5 to Column	29	0.5	AGCT5678	Yes
Tet to Column	34	0.5	AGCT5678	Yes
5 to Column	29	0.5	AGCT5678	Yes
Column 1 Off	141	0	AGCT5678	Yes
Column 2 On	142	0	AGCT5678	Yes
18 to Waste	64	4	AGCT5678	Yes
Block flush	1	3	AGCT5678	Yes
Block Vent	111	2	AGCT5678	Yes
5 to Waste	54	1.7		Yes
Tet to Column	34	0.5	AGCT5678	Yes
5 to Column	29	0.5	AGCT5678	Yes
Tet to Column	34	0.5	AGCT5678	Yes
5 to Column	29	0.5	AGCT5678	Yes
Tet to Column	34	0.5	AGCT5678	Yes
5 to Column	29	0.5	AGCT5678	Yes
Tet to Column	34	0.5	AGCT5678	Yes
5 to Column	29	0.5	AGCT5678	Yes
Tet to Column	34	0.5	AGCT5678	Yes
5 to Column	29	0.5	AGCT5678	Yes
Tet to Column	34	0.5	AGCT5678	Yes
5 to Column	29	0.5	AGCT5678	Yes
Tet to Column	34	0.5	AGCT5678	Yes
5 to Column	29	0.5	AGCT5678	Yes
Tet to Column	34	0.5	AGCT5678	Yes
5 to Column	29	0.5	AGCT5678	Yes
Wait	103	30	AGCT5678	Yes
Tet to Column	34	0.5	AGCT5678	Yes
5 to Column	29	0.5	AGCT5678	Yes
Tet to Column	34	0.5	AGCT5678	Yes
5 to Column	29	0.5	AGCT5678	Yes
Tet to Column	34	0.5	AGCT5678	Yes
5 to Column	29	0.5	AGCT5678	Yes
Tet to Column	34	0.5	AGCT5678	Yes
5 to Column	29	0.5	AGCT5678	Yes
Tet to Column	34	0.5	AGCT5678	Yes
5 to Column	29	0.5	AGCT5678	Yes
Tet to Column	34	0.5	AGCT5678	Yes
5 to Column	29	0.5	AGCT5678	Yes
Tet to Column	34	0.5	AGCT5678	Yes
5 to Column	29	0.5	AGCT5678	Yes
Tet to Column	34	0.5	AGCT5678	Yes
5 to Column	29	0.5	AGCT5678	Yes
Column 2 Off	143	0	AGCT5678	Yes
Column 3 On	144	0	AGCT5678	Yes
18 to Waste	64	4	AGCT5678	Yes
Block flush	1	3	AGCT5678	Yes
Block Vent	111	2	AGCT5678	Yes
5 to Waste	54	1.7		Yes
Tet to Column	34	0.5	AGCT5678	Yes
5 to Column	29	0.5	AGCT5678	Yes
Tet to Column	34	0.5	AGCT5678	Yes

5 to Column	29	0.5	AGCT5678	Yes
Tet to Column	34	0.5	AGCT5678	Yes
5 to Column	29	0.5	AGCT5678	Yes
Tet to Column	34	0.5	AGCT5678	Yes
5 to Column	29	0.5	AGCT5678	Yes
Tet to Column	34	0.5	AGCT5678	Yes
5 to Column	29	0.5	AGCT5678	Yes
Tet to Column	34	0.5	AGCT5678	Yes
5 to Column	29	0.5	AGCT5678	Yes
Tet to Column	34	0.5	AGCT5678	Yes
5 to Column	29	0.5	AGCT5678	Yes
Tet to Column	34	0.5	AGCT5678	Yes
5 to Column	29	0.5	AGCT5678	Yes
Wait	103	30	AGCT5678	Yes
Tet to Column	34	0.5	AGCT5678	Yes
5 to Column	29	0.5	AGCT5678	Yes
Tet to Column	34	0.5	AGCT5678	Yes
5 to Column	29	0.5	AGCT5678	Yes
Tet to Column	34	0.5	AGCT5678	Yes
5 to Column	29	0.5	AGCT5678	Yes
Tet to Column	34	0.5	AGCT5678	Yes
5 to Column	29	0.5	AGCT5678	Yes
Tet to Column	34	0.5	AGCT5678	Yes
5 to Column	29	0.5	AGCT5678	Yes
Tet to Column	34	0.5	AGCT5678	Yes
5 to Column	29	0.5	AGCT5678	Yes
Tet to Column	34	0.5	AGCT5678	Yes
5 to Column	29	0.5	AGCT5678	Yes
Tet to Column	34	0.5	AGCT5678	Yes
5 to Column	29	0.5	AGCT5678	Yes
Column 3 Off	145	0	AGCT5678	Yes
Column 4 On	146	0	AGCT5678	Yes
18 to Waste	64	4	AGCT5678	Yes
Block flush	1	3	AGCT5678	Yes
Block Vent	111	2	AGCT5678	Yes
5 to Waste	54	1.7		Yes
Tet to Column	34	0.5	AGCT5678	Yes
5 to Column	29	0.5	AGCT5678	Yes
Tet to Column	34	0.5	AGCT5678	Yes
5 to Column	29	0.5	AGCT5678	Yes
Tet to Column	34	0.5	AGCT5678	Yes
5 to Column	29	0.5	AGCT5678	Yes
Tet to Column	34	0.5	AGCT5678	Yes
5 to Column	29	0.5	AGCT5678	Yes
Tet to Column	34	0.5	AGCT5678	Yes
5 to Column	29	0.5	AGCT5678	Yes
Tet to Column	34	0.5	AGCT5678	Yes
5 to Column	29	0.5	AGCT5678	Yes
Tet to Column	34	0.5	AGCT5678	Yes
5 to Column	29	0.5	AGCT5678	Yes
Tet to Column	34	0.5	AGCT5678	Yes
5 to Column	29	0.5	AGCT5678	Yes
Wait	103	30	AGCT5678	Yes
Tet to Column	34	0.5	AGCT5678	Yes
5 to Column	29	0.5	AGCT5678	Yes
Tet to Column	34	0.5	AGCT5678	Yes
5 to Column	29	0.5	AGCT5678	Yes
Tet to Column	34	0.5	AGCT5678	Yes
5 to Column	29	0.5	AGCT5678	Yes
Tet to Column	34	0.5	AGCT5678	Yes
5 to Column	29	0.5	AGCT5678	Yes
Tet to Column	34	0.5	AGCT5678	Yes
5 to Column	29	0.5	AGCT5678	Yes
Tet to Column	34	0.5	AGCT5678	Yes
5 to Column	29	0.5	AGCT5678	Yes
Tet to Column	34	0.5	AGCT5678	Yes
5 to Column	29	0.5	AGCT5678	Yes
Tet to Column	34	0.5	AGCT5678	Yes
5 to Column	29	0.5	AGCT5678	Yes
Column 4 Off	147	0	AGCT5678	Yes
18 to Waste	64	4		Yes
Wait	103	300	AGCT5678	Yes
18 to Waste	64	4		Yes
Block flush	1	3	AGCT5678	Yes

Phos Prep	101	3		Yes
Column 1 On	140	0		Yes
Block Vent	111	2	AGCT5678	Yes
A to Column	25	8	A	Yes
G to Column	26	8	G	Yes
C to Column	27	8	C	Yes
T to Column	28	8	T	Yes
5 to Column	29	8	5	Yes
6 to Column	30	8	6	Yes
7 to Column	31	8	7	Yes
8 to Column	32	8	8	Yes
Wait	103	60	AGCT5678	Yes
A to Column	25	8	A	Yes
G to Column	26	8	G	Yes
C to Column	27	8	C	Yes
T to Column	28	8	T	Yes
5 to Column	29	8	5	Yes
6 to Column	30	8	6	Yes
7 to Column	31	8	7	Yes
8 to Column	32	8	8	Yes
Column 1 Off	141	0		Yes
Column 2 On	142	0		Yes
18 to Waste	64	4		Yes
Block flush	1	3	AGCT5678	Yes
Block Vent	111	2	AGCT5678	Yes
A to Column	25	8	A	Yes
G to Column	26	8	G	Yes
C to Column	27	8	C	Yes
T to Column	28	8	T	Yes
5 to Column	29	8	5	Yes
6 to Column	30	8	6	Yes
7 to Column	31	8	7	Yes
8 to Column	32	8	8	Yes
Wait	103	60	AGCT5678	Yes
A to Column	25	8	A	Yes
G to Column	26	8	G	Yes
C to Column	27	8	C	Yes
T to Column	28	8	T	Yes
5 to Column	29	8	5	Yes
6 to Column	30	8	6	Yes
7 to Column	31	8	7	Yes
8 to Column	32	8	8	Yes
Column 2 Off	143	0		Yes
Column 3 On	144	0		Yes
18 to Waste	64	4		Yes
Block flush	1	3	AGCT5678	Yes
Block Vent	111	2	AGCT5678	Yes
A to Column	25	8	A	Yes
G to Column	26	8	G	Yes
C to Column	27	8	C	Yes
T to Column	28	8	T	Yes
5 to Column	29	8	5	Yes
6 to Column	30	8	6	Yes
7 to Column	31	8	7	Yes
8 to Column	32	8	8	Yes
Wait	103	60	AGCT5678	Yes
A to Column	25	8	A	Yes
G to Column	26	8	G	Yes
C to Column	27	8	C	Yes
T to Column	28	8	T	Yes
5 to Column	29	8	5	Yes
6 to Column	30	8	6	Yes
7 to Column	31	8	7	Yes
8 to Column	32	8	8	Yes
Column 3 Off	145	0		Yes
Column 4 On	146	0		Yes
18 to Waste	64	4		Yes
Block flush	1	3	AGCT5678	Yes
Block Vent	111	2	AGCT5678	Yes
A to Column	25	8	A	Yes
G to Column	26	8	G	Yes
C to Column	27	8	C	Yes
T to Column	28	8	T	Yes
5 to Column	29	8	5	Yes

6 to Column	30	8	6	Yes
7 to Column	31	8	7	Yes
8 to Column	32	8	8	Yes
Wait	103	60	AGCT5678	Yes
A to Column	25	8	A	Yes
G to Column	26	8	G	Yes
C to Column	27	8	C	Yes
T to Column	28	8	T	Yes
5 to Column	29	8	5	Yes
6 to Column	30	8	6	Yes
7 to Column	31	8	7	Yes
8 to Column	32	8	8	Yes
Column 4 Off	147	0		Yes
18 to Waste	64	4		Yes
Wait	103	300	AGCT5678	Yes
18 to Waste	64	4		Yes
Block flush	1	3	AGCT5678	Yes
Phos Prep	101	3		Yes
Column 1 On	140	0		Yes
Block Vent	111	2	AGCT5678	Yes
A to Column	25	8	A	Yes
G to Column	26	8	G	Yes
C to Column	27	8	C	Yes
T to Column	28	8	T	Yes
5 to Column	29	8	5	Yes
6 to Column	30	8	6	Yes
7 to Column	31	8	7	Yes
8 to Column	32	8	8	Yes
Column 1 Off	141	0		Yes
Column 2 On	142	0		Yes
18 to Waste	64	4		Yes
Block flush	1	3	AGCT5678	Yes
Block Vent	111	2	AGCT5678	Yes
A to Column	25	8	A	Yes
G to Column	26	8	G	Yes
C to Column	27	8	C	Yes
T to Column	28	8	T	Yes
5 to Column	29	8	5	Yes
6 to Column	30	8	6	Yes
7 to Column	31	8	7	Yes
8 to Column	32	8	8	Yes
Column 2 Off	143	0		Yes
Column 3 On	144	0		Yes
18 to Waste	64	4		Yes
Block flush	1	3	AGCT5678	Yes
Block Vent	111	2	AGCT5678	Yes
A to Column	25	8	A	Yes
G to Column	26	8	G	Yes
C to Column	27	8	C	Yes
T to Column	28	8	T	Yes
5 to Column	29	8	5	Yes
6 to Column	30	8	6	Yes
7 to Column	31	8	7	Yes
8 to Column	32	8	8	Yes
Column 3 Off	145	0		Yes
Column 4 On	146	0		Yes
18 to Waste	64	4		Yes
Block flush	1	3	AGCT5678	Yes
Block Vent	111	2	AGCT5678	Yes
A to Column	25	8	A	Yes
G to Column	26	8	G	Yes
C to Column	27	8	C	Yes
T to Column	28	8	T	Yes
5 to Column	29	8	5	Yes
6 to Column	30	8	6	Yes
7 to Column	31	8	7	Yes
8 to Column	32	8	8	Yes
Column 4 Off	147	0		Yes
18 to Waste	64	4		Yes
Wait	103	750	AGCT5678	Yes
Wait	103	750	AGCT5678	Yes
Reverse flush	2	7	AGCT5678	Yes
Block flush	1	3	AGCT5678	Yes
18 to Waste	64	4		Yes

18 to Column	42	10	AGCT5678	Yes
Reverse flush	2	7	AGCT5678	Yes
Block flush	1	3	AGCT5678	Yes
15 to Column	41	30	AGCT5678	Yes
18 to Waste	64	4		Yes
Block flush	1	3	AGCT5678	Yes
Wait	103	45	AGCT5678	Yes
18 to Column	42	8	AGCT5678	Yes
Flush to Waste	4	6	AGCT5678	Yes
18 to Column	42	8	AGCT5678	Yes
Reverse flush	2	7	AGCT5678	Yes
18 to Column	42	8	AGCT5678	Yes
Reverse flush	2	5	AGCT5678	Yes
Block flush	1	3	AGCT5678	Yes
Start Detrityl	105	0	AGCT5678	Yes
18 to Waste	64	4	AGCT5678	Yes
18 to Column	42	10	AGCT5678	Yes
Reverse flush	2	7	AGCT5678	Yes
Block flush	1	3	AGCT5678	Yes
If Monitoring	167	0		Yes
19 to Column	44	35	AGCT5678	Yes
14 to Column	40	3	AGCT5678	No
Monitor Trityls	135	0	AGCT5678	No
14 to Column	40	50	AGCT5678	No
Monitor noise	136	0	AGCT5678	No
14 to Column	40	10	AGCT5678	No
Stop monitor	137	0	AGCT5678	No
18 to Column	42	10	AGCT5678	No
Reverse flush	2	8	AGCT5678	No
If not Monitoring	168	0		Yes
14 to Column	40	6	AGCT5678	No
Trityl Flush	3	5	CT5678	No
14 to Column	40	6	AGCT5678	No
Wait	103	5	AGCT5678	No
Trityl Flush	3	5	AGCT5678	Yes
14 to Column	40	6	AGCT5678	Yes
Wait	103	5	AGCT5678	Yes
Trityl Flush	3	5	AGCT5678	Yes
18 to Column	42	10	AGCT5678	No
Trityl Flush	3	8	AGCT5678	Yes
End Monitoring	169	0		Yes
18 to Column	42	8	AGCT5678	Yes
Reverse flush	2	7	AGCT5678	Yes
Block flush	1	4	AGCT5678	Yes

15. Molar extinction calculation for the 4,4'-dimethoxytrityl (DMT) group

To calculate the molar extinction coefficient, we prepared solutions of DMT-dT and thymidine at various concentrations and measured their UV absorbance at 260 nm, as listed in the table below. All measurements were performed using dry methanol as the solvent.

Sample	Replicate	Concentration (M)	Absorbance
thymidine	1	0.0001	0.9402
thymidine	1	0.00005	0.4959
thymidine	1	0.000025	0.2389
thymidine	2	0.0001	1.2027
thymidine	2	0.00005	0.6224
thymidine	2	0.000025	0.3002
thymidine	3	0.0001	1.107
thymidine	3	0.00005	0.5715
thymidine	3	0.000025	0.2747
DMT-thymidine	1	0.0001	0.9231
DMT-thymidine	1	0.00005	0.4902
DMT-thymidine	1	0.000025	0.2369
DMT-thymidine	2	0.0001	1.1594
DMT-thymidine	2	0.00005	0.6196
DMT-thymidine	2	0.000025	0.3034
DMT-thymidine	3	0.0001	1.10583
DMT-thymidine	3	0.00005	0.5689
DMT-thymidine	3	0.000025	0.2674

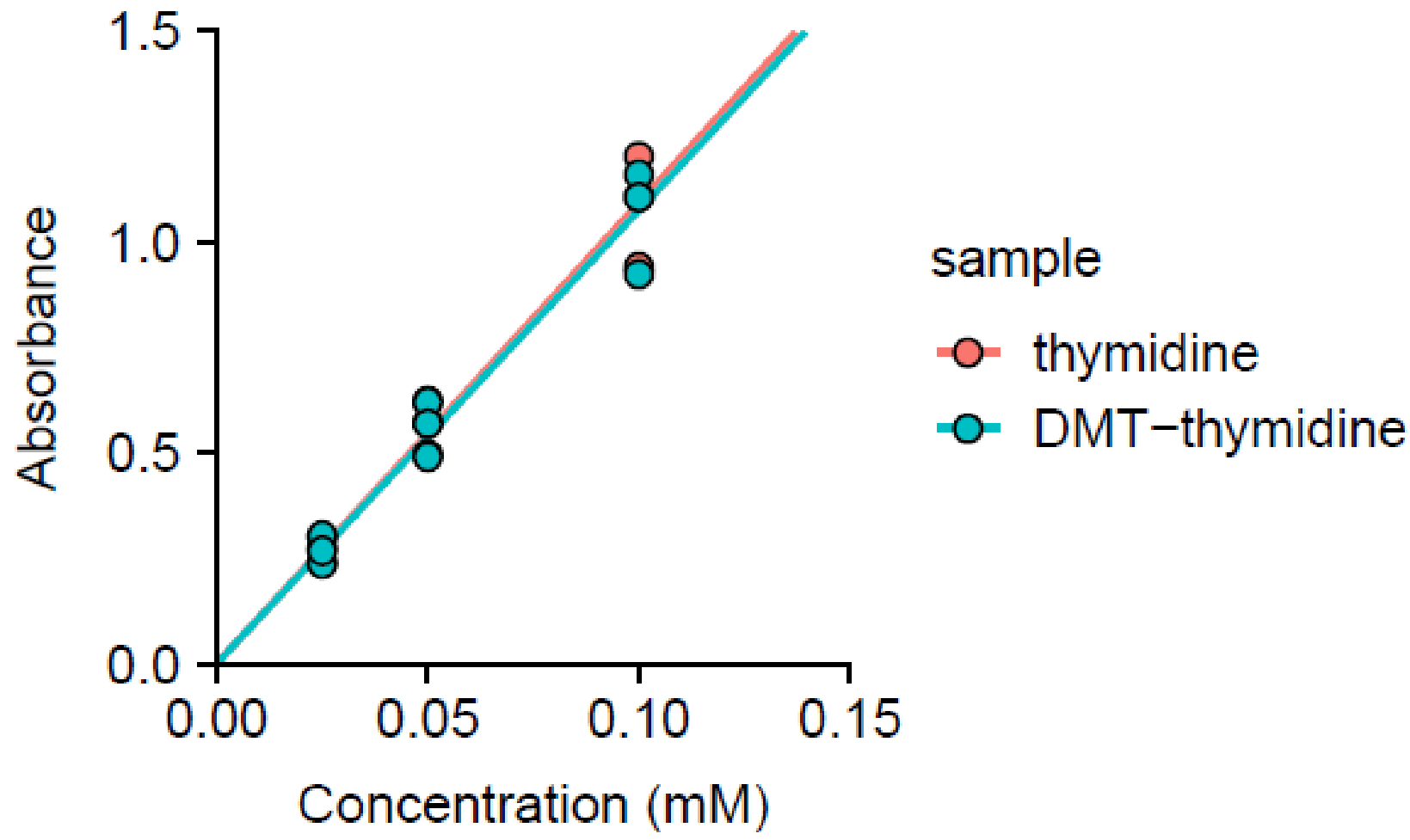

A graph was plotted with absorbance on the Y-axis and concentration (mM) on the X-axis. The molar extinction coefficient was calculated by fitting the Beer-Lambert equation to the experimental data using custom R script (see Supplementary Scripts section).

**Sample**	**ε (M^{-1}*cm^{-1})**
thymidine	10916 ± 384
DMT-thymidine	10742 ± 371

16. References

1 Hall, L. M., Gerowska, M. & Brown, T. A highly fluorescent DNA toolkit: synthesis and properties of oligonucleotides containing new Cy3, Cy5 and Cy3B monomers. *Nucleic Acids Research* **40**, e108–e108 (2012). https://doi.org/10.1093/nar/gks303

2 Dhara, D. *et al.* Synthesis, Biophysical and Biological Evaluation of Splice-Switching Oligonucleotides with Multiple LNA-Phosphothiotriester Backbones. *Journal of the American Chemical Society* **146**, 29773–29781 (2024). https://doi.org/10.1021/jacs.4c11402

3 Taemaitree, L., Shivalingam, A., El-Sagheer, A. H. & Brown, T. in *CRISPR Guide RNA Design: Methods and Protocols* (eds Tudor A. Fulga, David J. H. F. Knapp, & Quentin R. V. Ferry) 61–78 (Springer US, 2021). https://doi.org/10.1007/978-1-0716-0687-2_5
