# Supplementary material for: A versatile strategy for oligonucleotide functionalization via on-support phosphitylation
Source: Nat Chem. 2026 Jul 22;18(10):1747–54. doi: 10.1038/s41557-026-02214-6 (PMC13623645; doi:10.1038/s41557-026-02214-6)
Supplement: Supplementary file 3 [file 41557_2026_2214_MOESM3_ESM.pdf]

nature portfolio

Peer Review File

A versatile strategy for oligonucleotide functionalization via on-support phosphitylation

Corresponding Author: Professor Tom Brown

This file contains all reviewer reports in order by version, followed by all author rebuttals in order by version.

Version 0:

Reviewer comments:

Reviewer #1

(Comments for the Author)
The authors described a very simple and clever strategy for modifying one end of an oligonucleotide during the final step of solid-phase synthesis. It is even surprising that it was not explored earlier. The key step is the reaction between the terminal hydroxyl group with 2-Cyanoethyl N,N,N',N'-Tetraisopropylphosphordiamidite, which produces the 5'/3'-O-phosphoramidite of the oligonucleotide. This phosphoramidite can then react with alcohols, including nucleosides. The main advantage of this approach is that I uses stable alcohols instead of unstable phosphoramidites to functionalize the oligonucleotide terminus. The proposed method is compatible with a wide range of alcohols, including bifunctional compounds with e.g. azides, alkynes, alkenes, thiols, and even unprotected primary amines. The utility of this synthetic strategy was demonstrated by synthesizing a therapeutically relevant oligonucleotide – a 5'-azide derivative of MALAT1 Gapmer. After labeling with AF488 via SPAAC, it was used to visualize ASO in HeLa cells by fluorescence microscopy. The choice of this model ASO highlights the the ability to combine azido modification with phosphorothioate linkages, which alternative approaches struggle to achieve.

Given the simplicity and robustness of the method, as well as the scope of modifications that can be introduced into various types of oligonucleotides, I expect it to have a significant impact on the field of oligonucleotide synthesis.

From a technical standpoint, the manuscript is very well-written: the introduction shows the importance of the work and its context, especially in the field of therapeutic oligonucleotides; the story is concise and easy to follow; the abstract is sufficiently informative. The quality of the data is very high, and it is presented sufficiently for reproduction.

I have only few comments and questions that should be addressed before I can recommend publication.

1. A large excess of the phosphitylating agent and alcohol is used: 200-300 eqv for CPG and 40-50 eqv for PS. Have you tested a lower excess, which could be important for scaling up the synthesis? The classical phosphoramidite approach uses a very low excess of phosphoramidites, many of which (e.g. 6-aminohexyl, 5-hexynyl) are rather stable and inexpensive. Please comment on this in the manuscript.
2. Is tetrazole necessary for the reaction of oligonucleotide phosphoramidite with alcohols, or does BTT work as well?
3. Please include more experimental details in the table in paragraph 4 of the Supporting Information, such as coupling reaction time, alcohol excess, and temperature.
4. The reaction of oligonucleotide phosphoramidite on a highly loaded PS support with hexaethylene glycol produced a significant amount of dimeric product. Please compare the product distribution using CPG support or PS with lower loading. Is it possible to avoid or at least minimize the di-functionalized product by using an even higher excess of alcohol?

Reviewer #2

(Comments for the Author)
The authors describe on a a straightforward, cost-effective method for oligonucleotide modification via on-support oligonucleotide phosphitylation / in situ nucleophilic functionalization using readily available P(III) phosphitylating reagents. The authors' findings are expected to contribute to the advancement of oligonucleotide chemistry-based technologies. The manuscript is well executed and the data appear robust, with no major technical concerns.

Nevertheless, the advance reported here is largely incremental, and the implications for the broader scientific community are somewhat limited. Given the journal's emphasis on work of exceptional conceptual novelty and wide interest, I do not believe this study is a strong fit for the journal.

Reviewer #3

(Comments for the Author)
This manuscript presents an innovative and practical approach for site-selective functionalization of oligonucleotides via on-support phosphorylation and in situ coupling with stable alcohols or nucleosides. The work addresses significant limitations associated with conventional phosphoramidite-based synthesis, including monomer instability, cost, and limited functional-group tolerance. The methodology is validated across diverse substrates, functional groups, and synthetic directions, and its utility is convincingly demonstrated through the synthesis of a biologically relevant azide-modified antisense oligonucleotide followed by cellular imaging studies. Overall, the manuscript is well written, the experimental results are convincing, and the strategy has clear potential to impact oligonucleotide therapeutic development and chemical biology. In this regard, the work should be of broad interest to researchers working in nucleic acid chemistry, chemical biology, and oligonucleotide therapeutics. However, while the methodology is promising, several aspects would benefit from clarification or further strengthening:
1.Although the authors demonstrate modification at multiple positions, the practical limitations of the method are not clearly defined. Providing guidance on substrate classes or functional groups that perform poorly or are incompatible would help readers better evaluate the practical applicability of the strategy.
2.Quantitative comparison with existing modification strategies would strengthen the practical positioning of the method. In particular, discussion of efficiency, scalability, or workflow advantages relative to phosphoramidite-based approaches would help clarify when the present method offers decisive benefits.
3.On line 53, the authors state that their strategy enables "site-specific terminal and internal oligonucleotide modification." However, the experimental evidence largely demonstrates terminal modification. The only example of internal modification relies on a diol substrate (Entry 19), which inherently generates symmetric sequences and therefore cannot be generalized for arbitrary internal modification. Consequently, the claim regarding general internal modification appears overstated and should be moderated or further supported.
4.In lines 69–74, the authors state that conventional phosphoramidite chemistry is "poorly tolerant of certain functional groups." To better highlight the advance of the present method, the authors should clarify the mechanistic origin of these incompatibilities and explicitly explain how the current strategy circumvents these limitations.
5.Since the present strategy focuses on oligonucleotide modification rather than oligonucleotide synthesis, comparison with phosphoramidite monomer synthesis methods is not entirely appropriate. A more informative comparison would be with established strategies specifically developed for terminal or post-synthetic oligonucleotide modification.
6.In Figure 4, the depicted structures for the coupling products of 1e with phosphorodiamidite 6a (or 6b) appear inconsistent with the expected products. The authors are encouraged to carefully re-examine and correct these structures if necessary.
7.The current study demonstrates successful incorporation of various alcohol substrates. Exploring whether other nucleophiles, such as thiols, phenols, or amines, could also be accommodated would further broaden the utility of the method. Even preliminary data or discussion would be valuable.
8.In the experiments evaluating terminal nucleobase tolerance (Entries 1–4), only poly-T sequences are employed, whereas more structurally complex sequences appear only in later examples involving uracil termini. Inclusion of more sequence-diverse examples in these initial tests would strengthen confidence in the generality of the approach.
9.If the system can tolerate more complex delivery or targeting motifs, such as lipid-derived functionalities, inclusion of such examples would further enhance the translational relevance of the method.
10.Phosphorodiamidate morpholino oligomers (PMOs) represent another important therapeutic oligonucleotide backbone. It remains unclear whether the present methodology is applicable to PMO systems. Discussion or preliminary experiments addressing this point would substantially increase the practical value of the study.

Version 1:

Reviewer comments:

Reviewer #1

(Comments for the Author)
The authors addressed all of my comments in a very satisfactory manner. I appreciate the additional experiments added during the revision process, which help better assess the scope and limitations of the methodology. I recommend publishing the manuscript in its current form.

(Remarks on code availability)

Reviewer #3

(Comments for the Author)
The authors have adequately addressed all of my previous concerns, and the manuscript has been significantly improved. I now support publication in Nature Chemistry.

(Remarks on code availability)

Open Access This Peer Review File is licensed under a Creative Commons Attribution 4.0 International License, which permits use, sharing, adaptation, distribution and reproduction in any medium or format, as long as you give appropriate credit to the original author(s) and the source, provide a link to the Creative Commons license, and indicate if changes were made.
In cases where reviewers are anonymous, credit should be given to 'Anonymous Referee' and the source.
The images or other third party material in this Peer Review File are included in the article's Creative Commons license, unless indicated otherwise in a credit line to the material. If material is not included in the article's Creative Commons license and your intended use is not permitted by statutory regulation or exceeds the permitted use, you will need to obtain permission directly from the copyright holder.
To view a copy of this license, visit https://creativecommons.org/licenses/by/4.0/

Prof. Tom Brown
Professor of Nucleic Acid Chemistry
Department of chemistry, University of Oxford, UK.
Chemistry Research Laboratory,
University of Oxford, 12 Mansfield Road, Oxford, OX1 3TA, U.K

Reviewer #1 (Comments for the Author):

Comments: The authors described a very simple and clever strategy for modifying one end of an oligonucleotide during the final step of solid-phase synthesis. It is even surprising that it was not explored earlier. The key step is the reaction between the terminal hydroxyl group with 2-Cyanoethyl N,N,N',N'-Tetraisopropylphosphordiamidite, which produces the 5'/3'-O-phosphoramidite of the oligonucleotide. This phosphoramidite can then react with alcohols, including nucleosides. The main advantage of this approach is that uses stable alcohols instead of unstable phosphoramidites to functionalize the oligonucleotide terminus. The proposed method is compatible with a wide range of alcohols, including bifunctional compounds with e.g. azides, alkynes, alkenes, thiols, and even unprotected primary amines. The utility of this synthetic strategy was demonstrated by synthesizing a therapeutically relevant oligonucleotide – a 5'-azide derivative of MALAT1 Gapmer. After labeling with AF488 via SPAAC, it was used to visualize ASO in HeLa cells by fluorescence microscopy. The choice of this model ASO highlights the ability to combine azido modification with phosphorothioate linkages, which alternative approaches struggle to achieve.
Given the simplicity and robustness of the method, as well as the scope of modifications that can be introduced into various types of oligonucleotides, I expect it to have a significant impact on the field of oligonucleotide synthesis.
From a technical standpoint, the manuscript is very well-written: the introduction shows the importance of the work and its context, especially in the field of therapeutic oligonucleotides; the story is concise and easy to follow; the abstract is sufficiently informative. The quality of the data is very high, and it is presented sufficiently for reproduction.
I have only few comments and questions that should be addressed before I can recommend publication.

Response: We thank the referee for their constructive and supportive comments, as well as their valuable suggestions. The manuscript has been carefully revised to address all points raised, and detailed responses are provided below.

Comments: A large excess of the phosphitylating agent and alcohol is used: 200-300 eqv for CPG and 40-50 eqv for PS. Have you tested a lower excess, which could be important for scaling up the synthesis? The classical phosphoramidite approach uses a very low excess of phosphoramidites, many of which (e.g. 6-aminohexyl, 5-hexynyl) are rather stable and inexpensive. Please comment on this in the manuscript.

Response: We appreciate the reviewer's comment. While phosphoramidites such as 6-aminohexyl or 5-hexynyl are inexpensive and do not require high excess, our initial protocol for manual synthesis (to which the Reviewer refers), and its further version for automated synthesis on ABI 394, were designed to ensure quantitative coupling on both CPG- and PS-supported sequences (for automated synthesis: CPG (1 μmol), 120 equiv. of phosphitylating agent, 150 equiv. of alcohol; PS (5.25 μmol), 22.9 equiv. of phosphitylating agent, 28.6 equiv. of alcohol. We have added these details concerning excesses used in the automated protocol to the Supplementary Information.
Additionally, we evaluated whether reduction of the excess phosphoramidite is possible to attain high conversion (Figure S1). To this end, we used a PS-supported mixed sequence to carry out all experiments (see Figure 2a; 5.25 μmol). We fixed the concentrations of phosphitylating agent (0.3

M) in acetonitrile, and decan-1-ol (0.25 M) with 1*H*-tetrazole (0.45M) in acetonitrile (premix together 1:1 v/v), and varied delivery times of these solutions on an ABI 394 synthesiser to test different excesses (please see the table and chromatograms below).

Entry	[P–N] (equiv.)	Decan-1-ol (equiv.)	Conversion (%)
1	22.9	28.6	92
2	17.1	28.6	91
3	11.4	28.6	88
4	22.9	21.4	86
5	22.9	14.3	76

[P–N] = 3-((bis(diisopropylamino)phosphino)oxy)propanenitrile

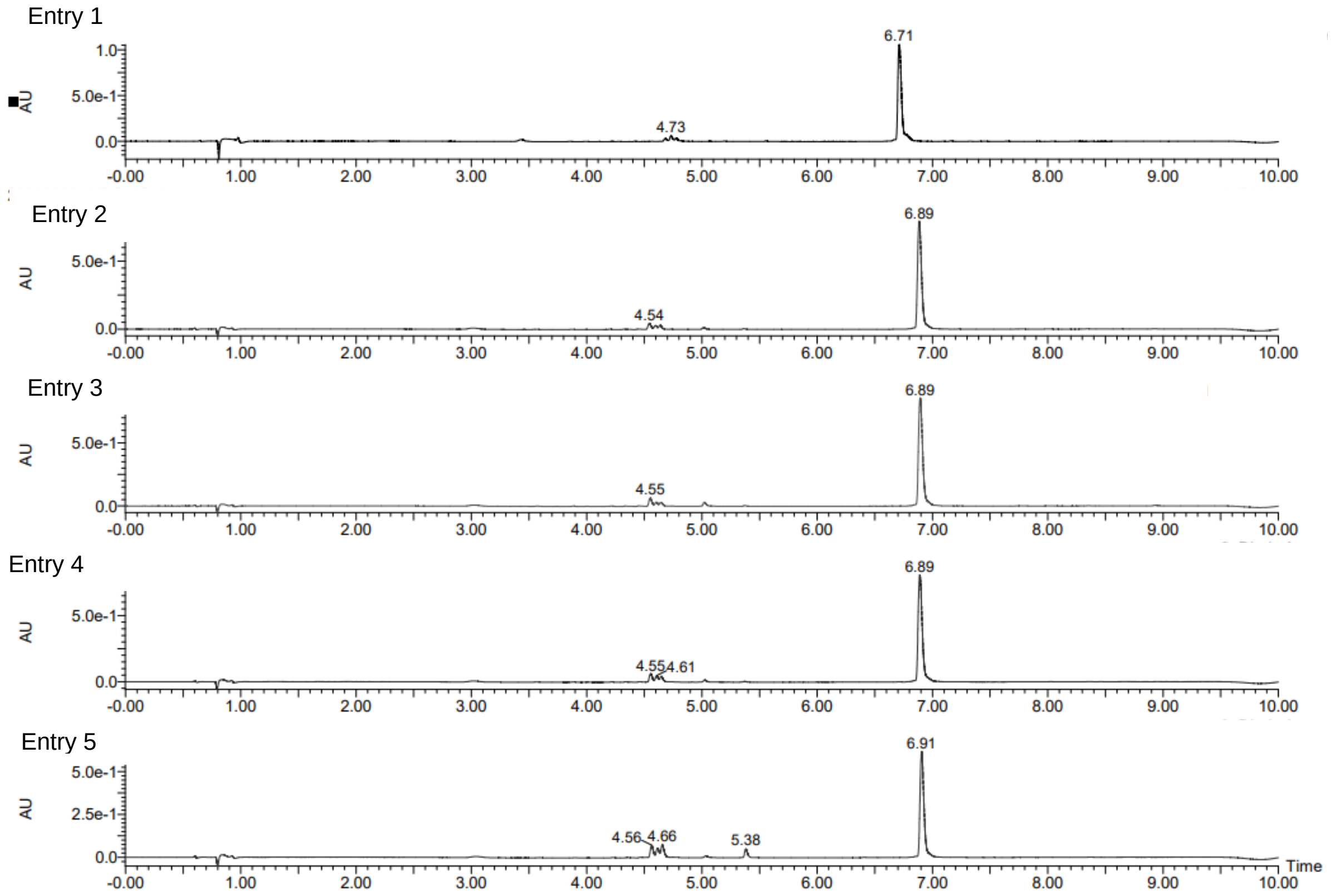

Figure S1. Stacked ion pair reversed-phase UHPLC chromatogram of the crude reaction mixture (UV absorbance at 260 nm *vs*. time in minutes).

Pleasingly, reduction of the excess of the phosphitylating agent to half (11.4 equiv.) and alcohol to ¾ (21.4 equiv.) was possible without significant reduction in the conversion. **These results demonstrate that efficient coupling can indeed be maintained at lower equivalents under optimized delivery conditions**. It is well established that, for larger-scale syntheses on optimized automated platforms (e.g., ÄKTA oligosynt), at pilot scale, high conversion can be achieved using reduced equivalents of coupling reagent. Importantly, for expensive or less readily available alcohols and nucleosides, we have implemented straightforward recovery strategies, allowing efficient recycling of unreacted reagents. This minimizes waste and cost for valuable substrates in large-scale synthesis while maintaining robust and simple conditions for small-scale screening.

The following sentence has been added to the revised manuscript:

'While high reagent equivalents were used to ensure complete coupling on small-scale CPG- and PS-supported sequences (for automated synthesis: CPG (1 μmol), 120 equiv. of phosphitylating

agent, 150 equiv. of alcohol; PS (5.25 µmol), 22.9 equiv. of phosphitylating agent, 28.6 equiv. of alcohol), lower equivalents (11.4 equiv. phosphitylating agent, 21.4 equiv. alcohol for 5.25 µmol synthesis on PS support) were also found to lead to efficient reaction without significant reduction in conversion (See Supplementary Information Section 3.3). Importantly, efficient recovery strategies allow recycling of more valuable alcohols and nucleosides. It is well established that, for larger-scale syntheses on optimized automated platforms at pilot scale, high conversion can be achieved using reduced equivalents of coupling reagent.

Comments: Is tetrazole necessary for the reaction of oligonucleotide phosphoramidite with alcohols, or does BTT work as well?

Response: Coupling of the P(III) phosphoramidite with alcohols proceeded with the highest conversion using 1*H*-tetrazole as the activator, although BTT also promoted the reaction with slightly lower coupling efficiency. Efficient phosphoramidite/alcohol coupling requires a balance between activation rate and nucleophile capture; presumably, strong activators can accelerate competing side pathways, whereas 1*H*-tetrazole promotes formation of a more controlled reaction of the tetrazolide intermediate that favours productive nucleophilic substitution. In this context, 1*H*-tetrazole provides an appropriate balance of acidity and nucleophilicity of the conjugate base, enabling efficient generation of the reactive phosphite intermediate.

A parallel reaction on an ABI394 synthesiser (see section 3.3 in the Supporting Information for the method) using a $(dT)_{10}$ sequence confirmed that phosphitylation of **1b** followed by coupling (of the resultant phosphoramidite **2b**) with 9-dec-1-en-1-ol gave 88% conversion with 1*H*-tetrazole (0.45 M), compared to 68% with BTT (0.3 M) (Figure S2).

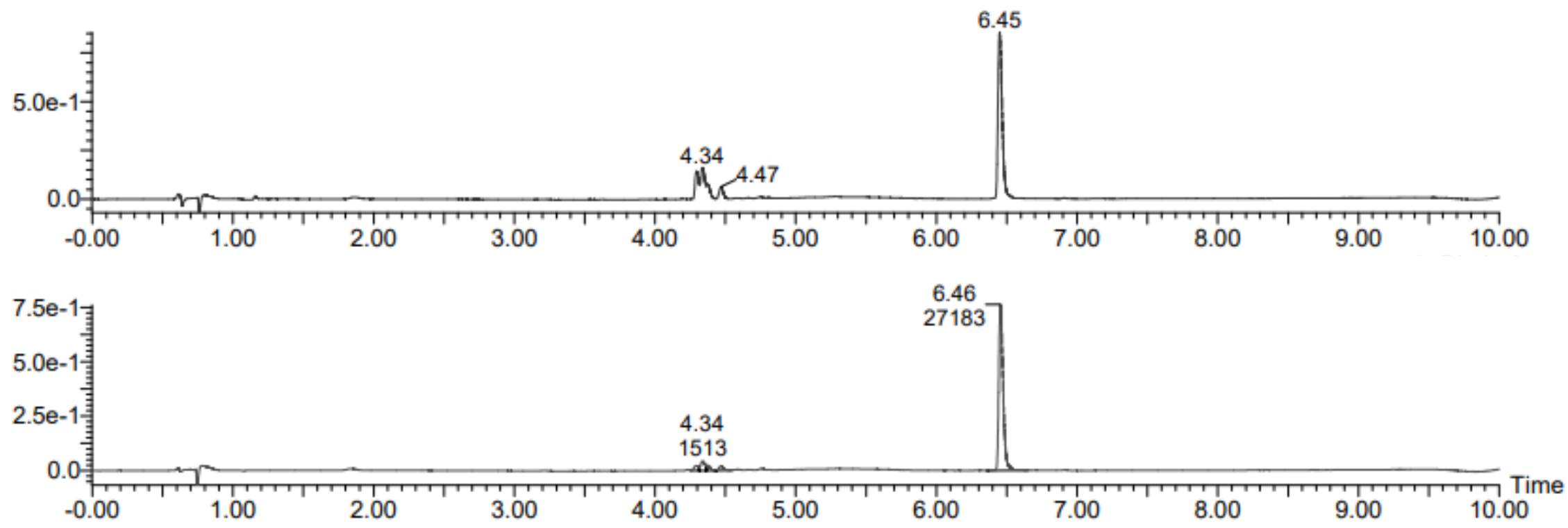

Figure S2. Stacked ion-pair reversed-phase UHPLC chromatograms of the crude reaction mixtures from the coupling of 9-decen-1-ol with P(III) phosphoramidite **2b** using 1*H*-tetrazole (bottom) and BTT (top) as activators (UV absorbance at 260 nm vs. time in minutes).

The following paragraph has been added to the revised Supporting information.
'A parallel reaction on an ABI394 synthesiser using a $(dT)_{10}$ sequence confirmed that phosphitylation of **1b** followed by coupling (of the resultant phosphoramidite **2b**) with 9-dec-1-en-1-ol gave 88% conversion with 1*H*-tetrazole (0.45 M), compared to 68% with BTT (0.3 M). BTT was highly effective as an activator for the phosphitylation of the 5'-OH of the oligonucleotide chain, producing near-quantitative conversion to the phosphoramidite intermediates (e.g., **2b**), whereas 1*H*-tetrazole proved superior for subsequent alcohol coupling. This is likely due to the optimal acidity and controlled reactivity of the tetrazolide intermediate compared to that formed from BTT, which facilitates efficient nucleophilic substitution and higher conversion.'

Comments: Please include more experimental details in the table in paragraph 4 of the Supporting Information, such as coupling reaction time, alcohol excess, and temperature.

Response: We thank the reviewer for this helpful suggestion. Section 4 of the Supporting Information (Figure S3) has now been updated, and the scheme has been revised to include the reagent equivalents as a footnote.

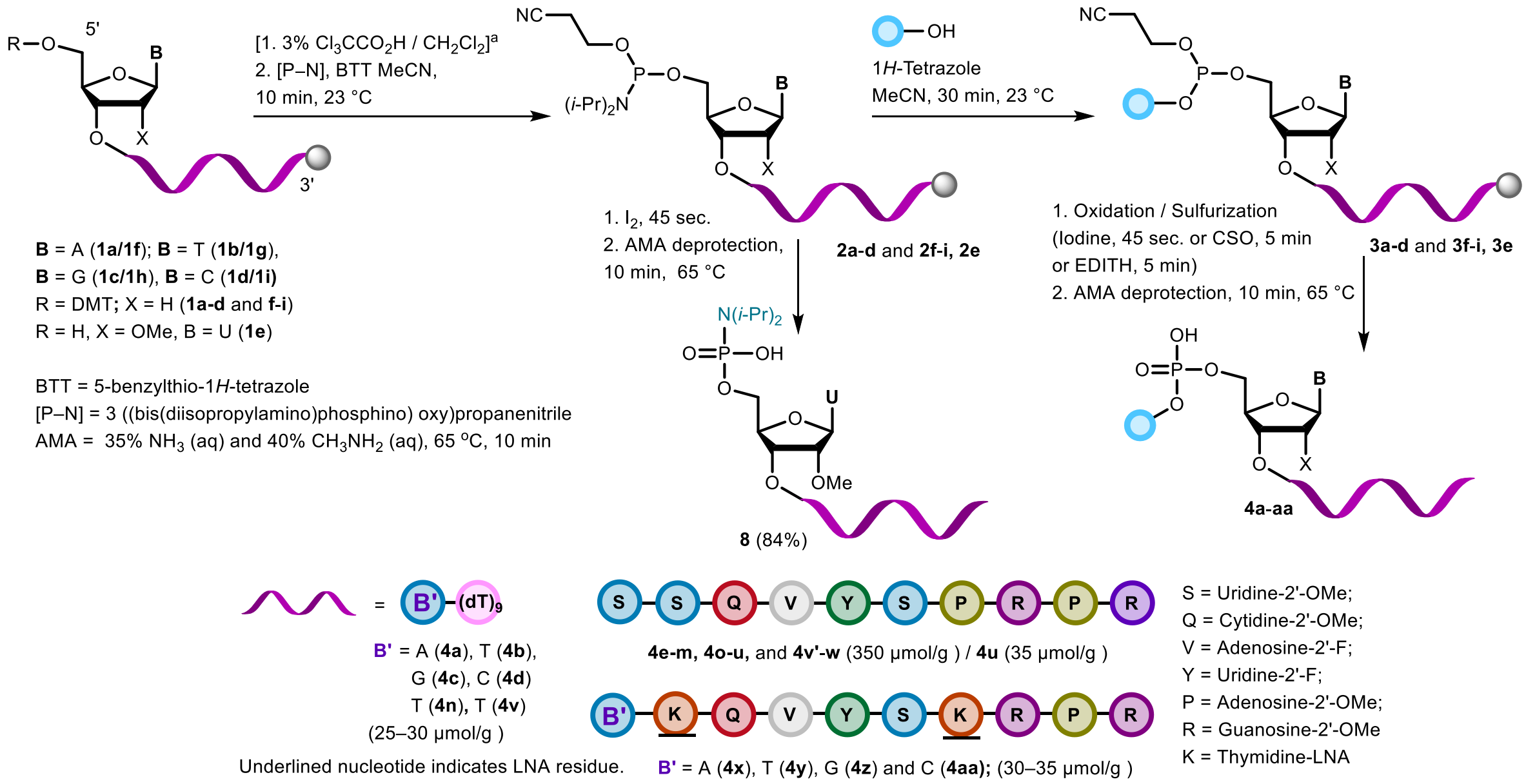

Figure S3. Functionalization at the 5'-position of CPG / mixed-sequence polystyrene-supported oligonucleotides.

For automated synthesis (ABI 394):

i. CPG-supported sequences (1 µmol scale): 120 equiv. of the phosphitylating agent and 150 equiv. of alcohol were employed.

ii. PS-supported sequences (5.25 µmol scale): 22.9 equiv. of the phosphitylating agent and 28.6 equiv. of alcohol were used.

When acetonitrile alone was used to dissolve the alcohol, lower equivalents (e.g., 11.4 equiv. of phosphitylating agent and 21.4 equiv. of alcohol for a 5.25 µmol synthesis on PS-supported sequences were sufficient.

Comments: The reaction of oligonucleotide phosphoramidite on a highly loaded PS support with hexaethylene glycol produced a significant amount of dimeric product. Please compare the product distribution using CPG support or PS with lower loading. Is it possible to avoid or at least minimize the di-functionalized product by using an even higher excess of alcohol?

Response: We thank the reviewer for this insightful comment. To evaluate the influence of support loading and alcohol concentration on product distribution, comparative coupling experiments were performed using lower-loading CPG-supported oligonucleotide sequences (25–35 µmol g^{-1}). Couplings with hexaethylene glycol were carried out in acetonitrile at alcohol concentrations ranging from 0.25 to 3.0 M. We also compared the product distribution between high-loading PS support (350 µmol g^{-1}) and the same sequence on CPG support (35 µmol g^{-1}).

UHPLC analysis of the crude reaction mixtures demonstrated that increasing alcohol concentration progressively suppressed formation of the dimeric product, consistent with increased intermolecular coupling events with the external nucleophile under conditions of high nucleophile excess (Figure

S4). However, elevated alcohol concentrations resulted in a marked decrease in overall coupling efficiency of **4u**, accompanied by increased levels of unreacted phosphoramidite.

Representative product distributions are summarized below.

For the CPG supported mixed sequence (loading 35 µmol/g)

Entry	alcohol (M)	**4u** (%)	**4u**- difunctionalized (%)	Phosphoramidate
1	0.25	59	23	18
2	1	51	4	44
3	2	36	1	62
4	3	24	-	75

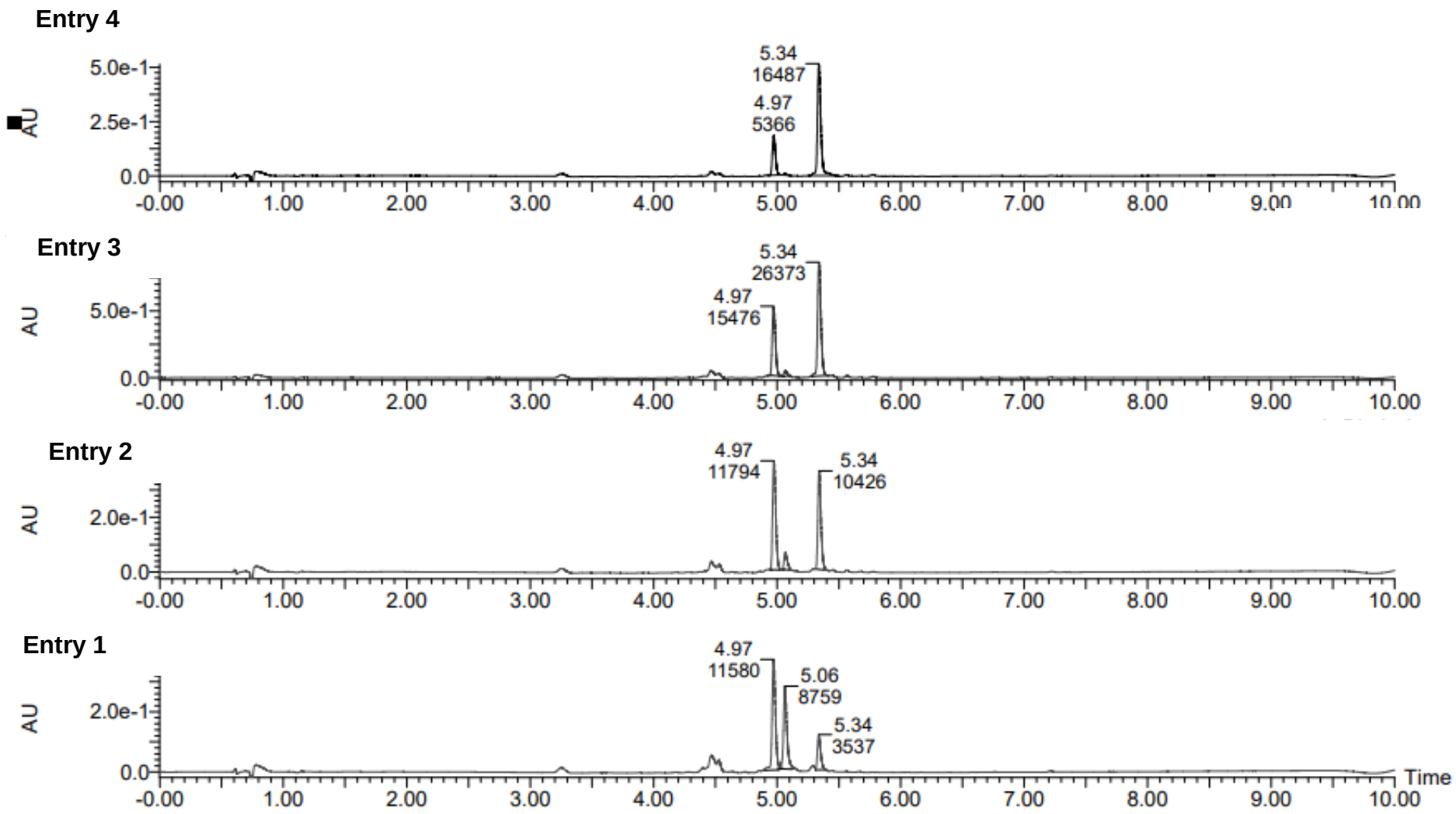

Figure S4. Stacked ion pair reversed-phase UHPLC chromatogram of the crude reaction mixture (UV absorbance at 260 nm *vs*. time in minutes).

Consistent trends were observed when comparing different solid supports. For the high-loading PS support (350 µmol g^{-1}), coupling with a 0.25 M solution of hexaethylene glycol in acetonitrile afforded 53% of the desired oligonucleotide **4u** and 35% of the corresponding dimer. Performing the same reaction on the lower-loading CPG support (35 µmol g^{-1}) under identical conditions gave 59% of **4u** and 23% dimer. These results indicate that the overall distribution of monomeric and dimeric products is largely maintained across different supports, with the lower-loading CPG providing a slight improvement in the ratio of desired monomer to dimer.

Based on these data, an alcohol concentration of 0.25 M was identified as providing the optimal balance between efficient coupling and minimization of intermolecular side reactions for the hexaethylene glycol reaction.

For the PS supported SSQVYSPRPR sequence (350 µmol g⁻¹)

Sequence	rt	Area (A)	Extinction coefficient (ε)	A/ε	Conversion
SSQVYSPRPR	4.813	14377.582	110270	0.1303853	
SSQVYSPRPR-**R**	5.257	46160.59	110270	0.4186142	53%
(SSQVYSPRPR-**R**)$_2$	5.338	30186.652	220540	0.13687608	35%
SSQVYSPRPR	5.615	10910.318	110270	0.098941852	

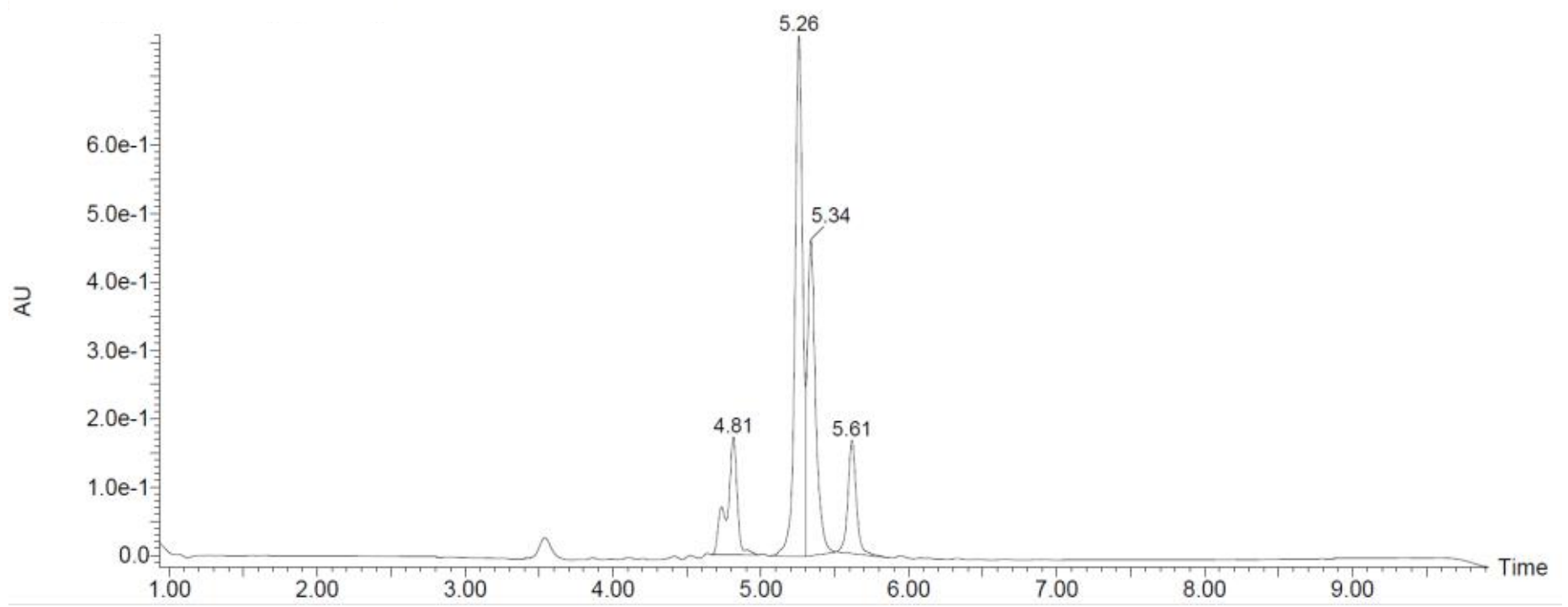

For the CPG supported SSQVYSPRPR sequence (35 µmol g⁻¹)

Sequence	rt	Area (A)	Extinction coefficient (ε)	A/ε	Conversion
SSQVYSPRPR-**R**	4.971	11580.483	110270	0.10501934	59%
(SSQVYSPRPR-**R**)$_2$	5.063	8758.836	220540	0.03971541	23%
SSQVYSPRPR	5.337	3537.372	110270	0.03207919	

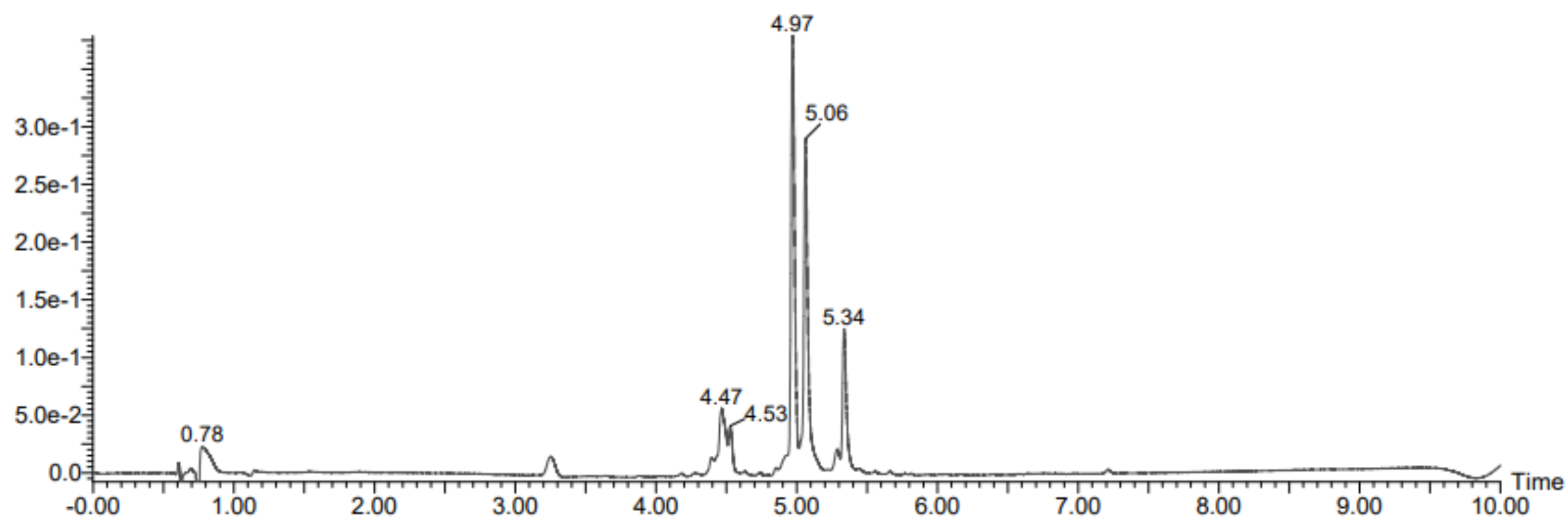

The following sentences have been added to the manuscript:

'Comparative studies using the same sequence on a high-loading PS support (350 µmol g⁻¹) and a lower-loading CPG support (35 µmol g⁻¹) under identical conditions revealed that the overall distribution of monomeric and dimeric products was largely maintained, with the lower-loading CPG showing a slight improvement in the ratio of desired monomer to dimer (Entry 19, Section 4.2 of the Supplementary Information). Further experiments using the lower-loading CPG sequences (35 µmol g⁻¹) with varying concentrations of hexaethylene glycol (0.25–3.0 M) demonstrated that increasing the alcohol concentration progressively suppresses dimer formation, but slightly reduces overall coupling efficiency. Based on these data, an alcohol concentration of 0.25 M was identified as providing the optimal balance between efficient coupling and minimization of intermolecular di-functionalization.'

Reviewer #2 (Comments for the Author):

The authors describe on a straightforward, cost-effective method for oligonucleotide modification via on-support oligonucleotide phosphitylation / *in situ* nucleophilic functionalization using readily available P(III) phosphitylating reagents. The authors' findings are expected to contribute to the advancement of oligonucleotide chemistry-based technologies. The manuscript is well executed and the data appear robust, with no major technical concerns. Nevertheless, the advance reported here is largely incremental, and the implications for the broader scientific community are somewhat limited. Given the journal's emphasis on work of exceptional conceptual novelty and wide interest, I do not believe this study is a strong fit for the journal.

Response: We thank the referee for the evaluation of our manuscript. While we regret that the work did not fully meet the referee's expectations, we believe that the revised manuscript should more clearly demonstrate a significant advance, and a straightforward and efficient synthetic protocol for oligonucleotide modification. We anticipate that this methodology will attract significant interest within the oligonucleotide community and make a meaningful contribution to the field.

Reviewer #3 (Comments for the Author):

This manuscript presents an innovative and practical approach for site-selective functionalization of oligonucleotides via on-support phosphorylation and in situ coupling with stable alcohols or nucleosides. The work addresses significant limitations associated with conventional phosphoramidite-based synthesis, including monomer instability, cost, and limited functional-group tolerance. The methodology is validated across diverse substrates, functional groups, and synthetic directions, and its utility is convincingly demonstrated through the synthesis of a biologically relevant azide-modified antisense oligonucleotide followed by cellular imaging studies. Overall, the manuscript is well written, the experimental results are convincing, and the strategy has clear potential to impact oligonucleotide therapeutic development and chemical biology. In this regard, the work should be of broad interest to researchers working in nucleic acid chemistry, chemical biology, and oligonucleotide therapeutics. However, while the methodology is promising, several aspects would benefit from clarification or further strengthening:

Response: We sincerely thank the referee for their insightful comments and helpful suggestions and appreciate their positive evaluation of our work. The manuscript has been thoroughly revised in response to all remarks, and our detailed point-by-point responses are presented below.

Comments: Although the authors demonstrate modification at multiple positions, the practical limitations of the method are not clearly defined. Providing guidance on substrate classes or functional groups that perform poorly or are incompatible would help readers better evaluate the practical applicability of the strategy.

Response: The optimized conditions revealed high chemoselectivity and up to 95% coupling efficiency for primary alcohols and phenols. In contrast secondary alcohols, exemplified by the 3'-OH of a nucleoside [5'-DMT-dT], exhibited reduced reactivity, resulting in a lower conversion of 82% which is consistent with steric impediment around the nucleophilic centre. Nucleosides bearing additional substitution at the 2'-position (e.g., 2'-1-azidopropane or 2'-propyl methanesulfonate) displayed even lower conversion, presumably due to the further increased steric congestion and conformational constraints. Attempted use of hexadecylamine and 1-decanethiol as nucleophiles for

the phosphoramidite coupling afforded little to no detectable formation of the desired product, with the major species identified as either unreacted phosphoramidite, or its hydrolysis products.

We have added the following sentence to the manuscript to improve discussion of both the scope and limitations of the current strategy:

'Under the optimized conditions, a broad range of oligonucleotide functionalizations was achieved using primary aliphatic alcohol and phenol nucleophiles, with high functional group tolerance and chemoselectivity over other nucleophiles, and with coupling efficiencies of up to 95%. In contrast, secondary alcohols and nucleophiles such as alkyl amines and thiols showed poor conversion, with predominantly unreacted phosphoramidite intermediates observed.'

Comments: Quantitative comparison with existing modification strategies would strengthen the practical positioning of the method. In particular, discussion of efficiency, scalability, or workflow advantages relative to phosphoramidite-based approaches would help clarify when the present method offers decisive benefits.

Response: We thank the reviewer for this valuable suggestion. In the original version of the manuscript, we had included the following in the introduction:

'This synthesis relies on phosphoramidite building blocks, which are intrinsically sensitive and require storage at low temperatures under an inert atmosphere to avoid degradation *via* oxidation and/or hydrolysis. Even when handled under these conditions, their routine use in solution form at ambient temperature inevitably leads to degradation over time *via* both autocatalytic and hydrolytic pathways. In addition, phosphoramidite monomers that are used to assist cell uptake and targeting are expensive and limited in range and availability, and the excess reagent needed to drive the coupling reaction to completion is not easily recovered.'

We have now added additional discussion and comparison as follows:

Introduction: Post-synthetic modifications such as activated ester-based amide bond formation, CuAAC or other click conjugations offer a potential solution to these challenges but can require additional steps such as protecting group manipulation or functional group activation (and associated purifications).

Discussion: Many existing oligonucleotide modification strategies provide ≥98% stepwise coupling efficiency with growing oligonucleotide chains, but are critically dependent on the prior preparation of chemically-modified phosphoramidites. Due to their low shelf-life arising from poor stability towards hydrolysis and oxidation, only a limited number of expensive and unstable phosphoramidites are available, and necessitate protecting group chemistry when non-tolerated functional groups are present. This limits both the scope and flexibility of oligonucleotide functionalization. Here, we developed a straightforward, cost-effective method for oligonucleotide modification *via* on-support oligonucleotide phosphitylation and *in situ* nucleophilic functionalization using ubiquitous P(III) phosphitylating reagents, and an array of readily available alcohol nucleophiles. The advantages of this strategy are emphasized by our ability to directly employ bifunctional nucleophiles such as 6-aminohexyl and 6-thiohexyl, as well as azido-alcohols, which would not be tolerated under the conventional phosphoramidite approach. As a result, our methodology simplifies the workflow of oligonucleotide functionalization and purification, while maintaining coupling efficiencies of up to 95%. Furthermore, the unreacted nucleosides and alcohols used in the coupling step can be

recovered in over 90% yield with high purity through a simple aqueous work-up and extraction protocol, markedly improving recyclability. This methodology therefore provides convenient access to a wide range of terminal and internal oligonucleotide modifications, offering flexibility that cannot be matched by the current provision of modified phosphoramidites.

Comments: On line 53, the authors state that their strategy enables “site-specific terminal and internal oligonucleotide modification.” However, the experimental evidence largely demonstrates terminal modification. The only example of internal modification relies on a diol substrate (Entry 19), which inherently generates symmetric sequences and therefore cannot be generalized for arbitrary internal modification. Consequently, the claim regarding general internal modification appears overstated and should be moderated or further supported.

Response: To demonstrate internal modification, the 5'-OH of **1e** was phosphitylated under optimized conditions and coupled with DMTO-PEG$_6$-OH (Figure S5). Subsequent detritylation with 3% trichloroacetic acid followed by coupling with DMT-dT phosphoramidite under standard conditions afforded **4ab** in 75% conversion. This result demonstrates that the methodology is indeed effective for internal modification under the described conditions.

The following statement has been added to the manuscript.
‘After establishing the protocol for terminal modifications, we sought to evaluate whether the methodology could be extended to site-selective internal modifications. We therefore investigated the phosphitylation of the 5'-OH of **1e**, followed by coupling with DMTO-PEG$_6$-OH (Fig. 3a, Section 5 of the Supplementary Information). Subsequent detritylation using 3% trichloroacetic acid was followed by DMT-dT phosphoramidite coupling, affording **4ab** in 77% conversion (over two steps). This result demonstrates that the strategy is effective for controlled internal functionalization.’

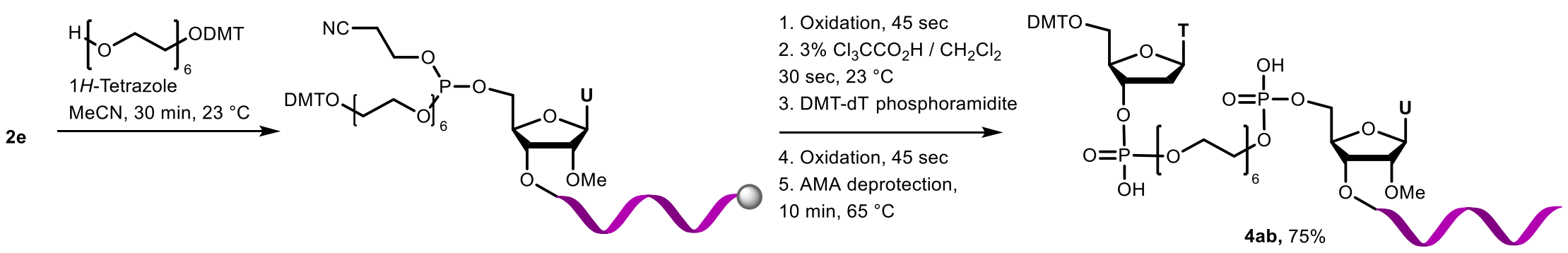

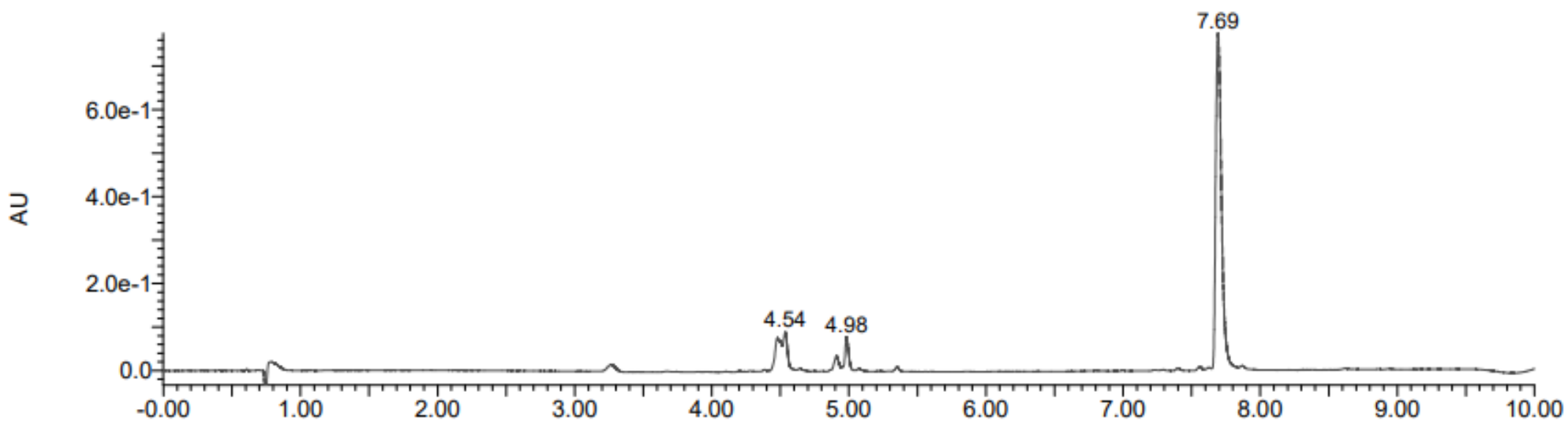

Figure S5. Internal modification of PS-supported oligonucleotides using DMTO-PEG$_6$-OH (top). Stacked ion pair reversed-phase UHPLC chromatogram of the crude reaction mixture of **4ab** oligonucleotide (UV absorbance at 260 nm *vs*. time in minutes) (bottom).

Comments: In lines 69–74, the authors state that conventional phosphoramidite chemistry is "poorly tolerant of certain functional groups." To better highlight the advance of the present method, the authors should clarify the mechanistic origin of these incompatibilities and explicitly explain how the current strategy circumvents these limitations.

Response: We thank the reviewer for this point. Conventional phosphoramidite chemistry often requires reactive functional groups such as amines to be protected, because the highly electrophilic phosphoramidite intermediates can undergo undesired side reactions with nucleophilic or redox-sensitive moieties. As outlined in the manuscript and detailed in Section 9.1 of the Supporting Information, challenges associated with the reaction of azido phosphoramidites, including potential Staudinger-like reduction, further limit the compatibility of conventional methods. Our strategy circumvents these limitations as nucleophilic attack by the primary alcohol outcompetes side reactions of azide and free amine groups. This approach therefore avoids the need for protecting groups, and enhances functional group tolerance.

We have extended our discussion, and the following paragraph has been added to the revised manuscript:

'This is important, since the current 'phosphoramidite monomer' approach is poorly tolerant of certain functional groups, which restricts the diversity and tunability of oligonucleotide conjugates and, consequently, the physicochemical properties and biological activity of modified oligonucleotides. The introduction of an azide functionality is highly desirable for alkyne-based bioconjugation, however it remains challenging to achieve via traditional solid-phase oligonucleotide synthesis due to the incompatibility of azides with the P(III) phosphoramidite reagents typically employed during coupling. Likewise, the installation of free amine groups generally requires amine protection during synthesis to prevent side reactions during the coupling step. This reduces synthetic efficiency and results in burdensome purification requirements. Our strategy circumvents these limitations because nucleophilic attack on the phosphoramidite by the primary alcohol outcompetes potential side reactions involving azide or free amine functionalities. This chemoselectivity eliminates the need for protecting groups and enables improved functional group tolerance.'

Comments: Since the present strategy focuses on oligonucleotide modification rather than oligonucleotide synthesis, comparison with phosphoramidite monomer synthesis methods is not entirely appropriate. A more informative comparison would be with established strategies specifically developed for terminal or post-synthetic oligonucleotide modification.

Response: We thank the reviewer for this comment. To address this, we have added the following paragraph in the introduction section of the revised manuscript.

'Post-synthetic modifications such as ester-based amide bond formation, CuAAC or other click conjugations offer a potential solution to these challenges, but can require additional steps such as protecting group manipulation or functional group activation (and associated purifications).'

Comments: In Figure 4, the depicted structures for the coupling products of **1e** with phosphorodiamidite **6a** (or **6b**) appear inconsistent with the expected products. The authors are encouraged to carefully re-examine and correct these structures if necessary.

Response: We thank the reviewer for this comment and sincerely apologize for the error in the intermediate phosphoramidite structures. The structures have now been corrected and the scheme clarified, and the updated version has been included in the revised manuscript (Figure S6).

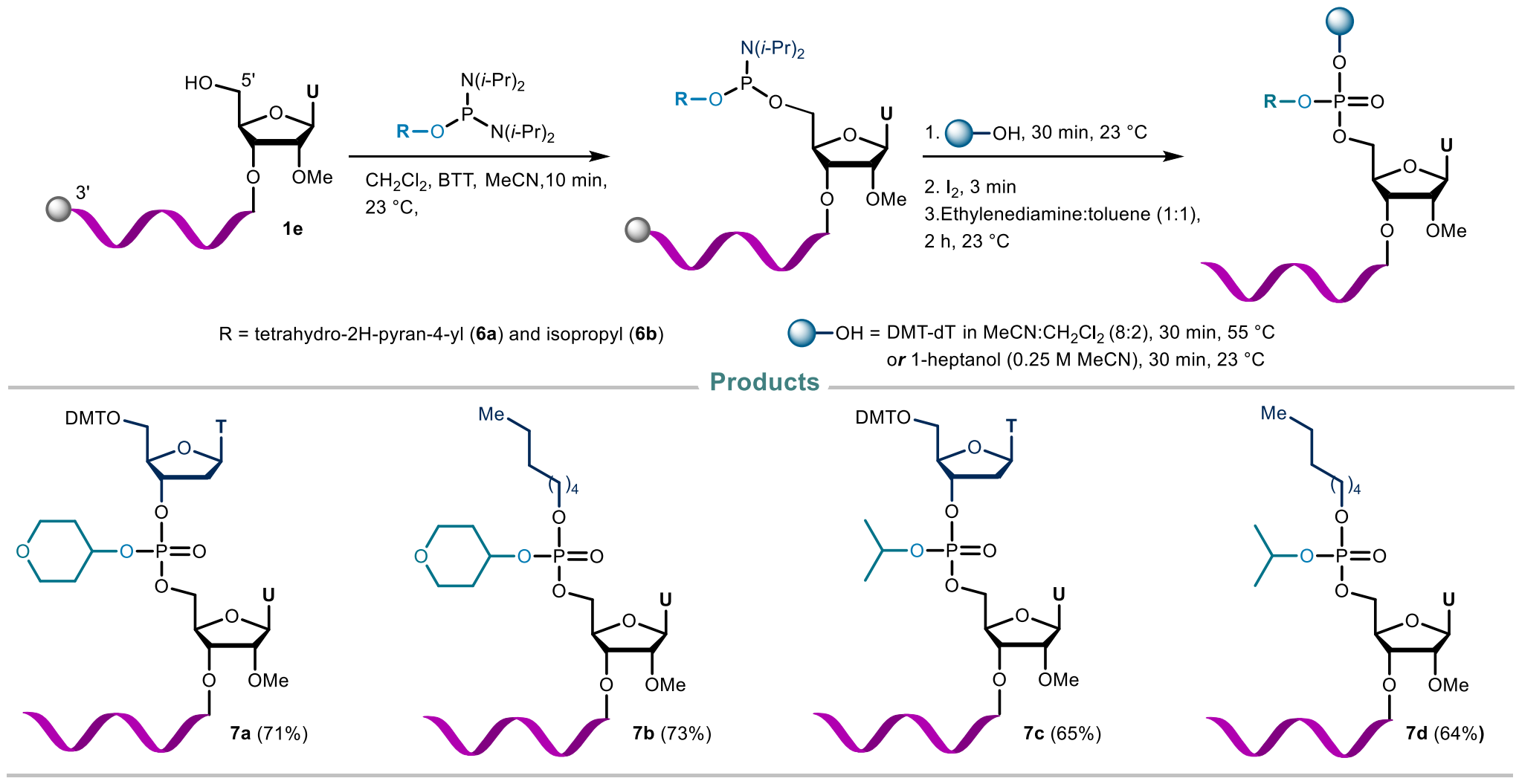

Figure S6. Synthesis of oligonucleotides containing a phosphotriester backbone.

Comments: The current study demonstrates successful incorporation of various alcohol substrates. Exploring whether other nucleophiles, such as thiols, phenols, or amines, could also be accommodated would further broaden the utility of the method. Even preliminary data or discussion would be valuable.

Response: We thank the reviewer for this constructive suggestion. Pleasingly, when aromatic alcohols such as phenols were employed for coupling with the corresponding *in situ*-formed phosphoramidite **2e**, the phenol-coupled oligonucleotide **4z** was obtained in near-quantitative conversion. Both CPG- and PS-supported sequences were tested, affording the corresponding phenol-coupled phosphotriesters **4z** and **4z'** (in 95% and 94% conversion, respectively (Figure S7). The lipophilic δ-tocopherol moiety, valuable for enhancing membrane association and delivery in therapeutic oligonucleotides, was successfully incorporated using our method, affording the modified oligonucleotide **4w** (Entry 21) in 90% conversion.

We also evaluated hexadecylamine and 1-decanethiol as nucleophiles. These cases resulted in poor conversion to the desired oligonucleotides, with the major products identified as either unreacted phosphoramidite or its hydrolysis products. We have included a brief discussion of this point when addressing the limitations of our current methodology.

The following statement has been added to the revised manuscript:

'We next sought to evaluate the reactivity of aromatic alcohols. Pleasingly, phenols also coupled efficiently with the in situ-formed phosphoramidite **2b**/**2e**, delivering phenol-functionalized oligonucleotides **4v**/**4v'** in near-quantitative conversion (95% and 94% for CPG- and PS- supported mixed sequences, respectively; Entry 20). Furthermore, the lipophilic δ-tocopherol moiety, an important motif used to enhance membrane association and delivery of therapeutic oligonucleotides,

was successfully incorporated, affording the δ-tocopherol-modified oligonucleotide **4w** in 90% conversion (Entry 21) and further demonstrating the robustness of the methodology.'

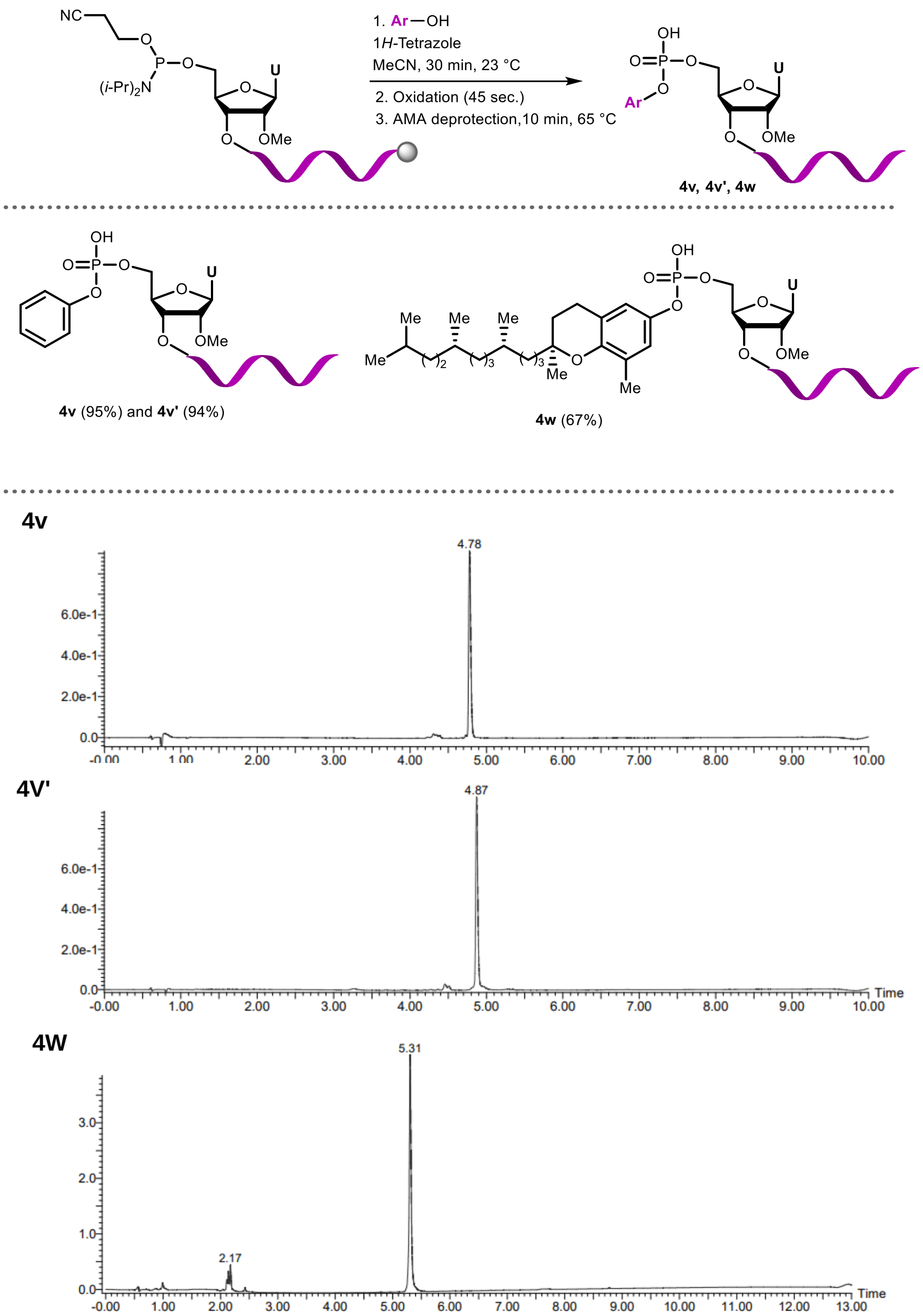

Figure S7. **a.** 5′-Functionalization of CPG- and PS-based mixed-sequence oligonucleotides using phenols. **b–d.** Ion-pair reversed-phase UHPLC chromatograms of the crude reaction mixtures for phenol-coupled oligonucleotides: **b. 4z, c. 4z′**, and **d.** δ-tocopherol–modified oligonucleotide **4w** (UV absorbance at 260 nm vs. time in minutes).

Comments: In the experiments evaluating terminal nucleobase tolerance (Entries 1–4), only poly-T sequences are employed, whereas more structurally complex sequences appear only in later examples involving uracil termini. Inclusion of more sequence-diverse examples in these initial tests would strengthen confidence in the generality of the approach.

Response: We thank the reviewer for this suggestion. To address it, we further evaluated terminal nucleobase tolerance using more structurally diverse sequences in addition to poly-T. A CPG-based sequence containing LNA, 2'-*O*-Me, and 2'-F modifications with different terminal bases [A (**1f**), T

(**1g**), G (**1h**), and C (**1i**)], which is structurally more complex and includes both LNA and 2'-substitutions, was prepared and investigated. Using our optimized reaction conditions, coupling of the respective phosphoramidite intermediates (**2f-i**) with decan-1-ol afforded the desired oligonucleotides in 90–91% conversion (Figure S8).
The following paragraph has been now added to the revised manuscript:

'To further evaluate the generality of the method, we investigated terminal nucleobase variation using other structurally diverse sequences. A CPG based sequence (30–35 µmol g⁻¹), containing an LNA 2'-*O*-Me, and 2'-F modifications, was prepared with different terminal bases [A (**1f**), T (**1g**), G (**1h**), and C (**1i**)] and subjected to the optimized reaction conditions using decan-1-ol as nucleophile. Once again, the desired oligonucleotides (**4x–aa**; Entry 22) were obtained in near-quantitative conversion (90–91%), demonstrating the broad applicability of this strategy across diverse nucleobase contexts and complex sequences.'

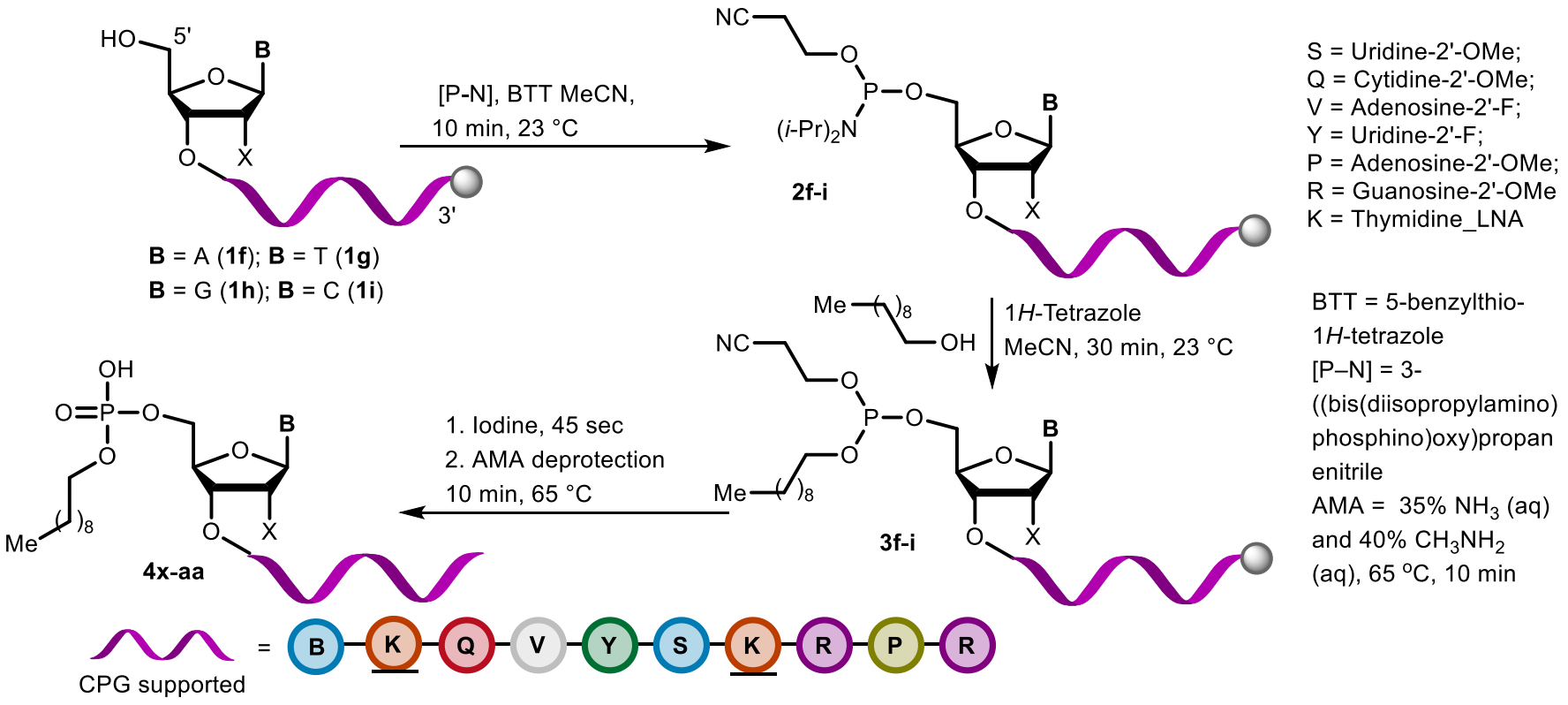

Entry	Solid Support	[B]	Alcohol	[Ox]	Product	Conversion (%)
1[a]	CPG	A	decan-1-ol	I_2	**4x**	90
2[a]	CPG	T	decan-1-ol	I_2	**4y**	91
3[a]	CPG	G	decan-1-ol	I_2	**4z**	91
4[a]	CPG	C	decan-1-ol	I_2	**4aa**	90

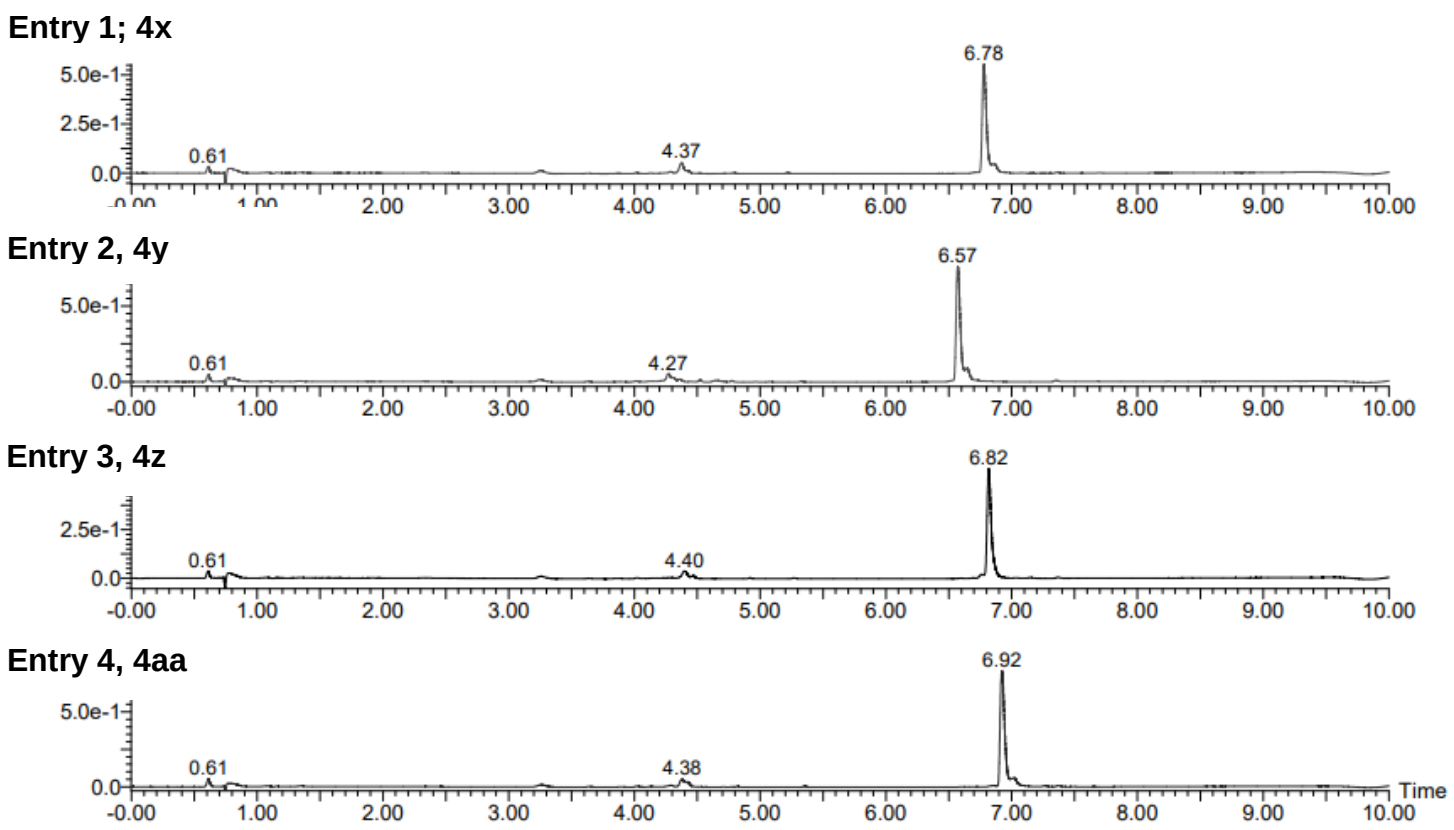

Figure S8. Functionalization at the 5'-position of mixed-sequence CPG-supported oligonucleotides (top). Stacked ion pair reversed-phase UHPLC chromatogram of the crude reaction mixture of **4x-aa,** (UV absorbance at 260 nm *vs*. time in minutes) (bottom).

Comments: If the system can tolerate more complex delivery or targeting motifs, such as lipid-derived functionalities, inclusion of such examples would further enhance the translational relevance of the method.

Response: We thank the reviewer for these constructive suggestions. To explore the potential for incorporating complex lipidic motifs, we evaluated the coupling efficiencies of 1-tetradecanol, oleyl alcohol, and δ-tocopherol. While 1-tetradecanol and oleyl alcohol exhibited high coupling efficiencies of 84% and 88%. Notably, δ-tocopherol, a biologically relevant lipophilic motif widely used in nucleic acid research, coupled efficiently with phosphoramidite **2e**, affording 90% conversion (Figure S9). Overall, these results demonstrate that the methodology is compatible with sterically demanding, hydrophobic motifs, highlighting its potential for translational applications.

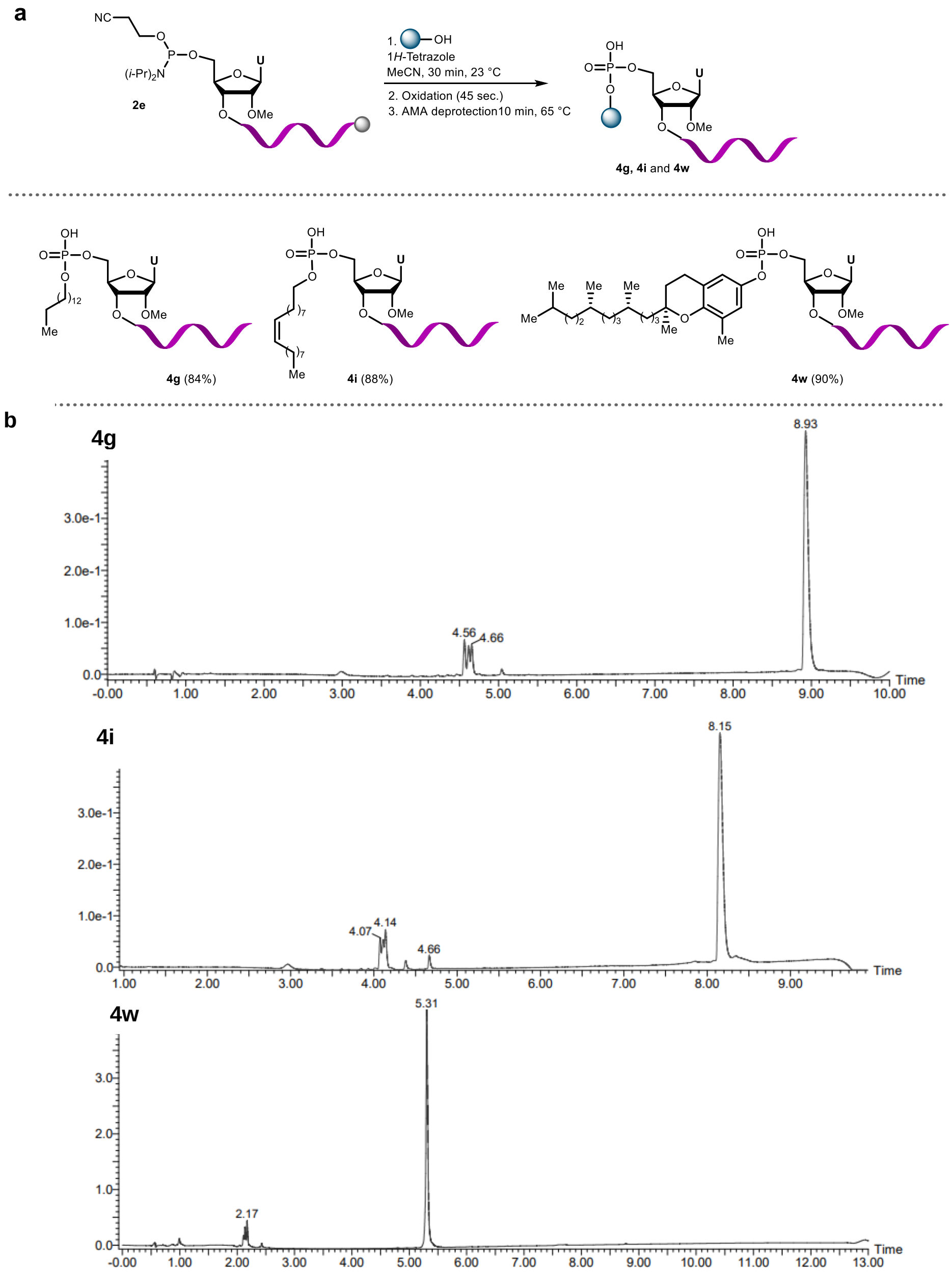

Figure S9. a,5'-Functionalization of mixed-sequence polystyrene-supported oligonucleotides with lipidic alcohols. b, Ion pair reversed-phase UHPLC chromatogram of the crude reaction mixture of 1-tetradecanol (**4g**), *cis*-9-octadecen-1-ol (**4i**) and , δ-tocopherol (**4w**). UV absorbance at 260 nm *vs*. time in minutes).

Comments: Phosphorodiamidate morpholino oligomers (PMOs) represent another important therapeutic oligonucleotide backbone. It remains unclear whether the present methodology is applicable to PMO systems. Discussion or preliminary experiments addressing this point would substantially increase the practical value of the study.

Response: We thank the reviewer for this valuable suggestion. PMOs represent an important class of therapeutic oligonucleotides; however, extending the present methodology to PMO synthesis is non-trivial. This is due to the substantial differences between the chemistry normally employed in PMO synthesis [P(V) chemistry] and the conventional phosphoramidite-based [P(III)] oligonucleotide synthesis cycle. Adapting our protocol would therefore require extensive re-optimization and we respectfully feel is beyond the scope of the current study. Nonetheless, we recognize this as an attractive avenue for future investigation.
